# Coordination Chemistry of Solvated Metal Ions in Soft Donor Solvents

**DOI:** 10.3390/molecules30153063

**Published:** 2025-07-22

**Authors:** Kersti B. Nilsson, Mikhail Maliarik, Ingmar Persson

**Affiliations:** 1 Rejlers Sverige AB, Stationsgatan 12, SE-753 40 Uppsala, Sweden; kersti.nilsson@rejlers.se; 2 Department of Molecular Sciences, Swedish University of Agricultural Sciences, P.O. Box 7015, SE-750 07 Uppsala, Sweden; 3 Metso (Sweden) AB, Gymnasievägen 26 A, SE-931 27 Skellefteå, Sweden; mikhail.maliarik@metso.com; 4 Department of Chemistry, Royal Institute of Technology, SE-100 44 Stockholm, Sweden

**Keywords:** soft donor solvents, metal ion solvates, coordination chemistry, covalent bonding estimate, liquid ammonia

## Abstract

The structures of hexaammine solvated indium(III) and thallium(III) ions in liquid ammonia solution are determined by EXAFS. Both complexes have regular octahedral coordination geometry with mean In-N and Tl-N bond distances of 2.23(1) and 2.29(2) Å, respectively. Ammine solvated thallium(III) in liquid ammonia is characterized with ^205^Tl NMR measurements. Solvents such as liquid ammonia, N,N-dimethylthioformamide (DMTF), trialkyl and triphenyl phosphite and phosphine are strong electron pair donors and thereby able to form bonds with a large covalent contribution with strong electron pair acceptors. A survey of reported structures of ammine, DMTF, trialkyl and triphenyl phosphite and phosphine solvated metal ions in the solid state and solution is presented. The M-N and M-S bond distances in ammine and DMTF solvated metal ions are compared with the M-O bond distance in the corresponding metal ion hydrates, expected to form mainly electrostatic interactions with metal ions. The d^10^ metal ions have high ability to form bonds with a high degree of covalency with increasing ability down the group and with decreasing charge of the metal ion. The difference in M-N and M-O bond distances between ammine solvated and hydrated metal ions with the same coordination geometry decreases significantly with the increasing ability of the metal ion to form bonds with a large covalent contribution. This difference correlates well with the covalent bonding index, *γ*_M_^2^*r.

## 1. Introduction

Current knowledge about the coordination chemistry of solvated metal ions is dominated by oxygen donor solvents as they are by far the most common ones [1]. As oxygen has high electronegativity [2], M-O bonds are mainly electrostatic in their character. This means that the coordination number in such metal ion solvates is mainly given by the ratio of the metal ion radius and the atomic radius of oxygen in the solvent molecule to balance the attraction between the positively charged metal ion and the negative end of molecule dipoles and the repulsion between the latter. This often results in coordination geometries with high symmetry such as tetrahedrons, octahedrons and square antiprisms. The predicted metal ion radii in different coordination geometries are summarized by Shannon [3], and the atomic radius of oxygen in coordinated water molecules has been estimated to be 1.34 Å [4]. The atomic radius of oxygen in oxygen donor solvents may vary within a couple of hundredths of an Å, but only in a limited number of cases will it affect the coordination number and geometry of the metal ion. One such example is lanthanoid(III) ions, which are nine-coordinated in a tricapped trigonal prismatic fashion in aqueous solution and most solid hydrates [5], while in *N*,*N*-dimethylformamide (DMF) solvates, they have coordination numbers between eight and nine (Table S1), and in dimethylsulfoxide (DMSO) solvates, they are eight-coordinated in both solution and the solid state [6]. Another factor with impact on the coordination number is the presence of chemical groups in close vicinity to the coordinating donor atom. This may cause steric restrictions that affect coordination to a metal ion. The bulkiness around the coordinating oxygen atom in *N,N*’-dimethylpropyleneurea reduces the coordination number in many metal ion complexes [7].

The binding properties of metal ions were previously systemized into class (a) and (b), depending on whether they formed the strongest complexes in solution with ligands with donor atoms in the order O > S < Se > Te, N > P > As > Sb or F^−^ >> CL^−^ > Br^−^ > I^−^ and O << S < Se < Te, N << P > As or F^−^ << Cl^−^ < Br^−^ < I^−^, respectively [8]. This view was further developed by Pearson who introduced the concept of hard Lewis acids (electron pair acceptors) for class (a) metal ions and soft Lewis acids for class (b) metal ions. The ligands were classified in the same way, with ligands with oxygen donor atoms, fluoride, ammonia and amines being classed as hard Lewis bases (electron pair donors) and ligands with sulfur, phosphorus, carbon donor atoms, and iodide and bromide being classed as soft Lewis bases [9,10].

In order to further tune the electron pair donor properties of solvents, a number of scales have been introduced to estimate the electron pair donor ability of solvents to participate in covalent bonding in an electron acceptor (metal ion)–electron donor (solvent) bond [11]. Such scales are fully dependent on the bonding character of the electron acceptor used in the scale. The first of these scales was the donor number concept, *D*_N_, proposed by Gutmann et al. [12]. They used antimony(V) chloride as a probe in dilute 1,2-dichloroethane solution; the solvent under study formed an adduct complex with antimony(V) chloride. The *D*_N_ value of a solvent is the heat of adduct formation in kcal∙mol^−1^. The main drawback with the *D*_N_ concept is that solvents forming strong covalent bonds decompose the probe. Later, the donor strength scale, *D*_S_, was introduced. The *D*_S_ value is the difference in the symmetric Hg-Br bond stretching frequency in cm^−1^ of solvated mercury(II) bromide in the neat solvent, *ν*(Hg-Br)_solvent_, and the stretching frequency of HgBr_2_ in the gas phase, *ν*(Hg-Br)_gas_ [11]. The *D*_S_ concept can be applied in all kinds of solvents without any restrictions. The *D*_S_ scale shows that the softest donor solvents are trialkyl phosphines (PR_3_) and phosphites (P(OR)_3_), liquid ammonia (NH_3_), alkyl amines (RNH_2_) and the sulfur donor solvent *N,N*-dimethylthioformamide (DMTF) [11]. Of these solvents, only liquid ammonia and DMTF have sufficiently high permittivity to allow electrolytes to be dissolved and dissociated and well-defined metal ion solvates to form. There is a vast amount of structure determinations of ammine solvated metal ions reported in the solid state, while for DMTF, trialkyl and triphenyl phosphines and phosphites, only a limited number of metal ion solvates in the solid state or solution have been reported (Tables S2–S4). Due to the low permittivity of the phosphorus donor solvents, only monovalent 1:1 electrolytes with a soft metal ion are able to dissolve and dissociate.

The aim of this study is to give an overview of the structures of metal ion solvates in solution and the solid state with soft donor solvents. The difference in M-N and M-O bond distances between ammine solvated and hydrated metal ions with the same coordination geometry will be presented as this shows important differences in the bonding properties and coordination geometry of metal ions. The impact that the coordination chemistry of soft donor solvents has on especially soft metal ions will be presented, discussed and correlated with a previously proposed covalent bonding index. The structures of the ammine solvated indium(III) and thallium(III) ions in liquid ammonia are reported as the number of studies on ammine solvated trivalent metal ions is limited, and the bonding properties of group 3A metal ions change from hard, aluminum(III), to soft, thallium(III).

## 2. Results and Discussion

### 2.1. EXAFS on the Ammine Solvated Indium and Thallium(III) Ions in Liquid Ammonia

**Figure 1:**
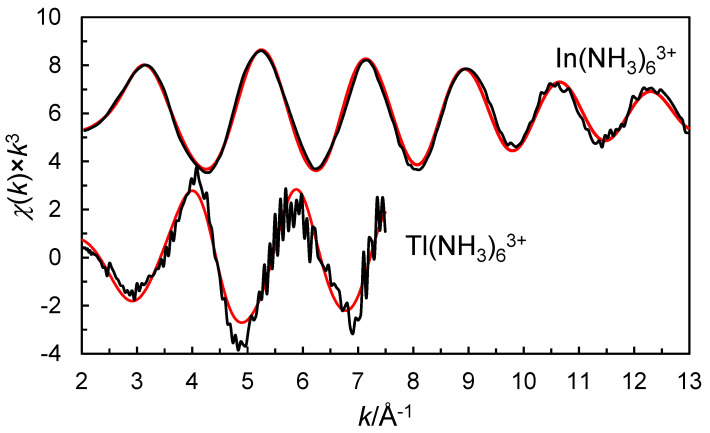
*k*^3^-weighted EXAFS data of experimental (black line) and theoretical (red line) data of the ammonia solvated indium(III) (offset 6) and thallium(III) ion (no offset) in liquid ammonia.

**Figure 2:**
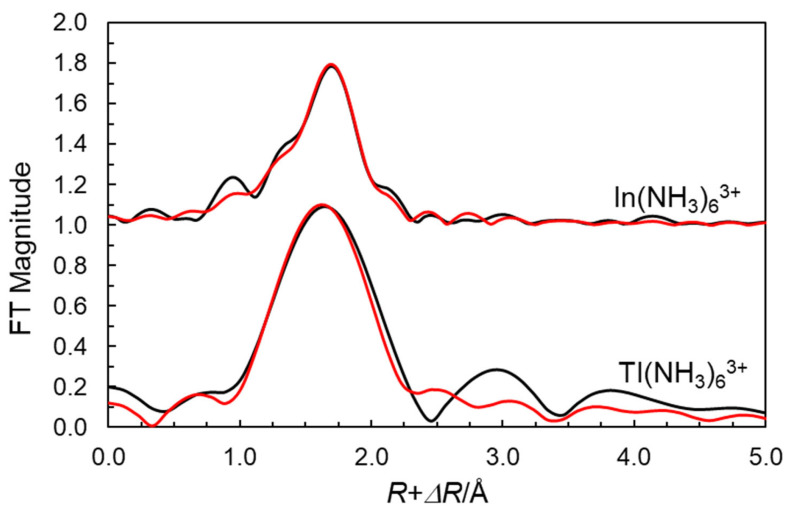
Fourier transforms of EXAFS data of experimental (black line) and theoretical (red line) data of the ammonia solvated indium(III) (offset 1.0) thallium(III) (no offset) ions in liquid ammonia.

The refinement of the EXAFS spectrum of indium(III) perchlorate in liquid ammonia gives an In-N bond distance of 2.232(7) Å and a multiple scattering signal at twice the In-N bond distance of 4.45(12) Å, supporting an octahedral configuration around indium (Table 1). The refinement of the EXAFS spectrum of the liquid ammonia solution of thallium(III) perchlorate gives a Tl-N bond distance of 2.29(2) Å and a multiple scattering signal at twice the Tl-N bond distance of 4.60(8) Å (Table 1), which supports an octahedral configuration also around thallium(III). The fittings of the EXAFS functions and the Fourier transforms are given in Figure 1 and Figure 2.

### 2.2. ^205^Tl NMR Characterization of the Ammine Solvated Thallium(III) Ion in Liquid Ammonia

The ^205^Tl NMR spectrum of the ammine solvated thallium(III) ion in liquid ammonia results in the appearance of a single narrow resonance (cf. Table 2 and Figure S1). The chemical shift in the signal, 2769 ppm, is indicative of the oxidation state of thallium(III) [13,14]. This chemical shift appears to be close to the resonances of some other thallium(III) complexes with soft nitrogen donor ligands, ethylenediamine (en) and diethylenetriamine (dien) in pyridine solution, [Tl(en)_3_]^3+^ (2889 ppm) [15] and [Tl(dien)_2_]^3+^ (2692 ppm) [16]. In these complexes, the configuration around the thallium(III) ion is made up of six nitrogen atoms in somewhat distorted octahedra. Taking into account the very large range of ^205^Tl chemical shifts (over 3000 ppm) [13,14], the *δ* value of the ^205^Tl signal in the liquid ammonia solution points to a similar coordination environment of the thallium(III) ion. One can therefore assume that a more or less regular octahedral [Tl(NH_3_)_6_]^3+^ complex is present in the liquid ammonia solution of thallium(III) perchlorate.

The spin–lattice relaxation times (*T*_1_) of the ^205^Tl nucleus in a number of thallium(III) complexes in solution have been determined previously [17]. At the same concentration of the complexes (0.05 mol∙dm^−3^), applied magnetic field (288.5 MHz), and temperature (298 K), octahedral, [Tl(OH_2_)_6_]^3+^, and tetrahedral, [Tl(CN)_4_]^−^, species have essentially the same *T*_1_ values, 0.91 ± 0.08 and 1.09 ± 0.03 s, respectively, which are in excellent agreement with previously reported values, (0.93 ± 0.07) and 0.96 ± 0.02 [18], respectively. As it is expected for these species to have cubic symmetry (*O*_h_ and *T*_d_), the chemical shift anisotropy (Δ*σ*) of the species has been found to be close to zero [18]. By decreasing the symmetry from *O*_h_ to, e.g., *C*_4v_ ([TlCl(OH_2_)_5_]^2+^), this results in a sharp drop in the *T*_1_ value, 0.06 ± 0.01 s, while the Δ*σ* value increases to 1300 ppm [18]. The ^205^Tl spin–lattice relaxation time determined in this work for the ammine solvated thallium(III) ion in liquid ammonia solution is 0.51 ± 0.01 s. This is about a half of the relaxation time of the ^205^Tl nuclei in [Tl(NH_3_)_6_]^3+^ compared to [Tl(OH_2_)_6_]^3+^ that may be attributed to some distortions of the octahedron rather than a substantial decrease in the symmetry. A scalar coupling relaxation mechanism, via coupling to rapidly relaxing ^14^N nuclei of the solvated ammonia molecules, can also contribute to shortening of the ^205^Tl relaxation time in this case.

The lack of either the ^15^N or ^1^H spin–spin coupling of the ^205^Tl signal can be attributed to very fast exchange of ammonia molecules between the first solvation shell and bulk ammonia. This is further supported by the lack of a separate signal of the [Tl(NH_3_)_6_]^3+^ species in ^1^H NMR spectra of the solution; the single resonance of NH_3_ is observed at 1.15 ppm (6 Hz).

^205^Tl chemical shifts in monovalent thallium are usually found in the range between –200 and +200 ppm [13,17]. Chemical shifts in the thallium(I) ion in both aqueous and liquid ammonia seem to be notable exceptions since ^205^Tl resonance is strongly shifted to higher frequency; see Table 2. It should be noted that there is over a 1000 ppm difference in the values of the ^205^Tl chemical shift in the thallium(I) ion in aqueous and liquid ammonia. This should be clearly related to significant differences in the coordination environment, configuration and bond strength of the Tl^I^–solvate bond.

### 2.3. Structure Relationship Between Hydrated and Ammine Solvated Metal Ions

**Figure 3:**
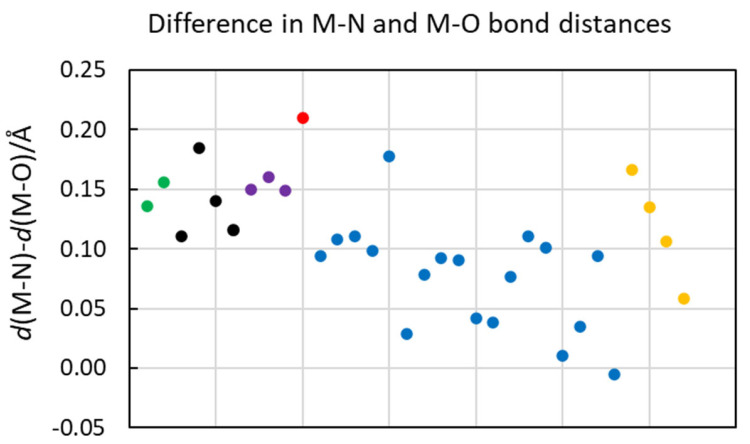
Illustration of difference in M-N bond distances in metal ion ammine solvates and M-O in metal ion hydrates with same coordination number and figure presented in Table 3; Li^+^ and Na^+^ ions (green dots), Mg^2+^, Ca^2+^, Sr^2+^ and Ba^2+^ ions (black dots), La^3+^, Sm^3+^ and Yb^3+^ ions (purple dots), zirconium (IV) ion (red dot), transition metal ions (blue dots) and Al^3+^, Ga^3+^, In^3+^ and Tl^3+^ group 3A metal ions (yellow dots).

**Figure 4:**
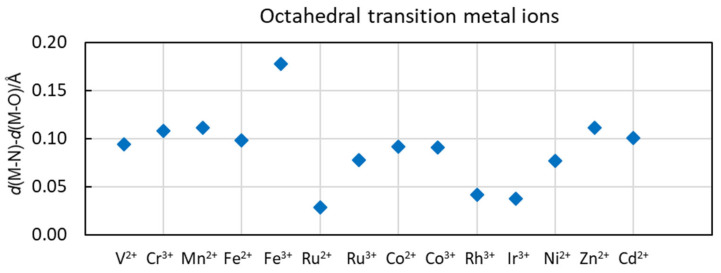
Difference in M-N and M-O bond distances in octahedral transition metal ion ammine solvates and hydrates.

The M-O and M-N bond distances in the structures of the hydrated and ammine solvated metal ions in the solid state and aqueous and liquid ammonia solution, respectively, are summarized in Table 3 and Figure 3 and Figure 4. It is clearly seen that the M-N bond distances in ammine solvated metal ions are longer than the M-O bond distance in the corresponding metal ion hydrate with the same coordination number. This is expected as the atomic radius of three-coordinated oxygen in the oxidation state -II, 1.36 Å (as water), is shorter than the one of four-coordinated nitrogen in the oxidation state -III, 1.46 Å (as ammonia) [3]. The difference in M-N versus M-O bond distance is the largest for typical hard metal ions forming mainly electrostatic bonds, but it decreases sharply with the increasing softness of the metal ion and thus with an increasing covalent contribution to the metal ion–solvate bond (Figure 3, Figure 4, Figure S2 and Figure S3). This becomes obvious for group 3A metal ions, with the typically hard electron pair acceptor aluminum(III) having a difference of 0.17 Å compared to the fairly soft electron pair acceptor thallium(III) with a difference of 0.06 Å at the end of the group, and gallium(III) and indium(III) ions in between (Table 3 and Figure 3 and Figure S2). Alkaline, alkaline earth and lanthanoid(III) ions are regarded as typically hard metal ions with *d*(M-N)–*d*(M-O) differences in ca. 0.15 Å, while for most of the transition metal ions, the difference is ca. 0.10 Å. The exceptions are iron(III) with a difference of 0.18 Å, while for the regarded soft metal ions ruthenium(II), rhodium(III) and iridium(III), the difference is less than 0.05 Å (Table 3 and Figure 3 and Figure S3). This is also true for soft palladium(II) and platinum(II) ions with square planar coordination (Table 3 and Figure S3). This shows that with increasing electron overlap in the metal ion–solvent bond, it becomes significantly shorter than expected from the ionic radius of the metal ions and the atomic radius of nitrogen.

### 2.4. Overview of Solvate Structures of d^10^ Metal Ions with Nitrogen Solvents

#### 2.4.1. Ammine, Acetonitrile and Pyridine Solvated Copper(I) Ions

The electron pair donor ability of the three nitrogen donor solvents used in this comparison differ significantly with *D*_S_ values of 12, 38 and 69 for acetonitrile, pyridine and liquid ammonia, respectively [11]. The solvated copper(I) ion binds four acetonitrile and pyridine molecules in a tetrahedral fashion in solution, with mean Cu-N bond distances of 1.99 and 2.06 Å, respectively [20]; the corresponding Cu-N bond distances in the solid state are 1.995 (Table S5, 54 structures) and 2.050 Å [21,22,23,24], respectively. The significantly shorter Cu-N bond to acetonitrile depends on the smaller nitrogen atomic radius in acetonitrile, triple bound to carbon, than in pyridine with two delocalized bonds to neighboring carbon atoms. A limited number of two-coordinated linear complexes of acetonitrile and pyridine and a three-coordinate acetonitrile complex are reported the solid state (Tables S5 and S6). Copper(I) forms two-coordinated complexes with both acetonitrile and pyridine in aqueous solution [25]. Copper(I) binds only three ammonia molecules in liquid ammonia, most likely in a triangular configuration with a Cu-N bond distance of 2.00 Å [26], even though there is sterically room for more. One three-coordinated ammine copper(I) complex has been reported in the solid state [27], but it has an unusual T-shaped coordination geometry.

#### 2.4.2. Ammine, Acetonitrile and Pyridine Solvated Silver(I) Ions

As for copper(I), the coordination chemistry of silver(I) in nitrogen donor solvents varies with solvents. The solvated silver(I) ion binds four acetonitrile and pyridine molecules in a tetrahedral fashion in solution with mean Ag-N bond distances of 2.25 and 2.30 Å, respectively [28,29]; the corresponding Ag-N bond distances in the solid state are 2.277 [30,31,32,33,34,35,36,37,38] and 2.318 Å [39,40,41,42,43,44], respectively. Silver(I) forms two-coordinated complexes with both acetonitrile and pyridine in aqueous solution [25]. Silver(I) binds three ammonia molecules in liquid ammonia at 2.26 Å but only two in concentrated aqueous ammonia at 2.15 Å [45]. In the solid state, it has been reported that silver(I) binds two, three and four ammonia molecules in linear, triangular and tetrahedral coordination geometry with mean Ag-N bond distances of 2.124 (54 structures), 2.280 Å (4 structures) and 2.348 Å (1 structure), respectively (Table S2).

#### 2.4.3. Ammine, Acetonitrile and Pyridine Solvated Gold(I) Ions

Acetonitrile, pyridine and ammine solvated gold(I) ions are all linear in the solid state with Au-N bond distances of 1.960 [46,47,48,49], 2.023 [50,51], and 2.038 Å [52,53,54], respectively, and in liquid and aqueous ammonia solutions with Au-N bond distances of 2.022 and 2.025 Å, respectively [45]. However, in acetonitrile and pyridine solution, the bond distances are 2.19 and 2.16 Å, respectively [55], indicating coordination numbers around four.

#### 2.4.4. Ammine and Acetonitrile Solvated Zinc(II) Ions

Both tetrahedrally and octahedrally coordinated acetonitrile and ammine solvated zinc(II) ions are reported in the solid state, with mean Zn-N bond distances of 1.99 [56,57], and 2.13 Å [58,59], and 2.02 and 2.20 Å, respectively (18 tetrahedral and 6 octahedral structures) (Table S2). No pyridine solvates of zinc(II) are reported either in the solid state or pyridine solution. The ammine solvated zinc(II) ion is five-coordinated in liquid ammonia with a mean Zn-N bond distance of 2.12 Å, while it is four-coordinated in aqueous ammonia with a mean Zn-N bond distance of 2.03 Å [60].

#### 2.4.5. Ammine Solvated Cadmium(II) Ions

No cadmium acetonitrile and pyridine solvates are reported in either the solid state or solution. Both tetra- and hexaamminecadmium(II) ions are reported in the solid state, with mean Cd-N bond distances of 2.29 [61], and 2.37 Å [62,63,64,65], respectively. The ammine solvated cadmium(II) ion is six-coordinated in an octahedral fashion in both liquid and aqueous ammonia, with mean Cd-N bond distances of 2.35 Å [60].

#### 2.4.6. Ammine and Pyridine Solvated Mercury(II) Ions

No mercury(II) acetonitrile solvate is reported in either the solid state or solution. A six-coordinated pyridine solvated mercury(II) ion is reported in both the solid state and solution. This complex displays a second-order Jahn–Teller distortion, with mean Hg-N bond distances of 2.44 and 2.48 Å in the square planar and equatorial positions, respectively [66]. The ammine solvated mercury(II) ion in the solid state has a linearly distorted tetrahedral coordination with a N-Hg-N bond angle of 122° in solid [Hg(NH_3_)_4_](ClO_4_)_2_ with Hg-N bond distances of 2.175, 2.255 and 2 × 2.277 Å [67]. A similar tetrahedral coordination is maintained in liquid and aqueous ammonia solution, with mean Hg-N bond distances of 2.225 and 2.226 Å, respectively [67]. The heating of solid [Hg(NH_3_)_4_](ClO_4_)_2_ to 400 °C results in the formation of bis-solvated [Hg(NH_3_)_2_](ClO_4_)_2_, with a Hg-N bond distance of 2.055 Å [67].

#### 2.4.7. Summary of Coordination Chemistry of Solvated Metal Ions with Nitrogen Donor Solvents

Acetonitrile and pyridine solvated copper(I), silver(I) and gold(I) ions are four-coordinate in a tetrahedral fashion in solution with M-N bond distances of 1.99, 2.25, 2.19 Å, and 2.06, 2.30, 2.16 Å in acetonitrile and pyridine, respectively [20,28,29,55]. Two-coordinated acetonitrile, pyridine and ammine solvated copper(I), silver(I) and gold(I) ions in a linear fashion are reported in the solid state, with mean M-N bond distances of 1.84, 2.12, 1.96 Å, 1.90, 2.05, 2.023 Å, and 1.89, 2.12, 2.04 Å, respectively, vide supra. Ammine solvated zinc(II), cadmium(II) and mercury(II) ions in a tetrahedral fashion in the solid state have M-N bond distances of 2.02, 2.29 and 2.31 Å, respectively, vide supra. This shows that the Au-N bond distances are significantly shorter than the corresponding Ag-N ones at all coordination numbers in all solvents. For the zinc group, the M-N bond distance increases down the group, even though the difference between cadmium(II) and mercury(II) is small. The ionic radii proposed by Shannon for six-coordinated complexes of copper(I), silver(I), gold(I), zinc(II), cadmium(II) and mercury(II) are 0.77, 1.15, 1.37, 0.74, 0.95 and 1.02 Å, respectively, increasing down the groups. The Hg-N bond, and especially the Au-N bond, distances are much shorter than expected from the ionic radii, which reflects a significant covalent contribution in these bonds. Further evidence of this fact is provided by the small difference between the M-N and M-O bond distances between d^10^ metal ions and soft donor solvent molecules (Figure 3, Figure 4 and Figure S3). Furthermore, low coordination numbers are favored in solvated soft metal ions with soft donor solvents seen in, e.g., liquid ammonia, where copper(I) and silver(I) ions are three-coordinated, the gold(I) ion two-coordinated and the zinc(II) and mercury(II) ions five- and four-coordinated, respectively, vide supra, even though there is sterically room for higher coordination numbers.

### 2.5. Structure Relationship Between Hydrated and N,N-Dimethylthioformamide Solvated Metal Ions

Only a limited number of structures of solvated metal ions with sulfur donor solvents have been reported, mainly due to low permittivity. *N,N*-dimethylthioformamide (DMTF) is one of few sulfur donors with sufficient permittivity to dissolve and dissociate electrolytes [68]. The reported structures of DMTF solvated metal ions in the solid state include [Fe(DMTF)_6_](ClO_4_)_2_, (*d*(Fe-S) = 2.541 Å) [69], [Ni(DMTF)_6_](ClO_4_)_2_, (*d*(Ni-S) = 2.459 Å) [70], [Cu(DMTF)_4_]ClO_4_, (*d*(Cu-S) = 2.337 Å) [71], [Au(DMTF)_4_]BF_4_, (*d*(Au-S) = 2.290 (EXAFS data) Å) [71], [Zn(DMTF)_4_](CF_3_SO_3_)_2_, (*d*(Zn-S) = 2.240 Å) [72], [Cd(DMTF)_6_](ClO_4_)_2_, (*d*(Cd-S) = 2.714 Å) [72], and [Hg(DMTF)_2_](ClO_4_)_2_, (*d*(Hg-S) = 2.351 Å) [72], and in DMTF solution, [Fe(DMTF)_4_]^3+^ (*d*(Fe-S) = 2.206 Å) [68], [Ni(DMTF)_6_]^2+^ (*d*(Ni-S) = 2.454 Å) [70], [Cu(DMTF)_4_]^+^ (*d*(Cu-S) = 2.36 Å) [71], [Ag(DMTF)_4_]^+^ (*d*(Ag-S) = 2.58 Å) [71], [Au(DMTF)_2_]^+^ (*d*(Au-S) = 2.283 Å) [71], [Zn(DMTF)_6_]^2+^ (*d*(Zn-S) = 2.362 Å) [73], [Cd(DMTF)_6_]^2+^ (*d*(Cd-S) = 2.69 Å) [73], and [Hg(DMTF)_2_]^2+^ (*d*(Hg-S) = 2.527 Å) [73], [Ga(DMTF)_4_]^2+^ (*d*(Ga-S) = 2.233 Å) [74], and [Bi(DMTF)_6_]^2+^ (*d*(Bi-S) = 2.794 Å) [75]. In order to estimate the difference in atomic radius between DMTF sulfur and water oxygen, the M-S and M-O bond distances for the iron(II), nickel(II) and cadmium(II) DMTF solvates and hydrates in Table 3 are used; this difference is ca. 0.41 Å. The iron(II), nickel(II) and cadmium(II) ions form six-coordinated octahedral DMTF solvate complexes in both the solid state and DMTF solution, while the zinc(II) and mercury(II) ions form four-coordinated tetrahedral DMTF solvate complexes in DMTF solution [73], even though their ionic radii are larger than that of, e.g., nickel(II) [3]. For mercury(II), the solvate precipitating from DMTF solution is a linear bis-solvate [72]. The reason for its lower coordination number is most likely due to higher covalency. Copper(I) forms a tetra-coordinated solvate in both the solid state and DMTF solution [71]. Silver(I) forms a dimeric three-coordinate DMTF solvate in the solid state and a four-coordinated solvate in a tetrahedral fashion in DMTF solution [71]. The gold(I) ion forms a linear bis solvate in both the solid state and DMTF solution [71]. The lower coordination number of gold(I) is due to electronic factors, with a high degree of the covalency of the Au-S bonds promoting a linear configuration. The gallium(III) and bismuth(III) ions bind four and six DMTF molecules in their solvates, respectively [74,75]. The low coordination numbers of the gallium(III), iron(III) and bismuth(III) ions are certainly due to influence by steric factors as their ionic radii are too small to accommodate more than four, four and six sulfur atoms, respectively.

### 2.6. Summary of Solvate Structures of Monovalent d^10^ Metal Ions with Phosphorus Donor Solvents

The coordination chemistry of the trialkyl and triphenyl phosphite and phosphine solvated monovalent d^10^ metal ions does not follow any obvious pattern. The trialkyl phosphines form two- and four-coordinated complexes with copper(I) and silver(I) in the solid state, while for gold(I), only two-coordinate linear complexes are reported (Table S4). The complexes with triphenyl phosphine follow a different pattern. For copper(I), only three-coordinated triangular complexes, for silver(I) three- and four-coordinated complexes, and for gold(I) two-, three- and four-coordinated complexes are reported, as seen in Table S4. Only one homoleptic phosphite complex has been reported in the solid state, a four-coordinate tetrahedral copper(I) complex (Table S4). The same Cu-P bond distances have been observed in a series of trialkyl phosphite and triphenyl phosphite solvated copper(I) ions in solution as determined by EXAFS, showing that they all are four-coordinated. Shorter Ag-P and Au-P bond distances of triethyl and triphenyl phosphite and tri-n-butyl phosphine solvated silver(I) and gold(I) ions in the solid state indicate a coordination number of three in solution (Tables S4 and S7).

### 2.7. Structural Effects of Soft Solvents on the Coordination Chemistry of Soft Metal Ions

All metal ions except the mono- and divalent d^10^ metal ions have the same coordination number and geometry independent of the binding character of the solvent donor atom as long as no steric restrictions apply. The iron(III) and gallium(III) ions have too small ionic radii to accommodate six sulfur atoms, resulting in tetrahedral coordination geometry in DMTF [68,74], and the bismuth(III) ion binds only six DMTF molecules, while it is eight-coordinated in aqueous solution [75].

**Figure 5:**
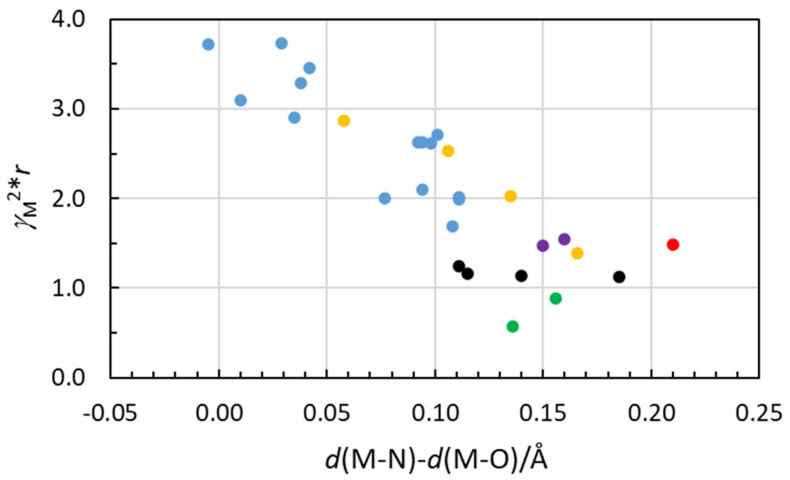
Scatter plot showing the correlation between the difference in M-N and M-O bond distances in ammine solvated and hydrated metal ions and the covalent bonding-index, *γ*_M_^2^*r. The color coding is the same as in Figure 3, Li^+^ and Na^+^ ions (green dots), Mg^2+^, Ca^2+^, Sr^2+^ and Ba^2+^ ions (black dots), La^3+^, Sm^3+^ and Yb^3+^ ions (purple dots), zirconium (IV) ion (red dot), transition metal ions (blue dots) and Al^3+^, Ga^3+^, In^3+^ and Tl^3+^ group 3A metal ions (yellow dots).

The solvated aluminum(III) ion and the trivalent d^10^ metal ions all have octahedral coordination geometry in both the hydrates and the ammine solvates, but the difference in the M-O and M-N bond distances changes a lot down the group (Table 3 and Figure 5). The bonding character of group 3A ions include the aluminum(III) ion, a typically hard electron pair acceptor, forming mainly electrostatic interactions, while the thallium(III) ion is regarded as a soft electron pair acceptor, forming bonds with a large degree of covalency. The relatively high charge density on trivalent metal ions results in an electrostatic bonding contribution that requires as many ligands as the space allows to shield the charge on the metal ion as efficiently as possible. By using the Al-N bond distance in the hexa-amminealuminum(III) ion, 2.048 Å [76,77,78,79], and the ionic radius of aluminum, 0.54 Å [3], the atomic radius of ammine nitrogen becomes 1.51 Å in a M-N bond with a high degree of electrostatic contribution; this atomic radius of ammonia nitrogen is slightly larger than that proposed by Shannon, 1.46 Å [3]. By using an atomic radius of nitrogen in ammonia of 1.51 Å and the proposed ionic radii of the gallium(III), indium(III) and thallium(III) ions, 0.62, 0.80 and 0.885 Å [3], the expected Ga-N, In-N and Tl-N bond distances become 2.13, 2.31 and 2.395 Å, respectively. However, the experimentally observed Ga-N, In-N and Tl-N bond distances are 2.08, 2.23 and 2.288 Å, thus, 0.05, 0.08 and 0.11 Å shorter than those predicted from ionic radii and the atomic radius of nitrogen in ammonia, respectively. This shows that the electronic overlap of the orbitals significantly shortens the M-N bond in ammine solvates with the increasing ability of the metal ion to participate in covalent interactions.

The metal ions regarded as being most soft display the smallest difference between the M-O and M-N bond distances, including silver(I), gold(I), mercury(II), palladium(II), platinum(II), rhodium(III), iridium(III) and thallium(III), as seen in Table 3 and Figure S3. The high charge density of the trivalent metal ions rhodium(III), iridium(III) and thallium(III) means that the hydrates and ammine solvates are six-coordinate octahedral. Palladium(II) and platinum(II) have the same square planar configuration in both the hydrates and ammine solvates. Among the divalent d^10^ metal ions, the hydrated and ammine solvated cadmium(II) ions are six-coordinated in an octahedral fashion, and while the ammine solvated zinc(II) ion can be either four- and six-coordinated in the solid state, it is four-coordinated in a tetrahedral fashion in aqueous ammonia but five-coordinated in liquid ammonia [60] (Table 3 and Table S2). The softest of the divalent d^10^ metal ions, mercury(II), is four-coordinate tetrahedral, maybe somewhat distorted, in liquid ammonia, while it is four- or two-coordinated in the solid state; thus, the ammine solvated mercury(II) ion has a lower coordination number than sterically possible. The trialkyl phosphine and phosphite solvated copper(I) ion is four-coordinate tetrahedral in solution, while the silver(I) and gold(I) ions seem to be three-coordinate in solution, thus possessing a coordination number lower than sterically possible.

The coordination number of the monovalent d^10^ metal ions copper(I), silver(I) and gold(I) solvates never exceeds four, despite the ionic radii proposed by Shannon^3^ and the observed M-N bond distances in comparison with other metal ions that are six-coordinated such as ammine solvated cobalt(III), rhodium(III) and chromium(III) ions (Table S2) and even though, sterically, there should be room for higher coordination numbers than observed.

### 2.8. Correlation Between Difference in Hydrate and Ammine Solvate Metal Ion Solvate Bond Distacnes and Covalent Bonding Index

To classify the ability of metal ions to form covalent bonds, a covalent bonding index, (*χ*_m_)^2^*r*, has been introduced, where *χ*_m_ is the electronegativity and *r* is the ionic radius of the metal ion [79,80]. Electronegativity is regarded as the energy of the empty valence orbital energy of a metal ion and a measure of its ability to accept electrons and thereby to form covalent bonds. The covalent bonding indices of the metal ions in Table S8 have been calculated from electronegativity values, *χ*_m_^2^, and the ionic radii calculated from the mean M-O bond distances in hydrated metal ions in the solid state [19]. The covalent bonding indices of the metal ions are plotted and compared to the observed difference in M-O/N bond distances in the hydrates and ammine solvates (Figure 5). There is a reasonably good correlation between these parameters. This shows that the difference in M-O/N bond distances in metal ion hydrates and ammine solvates can be used as a measure of the ability of a metal to form covalent interactions. The uncertainty in the *χ*_m_ values is most likely larger than in the reported mean bond distances as significantly different values are reported in the literature [79,81]. The preference of low coordination numbers of the metal ions with most donor solvents, vide supra, may indicate relative *χ*_m_ values higher than the calculated ones. The slightly shorter Ag-N bond distance compared to that of Ag-O in the linear silver(I) ammine solvate and hydrate, respectively, supports this indication.

## 3. Experiment

The Distillation of Liquid Ammonia: Liquid ammonia was prepared by the distillation of aqueous ammonia (25%, Merck, Darmstadt, Germany). The distillation procedure has been described elsewhere [26]. 

Chemicals: Indium perchlorate, hexahydrate, [In(H_2_O)_6_](ClO_4_)_3_, (G. F. Smith Chemicals, Columbus, OH, USA) was used as purchased. Hexakis(dimethylsulfoxide)thallium(III) perchlorate, [Tl(OS(CH_3_)_2_)_6_](ClO_4_)_3_, was prepared as described elsewhere [82].

Warning! Thallium is a toxic element and thallium samples must be handled with great care and personal protection for everyone in the same space

The Preparation of Liquid Ammonia Solutions: Indium perchlorate hexahydrate and hexakis(dimethylsulfoxide)thallium(III) perchlorate were dissolved in liquid ammonia at ca. 220 K. Ligand exchange reactions take place instantaneously and ice and solid dimethylsulfoxide freeze out, respectively. The prepared solutions were immediately transferred to an EXAFS cell or an NMR tube for measurements.

*EXAFS Data Collection:* Indium K and thallium L_III_-edge X-ray absorption spectra were recorded at the wiggler beam line 4–1 (old station) at the Stanford Synchrotron Radiation Lightsource (SSRL), Stanford, CA, USA. The workstation was equipped with a Si(220) double-crystal monochromator. SSRL operated at 3.0 GeV and a maximum current of 100 mA. Data collection was performed in transmission mode. Higher-order harmonics were reduced by detuning the second monochromator crystal to 80 and 50% of the maximum intensity at the end of the scans for indium and thallium, respectively. The liquid ammonia solution of indium(III) was kept in a 2 mm Vitone spacer between two titanium frames with 35–55 μm thick glass windows equipped with a copper rod dipped into a methanol/liquid nitrogen cooling mixture of ca. 175 K as described elsewhere [26]. The liquid ammonia solution of thallium(III) perchlorate was kept in a 1.5 mL air-tight NMR tube with a diameter of ca. 5 mm. The measurement of this solution was performed at ambient room temperature.

*EXAFS Data Analysis:* The energy scales of the X-ray absorption spectra were calibrated by assigning the first inflection point of the K edge of an indium foil to 27,918 eV and of the L_III_ edge of a thallium foil to 12,658 eV [83]. For each sample 6 scans were averaged, giving satisfactory data (*k*^3^-weighted) in the *k* ranges 2–13 and 2–7.5 Å^−1^ for indium and thallium, respectively. The EXAFSPAK program package (Stanford Synchrotron Light Source, Stanford, CA, USA)was used for data treatment and curve-fitting [84]. The EXAFS oscillations were obtained after performing standard procedures for pre-edge subtraction, and spline removal. The *k*^3^-weighted model functions were calculated using ab initio calculated phase and amplitude parameters obtained by the FEFF7 program package (version 7.02) [85].

*NMR Measurement:* The NMR measurement was performed on a Bruker DMX500 spectrometer (Billerica, MA, USA) at a probe temperature of 298 (±0.5) °C. The ^205^Tl NMR spectrum was recorded at 288.5 MHz in a 5 mm NMR tube with a PTFE valve, Wilmad^®^ (Vineland, NJ, USA). The [Tl(NH_3_)_6_]^3+^ complex in liquid ammonia was characterized by a sharp signal with a chemical shift of 2769 ppm (Figure S1) indicative of the oxidation state +III of thallium [13,17].

## 4. Conclusions

Ammonia and sulfur and phosphorus donor solvents are strong electron pair donors (Lewis bases) with good ability to form bonds with a significant degree of covalency to strong electron pair acceptors (Lewis acids). Water, on the other hand, is a weak electron pair donor that prefers to form electrostatic interactions due to the high electronegativity of the oxygen atom. This compilation of coordination numbers and coordination geometries of metal ion solvates of strong Lewis bases shows that the copper(I), silver(I), gold(I), zinc(II) and mercury(II) ion form solvate complexes in solution with lower coordination numbers than sterically possible, while the other metal ions have the highest sterically possible coordination numbers, as also found in their hydrates. The difference in the M-N and M-O bond distance of the ammine solvates and hydrates gives a measure of the covalent contribution to the M–solvate bond, which correlates well with the covalent bonding index, *γ*_M_^2^*r (Figure 5).

## Figures and Tables

**Table 1:** The results of In K and Tl L_3_-edge EXAFS data using *k*^3^-weighting. The parameters are frequency (*N*), mean bond distances (*d*/Å), Debye–Waller parameter (*σ*^2^/Å^2^), threshold energy (*E*_o_/eV) and amplitude reduction factor (*S*_o_^2^).

Sample/Species	Scattering Path	*N*	*d*	*σ* ^2^	*E*_o_	*S*_o_^2^
[In(H_2_O)_6_](ClO_4_)_3_ in NH_3_(l)						
[In(NH_3_)_6_]^3+^	In-N	6	2.232(3)	0.0054(3)	27,923.4(4)	0.95(3)
	MS(InN_6_)	3 × 6	4.45(6)	0.0145(12)		
[Tl(OS(CH_3_)_2_)_6_](ClO_4_)_3_ in NH_3_(l)						
[Tl(NH_3_)_6_]^3+^	Tl-N	6	2.288(8)	0.008414)	12,663.1(9)	1.19(11)
	MS(TlN_6_)	3 × 6	4.60(4)	0.009(5)		

**Table 2:** ^205^Tl NMR parameters at 288.5 MHz and 298 K for ammine solvated thallium(I) and thallium(III) ions in liquid and aqueous ammonia ^a^.

Sample/Species	*C*_Tl_ (mol∙dm^−3^)	*δ* (ppm)	Line Width (Hz)
[Tl(OS(CH_3_)_2_)_6_](ClO_4_)_3_ in NH_3_(l)			
[Tl(NH_3_)_6_]^3+^	0.046	2769	35
TlNO_3_ in NH_3_(l)			
[Tl(NH_3_)_n_]^+^	0.037	1768	70
TlNO_3_ in NH_3_(aq)			
[Tl(NH_3_)_n_]^+^	0.044	750	30

^a^ Dissolution of [Tl(OS(CH_3_)_2_)_6_](ClO_4_)_3_ in aqueous ammonia results in immediate hydrolysis of thallium(III) ion and insoluble thallium(III) hydroxide precipitates from solution.

**Table 3:** A summary of M-N bond distances in ammine solvated metal ions in the solid state and in liquid ammonia solution (italic), M-S bond distances in *N,N*-dimethylthioformamide (DMTF) solvated metal ions in the solid state and in DMTF solution (italic), M-O bond distances in hydrated metal ions in the solid state, the difference in M-N and M-O bond distances between ammine solvated and hydrated metal ions (*Diff (NH_3_-aq*), and the difference in M-S and M-O bond distances between DMTF solvated and hydrated metal ions. *CN* denotes the coordination number and *N* the number of reported structures. The citations for the reported structures in this table are given in Table S2 (ammine solvates) and Table S3 (DMTF solvates) and in Table S1 in [19] (hydrates).

Ion	*d*(M-N)/Å	*CN*	*N*	*d*(M-S)/Å	*CN*	*N*	*d*(M-O)/Å	*CN*	*N*	*Diff (NH_3_-aq)*	*Diff (DMTF-aq)*
Li^+^	2.078	4	23				1.942	4	58	0.136	
Na^+^	2.497	5	3				2.341	5	2	0.156	
K^+^	2.891	6	1								
Mg^2+^	2.177	6	5				2.066	6	541	0.111	
Ca^2+^	2.509	6	2				2.324	6	16	0.185	
	2.553	7	1				2.400	7	19	0.153	
	2.619	8	2				2.474	8	11	0.145	
Sr^2+^	2.753	8	4				2.613	8	20	0.140	
Ba^2+^	2.892	8	1				2.777	8	4	0.115	
	2.962	9	1				2.832	9	4	0.130	
Y^3+^	2.474	6	1								
	2.487	7	1								
La^3+^	2.678	6	1								
	2.706	9	1				2.556	9	10	0.150	
Ce^3+^	2.654	6	1								
Sm^3+^	2.632	9	1				2.472	9	4	0.160	
Yb^3+^	2.475.	6	1				2.326	8	29	0.149	
Zr^4+^	2.407	8	1				2.197	8	1	0.210	
V^2+^	2.225	6	1				2.131	6	4	0.094	
Cr^2+^	2.224	6	1				2.166	6	2	0.058	
Cr^3+^	2.073	6	20				1.965	6	24	0.108	
Mn^2+^	2.285	6	18				2.174	6	169	0.111	
Fe^2+^	2.218	6	10	2.541	6	1	2.120	6	95	0.098	0.421
Fe^3+^				2.206	4	1					
	2.173	6	1				1.995	6	21	0.178	
Ru^2+^	2.140	6	1				2.111	6	3	0.029	
Ru^3+^	2.099	6	11				2.021	6	2	0.078	
Os^3+^	1.964	6	1								
Co^2+^	2.179	6	12				2.087	6	471	0.092	
Co^3+^ (LS)	1.964	6	228				1.873	6	1	0.091	
Rh^3+^	2.064	6	6				2.022	6	4	0.042	
Ir^3+^	2.080	6	7				2.042	6	1	0.038	
Ni^2+^	2.132	6	21	2.459	6	1	2.055	6	399	0.077	0.404
Pd^2+^	2.039	4	33				2.029	6	3	0.010	
Pt^2+^	2.047	4	45				2.012	6	1	0.035	
Cu^+^	*2.004*	*3*	*1*								
				2.336	4	1					
Cu^2+^	2.008	4	8	2.36	4	1	1.927	4	4	0.081	0.43
	2.072	5	6				2.009	5	5	0.063	
	2.191	6	1				2.097	6	117	0.094	
Ag^+^	2.124	2	54				2.129	2	6	−0.005	
	*2.280*	*3*	*4*								
	2.348	4	1	2.58	4	1					
Au^+^	2.038	2	5	2.283	2	2					
Au^3+^	2.024	4	1				2.097	4	1	−0.073	
Zn^2+^	2.022	4	18	2.340	4	1	1.977	4	3	0.045	0.363
	*2.117*	*5*	*1*				*2.017*	*5*	*4*	*0.100*	
	2.199	6	6				2.088	6	274	0.111	
Cd^2+^	2.289	4	1								
	2.367	6	4	2.682	6	2	2.266	6	58	0.101	0.416
Hg^2+^	2.074	2	1	2.351	2	1					
	2.306	4	2								
				2.527	6	1					
Al^3+^	2.048	6	3				1.882	6	99	0.166	
Ga^3+^				2.333	4	1					
	2.081	6	1				1.946	6	13	0.135	
In^3+^	*2.232*	*6*	*1*				2.126	6	11	0.106	
Tl^3+^	*2.288*	*6*	*1*				2.230	6	1	0.058	
Bi^3+^				2.794	6	1					

## Data Availability

The original contributions presented in this study are included in the article/Supplementary Material. Further inquiries can be directed to the corresponding author.

## Supplementary Materials

The following supporting information can be downloaded at: https://www.mdpi.com/article/10.3390/molecules30153063/s1, Figure S1: ^205^Tl NMR spectrum of a 0.046 M solution of hexaamminethallium(III) perchlorate in liquid ammonia at room temperature; Figure S2: Difference in M-N and M-O bond distances in group 3A metal ion ammine solvates and hydrates. Figure S3: Difference in M-N and M-O bond distances in the softest metal ion ammine solvates and hydrates; Table S1: Summary of reported crystal structures of *N,N*-dimethylformamide solvated metal ions in the solid state; Table S2: Summary of structures of ammine solvated metal ions in the solid state, liquid ammonia and aqueous ammonia solution; Table S3: Structure of *N,N’*-dimethylthioformamide solvated metal ions in the solid state and *N,N’*-dimethylthioformamide solution; Table S4: Summary of structures of homoleptic copper(I), silver(I) and gold(I) complexes with phosphorus donor ligands in the solid state and solution; Table S5: Summary of structures of acetonitrile solvated metal ions in the solid state; Table S6: Summary of structures of pyridine solvated metal ions in the solid state; Table S7: Summary of M-P bond distances homoleptic copper(I), silver(I) and gold(I) complexes of trialkyl- and triphenyl phosphites and phosphines in the solid state and solution; Table S8: Electronegativity values, *χ*_m_, from ref. 1, ionic radius, *r*/Å, from ref. 2, and the calculated covalent index, (*χ*_m_)^2^*r*, of metal ions.
