# Supplementary material for: Coordination Chemistry of Solvated Metal Ions in Soft Donor Solvents
Source: Molecules. 2025 Jul 22;30(15):3063. doi: 10.3390/molecules30153063 (PMC12348266; doi:10.3390/molecules30153063)
Supplement: Supplementary file 1 [file molecules-30-03063-s001.zip › molecules-3704351-supplementary.pdf]

# Coordination Chemistry of Solvated Metal Ions in Soft Donor Solvents

**Kersti B. Nilsson, Mikhail Maliarik and Ingmar Persson**

**Supporting Material**

**Table S1.** Summary of reported crystal structures of *N,N*-dimethylformamide solvated metal ions in the solid state. Citations in purple text have not been included in the calculated mean bond distances.

**Lanthanum(III)**

*Eight-coordination, square antiprismatic configuration*

| CSD code | *d*(La-O) | Reference, compound formula |
|---|---|---|
| KIKVIK | 2.456 Å | Yarovoi, S. S. ; Mironov, Y. V. ; Solodovnikov, S. F. ; Solodovnikova, Z. A. ; Naumov, D. Y. ; Fedorov, V. E. *Koord. Khim.* **2006**, *32*, 743. $[La(OCHN(CH_3)_2)_8][Re_6Br_8S_6]$ |
| KIKWIL | 2.459 Å | Yarovoi, S. S. ; Mironov, Y. V. ; Solodovnikov, S. F. ; Solodovnikova, Z. A. ; Naumov, D. Y. ; Fedorov, V. E. *Koord. Khim.* **2006**, *32*, 743. $[La(OCHN(CH_3)_2)_8][Re_6Br_6Se_8]$ |
| HOXNIR | 2.462 Å | Chen Ling; Wu Xin-tao; Gao Xian-cheng; Zhang Wen-jian; Lin Ping, *J. Chem. Soc., Dalton Trans.* **1999**, 4303. $[La(OCHN(CH_3)_2)_8]_{2n}[Ag_{10}S_{32}W_8]_n$ |
| CUZYIH | 2.467 Å | Saha, S. ; Jana, P. P. ; Gomez-Garcia, C. J. ; Harms, K. ; Nayek, H. P. *Polyhedron* **2016**, *104*, 58. $[La(OCHN(CH_3)_2)_8][W_{12}PO_{40}]$ |
| DIQHUG | 2.469 Å | Ling, C. ; Xintao, W. ; Ping, L. *J. Chem. Cryst.* **1999**, *29*, 629. $[La(OCHN(CH_3)_2)_6(OCHN(C_2H_5)_2)_2][La(OCHN(CH_3)_2)_4(OCHN(C_2H_5)_2)_4][Ag_{10}W_8S_{32}]$ |
| AMOZOU | 2.476 Å | Gao, Y.; Chen, N.; Tian, Y.; Zhang, J.; Jia, D. *Inorg. Chem.* **2021**, *60*, 3761. $[La(OCHN(CH_3)_2)_8][Ag_9I_{12}]\cdot 2H_2O$ |
| NEQSUO | 2.480 Å | Skelton, B. W. ; Harrowfield, J. M. CCDC deposition number 1589427, **2017**. $[La(OCHN(CH_3)_2)_8](ClO_4)_3$ |
| KACDEB | 2.482 Å | Chen, N.; Gao, Y. ; Tian, Y. ; Wu, B. ; Jia, D. ; Zhao, S. *New. J. Chem.* **2020**, *44*, 19166. $[La(OCHN(CH_3)_2)_8][Pb_3I_9]$ |
| ODOLAY | 2.482 Å | Zhang, J.; Yang, X.; Ren, T.; Jia, D. *Dalton Trans.* **2023**, *52*, 6804. $[La(OCHN(CH_3)_2)_8][Bi_2I_9]$ |
| **Mean** | **2.470 Å/9 structures** | |

*Nine-coordination*

| ICSD/CSD code | *d*(La-O) | Reference, compound formula |
|---|---|---|
| YEKMUX | 2.528 Å | Berthet, J.-C.; Thuery, P.; Ephritikhine, M. *Polyhedron* **2006**, *25*, 1700. $[La(OCHN(CH_3)_2)_9]I_3$ |
| FEZCIX | 2.546 Å | Liu, S.; Plecnik, C. E. ; Meyers, E. A.; Shore, S. G. *Inorg. Chem.* **2005**, *44*, 282. $[La(OCHN(CH_3)_2)_9][Cu_{12}(CN)_{18}]\cdot 2(CH_3)_2NCHO$ |
| YELMUX01 | 2.548 Å | Hoch, C. *Z. Krist. Cryst. Mater.* **2020**, *235*, 401. $[La(OCHN(CH_3)_2)_9]I_3$ |
| BOWGIF | 2.557 Å | Danjo, H.; Nakagawa, T.; Katagiri, K.; Kawahata, M.; Yoshigai, S.; Miyazawa, T.; Yamaguchi, K. *Cryst. Growth Des.* **2015**, *15*, 384. $[La(OCHN(CH_3)_2)_9](C_{60}H_{30}B_3O_{12})\cdot(CH_3)_2NCHO$ |

| | | |
|---|---|---|
| **Mean** | **2.545 Å/4 structures** | |

**Cerium(III)**

*Eight-coordination, square antiprismatic configuration*

| ICSD/CSD code | *d*(Ce-O) | Reference, compound formula |
|---|---|---|
| XILJIN | 2.423 Å | Fang, W.; Tang, C.; Chen, R.; Jia, D.; Jiang, W.; Zhang, Y. *Dalton Trans.* **2013**, *42*, 15150. $[Ce(OCHN(CH_3)_2)_8][Ag_6I_9]$ |
| KIKVOQ | 2.455 Å | Yarovoi, S. S. ; Mironov, Y. V. ; Solodovnikov, S. F. ; Solodovnikova, Z. A. ; Naumov, D. Y. ; Fedorov, V. E. *Koord. Khim.* **2006**, *32*, 743. $[Ce(OCHN(CH_3)_2)_8][Re_6Br_8S_6]$ |
| KIKWOR | 2.457 Å | Yarovoi, S. S. ; Mironov, Y. V. ; Solodovnikov, S. F. ; Solodovnikova, Z. A. ; Naumov, D. Y. ; Fedorov, V. E. *Koord. Khim.* **2006**, *32*, 743. $[Ce(OCHN(CH_3)_2)_8][Re_6Br_6Se_8]$ |
| ATAPIW | 2.461 Å | Huang, W.; Wei, H. ; Li, L.; Qian, J. ; Zhang, C. *J. Cluster Sci.* **2016**, *27*, 1463. $[Ce(OCHN(CH_3)_2)_8][Cu_8I_{11}]\cdot C_2H_5OH$ |
| XILHUX | 2.470 Å | Fang, W.; Tang, C.; Chen, R.; Jia, D.; Jiang, W.; Zhang, Y. *Dalton Trans.* **2013**, *42*, 15150. $[Ce(OCHN(CH_3)_2)_8]_2[Ag_{10}Pb_3I_{22}]$ |
| OGICOW 150811 | 2.471 Å | Jobic, S.; Poirier-Coutansais, S.; Evain, M.; Brec, R., *Mater. Res. Bull.* **2001**, *36*, 2637. $[Ce(OCHN(CH_3)_2)_8][WS_4]_3\cdot H_2O$ |
| YEKMIL | 2.472 Å | Berthet, J.-C.; Thuery, P., Ephritikhine, M.; *Polyhedron*, **2006**, *25*, 1700, $[Ce(OCHN(CH_3)_2)_8]I_3$ |
| **Mean** | **2.464 Å/6 structures** | |

*Nine-coordination*

| ICSD/CSD code | *d*(Ce-O) | Reference, compound formula |
|---|---|---|
| YEKNAE | 2.507 Å | Berthet, J.-C.; Thuery, P.; Ephritikhine, M. *Polyhedron* **2006**, *25*, 1700. $[Ce(OCHN(CH_3)_2)_9]I_3$ |
| **Mean** | **2.507 Å/1 structure** | |

**Praseodymium(III)**

*Seven-coordination*

| ICSD/CSD code | *d*(Pr-O) | Reference, compound formula |
|---|---|---|
| AMOZUA | 2.426 Å | Gao, Y.; Chen, N.; Tian, Y.; Zhang, J.; Jia, D. *Inorg. Chem.* **2021**, *60*, 3761. $[Pr(OCHN(CH_3)_2)_7]_2[Ag_{16}I_{22}]$ |
| **Mean** | **2.426 Å/1 structure** | |

*Eight- and nine-coordination*

| ICSD/CSD code | d(Pr-O) | Reference, compound formula |
|---|---|---|
| XILJAF | 2.448 Å | Fang, W.; Tang, C.; Chen, R.; Jia, D.; Jiang, W.; Zhang, Y. *Dalton Trans.* **2013**, *42*, 15150. $[Pr(OCHN(CH_3)_2)_8]_2[Ag_{10}Pb_3I_{22}]$ |
| XILJOT | 2.449 Å (8)<br>2.500 Å (9) | Fang, W.; Tang, C.; Chen, R.; Jia, D.; Jiang, W.; Zhang, Y. *Dalton Trans.* **2013**, *42*, 15150. $[Pr(OCHN(CH_3)_2)_8]_2[Pr(OCHN(CH_3)_2)_9]_4[Pb_{22}I_{62}]$ |
| KIKWUX | 2.458 Å | Yarovoi, S. S. ; Mironov, Y. V. ; Solodovnikov, S. F. ; Solodovnikova, Z. A. ; Naumov, D. Y. ; Fedorov, V. E. *Koord. Khim.* **2006**, *32*, 743. $[Pr(OCHN(CH_3)_2)_8][Re_6Br_6Se_8]$ |
| XILJEJ | 2.505 Å | Fang, W.; Tang, C.; Chen, R.; Jia, D.; Jiang, W.; Zhang, Y. *Dalton Trans.* **2013**, *42*, 15150. $[Pr(OCHN(CH_3)_2)_8]_2[Ag_6I_9]$ |
| **Mean** | **2.452 Å/3 structures eight-coordination**<br>**2.500 Å/1 structure nine-coordination** | |

**Neodymium(III)**

*Eight-coordination, square antiprismatic configuration*

| ICSD/CSD code | d(Nd-O) | Reference, compound formula |
|---|---|---|
| KIKVUW | 2.408 Å | Yarovoi, S. S. ; Mironov, Y. V. ; Solodovnikov, S. F. ; Solodovnikova, Z. A. ; Naumov, D. Y. ; Fedorov, V. E. *Koord. Khim.* **2006**, *32*, 743. $[Nd(OCHN(CH_3)_2)_8][Re_6Br_8S_6]$ |
| HOXNOX | 2.417 Å | Cheng Ling; Wu Xin-tao; Gao Xian-cheng; Zhang Wen-jian; Lin Ping, *J. Chem. Soc., Dalton Trans.* **1999**, 4303. $[Nd(OCHN(C_2H_5)_2)_8]_{2n}[Ag_{10}S_{32}W_8]_n$ |
| YEKMOR01 | 2.430 Å | Hoch, C. *Z. Krist. Cryst. Mater.* **2020**, *235*, 401. $[Nd(OCHN(CH_3)_2)_9]I_3$ |
| TAQZIU | 2.433 Å | Huang, Q.; Wu, X.; Wang, Q.; Sheng, T.; Lu, J. *Angew. Chem., Int. Ed.* **1996**, *35*, 868. $[Nd(OCHN(CH_3)_2)_8[Ag_5W_4S_{16}]$ |
| TAQSIU01 | 2.433 Å | Huang, Q.; Wu, X.; Lu, J. *Polyhedron* **1997**, *16*, 833. $[Nd(OCHN(CH_3)_2)_8[Ag_5W_4S_{16}]$ |
| NEPSIN | 2.436 Å | Skelton, B. W.; Harrowfield, J. M. CCDC deposit number 1589285, **2017**. $[Nd(OCHN(CH_3)_2)_8](ClO_4)_3$ |
| YEKMOR | 2.437 Å | Berthet, J.-C.; Thuery, P.; Ephritikhine, M. *Polyhedron* **2006**, *25*, 1700. $[Nd(OCHN(CH_3)_2)_9]I_3$ |
| **Mean** | **2.428 Å/7 structures** | |

**Samarium(III)**

*Seven-coordination*

| ICSD/CSD code | d(Sm-O) | Reference, compound formula |
|---|---|---|

| | | |
|---|---|---|
| AMUBAO | 2.338 Å | Gao, Y.; Chen, N.; Tian, Y.; Zhang, J.; Jia, D. *Inorg. Chem.* **2021**, *60*, 3761. $[Sm(OCHN(CH_3)_2)_7]_2[Ag_{16}I_{22}]$ |
| **Mean** | **2.338 Å/1 structure** | |

*Eight-coordination, square antiprismatic configuration*

| ICSD/CSD code | *d*(Sm-O) | Reference, compound formula |
|---|---|---|
| KACDIF | 2.379 Å | Chen, N.; Gao, Y.; Tian, Y.; Wu, B.; Jia, D.; Zhao, S. *New J. Chem.* **2020**, *44*, 19166. $[Sm(OCHN(CH_3)_2)_8][Pb_5I_{13}]$ |
| JABTOZ | 2.398 Å | Hoch, C. *Z. Krist. Cryst. Mater.* **2020**, *235*, 401. $[Sm(OCHN(CH_3)_2)_9]I_3$ |
| JABTOZ01 | 2.413 Å | Bokouende, S. S; Kajjam, A. B.; Ward, C. L.; Allen, M. J. CCDC deposition number 2026698, **2020** $[Sm(OCHN(CH_3)_2)_9]I_3$ |
| **Mean** | **2.397 Å/3 structures** | |

**Europium(III)**

*Eight-coordination, square antiprismatic configuration*

| ICSD/CSD code | *d*(Eu-O) | Reference, compound formula |
|---|---|---|
| NIMFEX | 2.325 Å | Zhang, D.; Xue, Z.-Z.; Pan, J.; Shang, M.-M.; Mu, Y.; Han, S.-D.; Wang, G.-M. *Cryst. Growth Des.* **2018**, *18*, 7041. $[Eu(OCHN(CH_3)_2)_8][Ag_6I_9]$ |
| PAZGEG | 2.365 Å | Pells, J. A.; Guan, D.; Leznoff, D. B. *Eur. J. Inorg. Chem.* **2022**, e202200049. $(C_4H_9)_4N[Eu(OCHN(CH_3)_2)_8][Au(S_2CC(CN)_2)_2]_2$ |
| KIKWAD | 2.384 Å | Yarovoi, S. S. ; Mironov, Y. V. ; Solodovnikov, S. F. ; Solodovnikova, Z. A. ; Naumov, D. Y. ; Fedorov, V. E. *Koord. Khim.* **2006**, *32*, 743. $[Eu(OCHN(CH_3)_2)_8][Re_6Br_8S_6]$ |
| FEZCOD | 2.386 Å | Liu, S.; Plecnik, C. E.; Meyers, E.A.; Shore, S. G.; Inorg. Chem. **2005**, 44, 282. $[Eu(OCHN(CH_3)_2)_8](Cu_6(CN)_9]\cdot 0.64(CH_3)_2NCHO$ |
| BOWGOL | 2.390 Å | Danjo, H.; Nakagawa, T.; Katagiri, K.; Kawahata, M.; Yoshigai, S.; Miyazawa, T.; Yamaguchi, K. *Cryst. Growth Des.* **2015**, *15*, 384. $[Eu(OCHN(CH_3)_2)_8](B_3C_{60}H_{30}O_{12})\cdot 0.64(CH_3)_2NCHO$ |
| JABSUE | 2.391 Å | Hoch, C. *Z. Krist. Cryst. Mater.* **2020**, *235*, 401. $[Eu(OCHN(CH_3)_2)_9]I_3$ |
| VALZUG | 2.394 Å | Ghandour, Y.; Hammami, I.; Najmudin, S.; Bonifacio, C.; Belkhiria, M. S. *Acta Crystallogr., Sect. E* **2016**, *72*, 448. $[Eu(OCHN(CH_3)_2)_8][Mo_{12}PO_{40}]$ |
| ODONOO | 2.400 Å | Zhang, J.; Yang, X.; Ren, T.; Jia, D. *Dalton Trans.* **2023**, *52*, 6804. $[Eu(OCHN(CH_3)_2)_8][Bi_2I_9]$ |
| **Mean** | **2.387 Å/7 structures** | |

**Gadolinium(III)**

*Eight-coordination, square antiprismatic configuration*

| ICSD/CSD code | $d$(Gd-O) | Reference, compound formula |
|---|---|---|
| PAZGOQ | 2.354 Å | Pells, J. A.; Guan, D.; Leznoff, D. B. *Eur. J. Inorg. Chem.* **2022**, e202200049. $(C_4H_9)_4N[Gd(OCHN(CH_3)_2)_8][Au(S_2CC(CN)_2)_2]_2$ |
| OGOJOJ | 2.377 Å | Liu Shengming; Meyers, E. A.; Shore, S. G., *Angew. Chem., Int. Ed. Engl.* **2002**, *41*, 3609. $[Gd(OCHN(CH_3)_2)_8]_n[Cu_6(CN)_9]_n{\cdot}2(CH_3)NCHO$ |
| PAZGIK | 2.379 Å | Pells, J. A. Guan, D.; Leznoff, D. B. . *Eur. J. Inorg. Chem.* **2022**, e202200049. $(C_4H_9)_4N[Gd(OCHN(CH_3)_2)_8][Au(S_2CC(CN)_2)_2]_2{\cdot}2(CH_3)_2NHCO$ |
| JABTAL | 2.380 Å | Hoch, C. *Z. Krist. Cryst. Mater.* **2020**, *235*, 401. $[Gd(OCHN(CH_3)_2)_8]I_3$ |
| IXOXOI 110488 | 2.387 Å | Harrowfield, J.M.; Skelton, B.W.; White, A.H.; Wilner, F.R.; *Inorg.Chim.Acta.* **2004**, *357*, 2358. $(CH_3)_2NH_2\ [Gd(OCHN(CH_3)_2)_8](ClO_4)_4$ |
| **Mean** | **2.375 Å/5 structures** | |

**Terbium(III)**

*Eight-coordination, square antiprismatic configuration*

| ICSD/CSD code | $d$(Tb-O) | Reference, compound formula |
|---|---|---|
| PAZGAC | 2.342 Å | Pells, J. A.; Guan, D.; Leznoff, D. B. *Eur. J. Inorg. Chem.* **2022**, e202200049. $(C_4H_9)_4N[Tb(OCHN(CH_3)_2)_8][Au(S_2CC(CN)_2)_2]$ |
| NALDOW | 2.354 Å | Zhang, J.; Liu, Y.; Gong, L.; Xu, G.; Zhang, C. *Polyhedron* **2016**, *109*, 67. $[Tb(OCHN(CH_3)_2)_8][Ag_3W_3S_{12}]$ |
| RUNSOJ | 2.361 Å | Mishra, S.; Jeanneau, E.; Ledoux, G.; Daniele, S. *Dalton Trans.* **2009**, 4954. $[Tb(OCHN(CH_3)_2)_8][Ag_6I_9]$ |
| PARCOC | 2.362 Å | Mishra, S.; Jeanneau, E.; Iasco, O.; Ledoux, G.; Luneau, D.; Daniele, S. *Eur. J. Inorg. Chem.* **2012**, 2749. $[Tb(OCHN(CH_3)_2)_8][Bi_2I_9]$ |
| IXOYAV | 2.363 Å | Harrowfield, J.M.; Skelton, B.W.; White, A.H.; Wilner, F.R.; *Inorg.Chim.Acta.* **2004**, *357*, 2358. $[Tb(OCHN(CH_3)_2)_8](ClO_4)_3$ |
| PARTAF | 2.363 Å | Mishra, S.; Jeanneau, E.; Ledoux, G.; Daniele, S. *CrystEngComm* **2012**, *14*, 3894. $[Tb(OCHN(CH_3)_2)_8][CuI_2]I$ |
| PARCOC01 | 2.364 Å | Zhang, J.; Yang, X.; Ren, T.; Jia, D. *Dalton Trans.* **2023**, *52*, 6804. $[Tb(OCHN(CH_3)_2)_8][Bi_2I_9]$ |
| PARCES | 2.367 Å | Mishra, S.; Jeanneau, E.; Iasco, O.; Ledoux, G.; Luneau, D.; Daniele, S. *Eur. J. Inorg. Chem.* **2012**, 2749. $[Tb(OCHN(CH_3)_2)_8][Pb_3I_9]{\cdot}(CH_3)_2NCHO$ |
| BOWGUR | 2.368 Å | Danjo, H.; Nakagawa, T.; Katagiri, K.; Kawahata, M.; Yoshigai, S.; Miyazawa, T.; Yamaguchi, K. *Cryst. Growth Des.* **2015**, *15*, 384. $[Tb(OCHN(CH_3)_2)_8](B_3C_{60}H_{30}O_{12}){\cdot}3.42(CH_3)_2NCHO$ |
| RUNSEZ | 2.369 Å | Mishra, S.; Jeanneau, E.; Ledoux, G.; Daniele, S. *Dalton Trans.* **2009**, 4954. $[Tb(OCHN(CH_3)_2)_8]I_3$ |

| | | |
|---|---|---|
| NOCBUF | 2.379 Å | Pan, J.; Zhang, D.; Han, S.-D.; Hu, J.-X.; Xue, Z.-Z.; Wang, G.-M. *Cryst. Growth Des.* **2019**, *19*, 1825. $[Tb(OCHN(CH_3)_2)_8][Cu_8I_{11}]$ |
| **Mean** | **2.363 Å/11 structures** | |

**Dysprosium(III)**

*Eight-coordination, square antiprismatic configuration*

| ICSD/CSD code | *d*(Dy-O) | Reference, compound formula |
|---|---|---|
| XAXGOU | 2.347 Å | Wang, M.; Huang, S.-W.; Li, J.-B.; Gong, A.-W.; Wu, H.-Y.; Li, H.-H.; Chen, Z.-R. *J. Cluster Sci.* **2012**, *23*, 383. $[Dy(OCHN(CH_3)_2)_9][Bi_2I_9]$ |
| JABSIS | 2.355 Å | Hoch, C. *Z. Krist. Cryst. Mater.* **2020**, *235*, 401. $[Dy(OCHN(CH_3)_2)_9]I_3$ |
| CUZYON | 2.363 Å | Saha, S.; Jana, P. P.; Gomez-Garcia, C. J.; Harms, K.; Nayek, H. P. *Polyhedron* **2016**, *104*, 58. $[Dy(OCHN(CH_3)_2)_9][W_{12}PO_{40}]$ |
| **Mean** | **2.355 Å/3 structures** | |

**Erbium(III)**

*Eight-coordination, square antiprismatic configuration*

| ICSD/CSD code | *d*(Er-O) | Reference, compound formula |
|---|---|---|
| XAXGUA | 2.324 Å | Wang, M.; Huang, S.-W.; Li, J.-B.; Gong, A.-W.; Wu, H.-Y.; Li, H.-H.; Chen, Z.-R. *J. Cluster Sci.* **2012**, *23*, 383. $[Dy(OCHN(CH_3)_2)_9][Bi_2I_9]$ |
| JABSOY | 2.331 Å | Hoch, C. *Z. Krist. Cryst. Mater.* **2020**, *235*, 401. $[Er(OCHN(CH_3)_2)_9]I_3$ |
| LAZBIZ | 2.332 Å | Li, H.-H.; Huang, S.-W.; Lian, Z.-X.; Liu, J.-B.; Wan, M.; Chen, Z.-R. *Z. Anorg. Allg. Chem.* **2012**, *638*, 851. $[Er(OCHN(CH_3)_2)_8][(Ag_6I_9]$ |
| FEZCUJ | 2.334 Å | Liu Shengming; Plecnik, C.E.; Meyers, E. A.; Shore, S. G., *Inorg Chem.* **2005**, *44*, 282. $[Er(OCHN(CH_3)_2)_8]_n[Cu_6(CN)_9]_n \cdot 2(CH_3)_2NCHO$ |
| BOWHAY | 2.337 Å | Danjo, H.; Nakagawa, T.; Katagiri, K.; Kawahata, M.; Yoshigai, S.; Miyazawa, T.; Yamaguchi, K. *Cryst. Growth Des.* **2015**, *15*, 384. $[Er(OCHN(CH_3)_2)_8](B_3C_{60}H_{30}O_{12}) \cdot 3.47(CH_3)_2NCHO$ |
| **Mean** | **2.332 Å/4 stgructures** | |

**Ytterbium(II)**
*Six-coordination, octahedral configuration*

| ICSD/CSD code | *d*(Yb-O) | Reference, compound formula |
|---|---|---|
| WOLFIP | 2.351 Å | $[Yb(OCHN(CH_3)_2)_6](B(C_6H_5)_4)_2{\cdot}(CH_3)_2NCHO$ |
| **Mean** | **2.351 Å/1 structure** | |

**Ytterbium(III)**
*Eight-coordination, square antiprismatic configuration*

| ICSD/CSD code | *d*(Yb-O) | Reference, compound formula |
|---|---|---|
| OMAYAD | 2.292 Å | Zhang, J.; Meng,S.; Song, Y.; Yang, J.; Wei, H.; Huang, W.; Cifuentes, M. P.; Humphrey, M. G.; Zhang, C. *New. J. Chem.* **2011**, *35*, 328. $[Yb(OCHN(CH_3)_2)_8][Ag_3W_3S_{12}]$ |
| JABTUF | 2.308 Å | Hoch, C. *Z. Krist. Cryst. Mater.* **2020**, *235*, 401. $[Yb(OCHN(CH_3)_2)_9]I_3$ |
| BOWHEC | 2.317 Å | Danjo, H.; Nakagawa, T.; Katagiri, K.; Kawahata, M.; Yoshigai, S.; Miyazawa, T.; Yamaguchi, K. *Cryst. Growth Des.* **2015**, *15*, 384. $[Yb(OCHN(CH_3)_2)_8](B_3C_{60}H_{30}O_{12}){\cdot}3.47(CH_3)_2NCHO$ |
| CUZYUT | 2.359 Å | Saha, S.; Jana, P. P.; Gomez-Garcia, C. J.; Harms, K.; Nayek, H. P. *Polyhedron* **2016**, *104*, 58. $[Yb(OCHN(CH_3)_2)_9][W_{12}PO_{40}]$ |
| **Mean** | **2.306 Å/3 structures** | |

**Lutetium(III)**
*Eight-coordination, square antiprismatic configuration*

| ICSD/CSD code | *d*(U-O) | Reference, compound formula |
|---|---|---|
| KIKWEH | 2.293 Å | Yarovoi, S. S. ; Mironov, Y. V. ; Solodovnikov, S. F. ; Solodovnikova, Z. A. ; Naumov, D. Y. ; Fedorov, V. E. *Koord. Khim.* **2006**, *32*, 743. $[Lu(OCHN(CH_3)_2)_8][Re_6Br_8S_6]$ |
| KIKXAE | 2.307 Å | Yarovoi, S. S. ; Mironov, Y. V. ; Solodovnikov, S. F. ; Solodovnikova, Z. A. ; Naumov, D. Y. ; Fedorov, V. E. *Koord. Khim.* **2006**, *32*, 743. $[Lu(OCHN(CH_3)_2)_8][Re_6Br_6Se_8]$ |
| JABTEP | 2.315 Å | Hoch, C. *Z. Krist. Cryst. Mater.* **2020**, *235*, 401. $[Lu(OCHN(CH_3)_2)_9]I_3$ |
| **Mean** | **2.305 Å/3 structures** | |

**Table S2.** Summary of structures of ammine solvated metal ions in the solid state, liquid ammonia and aqueous ammonia solution. Citations in purple text have not been included in the calculated mean bond distances.

**Solid state**

**Lithium(I)**

*Four- coordination, tetrahedral configuration*

| ICSD/CSD | *d*(Li-N) | Reference and formula |
|---|---|---|
| NUCZIU | 2.057 Å | Flacke, F.; Jacobs, H. *Eur. J. Solid State Inorg. Chem.* **1997**, *34*, 495. $[Li(NH_3)_4][Sn(SnC_6H_5)_3]\cdot C_6H_6$ |
| 54002 | 2.058 Å | Korber, N.; Richter, F. *Chem. Commun.* **1998**, 2023. $[Li(NH_3)_4]_3P_{11}\cdot 5NH_3$ |
| XICMIH | 2.058 Å | Michel, R.; Nack, T.; Neufeld, R.; Dieterich, J. M.; Mata, R. A.; Stalke, D. Angew. Chem., Int. Ed. 2013, 52, 734. $[Li(NH_3)_4]C_9H_7$ |
| XAQMEI | 2.059 Å | Wiesler, K.; Korber, N. *Z. Krist. Cryst. Mater.* **2005**, *220*, 188. $[Li(NH_3)_4]_4[Sn_3(C_6H_5)_6]\cdot C_{12}H_{24}O_6\cdot 4NH_3$ |
| 279632 | 2.063 Å | Korber, N.; Fleischmann, A. *J. Chem. Soc., Dalton Trans.* **2001**, 383. $[Li(NH_3)_4]_4Sn_9\cdot NH_3$ |
| 416230 | 2.064 Å | Hanauer, T.; Aschenbrenner, J.C.; Korber, N. *Inorg. Chem.* **2006**, *45*, 6723. $[Li(NH_3)_4]_2P_{14}\cdot 2NH_3$ |
| 414142 VIHGOK | 2.065 Å | Kraus, F.; Panda, M.; Müller, T.; Albert, B. *Inorg. Chem.* **2013**, *52*, 4692. $[Li(NH_3)_4]_2B_{12}H_{12}\cdot 2NH_3$ |
| 415257 | 2.067 Å | Hanauer, T.; Kraus, F.; Reil, M.; Korber, N. *Monatsh. Chem.* **2006**, *137*, 147. $[Li(NH_3)_4]_2As_4$ |
| OKEMIB | 2.067 Å | Michel, R.; Herbst-Irmer, R.; Stalke, D. *Organometallics* **2010**, *29*, 6169. . $[Li(NH_3)_4]C_5H_5\cdot NH_3$ |
| 279633 | 2.068 Å | Korber, N.; Fleischmann, A. *J. Chem. Soc., Dalton Trans.* **2001**, 383. $[Li(NH_3)_4]_4Pb_9\cdot NH_3$ |
| 415189 | 2.069 Å | Hanauer, T.; Grothe, M.; Reil, M.; Korber, N. *Helv. Chim. Acta* **2005**, *88*, 950. $[Li(NH_3)_4]_3As_7\cdot NH_3$ |
| 409556 | 2.074 Å | Korber, N. D.; Richter, F. *Z. Kristallogr. – New Cryst. Struct.* **2001**, *216*, 117. $[Li(NH_3)_4]_2Te_2$ |
| 412156 | 2.077 Å | Pfisterer, K.; Korber, N. *Z. Anorg. Allg. Chem.* **2002**, *628*, 762. $NH_4[Li(NH_3)_4](P(NH_2)_2S_3)\cdot NH_3$ |
| 413744 | 2.078 Å | Hanauer, T.; Kraus, F.; Korber, N. *Monatsh. Chem.* **2005**, *136*, 119. $[Li(NH_3)_4]RbSe_3$ |
| RUJWIC | 2.081 Å | Scotti, N.; Zachwieja, U.; Jacobs, H. Z. Anorg. Allg. Chem. 1997, 623, 1503. $[Li(NH_3)_4]_2[(H_5C_6)_2SnSnC_6H_5)_2]$ |
| 406510 | 2.082 Å | Korber, N. D.; Richter, F. D. C. *Angew. Chem., Int. Ed.* **1997**, *36*, 1512. $[Li(NH_3)_4]_3[Li(NH_3)_2Sb_5]\cdot 2NH_3$ |
| 263143 | 2.084 Å | Guentner, C.; Korber, N. *Acta Crystallogr., Sect. E* **2012**, *68*, i84. $[Li(NH_3)_4][Li(NH_3)_3S_3]$ |
| 425119 | 2.084 Å | Hamberger, M.; Liebig, S.; Friedrich, U.; Korber, N.; Ruschewitz, U. *Angew. Chem., Int. Ed.* **2012**, *51*, 13006. $[Li(NH_3)_4]C_2H$ |
| CUGJAT | 2.084 Å | Crumpton, A. E.; Heilmann, A.; Aldridge, S. *Angew. Chem., Int. Ed.* **2024**, *63*, e2024063. $[Li(NH_3)_4](C_{27}H_{28}BF_5OP)$ |
| 417846 | 2.087 Å | Panda, M.; Hofmann, K.; Prosenc, M. H.; Albert, B. *Dalton Trans.* **2008**, 3956. $[Li(NH_3)_4]_2(B_6H_6)\cdot 2NH_3$ |

| | | |
|---|---|---|
| COVLUV | | |
| 3708 | 2.088 Å | Lorenz, C.; Gaertner, S.; Korber, N. *Crystals* **2018**, *8*, 1. $[Li(NH_3)_4]_4[Sn_4(NH_3)_4]$ |
| 409539 | 2.091 Å | Korber, N.; Grothe, M. *Z. Kristallogr. – New Cryst. Struct.* **2001**, *216*, 117. $[Li(NH_3)_4]_3(AsSe_4)$ |
| XUNYAH | 2.092 Å | Huang, Z.; Gallucci, J.; Chen, X.; Yisgedu, T.; Lingam, H. K.; Shore, S. G.; Zhao, J.-C. *J. Mater. Chem.* **2010**, *20*, 2743. $[Li(NH_3)_4][Li(NH_3)_3]B_{12}H_{12}$ |
| 55079 | 2.094 Å | Jacobs, H.; Barlage, H.; Friedriszik, M. *Z. Anorg. Allg. Chem.* **2004**, *630*, 645. $Li(NH_3)_4]I$ |
| 55078 | 2.096 Å | Jacobs, H.; Barlage, H.; Friedriszik, M. *Z. Anorg. Allg. Chem.* **2004**, *630*, 645. $Li(NH_3)_4]$B**r** |
| **Mean** | **2.076 Å/25 structures** | |

**Sodium(I)**

*Five-coordination*

| ICSD/CSD | *d*(Na-N) | Reference and formula |
|---|---|---|
| 172466 | 2.496 Å | Hanauer, T.; Kraus, F.; Reil, M.; Korber, N. *Monatsh. Chem.* **2006**, *137*, 147. $[Na(NH_3)_5]_2As_4{\cdot}3NH_3$ |
| 281007 | 2.496 Å | Korber, N.; Reil, M. *Chem. Commun.* **2002**, 84. $[Na(NH_3)_5]_2As_4{\cdot}3NH_3$ |
| 413743 | 2.500 Å | Hanauer, T.; Kraus, F.; Korber, N. *Monatsh. Chem.* **2005**, *136*, 136 $[Na(NH_3)_5]RbSe_3{\cdot}3NH_3$ |
| **Mean** | **2.497 Å/3 structures** | |

*Six-coordination, octahedral configuration*

| ICSD/CSD | *d*(Na-N) | Reference and formula |
|---|---|---|
| GUMLOT | 2.604 Å | Friedrich, U.; Schmidt, F.; Korber, N. *Z. Anorg. Allg. Chem.* **2024**, *650*, e202400086. $[Na(NH_3)_6][Na(NH_3)_2(O_6C_{20}H_{24})]_2[Bi_3Sn_5]{\cdot}10NH_3$ |

**Potassium(I)**

*Six-coordination, octahedral configuration*

| ICSD/CSD | *d*(K-N) | Reference and formula |
|---|---|---|
| QOTQOH | 2.758 Å | Yang, Y.; Liu, Y.; Wu, H.; Zhou, W.; Gao, M.; Pan, H. *Z. Phys. Chem. Chem. Phys.* **2014**, *16*, 135. $[K(NH_3)_6][K(NH_3)_2(C_{12}H_{24}O_6)][Ti(NH_3)_2(C_5H_5)_2]$ $[Ti(NH_3)(C_5H_5)_2Sn_9]{\cdot}14NH_3$ |

*Dimeric $[(NH_3)_5K(NH_3)_2K(NH_3)_5]$, seven-coordination*

| | | |
|---|---|---|
| XEJWAL | 2.969 Å | Wiesler, K.; Suchentrunk, C.; Korber, N. *Helv. Chim. Acta* **2006**, *89*, 1158. $[(NH_3)_5K(NH_3)_2K(NH_3)_5][Sn_6(C_6H_5)_{12}]{\cdot}4NH_3$ |

**Rubidium(I)**

*Six-coordination, dimeric*

| ICSD/CSD | *d*(Rb-N) | Reference and formula |
|---|---|---|
| VIHGUQ | 3.179 Å | Yang, Y.; Liu, Y.; Wu, H.; Zhou, W.; Gao, M.; Pan, H. *Z. Phys. Chem. Chem. Phys.* **2014**, *16*, 135. $[Rb_2(NH_3)_8]B_{12}H_{12}$ |

**Beryllium(II)**

*Four-coordination, tetrahedral configuration*

| ICSD/CSD | *d*(Rb-N) | Reference and formula |
|---|---|---|
| TOJFEH | 1.726 Å | Augustinov, W.; Müller, M.; Thomas-Hargreaves, L. R.; Ivlev, S. I.; Buchner, M. R. *Inorg. Chem.* **2024**, *63*, 5208. $[Be(NH_3)_4]NH_4(CF_3SO_3)_3{\cdot}NH_3$ |

**Magnesium(II)**

*Six-coordination, octahedral configuration*

| ICSD/CSD | *d*(Mg-N) | Reference and formula |
|---|---|---|
| 290894 | 2.166 Å | Jones, M. O.; Royse, D. M.; Edwards, P. P.; David, W. I. F. *Chem. Phys.* **2013**, *427*, 38. $[Mg(NH_3)_6]Cl_2$ |
| 290895 | 2.167 Å | Jones, M. O.; Royse, D. M.; Edwards, P. P.; David, W. I. F. *Chem. Phys.* **2013**, *427*, 38. $[Mg(NH_3)_6]Br_2$ |
| 90154 | 2.172 Å | Hwang, I.-C.; Drews, T.; Seppelt, K. *J. Am. Chem. Soc.* **2000**, *122*, 8486. $[Mg(NH_3)_6]Hg_{22}$ |
| 290896 | 2.181 Å | Jones, M. O.; Royse, D. M.; Edwards, P. P.; David, W. I. F. *Chem. Phys.* **2013**, *427*, 38. $[Mg(NH_3)_6]I_2$ |
| 90156 | 2.197 Å | Hwang, I.-C.; Drews, T.; Seppelt, K. *J. Am. Chem. Soc.* **2000**, *122*, 8486. $[Mg(NH_3)_6]Cl_2$ |
| **Mean** | **2.177 Å/5 structures** | |
| HIPFIX | 2.128 + 2.291 Å (2.237 Å) | Yang, Y.; Liu, Y.; Wu, H.; Zhou, W.; Gao, M.; Pan, H. *Phys. Chem. Chem. Phys.* **2014**, *16*, 135. $Li_2[Mg(NH_3)_6](BH_4)_4$ |

**Calcium(II)**

*Six-coordination, octahedral configuration*

| ICSD/CSD | *d*(Ca-N) | Reference and formula |
|---|---|---|
| 427991 | 2.501 Å | Woidy, P.; Karttunen, A. J.; Müller, T. G.; Kraus, F. *Z. Naturforach., Teil B* **2014**, *69*, 1141. $[Ca(NH_3)_6]Cl_2$ |
| QAKGAL | 2.509 Å | Cremer, U.; Disch, S.; Ruschewitz, U. *Z. Anorg. Allg. Chem.* **2004**, *630*, 2304. $[Ca(NH_3)_6][Cu(CN)_3]$ |
| **Mean** | **2.505 Å/2 structures** | |

*Seven-coordination*

| ICSD/CSD | *d*(Ca-N) | Reference and formula |
|---|---|---|
| 87604 | 2.553 Å | Schimek, G. L.; Drake, G. W.; Kolis, J. W. *Acta Chem. Scand.* **1999**, *53*, 145. $[Ca(NH_3)_7]S_6$ |

*Eight-coordination, square antiprismatic configuration*

| | | |
|---|---|---|
| 427511 | 2.610 Å | Woidy, P.; Karttunen, A. J.; Müller, T. G.; Kraus, F. *Z. Naturforach., Teil B* **2014**, *69*, 1141. $[Ca(NH_3)_8]Br_2$ |
| 427990 | 2.627 Å | Woidy, P.; Karttunen, A. J.; Müller, T. G.; Kraus, F. *Z. Naturforach., Teil B* **2014**, *69*, 1141. $[Ca(NH_3)_8]I_2$ |
| **Mean** | **2.619 Å/2 structures** | |

**Strontium(II)**

*Eight-coordination, square antiprismatic configuration*

| ICSD/CSD | *d*(Sr-N) | Reference and formula |
|---|---|---|
| 165624 | 2.740 Å | Korber, N.; Daniels, J. *Inorg. Chem.* **1997**, *36*, 4906. $[Sr(NH_3)_8]HP_{11}\cdot NH_3$ |
| IFUZEO | 2.748 Å | Wendig, U.; Brumm, H.; Jansen, M. *Chem.-Eur. J.* **2002**, *8*, 2769. $[Sr(NH_3)_8]C_{70}\cdot 3NH_3$ |
| OBEFUX | 2.750 Å | Panthofer, M.; Wedig, U.; Brumm, H.; Jansen, M. *Solid State Sci.* **2004**, *6*, 619. $[Sr(NH_3)_8]_3C_{140}\cdot 19NH_3$ |
| 194516 | 2.772 Å | Johnsen, R. E.; Jensen, P. B.; Norby, P.; Vegge, T. *J. Phys Chem.* 2014, *118*, 24349. $[Sr(NH_3)_8]Cl_2$ |
| **Mean** | **2.753 Å/4 structures** | |

**Barium(II)**

*Seven-coordination; monocapped octahedral configuration*

| ICSD/CSD | *d*(Ba-N) | Reference and formula |
|---|---|---|
| NUSVAY | 2.891 Å | Himmel, K.; Jansen, M. *Inorg. Chem.* **1998**, *37*, 3437. $[Ba(NH_3)_7]C_{60}\cdot NH_3$ |
| NUSVA01 | 2.892 Å | Daniels, J.; Weigend, F.; Jansen, M. *Z. Anorg. Allg. Chem.* **2021**, *647*, 1132. $[Ba(NH_3)_7]C_{60}\cdot NH_3$ |
| **Mean** | **2.892 Å/2 structures** | |

*Nine-coordination*

| ICSD/CSD | *d*(Ba-N) | Reference and formula |
|---|---|---|
| EBOYOJ | 2.962 Å | Brumm, H.; Peters, E.; Jansen, M. *Z. Angew. Chem., Int. Ed.* **2001**, *40*, 2069. $[Ba(NH_3)_9]C_{70}\cdot 7NH_3$ |

**Yttrium(III)**

*Six-coordination, octahedral configuration*

| ICSD/CSD | *d*(Y-N) | Reference and formula |
|---|---|---|
| 251869<br>RURRUT | 2.474 Å | Jepsen, L. H.; Ley, M. B.; Cerny, R.; Lee, Y.-S.; Cho, Y. W.; Ravnsbk, D.; Besenbacher, F.; Skibsted, J.; Jensen, T. R. *Inorg. Chem.* **2015**, *54*, 7402. $[Y(NH_3)_6](BH_4)_3$ |

*Seven-coordination, monocapped octahedral configuration*

| | | |
|---|---|---|
| RURSAA | 2.487 Å | Jepsen, L. H.; Ley, M. B.; Cerny, R.; Lee, Y.-S.; Cho, Y. W.; Ravnsbk, D.; Besenbacher, F.; Skibsted, J.; Jensen, T. R. *Inorg. Chem.* **2015**, *54*, 7402. $[Y(NH_3)_7](BH_4)_3$ |

**Lanthanum(III)**

*Six-coordination, octahedral configuration*

| ICSD/CSD | *d*(La-N) | Reference and formula |
|---|---|---|
| 13723 | 2.678 Å | Grinderslev, J. B.; Ley, M. B.; Lee, Y.-S.; Jepsen, L. H.; Jørgensen, M.; Cho, Y. W.; Skibsted, J.; Jensen, T. R. *Inorg. Chem.* **2020**, *59*, 7768. $[La(NH_3)_6](BH_4)_3$ |

*Nine-coordination*

| ICSD/CSD | *d*(La-N) | Reference and formula |
|---|---|---|
| 84283 | 2.706 Å | Schimek, G.L.; Young, D.M.; Kolis, J.W. *Eur. J. Inorg. Solid State Chem.* **1997**, *34*, 1037. $NH_4[La(NH_3)_9]S_5S$ |

**Cerium(III)**

*Six-coordination, octahedral configuration*

| ICSD/CSD | *d*(Ce-N) | Reference and formula |
|---|---|---|
| 13723 | 2.654 Å | Grinderslev, J. B.; Ley, M. B.; Lee, Y.-S.; Jepsen, L. H.; Jørgensen, M.; Cho, Y. W.; Skibsted, J.; Jensen, T. R. *Inorg. Chem.* **2020**, *59*, 7768. $[Ce(NH_3)_6](BH_4)_3$ |

**Samarium(III)**

*Tricapped trigonal prismatic configuration*

| ICSD/CSD | *d*(Sm-N) | Reference and formula |
|---|---|---|
| FITSEH | 2.605+2.687 Å | Quitmann, C. C.; Müller-Buschbaum, K. *Z. Anorg. Allg. Chem.* **2005**, *631*, 564. $[Sm(NH_3)_9][Sm(NC_4H_4)_6]$ |

**Europium(0)**

*Six-coordination, octahedral configuration*

| ICSD/CSD | *d*(Eu-N) | Reference and formula |
|---|---|---|
| 49648 | 2.865 Å | Oesterreicher, H.; Mammano, N.; Sienko, M. J *J. Solid State Chem.* **1969**, *1*, 10. [$Eu(NH_3)_6$] |

**Europium(II)**

*Eight-coordination, square antiprismatic configuration*

| ICSD/CSD | *d*(Eu-N) | Reference and formula |
|---|---|---|
| 142663 | 2.752 Å | Rudel, S. S.; Graubner, T.; Karttunen, A. J.; Kraus, F. *Z. Anorg. Allg. Chem.* **2020**, *646*, 1396. [$Eu(NH_3)_8$]$I_2$ |

**Ytterbium(II)**

*Eight-coordination, square antiprismatic configuration*

| ICSD/CSD | *d*(Yb-N) | Reference and formula |
|---|---|---|
| 142664 | 2.648 Å | Rudel, S. S.; Graubner, T.; Karttunen, A. J.; Kraus, F. *Z. Anorg. Allg. Chem.* **2020**, *646*, 1396. [$Yb(NH_3)_8$]$I_2$ |

**Ytterbium(III)**

*Eight-coordination, square antiprismatic configuration*

| ICSD/CSD | *d*(Yb-N) | Reference and formula |
|---|---|---|
| KIFPIZ | 2.475 Å | Müller-Buschbaum, K. *Z. Anorg. Allg. Chem.* **2007**, *633*, 1403. [$Yb(NH_3)_8$]$Yb(NC_4H_4)_6$] |

**Zirconium(IV)**

*Eight-coordination, square antiprismatic configuration*

| ICSD/CSD | *d*(Zr-N) | Reference and formula |
|---|---|---|
| TOXPON | 2.407 Å | Huang, J.; Tan, Y.; Su, J.; Gu, Q.; Cerny, R.; Ouyang, L.; Sun, D.; Yu, X.; Zhu, M. *Chem. Commun.* **2015**, *51*, 2794. [$Zr(NH_3)_8$]$(BH_4)_4$ |

**Vanadium(II)**

*Six-coordination, octahedral configuration*

| ICSD/CSD | *d*(V-N) | Reference and formula |
|---|---|---|
| 78859 | 2.225 Å | Essmann, R.; Kreiner, G.; Niemann, A.; Rechenbach, D.; Schmieding, A.; Sichla, T.; Zachwieja, U.; Jacobs, H. *Z. Anorg. Allg. Chem.* **1996**, *622*, 1161. [$V(NH_3)_6$]$I_2$ |

**Chromium(II)**

*Six-coordination, octahedral configuration*

| ICSD/CSD | *d*(Cr-N) | Reference and formula |
|---|---|---|
| 78860 | 2.224 Å | Essmann, R.; Kreiner, G.; Niemann, A.; Rechenbach, D.; Schmieding, A.; Sichla, T.; Zachwieja, U.; Jacobs, H. *Z. Anorg. Allg. Chem.* **1996**, *622*, 1161. $[Cr(NH_3)_6]I_2$ |

**Chromium(III)**

*Six-coordination, octahedral configuration*

| ICSD/CSD | *d*(Cr-N) | Reference and formula |
|---|---|---|
| 22076 | 2.028 Å | Mori, M.; Saito, Y.; Watanabe, T. *Bull. Chem. Soc. Jpn.* **1961**, *34*, 295. $[Cr(NH_3)_6][CuCl_5]$ |
| 26063 | 2.059 Å | Goldfield, S. A.; Raymond, K. N. *Inorg. Chem.* **1971**, *10*, 2604. $[Cr(NH_3)_6][CuBr_5]$ |
| 10255 | 2.064 Å | Raymond, K. N.; Meek, D. W.; Ibers, J. A. *Inorg. Chem.* **1968**, *7*, 1111. $[Cr(NH_3)_6][CuCl_5]$ |
| 2728 | 2.067 Å | Wieghardt, K.; Weiss, J. *Acta Crystallogr., Sect. B* **1972**, *28*, 529. $[Cr(NH_3)_6][MnF_6]$ |
| 87620 | 2.067 Å | Reynolds, P. A.; Figgis, B. N.; Sobolev, A. N. *Aust. J. Chem.* **1999**, *52*, 219. $K[Cr(NH_3)_6](ClO_4)_2Cl_2$ |
| 280496 | 2.067 Å | Figgis, B. N.; Sobolev, A. N.; Reynolds, P. A. *Acta Crystallo., Sect B* **1998**, *54*, 613. $Cs[Cr(NH_3)_6](ClO_4)_2Cl_2$ |
| 201417 | 2.069 Å | Clegg, W. *J. Chem. Soc., Dalton Trans.* **1982**, 593. $[Cr(NH_3)_6][HgCl_5]$ |
| 631 | 2.071 Å | Clegg, W. *Acta Crystallogr., Sect. B* **1976**, *32*, 2907. $[Cr(NH_3)_6][ZnCl_4]Cl$ |
| 69045 | 2.071 Å | Moron, M. C.; Le Bail, A.; Pons, J. *J. Solid State Chem.* **1990**, *88*, 498. $[Cr(NH_3)_6][Ni(H_2O)_6]Cl_5\cdot0.5NH_4Cl$ |
| 2729 | 2.073 Å | Wieghardt, K.; Weiss, J. *Acta Crystallogr., Sect. B* **1972**, *28*, 529. $[Cr(NH_3)_6][FeF_6]$ |
| 80102 | 2.074 Å | Ohba, S.; Fujita, T.; Bernal, I. *Acta Crystallogr., Sect. C* **1995**, *51*, 1481. $[Cr(NH_3)_6][CuCl_5]$ |
| 161157 | 2.074 Å | Mikuli, E.; Gorska, N.; Wrobel, S.; Sciesinski, J.; Sciesinska, E. *Z. Naturforsch., Teil A* **2007**, *62*, 179. $[Cr(NH_3)_6](ClO_4)_3$ |
| 281758 | 2.077 Å | Alig, E.; Fink, L.; Bolte, M. *Acta Crystallo., Sect E* **2003**, *59*, i154. $[Na(H_2O)_2][Cr(NH_3)_6]Cl_4$ |
| 57271 | 2.078 Å | Bäucker, C.; Niewa, R. *Z. Anorg. Allg. Chem.* **2022**, *648*, e202200209. $[Cr(NH_3)_6][AlF_6]$ |
| 411105 | 2.078 Å | Göbbels, D.; Meyer, G. *Z. Anorg. Allg. Chem.* **2000**, *626*, 1499. $[Cr(NH_3)_6][Cr(NH_3)_2F_4](BF_4)_2$ |
| 4067 | 2.079 Å | Clegg, W.; Greenhalgh, D. A.; Straughan, B. P. *J. Chem. Soc., Dalton Trans.* **1975**, 2591. $[Cr(NH_3)_6][HgCl_5]$ |
| 408816 | 2.081 Å | Tebbe, K. F.; Gilles, T. *Z. Naturforsch., Teil B* **1998**, *53*, 1127. $[Cr(NH_3)_6]I_3I_4$ |
| LEBRUF | 2.081 Å | Dieterich, S.; Strahle, J. *Z. Naturforsch., Teil B* **1998**, *53*, 1127. $[Cr(NH_3)_6]I_3I_4$ |
| 80103 | 2.082 Å | Ohba, S.; Fujita, T.; Bernal, I. *Acta Crystallo., Sect C* **1993**, *48*, 1574. $NH_4[Cr(NH_3)_6](C_{14}H_{14}N_5)_4\cdot4H_2O$ |
| 157360 | 2.119 Å | Gorska, N.; Hetmanczyk, L.; Lasocha, W.; Mikuli, E. *J. Alloys Compd.* **2007**, *442*, 80. $[Cr(NH_3)_6](BF_4)_3$ |
| **Mean** | **2.073 Å/20 structures** | |

**Manganese(II)**

*Six-coordination, octahedral configuration*

| ICSD/CSD | *d*(Mn-N) | Reference and formula |
|---|---|---|
| 186767 | 2.240 Å | Reardon, H.; Hanlon, J. M.; Grant, M.; Fullbrook, I.; Gregory, D. H. *Crystals* **2012**, *2*, 193. $[Mn(NH_3)_6]Cl_2$ |
| 12921 | 2.263 Å | Li, S.; Han, J.; Zhang, L.; Jiang, W.; Jia, D. *J. Solid State Chem.* **2019**, *269*, 341. $[Mn(NH_3)_6][(Mn_2(Sb_2S_4)_2]\cdot 2N_2H_4$ |
| 78861 | 2.270 Å | Essmann, R.; Kreiner, G.; Niemann, A.; Rechenbach, D.; Schmieding, A.; Sichla, T.; Zachwieja, U.; Jacobs, H. *Z. Anorg. Allg. Chem.* **1996**, *622*, 1161. $[Mn(NH_3)_6]Cl_2$ |
| 426563 | 2.272 Å | Kysliak, O.; Beck, J. *Inorg. Chem. Commun.* **2013**, *38*, 146. $[Mn(NH_3)_6]S_6$ |
| 426402 | 2.274 Å | Kysliak, O.; Beck, J. *Z. Anorg. Allg. Chem.* **2013**, *639*, 2860. $[Mn(NH_3)_6][SbSe_2]_2$ |
| 426562 | 2.274 Å | Kysliak, O.; Beck, J. *Inorg. Chem. Commun.* **2013**, *38*, 146. $[Mn(NH_3)_6][S_{2.71}Se_{2.29}]$ |
| 425025 | 2.278 Å | Kysliak, O.; Beck, J. *Eur. J. Inorg. Chem.* **2013**, 124. $[Mn(NH_3)_6][TeS_3]$ |
| 193739 | 2.279 Å | Xiong, W.-W.; A., Eashwer U.; Ng, Y. T.; Ding, J.; Wu, T.; Zhang, Q. *J. Am. Chem. Soc.* **2013**, *135*, 1256. $[Mn(NH_3)_6][(Mn_2(As_2S_4)_2]\cdot 2N_2H_4$ |
| 202342 | 2.279 Å | Jacobs, H.; Bock, J.; Stuve, C. *J. Less-Common Met.* **1987**, *134*, 207. $[Mn(NH_3)_6]I_2$ |
| 426565 | 2.281 Å | Kysliak, O.; Beck, J. *Inorg. Chem. Commun.* **2013**, *38*, 146. $[Mn(NH_3)_6]Se_6$ |
| 12919 | 2.285 Å | Li, S.; Han, J.; Zhang, L.; Jiang, W.; Jia, D. *J. Solid State Chem.* **2019**, *269*, 341. $[Mn(NH_3)_6][(Mn(NH_4)_2)(Sb_2S_4)_2]\cdot H_2O$ |
| 425410 | 2.287 Å | Kysliak, O.; Marcus, M.; Bredow, T.; Beck, J. *Inorg. Chem.* **2013**, *52*, 8327. $[Mn(NH_3)_6]Te_4$ |
| PUHJIL | 2.287 Å | Himmel, K.; Jansen, M. *Eur. J. Inorg. Chem.* **1998**, 1183. $[Mn(NH_3)_6]C_{60}\cdot 6NH_3$ |
| 426564 | 2.288 Å | Kysliak, O.; Beck, J. *Inorg. Chem. Commun.* **2013**, *38*, 146. $[Mn(NH_3)_6]Se_3$ |
| 430316 | 2.288 Å | Müller, T. G.; Buchner, M. R.; Scheubeck, T. J.; Korber, N.; Kraus, F. *Z. Anorg. Allg. Chem.* **2016**, *642*, 796. $[Mn(NH_3)_6](N_3)_2\cdot 4NH_3$ |
| 429495 | 2.291 Å | Jepsen, L. H.; Ley, M. B.; Filinchuk, Y.; Besenbacher, F.; Jensen, T. R. *ChemSusChem* **2015**, *8*, 1452. $[Mn(NH_3)_6](BH_4)_2$ |
| 12918 | 2.293 Å | Li, S.; Han, J.; Zhang, L.; Jiang, W.; Jia, D. *J. Solid State Chem.* **2019**, *269*, 341. $[Mn(NH_3)_6][(Mn(NH_3)_3)(Sb_2S_4)_2]Cl_2$ |
| 186768 | 2.316 Å | Reardon, H.; Hanlon, J. M.; Grant, M.; Fullbrook, I.; Gregory, D. H. *Crystals* **2012**, *2*, 193. $[Mn(NH_3)_6]Br_2$ |
| 248621 | 2.328 Å | Xiong, W.-W.; Miao, J.; Li, P.-Z.; Zhao, Y.; Liu, B.; Zhang, Q. *J. Solid State Chem.* **2014**, *218*, 146. $[Mn(NH_3)_6][Ag_4Mn_4Sn_3Se_{13}]$ |
| 429496 | 2.342 Å | Jepsen, L. H.; Ley, M. B.; Filinchuk, Y.; Besenbacher, F.; Jensen, T. R. *ChemSusChem* **2015**, *8*, 1452. $Li_2[Mn(NH_3)_6](BH_4)_4$ |

| | | |
|---|---|---|
| **Mean** | **2.285 Å/18 structures** | |

**Iron(II)**

*Six-coordination, octahedral configuration*

| ICSD/CSD | *d*(Fe-N) | Reference and formula |
|---|---|---|
| 50465 | 2.152 Å | Schoening, R. A.; Meyer, G. . *Z. Anorg. Allg. Chem.* **1998**, *624*, 1389. $[Fe(NH_3)_6][TaF_6]_2$ |
| 84264 | 2.207 Å | Schimek, G. L.; Kolis, J. W.; Long, G.-J. *Chem. Mater.* **1997**, *9*, 2776. $[Fe(NH_3)_6][Cu_8Sb_3S_{13}]$ |
| 78862 | 2.207 Å | Essmann, R.; Kreiner, G.; Niemann, A.; Rechenbach, D.; Schmieding, A.; Sichla, T.; Zachwieja, U.; Jacobs, H. *Z. Anorg. Allg. Chem.* **1996**, *622*, 1161. $[Fe(NH_3)_6]Cl_2$ |
| 426558 | 2.211 Å | Kysliak, O.; Beck, J. *Inorg. Chem. Commun.* **2013**, *38*, 146. $[Fe(NH_3)_6]S_6$ |
| 425409 | 2.215 Å | Kysliak, O.; Beck, J. *Z. Inorg. Chem.* **2013**, *52*, 8327. $[Mn(NH_3)_6]Te_4{\cdot}NH_3$ |
| 78863 | 2.216 Å | Essmann, R.; Kreiner, G.; Niemann, A.; Rechenbach, D.; Schmieding, A.; Sichla, T.; Zachwieja, U.; Jacobs, H. *Z. Anorg. Allg. Chem.* **1996**, *622*, 1161. $[Fe(NH_3)_6]Br_2$ |
| 426559 | 2.216 Å | Kysliak, O.; Beck, J. *Inorg. Chem. Commun.* **2013**, *38*, 146. $[Fe(NH_3)_6]Se_6$ |
| 84265 | 2.218 Å | Schimek, G. L.; Kolis, J. W.; Long, G.-J. *Chem. Mater.* **1997**, *9*, 2776. $[Fe(NH_3)_6][AgSbS_4]$ |
| 84266 | 2.227 Å | Schimek, G. L.; Kolis, J. W.; Long, G.-J. *Chem. Mater.* **1997**, *9*, 2776. $[Fe(NH_3)_6][AgAsS_4]$ |
| 202343 | 2.229 Å | Jacobs, H.; Bock, J.; Stuve, C. *J. Less-Common Met.* **1987**, *134*, 207. $[Fe(NH_3)_6]I_2$ |
| CACDIW | 2.235 Å | Roedern, E.; Jensen, T. R. *Inorg. Chem.* **2015**, *54*, 10477. $[Fe(NH_3)_6](BH_4)_2$ |
| 430567 CACDOC | 2.321 Å | Roedern, E.; Jensen, T. R. *Inorg. Chem.* **2015**, *54*, 10477. $[Fe(NH_3)_6](LiB_2H_8)_2$ |
| **Mean** | **2.218 Å/10 structures** | |

**Iron(III)**

*Six-coordination, octahedral configuration*

| | | |
|---|---|---|
| 412650 | 2.173 Å | Bremm, S.; Meyer, G. *Z. Anorg. Allg. Chem.* **2003**, *629*, 1875. $[Fe(NH_3)_6]_3[Fe_8Br_{14}]$ |

**Ruthenium(II)**

*Six-coordination, octahedral configuration*

| ICSD/CSD | *d*(Ru-N) | Reference and formula |
|---|---|---|
| 23456 | 2.137 Å | Trehoux, J.; Novogorocki, G.; Thomas, D. *Compt. Rend. Seanc l'Academ. Sciences, Serie C* **1972**, *274*, 1384. $[Ru(NH_3)_6]Cl_2$ |
| 26100 | 2.143 Å | Stynes, H. C.; Ibers, J. A. *Inorg. Chem.* **1971**, *10*, 2304. $[Ru(NH_3)_6]I_2$ |
| **Mean** | **2.140 Å/2 structures** | |

**Ruthenium(III)**

*Six-coordination, octahedral configuration*

| ICSD/CSD | *d*(Ru-N) | Reference and formula |
|---|---|---|
| 91763 | 2.075 Å | Subramanian, M. A.; Harlow, R. H.; Rao, V. N. M. *Mater. Res. Bull.* **2000**, *35*, 1587. $[Ru(NH_3)_6][AlCl_6]$ |
| 280494 | 2.086 Å | Figgis, B.; Sobolev, A.; Reynolds, P. *Acta Crystallogr., Sect. B* **1998**, *54*, 613. $Cs[Ru(NH_3)_6](ClO_4)_2Cl_2$ |
| 79696 | 2.091 Å | Engelhardt, L. M.; Reynolds, P. A.; Sobolev, A. N. *Acta Crystallogr., Sect. C* **1995**, *51*, 1045. $[Ru(NH_3)_6]Cl_3$ |
| 280491 | 2.094 Å | Figgis, B.; Sobolev, A.; Reynolds, P. *Acta Crystallogr., Sect. B* **1998**, *54*, 613. $Rb[Ru(NH_3)_6](ClO_4)_2Cl_2$ |
| 280489 | 2.097 Å | Figgis, B.; Sobolev, A.; Reynolds, P. *Acta Crystallogr., Sect. B* **1998**, *54*, 613. $K[Ru(NH_3)_6](ClO_4)_2Cl_2$ |
| 425255 | 2.098 Å | Khranenko; S. P.; Bykova; E. A.; Yusenko; K. V.; Tyutyunnik; A. P.; Gromilov, S. A. . *J. Struct. Chem.* **2013**, *54*, 931. $[Ru(NH_3)_6](ReO_4)_3{\cdot}2H_2O$ |
| 189227 | 2.101 Å | Dolega, D.; Mikuli, E.; Gorska, N.; Inaba, A.; Holderna-Natkaniec, K.; Nitek, W. *J. Solid State Chem.* **2013**, *204*, 233. $[Ru(NH_3)_6](ClO_4)_3$ |
| 26101 | 2.104 Å | Stynes, H. C.; Ibers, J. A. *Inorg. Chem.* **1971**, *10*, 2304. $[Ru(NH_3)_6](BF_4)_3$ |
| 73574 | 2.109 Å | Blake, A. B.; Delfs, C. D.; Engelhardt, L. M.; Figgis, B. N.; Reynolds, P. A.; White, A. H.; Moubaraki, B.; Murray, K. S. *J. Chem., Dalton Trans.* **1993**, 1417. $[Ru(NH_3)_6](SCN)_3$ |
| 67571 | 2.112 Å | Reynolds, P. A.; Delfs, C. D.; Figgis, B. N.; Engelhardt, L. M.; Moubaraki, B.; Murray, K. S. *J. Chem. Soc., Dalton Trans.* **1992**, 2029. $[Ru(NH_3)_6]Br(SO_4)$ |
| 425256 | 2.118 Å | Khranenko; S. P.; Bykova; E. A.; Yusenko; K. V.; Tyutyunnik; A. P.; Gromilov, S. A. *J. Struct. Chem.* **2013**, *54*, 931. $[Ru(NH_3)_6](MO_4)Cl{\cdot}3H_2O$ |
| **Mean** | **2.099 Å/11 structures** | |

**Osmium(III)**

*Six-coordination, octahedral configuration*

| ICSD/CSD | *d*(Ru-N) | Reference and formula |
|---|---|---|
| 87617 | 2.111 Å | Reynolds, P. A.; Figgis, B. N.; Sobolev, A. N. *Aust. J. Chem.* **1999**, *52*, 219. $K[Os(NH_3)_6](ClO_4)Cl_2$ |

**Cobalt(II)**

*Six-coordination, octahedral configuration*

| ICSD/CSD | *d*(Co-N) | Reference and formula |
|---|---|---|
| 38257 | 2.113 Å | Barnet, M. T.; Craven, B. M.; Freeman, H. C.; Kime, N. E.; Ibers, J. A. *Chem. Commun.* **1966**, 307. $[Co(NH_3)_6]Cl_2$ |
| 430565 CACDES | 2.136 Å | Roedern, E.; Jensen, T. R. *Inorg. Chem.* **2015**, *54*, 10477. $[Co(NH_3)_6](BH_4)_{1.04}Cl_{0.96}$ |

| | | |
|---|---|---|
| 74039 | 2.165 Å | Newman, J. M.; Binns, M.; Hambley, T. W.; Freeman, H. C. *Inorg. Chem.* **1991**, *30*, 3499. $[Co(NH_3)_6]Cl_2$ |
| 73346 | 2.170 Å | Newman, J. M.; Binns, M.; Hambley, T. W.; Freeman, H. C. *Inorg. Chem.* **1991**, *30*, 3499. $[Co(NH_3)_6]Cl_2$ |
| 279640 RAJNIZ | 2.172 Å | Brumm, H.; Jansen, M. *Z. Anorg. Allg. Chem.* **2001**, *627*, 1433. $[Co(NH_3)_6]C_{60}\cdot 6NH_3$ |
| 78864 | 2.176 Å | Essmann, R.; Kreiner, G.; Niemann, A.; Rechenbach, D.; Schmieding, A.; Sichla, T.; Zachwieja, U.; Jacobs, H. *Z. Anorg. Allg. Chem.* **1996**, *622*, 1161. $[Co(NH_3)_6]Br_2$ |
| 30703 | 2.183 Å | Kummer, S.; Babel, D. *Z. Naturforsch., Teil B* **1984**, *39*, 1118. $[Co(NH_3)_6](BF_4)_2$ |
| 430565 CACDAO | 2.183 Å | Roedern, E.; Jensen, T. R. *Inorg. Chem.* **2015**, *54*, 10477. $[Co(NH_3)_6](BH_4)_2$ |
| 30704 | 2.186 Å | Kummer, S.; Babel, D. *Z. Naturforsch., Teil B* **1984**, *39*, 1118. $[Co(NH_3)_6](PF_6)_2$ |
| 239009 QUTGUJ | 2.188 Å | Müller, T. G.; Kraus, F. *Acta Crystallogr., Sect. E* **2015**, *71*, 1418. $[Co(NH_3)_6][Co(CO)_4]_2$ |
| 412471 | 2.191 Å | Grothe, M.; Korber, N. *Z. Anorg. Allg. Chem.* **2003**, *629*, 399. $[Co(NH_3)_6)_2](As_{12}Se_4)\cdot 12NH_3$ |
| 203230 | 2.195 Å | Schiebel, P.; Prandl, W.; Papoular, R.; Paulus, W. *Acta Crystallogr., Sect. A* **1996**, *52*, 189. $[Co(NH_3)_6](PF_6)_2$ |
| 203231 | 2.199 Å | Schiebel, P.; Prandl, W.; Papoular, R.; Paulus, W. *Acta Crystallogr., Sect. A* **1996**, *52*, 189. $[Co(NH_3)_6](PF_6)_2$ |
| **Mean** | **2.179 Å/12 structures** | |

**Cobalt(III)**

*Six-coordination, octahedral configuration*

| ICSD/CSD | *d*(Co-N) | Reference and formula |
|---|---|---|
| 56126 | 1.855 Å | Hentschel, H.; Rinne, F. *Zentralblatt Mineral., Geolog. Palaeontolog, Abt. A* **1927**, 390-392. $[Co(NH_3)_6]I_3$ |
| WEHBUH 155531 | 1.932 Å | Charushnikova, I. A.; Krot, N. N.; Polyakova, I. N. *Radiokhim.* **2005**, *47*, 495. $[Co(NH_3)_6]_2[(NpO_2)_2C_8O_{16}]\cdot 3H_2O$ |
| CITQOO | 1.932 Å | Andreev, G.; Budantseva, N.; Fedoseev, A. *Inorg. Chem. Commun.* **2019**, *99*, 160. $(NH_4)_2[Co(NH_3)_6]_2[Am_3C_{24}H_{21}O_{31}]\cdot 35H_2O$ |
| VUCDUU | 1.933 Å | Charushnikova, L. A.; Krot, N. N.; Makarenkov, V. I.; Starikova, Z. A. *Radiochim.* **2014**, *56*, 364. $[Co(NH_3)_6]_2[Tm_3C_{24}H_{25}O_{36}]\cdot 6H_2O$ |
| 22133 | 1.936 Å | Kime, N. E.; Ibers, J. A. *Acta Crystallogr., Sect. B* **1969**, *25*, 168-169. $[Co(NH_3)_6]I_3$ |
| QAGKAM | 1.936 Å | Fedosseev, A. M.; Grigoriev, M. S.; Budantseva, N. A.; Guillaumont, D.; Le Naour, C.; Simoni, E.; Den Auwer, C.; Moisy, P. *Compt. Rend. Chim.* **2010**, *13*, 839. $K[Co(NH_3)_6]_2[Am_3C_{24}H_{22}O_{31}]\cdot 18H_2O$ |
| JACREN | 1.939 Å | Tian, R.; Wang, F.; Du, C.; Feng, L.; Liu, Y.; Zhang, C.; Pan, Q. *Chem. Res. Chin. Univ.* **2014**, *30*, 889. $[Co(NH_3)_6][C_6H_2O_{13}]\cdot H_2O$ |

| | | |
|---|---|---|
| 175069 | 1.943 Å | Bernal, I.; Lalancette, R. A.; Syzdek, D.; Grebowicz, J. *J. Therm. Anal. Calorim.* **2022**, *147*, 11119. $[Co(NH_3)_6]Cl_3$ |
| 280487 | 1.946 Å | Figgis, B.; Sobolev, A.; Reynolds, P. *Acta Crystallogr., Sect. B* **1998**, *54*, 613. $Cs[Co(NH_3)_6](ClO_4)_2Cl_2$ |
| FIRJIZ | 1.947 Å | Zhang, Y.; Collison, D.; Livens, F. R.; Helliwell, M.; Eccles, H.; Tinker, N. *J. Alloys Compd.* **1998**, *271*, 139. $[Co(NH_3)_6]_2[U_2C_{20}H_{24}O_{20}]Cl_2\cdot 7H_2O$ |
| 280334 | 1.949 Å | Clark, D. L.; Donohoe, R. J.; Gordon, J. C.; Gordon, P. L.; Webster Keogh, D.; Scott, B. L.; Drew Tait, C.; Watkin, J. G. *J. Chem. Soc., Dalton Trans.* **2000**, 1975. $[Co(NH_3)_6][Sm(CO_3)_3(H_2O)]\cdot 4H_2O$ |
| 189677 | 1.949 Å | Sharma, R. P.; Bala, R.; Sharma, R.; Venugopalan, P. *CrystEngComm* **2006**, *8*, 215. $[Co(NH_3)_6]Cl_2(SeCN)$ |
| QUTNIC | 1.949 Å | Jiang, J.; Acunzo, A.; Koch, S. A. *J. Am. Chem. Soc.* **2001**, *123*, 12109. $[Co(NH_3)_6][Fe(CN)_5CO]\cdot 3H_2O$ |
| AFIKUW | 1.950 Å | Wang, X.-Y.; Justice, R.; Sevov, S. C. *Inorg. Chem.* **2007**, *46*, 4626. $[Co(NH_3)_6]_2(O_3SC_6H_4C_6H_4SO_3)_3\cdot 2C_4H_8O\cdot 4H_2O$ |
| 35152 | 1.952 Å | Bernal, I.; Korp, J. D.; Schlemper, E. O.; Hussain, M. S. *Polyhedron* **1982**, *1*, 365. $[Co(NH_3)_6][CuCl_5]$ |
| 38258 | 1.953 Å | Barnet, M. T.; Craven, B. M.; Freeman, H. C.; Kime, N. E.; Ibers, J. A. *Chem. Commun.* **1966**, 307. $[Co(NH_3)_6]I_3$ |
| 201418 | 1.953 Å | Clegg, W. *J. Chem. Soc., Dalton Trans.* **1982**, 593. $[Co(NH_3)_6][HgCl_5]$ |
| 189678 | 1.953 Å | Sharma, R. P.; Bala, R.; Sharma, R.; Venugopalan, P. *CrystEngComm* **2006**, *8*, 215. $[Co(NH_3)_6]_3Cl_4(N_3)_5$ |
| NALCOT | 1.953 Å | Brayshaw, P. A.; Hall, A. K.; Harrison, W. T. A.; Harrowfield, J. M.; Pearce, D.; Shand, T. M.; Skelton, B. W.; Whitaker, C. R.; White, A. H. *Eur. J. Inorg. Chem.* **2005**, 1127. $[Co(NH_3)_6][Tm(C_7H_3NO_4)_3]\cdot 8.5H_2O$ |
| VUCDOO | 1.953 Å | Charushnikova, L. A.; Krot, N. N.; Makarenkov, V. I.; Starikova, Z. A. *Radiochim.* **2014**, *56*, 364. $[Co(NH_3)_6]_2[Er_3C_{24}H_{25}O_{36}]\cdot 6H_2O$ |
| YUDJOY | 1.954 Å | Pook, N.-P.; Adam, A. *Z. Anorg. Allg. Chem.* **2014**, *640*, 2931. $K[Co(NH_3)_6][Ce_2C_{20}H_{24}N_4O_{20}]\cdot 7H_2O$ |
| 92479 | 1.955 Å | Staehler, O.; Preetz, W. *Z. Anorg. Allg. Chem.* **2000**, *626*, 2077. $[Co(NH_3)_6][Os(SCN)_6]$ |
| 415361 | 1.955 Å | Sharma, R. P.; Bala, R.; Sharma, R.; Venugopalan, P.; Salas, J. M.; Quiros, M. *J. Fluor. Chem.* **2005**, *126*, 1543. $[Co(NH_3)_6]Cl_2(BF_4)$ |
| EXEHIY | 1.955 Å | Abrahams, B. F.; Haywood, M. G.; Robson, R. *Chem.Commun.* **2004**, 938. $[[Co(NH_3)_6]3[NpO_2(SeO_4)_2(H_2O)_2]\cdot 2H_2O$ |
| GEWNOP | 1.955 Å | Fonari, M.; Kravtsov, V. CCDC deposition number 2167982, **2023**. $[Co(NH_3)_6](C_{15}H_{14}NO_2)_2Cl\cdot 2H_2O$ |
| MEHSEB | 1.955 Å | Darii, M.; Beleaev, E. S.; Kravtsov, V. C.; Bourosh, P.; Chumakov, Y.; Hauser, J.; Decurtins, S.; Liu, S.-X.; Sultanova, O.; Baca, S. G. *New J. Chem.* **2022**, *46*, 11404. $[Co(NH_3)_6]$(*o*-$HOOCC_6H_4COO)Cl_2\cdot 3H_2O$ |
| 66984 | 1.956 Å | Grigor'ev, M. S.; Plotnikova, T. E.; Budantseva, N. A.; Fedoseev, A. M.; Yanovskii, A. I.; Struchkov, Y. T. *Radiokhim.* **1992**, *34*, 1. $[Co(NH_3)_6][CuC_8HO_{25})]\cdot 2H_2O$ |
| 280485 | 1.956 Å | Figgis, B.; Sobolev, A.; Reynolds, P. *Acta Crystallogr., Sect. B* **1998**, *54*, 613. $NH_4[Co(NH_3)_6](ClO_4)_2Cl_2$ |

| | | |
|---|---|---|
| 280860 | 1.956 Å | Jiang, J.-F.; Acunzo, A.; Koch, S. A. *J. Am. Chem. Soc.* **2001**, *123*, 12109. $[Co(NH_3)_6][Fe(CN)_5(CO)]\cdot 4H_2O$ |
| AFIKOQ | 1.956 Å | Wang, X.-Y.; Justice, R.; Sevov, S. C. *Inorg. Chem.* **2007**, *46*, 4626. $[Co(NH_3)_6]_2(O_3SC_6H_4C_6H_4SO_3)_3\cdot 2CH_3CN\cdot 5H_2O$ |
| CAXYIM | 1.956 Å | Harrowfield, J. M. Ling, I.; Skelton, B. W.; Sobolev, A. N.; White, A. H. *Aust. J. Chem.* **2017**, *70*, 485. $[Co(NH_3)_6][Gd(C_7H_3NO_4)_3]\cdot 8H_2O$ |
| GEWNIJ | 1.956 Å | Fonari, M.; Kravtsov, V. CCDC deposition number 2167981, **2023**. $[Co(NH_3)_6](C_3H_2N_3O_3)_2Cl\cdot H_2O$ |
| PEWCOM | 1.956 Å | Cindric, M.; Stilinovic, V.; Rubcic, M.; Medak, G.; Jung, D. S.; Vrdoljak, V. *CrystEngComm* **2018**, *20*, 1889. $[Co(NH_3)_6]_2[Mo_4O_{11}(H_2O)(C_2O_4)_4]\cdot 6H_2O$ |
| PUJSIZ | 1.956 Å | Engelberg, N.; Bino, A.; Tshuva, E. Y. *Inorg. Chim. Acta.* **2020**, *503*, 119429. $[Co(NH_3)_6]_3[Ti_4C_{24}H_{19}O_{34}]\cdot 20H_2O$ |
| 240985 | 1.957 Å | Gubanov, A. I.; Kuratieva, N. V. *Acta Crystallogr., Sect. C* **2007**, *63*, i83. $[Co(NH_3)_6]_2[Zr_3F_{18}]\cdot 2H_2O$ |
| 411630 | 1.957 Å | Seitz, K.; Peschel, S.; Babel, D. *Z. Anorg. Allg. Chem.* **2001,** *627*, 929. $[Co(NH_3)_6][Fe(CN)_6]$ |
| 427471 | 1.957 Å | Halevi, O.; Bogoslavsky, B.; Grinstein, D.; Tibika-Apfelbaum, F.; Bino, A. *Inorg. Chim. Acta* **2014**, *421*, 228. $[Co(NH_3)_6]_2[Ru_2N(N_3)_{10}]\cdot 9.75H_2O$ |
| TAPWIR | 1.957 Å | Sharma, R. P.; Bala, R.; Sharma, R.; Bond, A. D. *Acta Crystallogr., Sect. C* **2005**, *61*, m356. $[Co(NH_3)_6](NaC_{21}H_{14}F_3O_7)]$(*p*-$FC_6H_4COO$) |
| VUCDII | 1.957 Å | Charushnikova, L. A.; Krot, N. N.; Makarenkov, V. I.; Starikova, Z. A. *Radiochim.* **2014**, *56*, 364. $[Co(NH_3)_6]_2[Tb_3C_{24}H_{25}O_{36}]\cdot 6H_2O$ |
| 8071 | 1.958 Å | Mauersberger, P.; Haupt, H. J.; Huber, F. *Acta Crystallogr., Sect. B* **1979**, *35*, 295. $[Co(NH_3)_6][Pb_4Cl_{11}]$ |
| 90931 | 1.958 Å | Reynolds, P. A.; Henning, R. W.; Schultz, A. J. *J. Solid State Chem.* **2000**, *149*, 60. $Cs[Co(ND_3)_6](ClO_4)_2Cl_2$ |
| 262783 | 1.958 Å | Wu Q.; Du C.; Lv Y.; Chen G.; Pan Q. *Acta Crystallogr., Sct. E* **2012**, *68*, i45. $[Co(NH_3)_6]_2Cl_5(NO_3)$ |
| CANYEW | 1.958 Å | Sharma, R. P.; Bala, R.; Sharma, R.; Kariuki, B. M.; Rychlewska, U.; Warzajtis, B. *J. Mol. Struct.* **2005**, *748*, 143. $[Co(NH_3)_6][NaC_{21}H_{17}O_7])C_6H_5COO$ |
| EYAQEA | 1.958 Å | Sharma, R. P.; Bala, R.; Sharma, R.; Venugopalan, P. *J. Mol. Struct.* **2004**, *694*, 229. $[Co(NH_3)_6](CH_3SO_3)_2Cl$ |
| GEZZIU | 1.958 Å | Takusagawa, F.; Shaw, J.; Everett, G. W. *Inorg. Chem.* **1988**, *27*, 3107. $[Co(NH_3)_6]_3$(*p*-$HOC_6H_4SO_3)_8Cl\cdot 13H_2O$ |
| KEQGOD | 1.958 Å | Sharma, R. P.; Bala, R.; Sharma, R.; Bond, A. D. *Acta Crystallogr., Sect. E* **2006**, *62*, m2113. $[Co(NH_3)_6](C_{34}H_{53}O_8)_3\cdot 3.3H_2O$ |
| MEHTAY | 1.958 Å | Darii, M.; Beleaev, E. S.; Kravtsov, V. C.; Bourosh, P.; Chumakov, Y.; Hauser, J.; Decurtins, S.; Liu, S.-X.; Sultanova, O.; Baca, S. G. *New J. Chem.* **2022**, *46*, 11404. $[Co(NH_3)_6]_2$(*o*-$_3OSC_6H_4COO)_3)\cdot C_2H_5OH\cdot 2.5H_2O$ |

| | | |
|---|---|---|
| MEHTIG | 1.958 Å | Darii, M.; Beleaev, E. S.; Kravtsov, V. C.; Bourosh, P.; Chumakov, Y.; Hauser, J.; Decurtins, S.; Liu, S.-X.; Sultanova, O.; Baca, S. G. *New J. Chem.* **2022**, *46*, 11404. $[Co(NH_3)_6]_{11}[Co(C_7H_3NO_4))_3]_8Cl\cdot 84H_2O$ |
| NALCUZ | 1.958 Å | Brayshaw, P. A.; Hall, A. K.; Harrison, W. T. A.; Harrowfield, J. M.; Pearce, D.; Shand, T. M.; Skelton, B. W.; Whitaker, C. R.; White, A. H. *Eur. J. Inorg. Chem.* **2005**, 1127. $[Co(NH_3)_6][Lu(C_7H_3NO_4)_3]\cdot 8.5H_2O$ |
| OTEXER | 1.958 Å | Geisheimer, A. R.; Wren, J. E. C.; Michaelis, V. K.; Kobayashi, M.; Sakai, K.; Kroeker, S.; Leznoff, D. B. *Inorg. Chem.* **2011**, *50*, 1265. $[Co(NH_3)_6][Au(CN)_4]_3\cdot 4H_2O$ |
| QAGJUF | 1.958 Å | Fedosseev, A. M.; Grigoriev, M. S.; Budantseva, N. A.; Guillaumont, D.; Le Naour, C.; Simoni, E.; Den Auwer, C.; Moisy, P. *Compt. Rend. Chim.* **2010**, *13*, 839. $K[Co(NH_3)_6]_2[Nd_3C_{24}H_{22}O_{31}]\cdot 18H_2O$ |
| REMMEC | 1.958 Å | Bala, R.; Sharma, R. P.; Sharma, R.; Kariuki, B. M. *Inorg. Chem. Commun.* **2006**, *9*, 852. $[Co(NH_3)_6)_3][Hg_2(SCN)_7]$ |
| 83480 | 1.959 Å | Morgan, K. R.; Gainsford, G. J.; Milestone, N. B. *Chem. Commun.* **1997**, 61. $(NH_4)_3[Co(NH_3)_6)_3][Al_2(PO_4)_4]_2\cdot 2H_2O$ |
| 90933 | 1.959 Å | Reynolds, P. A.; Henning, R. W.; Schultz, A. J. *J. Solid State Chem.* **2000**, *149*, 60. $Cs[Co(ND_3)_6](ClO_4)_2Cl_2$ |
| 170950 | 1.959 Å | Abrahams, B. F.; Haywood, M. G.; Robson, R. *Chem. Commun.* **2004**, 938. $[Co(NH_3)_6]_3[Cu_4(OH)(CO_3)_8]\cdot 2H_2O$ |
| 415732 | 1.959 Å | Sharma, R. P.; Bala, R.; Sharma, R.; Perez, J.; Miguel, D. *J. Mol. Struct.* **2006**, *788*, 49. $[Co(NH_3)_6](BrO_3)_3\cdot 0.5H_2O$ |
| MEHSOL | 1.959 Å | Darii, M.; Beleaev, E. S.; Kravtsov, V. C.; Bourosh, P.; Chumakov, Y.; Hauser, J.; Decurtins, S.; Liu, S.-X.; Sultanova, O.; Baca, S. G. *New J. Chem.* **2022**, *46*, 11404. $[Co(NH_3)_6](C_7H_3NO_4)Cl\cdot 2H_2O$ |
| NAKZUV | 1.959 Å | Brayshaw, P. A.; Hall, A. K.; Harrison, W. T. A.; Harrowfield, J. M.; Pearce, D.; Shand, T. M.; Skelton, B. W.; Whitaker, C. R.; White, A. H. *Eur. J. Inorg. Chem.* **2005**, 1127. $[Co(NH_3)_6](C_7H_3NO_4)Cl\cdot 2H_2O$ |
| NALBUY | 1.959 Å | Brayshaw, P. A.; Hall, A. K.; Harrison, W. T. A.; Harrowfield, J. M.; Pearce, D.; Shand, T. M.; Skelton, B. W.; Whitaker, C. R.; White, A. H. *Eur. J. Inorg. Chem.* **2005**, 1127. $[Co(NH_3)_6][Sm(C_7H_3NO_4)_3]\cdot 10H_2O$ |
| NALCAF | 1.959 Å | Brayshaw, P. A.; Hall, A. K.; Harrison, W. T. A.; Harrowfield, J. M.; Pearce, D.; Shand, T. M.; Skelton, B. W.; Whitaker, C. R.; White, A. H. *Eur. J. Inorg. Chem.* **2005**, 1127. $[Co(NH_3)_6][Tb(C_7H_3NO_4)_3]\cdot 10H_2O$ |
| NALCOT01 | 1.959 Å | Harrowfield, J. M.; Ling, I.; Skelton, B. W.; Sobolev, A. N.; White, A. H. *Aust. J. Chem.* **2017**, 70, 485. $[Co(NH_3)_6][Tm(C_7H_3NO_4)_3]\cdot 8.5H_2O$ |
| NEKHUK | 1.959 Å | Pyrch, M. M. F.; Bjorklund, J. L.; Williams, J. M.; Kasperski, M.; Mason, S. E.; Forbes, T. Z. *Inorg. Chem.* **2023**, *62*, 15023. $[Co(NH_3)_6]_2[(UO_2)(CO_3)_3]CO_3\cdot 3H_2O$ |
| SANCOA | 1.959 Å | Grigor'ev, M. S.; Charushnikova, I. A.; Starikova, Z. A.; Krot, N. N.; Polyakova, I. N. *Radiokhimiya* **2004**, *46*, 212. $[Co(NH_3)_6][NpO_2(C_3H_2O_4)](HOOCCH_2COO)$ |
| SICHAO | 1.959 Å | Klufers, P.; Mayer, P. *Z. Anorg. Allg. Chem.* **2007**, *633*, 903. $[Co(NH_3)_6][Bi(C_{10}H_{10}N_5O_5)_2]\cdot 2H_2O$ |

| | | |
|---|---|---|
| SUTYUC | 1.959 Å | Fedosseev, A. M.; Grigoriev, M. S.; Budantseva, N. A.; Guillaumont, D.; Le Naour, C.; Simoni, E.; Den Auwer, C.; Moisy, P. *Compt. Rendes Chim.* **2010**, *13*, 839. $[Co(NH_3)_6](Nd(C_6H_6NO_6)_2(H_2O)]\cdot 8H_2O$ |
| VUCFAC | 1.959 Å | Charushnikova, L. A.; Krot, N. N.; Makarenkov, V. I.; Starikova, Z. A. *Radiochim.* **2014**, *56*, 364. $[Co(NH_3)_6]_2[Ho_3C_{24}H_{25}O_{36}]\cdot 6H_2O$ |
| WABYAA | 1.959 Å | Reddy, D. S.; Duncan, S.; Shimizu, G. K. H. *Angew. Chem., Int. Ed.* **2003**, *42*, 1360. $[Co(NH_3)_6]_2(C_{10}H_6O_6S_2)_3\cdot 2C_4H_8O_2\cdot 2.5H_2O$ |
| 26068 | 1.960 Å | Epstein, E. F.; Bernal, I. *J. Chem. Soc. A* **1971**, 3628. $[Co(NH_3)_6][CdCl_5]$ |
| 59268 | 1.960 Å | Sobolev, A. N.; Figgis, B. N. *Acta Crystallogr., Sect. C* **1997**, *53*, 661. $[Co(NH_3)_6](S_2O_2)_3Cl\cdot H_2O$ |
| 154025 | 1.960 Å | Grigor'ev, M. S.; Antipin, M. Y.; Krot, N. N.; Bessonov, A. A. *Radiokhimiya* **2005**, *47*, 419. $[Co(NH_3)_6]_2[NpO_2(C_2O_4)_3]\cdot 3H_2O$ |
| 187076 | 1.960 Å | Khranenko, S. P.; Alexeyev, A. V.; Naumov, D. Y.; Plusnin, P. E.; Gromilov, S. A. *J. Struct. Chem.* **2012**, *53*, 748. $[Co(NH_3)_6](WO_4)Cl$ |
| 201236 | 1.960 Å | Figgis, B. N.; Skelton, B. W.; White, A. H. *Aust. J. Chem.* **1979**, *32*, 417. $[Co(NH_3)_6](CrO_4)Cl\cdot 3H_2O$ |
| 414607 | 1.960 Å | Sharma, R. P.; Bala, R.; Sharma, R.; Ferretti, V. *Inorg. Chim. Acta* **2005**, *358*, 3457. $[Co(NH_3)_6]Cl_2(ClO_3)$ |
| 420455 | 1.960 Å | Filatov, E. Y.; Yusenko, K. V.; Vikulova, E. S.; Plyusnin, P. E.; Shubin, Y. V. *Z. Kristallogr., Suppl. Issue* **2009**, *30*, 263-268. $[Co(NH_3)_6][Ir(C_2O_4)_3]$ |
| 421211 | 1.960 Å | Sharma, R. P.; Singh, A.; Saini, A.; Venugopalan, P.; Ferretti, V. *Inorg. Chem. Commun.* **2011**, *14*, 1. $[Co(NH_3)_6][HgBr_4Cl]$ |
| 431385 | 1.960 Å | Charushnikov, I. A.; Krot, N. N.; Grigor’ev, M. S.; Makarenkov, V. I. *Radiochem.* **2017**, *59*, 124. $[Co(NH_3)_6]_3[NpO_4(OH)_2)_2]_3\cdot 4H_2O$ |
| ADIYIW | 1.960 Å | Wang, X.-Y.; Justice, R.; Sevov, S. C. *Inorg. Chem.* **2007**, *46*, 4626. $[Co(NH_3)_6]_2(O_3SC_6H_4C_6H_4SO_3)_3\cdot(CH_3)_2NCHO\cdot 2H_2O$ |
| NALCIN | 1.960 Å | Brayshaw, P. A.; Hall, A. K.; Harrison, W. T. A.; Harrowfield, J. M.; Pearce, D.; Shand, T. M.; Skelton, B. W.; Whitaker, C. R.; White, A. H. *Eur. J. Inorg. Chem.* **2005**, 1127. $[Co(NH_3)_6][Y(C_7H_3NO_4)_3]\cdot 10H_2O$ |
| NALCUZ01 | 1.960 Å | Harrowfield, J. M.; Ling, I.; Skelton, B. W.; Sobolev, A. N.; White, A. H. *Aust. J. Chem.* **2017**, 70, 485. $[Co(NH_3)_6][Lu(C_7H_3NO_4)_3]\cdot 8.5H_2O$ |
| NEKJEW | 1.960 Å | Pyrch, M. M. F.; Bjorklund, J. L.; Williams, J. M.; Kasperski, M.; Mason, S. E.; Forbes, T. Z. *Inorg. Chem.* **2023**, *62*, 15023. $[Co(NH_3)_6]_2[(UO_2)(CO_3)_3]Cl_2\cdot H_2O$ |
| PEWCIG | 1.960 Å | Cindric, M.; Stilinovic, V.; Rubcic, M.; Medak, G.; Jung, D. S.; Vrdoljak, V. *CrystEngComm* **2018**, *20*, 1889. $[Co(NH_3)_6]_4[MoO_2)_4(C_2O_4)_4][MoO_3(H_2O)(C_2O_4)]_2\cdot 10H_2O$ |
| ADIYES | 1.961 Å | Wang, X.-Y.; Justice, R.; Sevov, S. C. *Inorg. Chem.* **2007**, *46*, 4626. $[Co(NH_3)_6]_2(O_3SC_6H_4C_6H_4SO_3)_3\cdot 2(CH_3)_2SO\cdot 5H_2O$ |

| | | |
|---|---|---|
| ADIYOC | 1.961 Å | Wang, X.-Y.; Justice, R.; Sevov, S. C. *Inorg. Chem.* **2007**, *46*, 4626. $[Co(NH_3)_6]_2(O_3SC_6H_4C_6H_4SO_3)_3\cdot C_2H_{11}N\cdot 5H_2O$ |
| CANYAS01 | 1.961 Å | Darii, M.; Beleaev, E. S.; Kravtsov, V. C.; Bourosh, P.; Chumakov, Y.; Hauser, J.; Decurtins, S.; Liu, S.-X.; Sultanova, O.; Baca, S. G. *New J. Chem.* **2022**, *46*, 11404. $[Co(NH_3)_6](o\text{-}HOOCC_6H_4COO)_2Cl\cdot 3H_2O$ |
| CAXPUP | 1.961 Å | Harrowfield, J. M. Ling, I.; Skelton, B. W.; Sobolev, A. N.; White, A. H. *Aust. J. Chem.* **2017**, *70*, 485. $[Co(NH_3)_6][Pu(C_7H_3NO_4)_3]\cdot 8.5H_2O$ |
| DERYEF | 1.961 Å | Grigoriev, M. S.; Krot, N. N.; Bessonov, A. A.; Lyssenko, K. A. *Acta Crystallogr., Sect. E* **2006**, *62*, m2889. $Li[Co(NH_3)_6][Pu(C_3H_2O_4)_4]\cdot 5H_2O$ |
| NALBEI | 1.961 Å | Brayshaw, P. A.; Hall, A. K.; Harrison, W. T. A.; Harrowfield, J. M.; Pearce, D.; Shand, T. M.; Skelton, B. W.; Whitaker, C. R.; White, A. H. *Eur. J. Inorg. Chem.* **2005**, 1127. $[Co(NH_3)_6][Ce(C_7H_3NO_4)_3]\cdot 5H_2O$ |
| MEHRUQ | 1.961 Å | Darii, M.; Beleaev, E. S.; Kravtsov, V. C.; Bourosh, P.; Chumakov, Y.; Hauser, J.; Decurtins, S.; Liu, S.-X.; Sultanova, O.; Baca, S. G. *New J. Chem.* **2022**, *46*, 11404. $[Co(NH_3)_6]Cl_3\cdot 2C_{12}H_8N_2\cdot 3H_2O$ |
| NALCIN01 | 1.961 Å | Harrowfield, J. M.; Ling, I.; Skelton, B. W.; Sobolev, A. N.; White, A. H. *Aust. J. Chem.* **2017**, 70, 485. $[Co(NH_3)_6][Y(C_7H_3NO_4)_3]\cdot 10H_2O$ |
| OKOCAS | 1.961 Å | Charushnikova, I. A.; Krot, N. N.; Starikova, Z. A. *Radiokhim.* **2001**, *43*, 438. $[Co(NH_3)_6][NpO_2C_{16}H_8O_8]\cdot 2H_2O$ |
| SIWZAZ | 1.961 Å | Chen Ming-Qin; Xu Jun-Xing; Zhang Hua-Ling *Jiegou Huaxue* **1991**, *10*, 1. $[Co(NH_3)_6][Co(C_9H_9NO_3)_2]Cl\cdot 10H_2O$ |
| XECQUT | 1.961 Å | Tian, R.; Yan, Y.; Zhang, C.; Wang, L. Pan, Q. *Acta Crystallogr., Sect. E* **2012**, *68*, m914. $[Co(NH_3)_6]_3[Co(C_2O_4)_3]_2Cl\cdot 12H_2O$ |
| ZOSQAD | 1.961 Å | Karn, L. M.; Britton, A.; Leznoff, D. B. *Inorg. Chem.* **2024**, *63*, 11977. $[Co(NH_3)_6]_2[Pt(SCN)_4]_3\cdot 5H_2O$ |
| 15524 | 1.962 Å | Herlinger, A. W.; Brown, J. N.; Dwyer, M. A.; Pavkovic, S. F. *Inorg. Chem.* **1981**, *20*, 2366. $[Co(NH_3)_6][HgCl_5]$ |
| 90930 | 1.962 Å | Reynolds, P. A.; Henning, R. W.; Schultz, A. J. *J. Solid State Chem.* **2000**, *149*, 60. $Cs[Co(ND_3)_6](ClO_4)_2Cl_2$ |
| 414606 | 1.962 Å | Sharma, R. P.; Bala, R.; Sharma, R.; Ferretti, V. *Inorg. Chim. Acta* **2005**, *358*, 3457. $[Co(NH_3)_6]Br_2(ClO_3)$ |
| BEFWIW | 1.962 Å | Tamain, C.; Autillo, M.; Guillaumont, D.; Guerin, L.; Wilson, R. E.; Berthon, C. *Inorg. Chem.* **2022**, *61*, 12337. $[Co(NH_3)_6]_2[Np(C_2O_4)_5]\cdot 4H_2O$ |
| CAXQOQ | 1.962 Å | Harrowfield, J. M. Ling, I.; Skelton, B. W.; Sobolev, A. N.; White, A. H. *Aust. J. Chem.* **2017**, *70*, 485. $[Co(NH_3)_6][Ce(C_7H_3NO_4)_3]\cdot 8.5H_2O$ |
| DOGQUP | 1.962 Å | Lagunova, V.; Rubilkin, P.; Filatov, E.; Plyusnin, P.; Kuratieva, N.; Korenev, S. *New. J. Chem.* **2024**, *48*, 1578. $[Co(NH_3)_6]_2[Cu_3C_{12}H_4O_{26}]\cdot 2H_2O$ |

| | | |
|---|---|---|
| HEQNAT | 1.962 Å | Bernhardt, E.; Brauer, D. J.; Finze, M.; Willner, H. *Angew. Chem., Int. Ed.* **2006**, *45*, 6383. $[Co(NH_3)_6]B(COO)_2(COOH)_2 \cdot 2H_2O$ |
| NALCEJ | 1.962 Å | Brayshaw, P. A.; Hall, A. K.; Harrison, W. T. A.; Harrowfield, J. M.; Pearce, D.; Shand, T. M.; Skelton, B. W.; Whitaker, C. R.; White, A. H. *Eur. J. Inorg. Chem.* **2005**, 1127. $[Co(NH_3)_6][Er(C_7H_3NO_4)_3] \cdot 10H_2O$ |
| SUTYOW | 1.962 Å | Fedosseev, A. M.; Grigoriev, M. S.; Budantseva, N. A.; Guillaumont, D.; Le Naour, C.; Simoni, E.; Den Auwer, C.; Moisy, P. *Compt. Rendes Chim.* **2010**, *13*, 839. $[Co(NH_3)_6](Yb(C_6H_6NO_6)_2(H_2O)] \cdot 8H_2O$ |
| WUBDEF | 1.962 Å | Altahan, M. A.; Beckett, M. A.; Coles, S. J.; Horton, P. N. *J. Mol. Struct.* **2020**, *1200*, 127071. $[Co(NH_3)_6]_2(H_4B_4O_9)_3 \cdot 11H_2O$ |
| XATROA | 1.962 Å | Charushnikova, I. A.; Krot, N. N.; Starikova, Z. A. *Radiokhimiya* **2004**, *46*, 521. $[Co(NH_3)_6][NpO_2(H_2O)_2(C_4H_4O_4)_2](OOCCHCHCOOH)_2 \cdot H_2O$ |
| YOMNOF | 1.962 Å | Halevi, O.; Bogoslavsky, B.; Grinstein, D.Tibika-Apfelbaum, F.; Bino, A. *Inorg. Chim. Acta* **2014**, *421*, 228. $[Co(NH_3)_6][RuN(O_2(CH_2)_2)_2]$ |
| 63358 | 1.963 Å | Grigor'ev, M. S.; Gulev, B. F.; Krot, N. N. *Radiokhimiya* **1986**, *28*, 690. $[Co(NH_3)_6][NpO_4(OH)_2] \cdot 2H_2O$ |
| 158113 | 1.963 Å | Bala, R.; Sharma, R. P.; Sharma, R.; Kariuki, B. M. *Inorg. Chem. Commun.* **2006**, *9*, 852. $[Co(NH_3)_6][(SCN)(Hg(SCN)_3)_2]$ |
| 162711 | 1.963 Å | Matikova-Malarova, M.; Cernak, J.; Massa, W.; Varret, F. *Inorg. Chim. Acta* **2009,** *362*, 443. $[Co(NH_3)_6]_2[Fe(CN)_6]Cl_2 \cdot 4H_2O$ |
| 411045 | 1.963 Å | Bond, D. L.; Clark, D. L.; Donohoe, R. J.; Gordon, J. C.; Gordon, P. L.; Keogh, D. W.; Scott, B. L.; Drew Tait, C.; Watkin, J. G. *Eur. J. Inorg. Chem.* **2001**, 2921. $[Co(NH_3)_6][Na(H_2O)(H_2O)_4]_2[Ho(CO_3)_4(H_2O)_4]$ |
| 411070 | 1.963 Å | Adam, A.; Dahm, M. *Z. Anorg. Allg. Chem.* **2000**, *626*, 494. $[Co(NH_3)_6]_2[Be_4O(CO_3)_6] \cdot 10H_2O$ |
| 415917 | 1.963 Å | Bala, R.; Sharma, R. M.A.Altahan, M.A.Beckett, S.J.Coles, P.N.Horton Sharma, U.; Burrows, A. D.; Cassar, K. *J. Mol. Struct.* **2007**, *832*, 156. $[Co(NH_3)_6]Br_2(BF_4)$ |
| 431384 | 1.963 Å | Charushnikov, I. A.; Krot, N. N.; Grigor'ev, M. S.; Makarenkov, V. I. *Radiochem.* **2017**, *59*, 124. $[Co(NH_3)_6][NpO_4(OH)_2)_2] \cdot H_2O_2$ |
| BEFWES | 1.963 Å | Tamain, C.; Autillo, M.; Guillaumont, D.; Guerin, L.; Wilson, R. E.; Berthon, C. *Inorg. Chem.* **2022**, *61*, 12337. $[Co(NH_3)_6]_2[U(C_2O_4)_5] \cdot 4H_2O$ |
| BEFWOC | 1.963 Å | Tamain, C.; Autillo, M.; Guillaumont, D.; Guerin, L.; Wilson, R. E.; Berthon, C. *Inorg. Chem.* **2022**, *61*, 12337. $[Co(NH_3)_6]_2[Pu(C_2O_4)_5] \cdot 4H_2O$ |
| CAXQUQ | 1.963 Å | Harrowfield, J. M. Ling, I.; Skelton, B. W.; Sobolev, A. N.; White, A. H. *Aust. J. Chem.* **2017**, *70*, 485. $[Co(NH_3)_6][Gd(C_7H_3NO_4)_3] \cdot 10H_2O$ |
| CAXXEH | 1.963 Å | Harrowfield, J. M. Ling, I.; Skelton, B. W.; Sobolev, A. N.; White, A. H. *Aust. J. Chem.* **2017**, *70*, 485. $[Co(NH_3)_6][Nd(C_7H_3NO_4)_3] \cdot 10H_2O$ |

| | | |
|---|---|---|
| CAXXIL | 1.963 Å | Harrowfield, J. M. Ling, I.; Skelton, B. W.; Sobolev, A. N.; White, A. H. *Aust. J. Chem.* **2017**, *70*, 485. $[Co(NH_3)_6][Eu(C_7H_3NO_4)_3]\cdot 10H_2O$ |
| MEHSUR | 1.963 Å | Darii, M.; Beleaev, E. S.; Kravtsov, V. C.; Bourosh, P.; Chumakov, Y.; Hauser, J.; Decurtins, S.; Liu, S.-X.; Sultanova, O.; Baca, S. G. *New J. Chem.* **2022**, *46*, 11404. $[Co(NH_3)_6](\textit{o}\text{-}O_3SC_6H_4COO)Cl\cdot 4H_2O$ |
| NALCAF01 | 1.963 Å | Harrowfield, J. M.; Ling, I.; Skelton, B. W.; Sobolev, A. N.; White, A. H. *Aust. J. Chem.* **2017**, 70, 485. $[Co(NH_3)_6][Tb(C_7H_3NO_4)_3]\cdot 10H_2O$ |
| NEKJAS | 1.963 Å | Pyrch, M. M. F.; Bjorklund, J. L.; Williams, J. M.; Kasperski, M.; Mason, S. E.; Forbes, T. Z. *Inorg. Chem.* **2023**, *62*, 15023. $[Co(NH_3)_6](CO_3)Cl$ |
| RENVIQ | 1.963 Å | Sharma, R. P.; Bala, R.; Sharma, R.; Perez, J.; Miguel, D. *J. Mol. Struct.* **2006**, *979*, 49. $[Co(NH_3)_6](\textit{p}\text{-}O_2NC_6H_4COO)_2Cl$ |
| TUTMAZ | 1.963 Å | Wang, F.-X.; Ren, G.-J.; Tian, R.-J.; Feng, L-.J.; Yang, Y.-H.; Deng, Y-Y.; Pan, Q.-H. *Chin. J. Struct. Chem.* **2020**, *39*, 1337. $[Co(NH_3)_6][Cd_2C_{18}H_6ClO_{12}]\cdot 3H_2O$ |
| WABXUT | 1.963 Å | Reddy, D. S.; Duncan, S.; Shimizu, G. K. H. *Angew. Chem., Int. Ed.* **2003**, *42*, 1360. $[Co(NH_3)_6](C_8H_{16}N_2O_6S_2)Cl\cdot 6H_2O$ |
| WEHBIV | 1.963 Å | Grigor'ev, M. S.; Antipin, M. Y.; Krot, N. N.; Bessonov, A. A. *Radiokhimiya* **2005**, *47*, 419. $[Co(NH_3)_6][(PuO_2)_2C_8O_{16}]\cdot 6H_2O$ |
| YAVZUR | 1.963 Å | Kurachi, S.; Ohba, S. *Bull. Chem. Soc. Jpn.* **1992**, *65*, 3033. $[Co(NH_3)_6](OC_6H_4NO_2)_3\cdot 4H_2O$ |
| 806 | 1.964 Å | Iwata, M. *Acta Crystallogr., Sect. B* **1977**, *33*, 59. $[Co(NH_3)_6][Cr(CN)_6]$ |
| 2560 | 1.964 Å | Iwata, M.; Saito, Y. *Acta Crystallogr., Sect. B* **1973**, *29*, 822. $[Co(NH_3)_6][Co(CN)_6]$ |
| 39550 | 1.964 Å | Grigor'ev, M. S.; Fedoseev, A. M.; Budantseva, N. A.; Yanovskii, A. I.; Struchkov, Y. T.; Krot, N. N. *Radiokhimiya* **1991**, *33*, 54. $[Co(NH_3)_6](H_8O_3)[NpO_2SO_4)_3]$ |
| 109859 | 1.964 Å | Grigor'ev, M. S.; Baturin, N. A.; Regel', L. L.; Krot, N. N. *Radiokhimiya* **1991**, *33*, 19. $[Co(NH_3)_6]_2[NpO_2(C_2O_4)_2]_2\cdot 6H_2O$ |
| 109860 | 1.964 Å | Grigor'ev, M. S.; Baturin, N. A.; Regel', L. L.; Krot, N. N. *Radiokhimiya* **1991**, *33*, 19. $[Co(NH_3)_6]_2[NpO_2(C_2O_4)_2H_2O]_2\cdot 3H_2O$ |
| 109997 | 1.964 Å | Gorol, M.; Mosch-Zanetti, N. C.; Noltemeyer, M.; Roesky, H. W. *Z. Anorg. Allg. Chem.* **2000**, *626*, 2318. $[Co(NH_3)_6]_2[NpO_2(C_2O_4)_3]\cdot 4H_2O$ |
| 161487 | 1.964 Å | Makotchenko, E. V.; Baidina, I. A.; Plyusnin, P. E. *Zh. Strukt. Khim.* **2007**, *48*, 282. $[Co(NH_3)_6][AuCl_4]Cl_2$ |
| 262942 | 1.964 Å | Tian R.; Yan Y.; Zhang C.; Wang L.; Pan Q. *Acta Crystallogr., Sect. E* **2012**, *68*, m914. $[Co(NH_3)_6]_3[Co(C_2O_4)_3]_2Cl_2\cdot 12H_2O$ |
| 280486 | 1.964 Å | Figgis, B.; Sobolev, A.; Reynolds, P. *Acta Crystallogr., Sect. B* **1998**, *54*, 613. $NH_4[Co(NH_3)_6](ClO_4)_2Cl_2$ |

| | | |
|---|---|---|
| 416414 | 1.964 Å | Bala, R.; Sharma, R. P.; Sharma, R.; Salas, J. M.; Quiros, M.; Harrison, W. T. A. *J. Mol. Struct.* **2007**, *828*, 174-180. $[Co(NH_3)_6]_2(HAsO_4)_3\cdot4H_2O$ |
| 411631 | 1.964 Å | Seitz, K.; Peschel, S.; Babel, D. *Z. Anorg. Allg. Chem.* **2001,** *627*, 929. $[Co(NH_3)_6]_2[Ni(CN)_6]\cdot2H_2O$ |
| 421686 | 1.964 Å | Baidina, I. A.; Filatov, E. Y.; Makotchenko, E. V.; Smolentsev, A. I. *J. Struct. Chem.* **2012**, *53*, 112. $[Co(NH_3)_6]_2(ReO_4)_3\cdot2H_2O$ |
| KUTCEH | 1.964 Å | Grigor'ev, M. S.; Baturin, N. A.; Regel', L. L.; Krot, N. N. *Radiokhimiya* **1991**, *33*, 19. $[Co(NH_3)_6]_2[Np_2O_4C_8O_{16}]\cdot6H_2O$ |
| SEDFEP | 1.964 Å | Heering, C.; Nateghi, B.; Janiak, C. *Crystals* **2016**, *6*, 22. $[Co(NH_3)_6]_2[C_{13}C_8O_5P]\cdot4H_2O$ |
| UHUXII | 1.964 Å | B.Pramanik, R.Sahoo, Y.Yoshida, A.K.Manna, H.Kitagawa, M.C.Das, M. C. Cjhem. Eur. J. 2024, 30, e2024002896. $[Co(NH_3)_6]_2(C_{10}H_6(SO_3)_2)_3\cdot3H_2O$ |
| XEDNAV | 1.964 Å | Gorol, M.; Mosch-Zanetti, N. C.; Noltemeyer, M.; Roesky, H. W. *Z. Anorg. Allg. Chem.* **2000**, *626*, 2318. $[Co(NH_3)_6]_2(C_2O_4)_3\cdot4H_2O$ |
| 28153 | 1.965 Å | Murray-Rust, P. *Acta Crystallogr., Sect. B* **1973**, *29*, 2559. $[Co(NH_3)_6]_4[Cu_5Cl_{17}]$ |
| 94486 | 1.965 Å | Bernal, I.; Yufit, D. S.; Howard, J. A. K. *Z. Kristallogr.* **2001**, *216*, 413. $[Co(NH_3)_6][CuCl_5]$ |
| 101232 | 1.965 Å | Louis–Jean, J.; Balasekaran, S. M.; Hagenbach, A.; Poineau, F. *Acta Crystallogr., Sect E* **2019**, *75*, 1158. $[Co(NH_3)_6][ReO_4][ReF_6]\cdot6H_2O$ |
| 137752 | 1.965 Å | Filatov, Evgeny; Lagunova, Varvara; Kochetygov, Ilia; Plyusnin, Pavel; Kuratieva, Natalia; Kostin, Gennadiy; Korenev, S. *Acta Crystallogr., Sect. B* **2022**, *78*, 537. $K_3[Co(NH_3)_6][Ir(C_2O_4)_3]\cdot6H_2O$ |
| 413593 | 1.965 Å | Sharma, R. P.; Bala, R.; Sharma, R.; Salas, J. M.; Quiros, M. *J. Coord. Chem.* **2005**, *58*, 217. $[Co(NH_3)_6]Cl(Cr_2O_7)\cdot H_2O$ |
| 421508 | 1.965 Å | Domonov, D. P.; Kuratieva, N. V.; Pechenyuk, S. I. *J. Struct. Chem.* **2011**, *52*, 358. $[Co(NH_3)_6]_2(C_2O_4)_3\cdot4H_2O$ |
| 427577 | 1.965 Å | Avisar-Levy, M.; Levy, O.; Ascarelli, O.; Popov, I.; Bino, A. *J. Alloys Comp.* **2015**, *635*, 48. $[Co(NH_3)_6]_2[PtCl_4]_3$ |
| ADIYUI | 1.965 Å | Wang, X.-Y.; Justice, R.; Sevov, S. C. *Inorg. Chem.* **2007**, *46*, 4626. $[Co(NH_3)_6]_2(O_3SC_6H_4C_6H_4SO_3)_3\cdot2(CH_3)_2CO\cdot4H_2O$ |
| GEFGOO | 1.965 Å | Sharma, R. P.; Bala, R.; Sharma, R.; Venugopalan, P. *Cryst. Eng. Comm.* **2006**, *8*, 215. $[Co(NH_3)_6]Cl_2(SeCN)$ |
| MEHSAX | 1.965 Å | Darii, M.; Beleaev, E. S.; Kravtsov, V. C.; Bourosh, P.; Chumakov, Y.; Hauser, J.; Decurtins, S.; Liu, S.-X.; Sultanova, O.; Baca, S. G. *New J. Chem.* **2022**, *46*, 11404. $[Co(NH_3)_6](C_{14}H_8O_4)(C_{14}HO_4)\cdot3H_2O$ |
| MIWTUJ | 1.965 Å | Seidel, R. W.; Goddard, R.; Gramm, V.; Ruschewitz, U. *Z. Naturforsch., Teil B* **2014**, *69*, 277. $[Co(NH_3)_6]_2(OOCCCCOO)_3\cdot3H_2O$ |

| | | |
|---|---|---|
| NALBIM | 1.965 Å | Brayshaw, P. A.; Hall, A. K.; Harrison, W. T. A.; Harrowfield, J. M.; Pearce, D.; Shand, T. M.; Skelton, B. W.; Whitaker, C. R.; White, A. H. *Eur. J. Inorg. Chem.* **2005**, 1127. $[Co(NH_3)_6][Pr(C_7H_3NO_4)_3]\cdot 5H_2O$ |
| NANDAI | 1.965 Å | Charushnikova, I. A.; Krot, N. N.; Polyakova, I. N. *Radiokhimiya* **2004**, *46*, 318. $[Co(NH_3)_6][(NpO_2)_2C_6H_4O_8]OH\cdot H_2O$ |
| NEKHOE | 1.965 Å | Pyrch, M. M. F.; Bjorklund, J. L.; Williams, J. M.; Kasperski, M.; Mason, S. E.; Forbes, T. Z. *Inorg. Chem.* **2023**, *62*, 15023. $[Co(NH_3)_6]_4[(UO_2)(CO_3)_3]_3\cdot 11.67H_2O$ |
| QECTOL | 1.965 Å | Filatov, E.; Lagunova, V.; Kochetygov, I.; Plyusnin, P.; Kuratieva, N.; Kostin, G.; Korenev, S. *Acta Crystallogr., Sect. E* **2022**, *78*, 537. $K_3[Co(NH_3)_6][Ir(C_2O_4)_3]_2\cdot 6H_2O$ |
| RATDUL | 1.965 Å | Kofod, P.; Harris, P.; Larsen, S. *Inorg. Chem.* **1997**, *36*, 2258. $[Co(NH_3)_6][FeCl_6]$ |
| VAFKOD | 1.965 Å | Dalrymple, S. A.; Parvez, M.; Shimizu, G. K. H. *Inorg. Chem.* **2002**, *41*, 6986. $[Co(NH_3)_6]$(*1,3,5*-$C_6H_3(CH_2SO_3)_3\cdot 3H_2O$ |
| 1694 | 1.966 Å | Kruger, G. J.; Reynhardt, E. C. *Acta Crystallogr., Sect. B* **1978**, *34*, 915. $[Co(NH_3)_6]Cl_3$ |
| 32718 | 1.966 Å | Beattie, J. K.; Moore, C. J. *Inorg. Chem.* **1982**, *21*, 1292. $[Co(NH_3)_6][Co(NH_3)_2(NO_2)_4][CoCH_3(NH_3)_2(NO_2)_3]_2$ |
| 35153 | 1.966 Å | Bernal, I.; Korp, J. D.; Schlemper, E. O.; Hussain, M. S. *Polyhedron* **1982**, *1*, 365. $[Co(NH_3)_6][CuCl_5]$ |
| 243806 | 1.966 Å | Poineau, F.; Mausolf, E.; Kerlin, W.; Czerwinski, K. *J. Radioanal. Nucl. Chem.* **2017**, *311*, 775. $[Co(NH_3)_6](TcO_4)_3\cdot 2H_2O$ |
| 415187 | 1.966 Å | Sharma, R. P.; Bala, R.; Sharma, R.; Rychlewska, U.; Warzajtis, B. *J. Fluor. Chem.* **2005**, *126*, 967. $[Co(NH_3)_6]Cl_2[SiF_6]\cdot 2H_2O$ |
| 417161 | 1.966 Å | Sharma, R. P.; Bala, R.; Pretto, L.; Ferretti, V.; Du Y.; Yu J. H. *J. Mol. Struct.* **2007**, *842*, 6. $[Co(NH_3)_6]Br(S_2O_3)\cdot H_2O$ |
| ASAWUN | 1.966 Å | Stein, I.; Ruschewitz, U. *Z. Naturforsch., Teil B* **2011**, *66*, 471. $[Co(NH_3)_6](HOOCCCCOO)Cl_2\cdot 2H_2O$ |
| CAXQEA | 1.966 Å | Harrowfield, J. M. Ling, I.; Skelton, B. W.; Sobolev, A. N.; White, A. H. *Aust. J. Chem.* **2017**, *70*, 485. $[Co(NH_3)_6][Cu(C_7H_3NO_4)_3]\cdot 8.5H_2O$ |
| EGUQAC | 1.966 Å | Lu, X.; Liu, C.; Xiao, X.; Nguyen, T. S.; Xiang, Z.; Chen, C.; Cui, S.;Yavuz, C. T.; Xu, Q.; Liu, B. *ACS Appl. Mater. Interfaces* **2023**, *15*, 54458. $[Co(NH_3)_6](HSO_4)_2]\cdot CO_2$ |
| NALBAE | 1.966 Å | Brayshaw, P. A.; Hall, A. K.; Harrison, W. T. A.; Harrowfield, J. M.; Pearce, D.; Shand, T. M.; Skelton, B. W.; Whitaker, C. R.; White, A. H. *Eur. J. Inorg. Chem.* **2005**, 1127. $[Co(NH_3)_6][La(C_7H_3NO_4)_3]\cdot 5H_2O$ |
| NEKJAS01 | 1.966 Å | Zhang, B.; Weberg, A. B.; Ahn, A. J.; Guron, M.; Jones, L. O.; Gau, M. R.; Schatz, G. C.; Schelter, E. J. *Chem. (Cell Press)* **2025**, *11*, 102361. $[Co(NH_3)_6]CO_3Cl$ |
| NEYBUQ | 1.966 Å | Gorska, N.; Inaba, A.; Hirao, Y.; Mikuli, E. *J. Coord. Chem.* **2013**, *66*, 1238. $[Co(NH_3)_6](BF_4)_3$ |

| | | |
|---|---|---|
| WIRLAM | 1.966 Å | Pascu, G.; Deville, C.; Clifford, S. E.; Guenee, L.; Besnard, C.; Kramer, K. W.; Liu, S.-X.; Decurtins, S.; Tuna, F.; McInnes, E. J. L.; Winpenny, R. E. P.; Williams, A. F. *Dalton Trans.* **2014**, *43*, 656. $[Co(NH_3)_6][CuCl(H_2O)(C_3H_3O_5)]$ |
| 16067 | 1.967 Å | Meek, D. W.; Ibers, J. A. *Inorg. Chem.* **1970**, *9*, 465. $[Co(NH_3)_6][ZnCl_4]Cl$ |
| 177746 | 1.967 Å | Avisar, S.; Shner, Y.; Abu-Rezi, R.; Popov, I.; Bino, A. *J. Alloys Compds.* **2022**, *891*, 161936. $[Co(NH_3)_6]_2[PdCl_4]_3\cdot 8H_2O$ |
| 414608 | 1.967 Å | Sharma, R. P.; Bala, R.; Sharma, R.; Ferretti, V. *Inorg. Chim. Acta* **2005**, *358*, 3457. $[Co(NH_3)_6]Cl_2(IO_3)\cdot H_2O$ |
| ASAWOH01 | 1.967 Å | Seidel, R. W.; Goddard, R.; Gramm, V.; Ruschewitz, U. *Z. Naturforsch., Teil B* **2014**, *69*, 277. $[Co(NH_3)_6](OOCCCCOO)(HOOCCCCOO)\cdot 2H_2O$ |
| CANYAS | 1.967 Å | Sharma, R. P.; Bala, R.; Sharma, R.; Kariuki, B. M.; Rychlewska, U.; Warzajtis, B. *J. Mol. Struct.* **2005**, *748*, 143. $[Co(NH_3)_6](o\text{-}COOH(COO)C_6H_4)_2Cl\cdot 3H_2O$ |
| DAJWAM | 1.967 Å | Clegg, W. *Acta Crystallogr., Sect. C* **1985**, *41*, 1164. $[Co(NH_3)_6][Fe(O_2C_3H_2O_2)_3]Cl\cdot 3H_2O$ |
| LECKIO | 1.967 Å | Dalrymple, S. A.; Shimizu, G. K. H. *Chem. Commun.* **2006**, 956. $[Co(NH_3)_6](C_9H_{11}SO_3)_2)_2Cl$ |
| PEFRIC | 1.967 Å | Sharma, R. P.; Bala, R.; Sharma, R.; Singh, K. N.; Ferretti, V. *J. Mol. Struct.* **2006**, *784*, 117. $[Co(NH_3)_6]_2(p\text{-}C_6H_4(CH_2SO_3)_2)_3\cdot 4H_2O$ |
| SUTZAJ | 1.967 Å | Fedosseev, A. M.; Grigoriev, M. S.; Budantseva, N. A.; Guillaumont, D.; Le Naour, C.; Simoni, E.; Den Auwer, C.; Moisy, P. *Compt. Rendes Chim.* **2010**, *13*, 839. $[Co(NH_3)_6](Am(C_6H_6NO_6)_2(H_2O)]\cdot 8H_2O$ |
| 2561 | 1.968 Å | Iwata, M.; Saito, Y. *Acta Crystallogr., Sect. B* **1973**, *29*, 822. $[Co(NH_3)_6][Co(CN)_6]$ |
| 66321 | 1.968 Å | Aoyama, T.; Ohba, S.; Saito, Y.; Bernal, I. *Acta Crystallogr., Sect. C* **1992**, *48*, 246. $[Co(NH_3)_6][CuCl_5]$ |
| 161488 | 1.968 Å | Makotchenko, E. V.; Baidina, I. A.; Plyusnin, P. E. *Zh. Strukt. Khim.* **2007**, *48*, 282. $[Co(NH_3)_6][AuBr_4]Br_2$ |
| 262513 | 1.968 Å | Han Y.; Li Y.; Yu J.; Pan Q.; Xu R. *Eur. J. Inorg. Chem.* **2012**, 36. $[Co(NH_3)_6][Zn_8(HPO_4)_8(PO_4)_2]PO_4$ |
| 380345 | 1.968 Å | Sharma, R. P.; Sharma, R.; Singh, A.; Saini, A.; Gubanov, A. I.; Smolentsev, A. I.; Venugopalan, P. *J. Mol. Struct.* **2010**, *980*, 261. $[Co(NH_3)_6]_2[Cd_3Br_{10}(H_2O)_2]Br_2\cdot 2H_2O$ |
| 391436 | 1.968 Å | Bala, R.; Sharma, R. P.; Sharma, U.; Burrows, A. D.;Cassar, K. *J. Mol. Struc.* **2007**, *832*, 156. $[Co(NH_3)_6]Cl_2(HC_2O_4)\cdot H_2O$ |
| 413763 | 1.968 Å | Sharma, R. P.; Bala, R.; Sharma, R.; Venugopalan, P. *J. Coord. Chem.* **2004**, *57*, 1563. $[Co(NH_3)_6]Cl(MoO_4)\cdot 3H_2O$ |
| BEFWAO | 1.968 Å | Tamain, C.; Autillo, M.; Guillaumont, D.; Guerin, L.; Wilson, R. E.; Berthon, C. *Inorg. Chem.* **2022**, *61*, 12337. $[Co(NH_3)_6]_2[Th(C_2O_4)_5]\cdot 4H_2O$ |
| EGABAR | 1.968 Å | Qin-He Pan, Q.-H.; Tian, R.-J.; Liu, S.-J.; Wu, Q.-H.; Zhu, Y.-Y.; Chen,Q.; Ren, X.-Y.; Hu, T.-L. *Chin. Chem. Lett.* **2013**, *24*, 861. $[Co(NH_3)_6][Cd_4C_{12}O_{24}]\cdot 4H_2O$ |

| | | |
|---|---|---|
| NALBOS | 1.968 Å | Brayshaw, P. A.; Hall, A. K.; Harrison, W. T. A.; Harrowfield, J. M.; Pearce, D.; Shand, T. M.; Skelton, B. W.; Whitaker, C. R.; White, A. H. *Eur. J. Inorg. Chem.* **2005**, 1127. $[Co(NH_3)_6][Pr(C_7H_3NO_4)_3]\cdot 10H_2O$ |
| NEYHOP | 1.968 Å | Bala, R.; Sharma, R. P.; Sharma, U.; Burrows, A. D.; Cassar, K. *J. Mol. Struct.* **2007**, *832*, 156. $[Co(NH_3)_6](HC_2O_4)Cl_2\cdot H_2O$ |
| VUCCON | 1.968 Å | Charushnikova, L. A.; Krot, N. N.; Makarenkov, V. I.; Starikova, Z. A. *Radiochim.* **2014**, *56*, 364. $[Co(NH_3)_6][La_3C_{18}H_{12}O_{24}]\cdot 6H_2O$ |
| VUCDEE | 1.968 Å | Charushnikova, L. A.; Krot, N. N.; Makarenkov, V. I.; Starikova, Z. A. *Radiochim.* **2014**, *56*, 364. $[Co(NH_3)_6][Pr_3C_{18}H_{12}O_{24}]\cdot 6H_2O$ |
| WEXRIB | 1.968 Å | Bala, R.; Sharma, R. P.; Bond, A. D. *J. Mol. Struct.* **2007**, *830*, 198. $[Co(NH_3)_6](CH_3SO_3)_3$ |
| WEXTID | 1.968 Å | Bala, R.; Sharma, R. P.; Venugopalan, P.; Harrison, W. T.A. *J. Mol. Struct.* **2007**, *830*, 8. $[Co(NH_3)_6](HO(OOC)CHCH(COO)OH)Cl\cdot H_2O$ |
| 117 | 1.969 Å | Haupt, H. J.; Huber, F.; Preut, H. *Z. Anorg. Allg. Chem.* **1976**, *422*, 97. $[Co(NH_3)_6][SnCl_4]Cl$ |
| 137751 | 1.969 Å | Filatov, Evgeny; Lagunova, Varvara; Kochetygov, Ilia; Plyusnin, Pavel; Kuratieva, Natalia; Kostin, Gennadiy; Korenev, S. *Acta Crystallogr., Sect. B* **2022**, *78*, 537. $[Co(NH_3)_6][Co(C_2O_4)_3]\cdot 3H_2O$ |
| 420661 | 1.969 Å | Sharma, R. P.; Sharma, R.; Kumar, A.; Venugopalan, P.; Brando, P.; Felix, V. *Inorg. Chem. Commun.* **2009**, *12*, 945. $[Co(NH_3)_6][HgBr_5]$ |
| BAKMAF | 1.969 Å | Li, X.; Lu, J.; Mu, W.; Chen, B.; Luo, D.; Liu, B.; Yang, Y.; Wei, H.; Peng, S. *Inorg. Chim. Acta* **2022**, *530*, 120675. $[Co(NH_3)_6][U_5C_{54}H_{84}N_6O_{51}]$ |
| NALBEI01 | 1.969 Å | Harrowfield, J. M.; Ling, I.; Skelton, B. W.; Sobolev, A. N.; White, A. H. *Aust. J. Chem.* **2017**, 70, 485. $[Co(NH_3)_6][Ce(C_7H_3NO_4)_3]\cdot 5H_2O$ |
| NALBIM01 | 1.969 Å | Harrowfield, J. M.; Ling, I.; Skelton, B. W.; Sobolev, A. N.; White, A. H. *Aust. J. Chem.* **2017**, 70, 485. $[Co(NH_3)_6][Pr(C_7H_3NO_4)_3]\cdot 5H_2O$ |
| QECTEB | 1.969 Å | Filatov, E.; Lagunova, V.; Kochetygov, I.; Plyusnin, P.; Kuratieva, N.; Kostin, G.; Korenev, S. *Acta Crystallogr., Sect. E* **2022**, *78*, 537. $[Co(NH_3)_6][Co(C_2O_4)_3]\cdot 3H_2O$ |
| VUCCUT | 1.969 Å | Charushnikova, L. A.; Krot, N. N.; Makarenkov, V. I.; Starikova, Z. A. *Radiochim.* **2014**, *56*, 364. $[Co(NH_3)_6][Ce_3C_{18}H_{12}O_{24}]\cdot 6H_2O$ |
| 380346 | 1.970 Å | Sharma, R. P.; Singh, A.; Venugopalan, P.; Smolentsev, A. I.; Gubanov, A. I. *J. Mol. Struct.* **2010**, *975*, 1. $[Co(NH_3)_6]_2[HgI_4]I\cdot H_2O$ |
| 421507 | 1.970 Å | Domonov, D. P.; Kuratieva, N. V.; Pechenyuk, S. I. *J. Struct. Chem.* **2011**, *52*, 358. $[Co(NH_3)_6][Fe(CN)_6]$ |
| CAXYAE | 1.970 Å | Harrowfield, J. M. Ling, I.; Skelton, B. W.; Sobolev, A. N.; White, A. H. *Aust. J. Chem.* **2017**, *70*, 485. $[Co(NH_3)_6][Ho(C_7H_3NO_4)_3]\cdot 10H_2O$ |

| | | |
|---|---|---|
| KUTCIL | 1.970 Å | Grigor'ev, M. S.; Baturin, N. A.; Regel', L. L.; Krot, N. N. *Radiokhimiya* **1991**, *33*, 19. $[Co(NH_3)_6]_2[Np_2O_4C_8O_{16}]\cdot 6H_2O$ |
| 67551 | 1.971 Å | Wen, H.; Miller, S. E.; House, D. A.; McKee, V.; Robinson, W. T. *Inorg. Chim. Acta* **1992**, *193*, 77. $[Co(NH_3)_6]$ $[HgCl_3)Cl(HgCl_2)Cl(HgCl_2)]\cdot H_2O$ |
| 411774 | 1.971 Å | Boudin, S.; Chardon, J.; Daturi, M.; Raveau, B. *J. Solid State Chem.* **2001**, *159*, 239. $[Co(NH_3)_6][(V_{1.5}P_{0.5}O_6(OH)]$ |
| 412059 | 1.971 Å | Dahm, M.; Adam, A. *Z. Naturforsch., Teil B* **2001**, *56*, 1117-1122. $[Co(NH_3)_6]ClCO_3$ |
| CAXXOR | 1.971 Å | Harrowfield, J. M. Ling, I.; Skelton, B. W.; Sobolev, A. N.; White, A. H. *Aust. J. Chem.* **2017**, *70*, 485. $[Co(NH_3)_6][Gd(C_7H_3NO_4)_3]\cdot 10H_2O$ |
| HOSFUS | 1.971 Å | Pook, N.-P.; Adam, A. *Z. Anorg. Allg. Chem.* **2014**, *640*, 2931. $[Co(NH_3)_6]_4[Ce_2C_6O_{22}](CO_3)_2\cdot 12H_2O$ |
| NANCUB | 1.971 Å | Charushnikova, I. A.; Krot, N. N.; Polyakova, I. N. *Radiokhimiya* **2004**, *46*, 318. $[Co(NH_3)_6][(NpO_2)_2C_6H_4O_8]NO_3\cdot 5H_2O$ |
| 10190 | 1.972 Å | Burns, J. H.; Baldwin, W. H.; Stokely, J. R. *Inorg. Chem.* **1973**, *12*, 466. $Li[Co(NH_3)_6][Np_2O_8](OH)_2\cdot 2H_2O$ |
| 39640 | 1.972 Å | Rakov, I. E.; Gorbunova, Y. E.; Mikhailov, Y. N.; Kokunov, Y. V. *Dokl. Akad. Nauk SSSR* **1992**, *322*, 906. $K[Co(NH_3)_6][SnF_3]_2(NO_3)_2\cdot 0.5H_2O$ |
| 66322 | 1.972 Å | Aoyama, T.; Ohba, S. Saito, Y.; Bernal, I. *Acta Crystallogr., Sect. C* **1992**, *48*, 246. $[Co(NH_3)_6][CuCl_5]$ |
| 260055 WUYVET | 1.972 Å | Visser, H. G.; Purcell, W. *Acta Crystallogr., Sect. E* **2008**, *64*, i76. $[Co(NH_3)_6][Mn(CN)_6]$ |
| CAFWEM | 1.972 Å | Zhang, Y.; Collison, D.; Livens, F. R.; Helliwell, M.; Heatley, F.; Powell, A. K.; Wocadlo, S.; Eccles, H. *Polyhedron* **2002**, *21*, 81. $[Co(NH_3)_6]_2[UC_{18}H_{20}O_{20}]Cl_2\cdot 6H_2O$ |
| RORNER | 1.973 Å | Klufers, P.; Mayer, P. *Z. Anorg. Allg. Chem.* **1997**, *623*, 1496. $[Co(NH_3)_6](SbC_{20}H_{20}N_{10}O_{10})\cdot 9H_2O$ |
| 60839 | 1.974 Å | Schulz, F.; Jansen, M. *Z. Anorg. Allg. Chem.* **1986**, *543*, 152. $[Co(NH_3)_6]_2(P_4O_{13})\cdot 5H_2O$ |
| AHIKUX | 1.974 Å | Baldwin, S. M.; Kastner, M. E. *Acta Crystallogr., Sect. XAT* |
| EDTACO | 1.974 Å | Schlemper, E. O. *J. Cryst. Mol. Struct.* **1977**, *7*, 81. $Na[Co(NH_3)_6]((OOCCH_2)_2NCH_2N(CH_2COO)_2\cdot 3.5\ H_2O$ |
| FECGAX | 1.974 Å | Gorska, N.; Inaba, A.; Hirao, Y.; Mikuli, E.; Holderna-Natkaniec, K. *RSC Adv.* **2012**, *2*, 4283. $[Co(NH_3)_6](ClO_4)_3$ |
| 23144 | 1.975 Å | Atoji, M.; Watanabe, T. *J. Chem. Phys.* **1952**, *20*, 1045. $[Co(NH_3)_6][BiCl_6]$ |
| MANGUF | 1.976 Å | Han, Y.; Li, Y.; Yu, J.; Pan, Q.; Xu, R. *Eur. J. Inorg. Chem.* **2012**, 36. $[Co(NH_3)_6]_3[Zn_8H_8O_{40}P_{10}]PO_4$ |
| MEHTEC | 1.976 Å | Darii, M.; Beleaev, E. S.; Kravtsov, V. C.; Bourosh, P.; Chumakov, Y.; Hauser, J.; Decurtins, S.; Liu, S.-X.; Sultanova, O.; Baca, S. G. *New J. Chem.* **2022**, *46*, 11404. $[Co(NH_3)_6][Co(H_2O)_4(NC_5H_3(COO)_2)_2]Cl\cdot 3H_2O$ |
| 805 | 1.977 Å | Iwata, M. *Acta Crystallogr., Sect. B* **1977**, *33*, 59. $[Co(NH_3)_6][Cr(CN)_6]$ |

| | | |
|---|---|---|
| 251045 | 1.977 Å | Clark, D. L.; Conradson, S. D.; Donohoe, R. J.; Gordon, P. L.; Keogh, D. W.; Palmer, P. D.; Scott, B. L.; Tait, C. D. Inporg. Chem. 2013, 52, 3547. $[Co(NH_3)_6][NpO_2(OH)_4]_3·H_2O$ |
| LECKOU | 1.977 Å | Dalrymple, S. A.; Shimizu, G. K. H. *Chem.Commun.* **2006**, 956. $[Co(NH_3)_6](\mathit{1,4}\text{-}C_6H_4(CH_2SO_3)_2)·C_6H_5NH_2·6H_2O$ |
| 39549 | 1.980 Å | Grigor'ev, M. S.; Fedoseev, A. M.; Budantseva, N. A.; Yanovskii, A. I.; Struchkov, Y. T.; Krot, N. N. *Radiokhimiya* **1991**, *33*, 54. $[Co(NH_3)_6][NpO_2SO_4)_2]·2H_2O$ |
| NALBAE01 | 1.980 Å | Harrowfield, J. M.; Ling, I.; Skelton, B. W.; Sobolev, A. N.; White, A. H. *Aust. J. Chem.* **2017**, 70, 485. $[Co(NH_3)_6][La(C_7H_3NO_4)_3]·5H_2O$ |
| VUCDAA | 1.980 Å | Charushnikova, L. A.; Krot, N. N.; Makarenkov, V. I.; Starikova, Z. A. *Radiochim.* **2014**, *56*, 364. $[Co(NH_3)_6][Nd_3C_{18}H_{12}O_{24}]·6H_2O$ |
| 65765 | 1.981 Å | du Bois, A.; Abriel, W. *Acta Crystallogr., Sect. C* **1989**, *45*, 1986. $[Co(NH_3)_6][TeCl_6]Cl·1.64H_2O$ |
| 37075 | 1.982 Å | Tebbe, K. F. *Acta Crystallogr., Sect. C* **1983**, *39*, 154. $[Co(NH_3)_6]I_3·2I_2$ |
| 10183 | 1.985 Å | Schroeder, D. R.; Jacobson, R. A. *Inorg. Chem.* **1973**, *12*, 515. $[Co(NH_3)_6][Sb_2F_9]$ |
| 10178 | 1.989 Å | Schroeder, D. R.; Jacobson, R. A. *Inorg. Chem.* **1973**, *12*, 210. $[Co(NH_3)_6][SbCl_6]$ |
| 1920 | 2.011 Å | Murray-Rust, P. *Acta Crystallogr., Sect. B* **1975**, *31*, 978. $[Co(NH_3)_6][CuCl_5]$ |
| 31568 | 2.018 Å | Watanabe, T.; Atoji, M.; Okazaki, C. *Acta Crystallogr.* **1950**, *3*, 405. $[Co(NH_3)_6][TlBr_6]$ |
| 31567 | 2.046 Å | Watanabe, T.; Atoji, M.; Okazaki, C. *Acta Crystallogr.* **1950**, *3*, 405. $[Co(NH_3)_6][TlCl_6]$ |
| 56127 | 2.166 Å | Wyckoff, R. W. G.; Hendriks, S. B.; McCutcheon, T. P. *Am. J. Sci., Serie 5* **1927**, *13*, 388. $[Co(NH_3)_6](ClO_4)_3$ |
| 35539 | 2.176 Å | Wyckoff, R. W. G. *Am. J. Sci., Ser. 5* **1927**, *13*, 223. $[Co(NH_3)_6]I_3$ |
| BANXOD | 2.234 Å | Birker, P. J. M. W. L..; Reedijk, J.; Verschoor, G. C. *Inorg. Chem.* **1981**, *20*, 2877. $[Co(NH_3)_6]_5[Ag_8C_{60}H_{108}ClN_{12}O_{24}S_{12}]_3·197H_2O$ |
| **Mean** | **1.964 Å/228 structures** | |

**Rhodium(III)**

*Six-coordination, octahedral configuration*

| ICSD/CSD | *d*(Rh-N) | Reference and formula |
|---|---|---|
| 187075 | 2.043 Å | Khranenko, S. P.; Bykova, E. A.; Alexeyev, A. V.; Tyutyunnik, A. P.; Gromilov, S. A. *J. Solid State Chem.* **2012**, *53*, 521. $[Rh(NH_3)_6](ReO_4)_3·2H_2O$ |
| 187073 | 2.061 Å | Khranenko, S. P.; Bykova, E. A.; Alexeyev, A. V.; Tyutyunnik, A. P.; Gromilov, S. A. *J. Solid State Chem.* **2012**, *53*, 521. $[Rh(NH_3)_6]Cl_3$ |
| 5826 | 2.062 Å | Bykov, Maxim; Yusenko, Kirill V.; Bykova, Elena; Pakhomova, Anna; Kraus, Werner; Dubrovinskaia, Natalia; Dubrovinsky, L. *Eur. J. Inorg. Chem.* **2019**, 3667. $[Rh(NH_3)_6]Cl_4(N_3)_5$ |

| | | |
|---|---|---|
| 187074 | 2.071 Å | Khranenko, S. P.; Bykova, E. A.; Alexeyev, A. V.; Tyutyunnik, A. P.; Gromilov, S. A. *J. Solid State Chem.* **2012**, *53*, 521. $[Rh(NH_3)_6](MoO_4)Cl\cdot 3H_2O$ |
| 23398 | 2.071 Å | Kimura, T.; Sakurai, T. *J. Solid State. Chem.* **1980**, *34*, 369. $[Rh(NH_3)_6]_3[Rh(SnCl_3)_4(SnCl_4)][SnCl_6]\cdot 4H_2O$ |
| 109998 XEDNEZ | 2.073 Å | Gorol, M.; Mosch-Zanetti, N. C.; Noltemeyer, M.; Roesky, H. W. *Z. Anorg. Allg. Chem.* **2019**, *60*, 1062. $[Rh(NH_3)_6][Fe(C_2O_4)_3]\cdot 4H_2O$ |
| **Mean** | **2.064 Å/6 structures** | |

**Iridium(III)**

*Six-coordination, octahedral configuration*

| ICSD/CSD | *d*(Ir-N) | Reference and formula |
|---|---|---|
| 424948 | 2.010 Å | Khranenko; S. P.; Bykova; E. A.; Yusenko; K. V.; Tyutyunnik; A. P.; Gromilov, S. A. *J. Struct. Chem.* **2013**, *54*, 931. $[Ir(NH_3)_6](ReO_4)_3\cdot 2H_2O$ |
| 123881 | 2.060 Å | Vasilchenko, D.; Berdyugin, S.; Komarov, V.; Sheven, D.; Kolesov, B.; Filatov, E.; Tkachev, S. *Inorg.Chem* **2022**, *61*, 5926. $[Rh(NH_3)_6][Pt(OH)_5Cl]Cl\cdot H_2O$ |
| 123879 | 2.067 Å | Vasilchenko, D.; Berdyugin, S.; Komarov, V.; Sheven, D.; Kolesov, B.; Filatov, E.; Tkachev, S. *Inorg.Chem* **2022**, *61*, 5926. $[Rh(NH_3)_6][Pt(OH)_5Cl]_{0.75}[Pt(OH)_4Cl_2]_{0.25}\cdot 1.5H_2O$ |
| QRCTIF | 2.078 Å | Filatov, E.; Lagunova, V.; Kochetygov, I.; Plyusnin, P.; Kuratieva, N.; Kostin, G.; Korenev, S. *Acta Crystallogr., Sect. B* **2022**, *78*, 537. $[Ir(NH_3)_6][Ir(C_2O_4)_3]$ |
| 419315 | 2.082 Å | Filatov, E. Y.; Yusenko, K. V.; Vikulova, E. S.; Plyusnin, P. E.; Shubin, Y. V. *Z. Kristallogr. Suppl. Issue* **2009**, *30*, 263. $[Ir(NH_3)_6][Co(C_2O_4)_3]\cdot H_2O$ |
| 109999 XEDNID | 2.088 Å | Gorol, M.; Mosch-Zanetti, N. C.; Noltemeyer, M.; Roesky, H. W. *Z. Anorg. Allg. Chem.* **2019**, *60*, 1062. $[Ir(NH_3)_6][Fe(C_2O_4)_3]\cdot 4H_2O$ |
| QOWHOC | 2.091 Å | Yusenko, K. V.; Pechenyuk, S. I.; Vikulova, E. S.; Semushina, Y. P.; Baidina, I. A.; Filatov, E. Y. *Zh. Strukt. Khim.* **2019**, *60*, 1062. $[Ir(NH_3)_6][Fe(C_2O_4)_3]\cdot 3H_2O$ |
| BIWHEY | 2.094 Å | Varygin, A. D.; Popov, A. A.; Gromilov, S. A.; Plyusnin, P. E.; Korenev, S. V. *J. Struct. Chem.* **2023**, *64*, 1261. $[Ir(NH_3)_6][Fe(CN)_6]$ |
| **Mean** | **2.080 Å/7 structures** | |

**Nickel(II)**

*Six-coordination, octahedral configuration*

| ICSD/CSD | *d*(Ni-N) | Reference and formula |
|---|---|---|
| 429169 | 2.055 Å | Breternitz, J.; Farrugia, L. J.; Godula-Jopek, A.; Saremi-Yarahmadi, S.; Malka, I. E.; Hoang, T. K. A.; Gregory, D. H. *J. Cryst. Growth* **2015**, *412*, 1. $[Ni(NH_3)_6](NO_3)_2$ |

| | | |
|---|---|---|
| RAFLIX | 2.082 Å | Nefedov, S. E.; Eremenko, I. L. *Zh. Neorg. Khim.* **2021**, *66*, 1380. $[Ni(NH_3)_6](OOC(CH_3)_3)_2$ |
| 433371 | 2.089 Å | Daigre, G.; Costuas, K.; Tarasenko, M. S.; Ledneva, A. Y.; Naumov, N. G.; Lemoine, P.; Guizouarn, T.; Molard, Y.; Amela-Cortes, M.; Audebrand, N.; Cordier, S. *Dalton Trans.* **2018**, *47*, 1122. $[Ni(NH_3)_6)_4][Ni_2(NH_3)_8][Mo_6Br_6S_2(CN)_6]_3\cdot 12H_2O$ |
| 433370 | 2.112 Å | Daigre, G.; Costuas, K.; Tarasenko, M. S.; Ledneva, A. Y.; Naumov, N. G.; Lemoine, P.; Guizouarn, T.; Molard, Y.; Amela-Cortes, M.; Audebrand, N.; Cordier, S. *Dalton Trans.* **2018**, *47*, 1122. $[Ni(NH_3)_6)_4](C_2(NO_2)_4)$ |
| MOXPUO | 2.119 Å | Dong, W.-S.; Mei, H.-Z.; Yu, Q.-Y.; Xu, M.-Q.; Li, Z.-Y.; Zhang, J.-G. *Dalton Trans.* **2024**, *53*, 13925. $[Ni(NH_3)_6)_4][Ni_2(NH_3)_8][Mo_6Br_6Se_2(CN)_6]_3\cdot 12H_2O$ |
| 78865 | 2.120 Å | Essmann, R.; Kreiner, G.; Niemann, A.; Rechenbach, D.; Schmieding, A.; Sichla, T.; Zachwieja, U.; Jacobs, H. *Z. Anorg. Allg. Chem.* **1996**, *622*, 1161. $[Ni(NH_3)_6]Cl_2$ |
| 424257 | 2.123 Å | Gaifulin, Y. M.; Smolentsev, A. I.; Mironov, Y. V. *J. Struct. Chem.* **2013**, *54*, 459. $(NH_4)_2[Ni(NH_3)_6]_5[(Re_{12}CS_{17}(CN)_6]_2\cdot 17H_2O$ |
| TOQPEJ | 2.123 Å | Szimhardt, N.; Bolter, M. F.; Born, M.; Klapotke, T. M.; Stierstorfer, J. *Dalton Trans.* **2017**, *46*, 5033. $[Ni(NH_3)_6](C_2N_{12}O_4)$ |
| 170748 | 2.126 Å | Wagner, M.; Lerner, H.W.; Bolte, M. *Acta Crystallogr., Sect. C* **2000**, *56*, e77. $[Ni(NH_3)_6]Cl_2$ |
| KAKZAX | 2.127 Å | Hummel, H.-U.; Beiler, F. *Z. Anorg. Allg. Chem.* **1988**, *565*, 147. $[Ni(NH_3)_6]((NC)_2CCS(O))\cdot 1.5H_2O$ |
| 410301 | 2.128 Å | Mockenhaupt, C.; Essmann, R.; Lutz, H. D. *Naturforsch., Teil B* **1999**, *54*, 843. $[Ni(NH_3)_6]SO_4$ |
| JIRDOG | 2.128 Å | Gayfulin, Y. M.; Smolentsev, A I.; Mironov, Y. V. *Zh. Strukt. Khim.* **2018**, *59*, 669. $K_2[Ni(NH_3)_6]_5[ReC_7N_6O_4S_{15}]_2\cdot 16H_2O$ |
| FOPJEB | 2.132 Å | Oelkers, B. *Eur. J. Inorg. Chem.* **2014**, 5838. $[Ni(NH_3)_6](C_{12}H_{10}N_2O_6S_2)$ |
| 426457 | 2.136 Å | Ledneva, A. Y.; Smolentsev, A. I.; Naumov, N. G. *J. Coord. Chem.* **2013**, *66*, 4363. $[Ni(NH_3)_6][Re_6S_8(CN)_4(NH_3)_2]\cdot 2H_2O$ |
| BICSIQ | 2.136 Å | Paul, B.; Nather, C.; Walfort, B.; Fromm, K. M.; Zimmermann, B.; Lang, H.; Janiak, C. *CrystEngComm* **2004**, *6*, 293. $[Ni(NH_3)_6]C_{20}H_{12}O_2\cdot 2C_{20}H_{14}O_2$ |
| NUHJUV | 2.139 Å | Himmel, K.; Jansen, M. *Chem. Commun.* **1998**, 1205. $[Ni(NH_3)_8]C_{60}\cdot 6NH_3$ |
| BAWLIW | 2.143 Å | Mironov, Y. V.; Gayfulin, Y. M.; Kozlova, S. G.; Smolentsev, A I.; Tarasenko, M. S.; Nizovtsev, A. S.; Fedorov, V. E. *Inorg. Chem.* **2012**, *51*, 4359. $[Ni(NH_3)_6]_3[Re_{12}C_7H_6S_{17}]\cdot 4H_2O$ |
| 407672 | 2.144 Å | Tebbe, K.F.; Gilles, T.; Radke, B. *Z. Naturforsch., Teil B* **1998**, *53*, 87. $[Ni(NH_3)_6](I_3)_2$ |
| 407673 | 2.148 Å | Tebbe, K.F.; Gilles, T.; Radke, B. *Z. Naturforsch., Teil B* **1998**, *53*, 87. $[Ni(NH_3)_6](I_5)_2$ |
| UCASEY | 2.150 Å | Gayfulin, Y. M.; Smolentsev, A. I.; Mironov, Y. V. *J. Coord. Chem.* **2011**, *64*, 3832. $[Ni(NH_3)_6][Ni(NH_3)_4(NCRe_6C_3N_2S_8)_2]\cdot 8H_2O$ |

| | | |
|---|---|---|
| 83756 XIRNUI | 2.158 Å | Podberezskaya, N. V.; Doronina, V. P.; Bakakin, V. V.; Yakovlev, I. I. *Zh. Strukt. Khim.* **1984**, *25*, 182. $[Ni(NH_3)_6](C(NO_2)_3)_2$ |
| 50466 | 2.164 Å | Schoening, R. A.; Meyer, G. . *Z. Anorg. Allg. Chem.* **1998**, *624*, 1389. $[Ni(NH_3)_6][TaF_6]_2$ |
| 81376 | 2.167 Å | Riou, A.; Cudennec, Y.; Gerault, Y.; Lecerf, A. *Compt. Rend. l'Akademie Sci., Serie IIb* **1996**, *322*, 247. $[Ni(NH_3)_6](NO_3)_2$ |
| **Mean** | **2.132 Å/22 structures** | |

**Palldium(II)**

*Four-coordination, square-planar configuration*

| ICSD/CSD | *d*(Pd-N) | Reference and formula |
|---|---|---|
| 428068 | 1.987 Å | Mulagaleev, R. F.; Soloviev, L. A.; Blokhin, A. I.; Kirik, S. D. *Russ. J. Coord. Chem.* **2003**, *29*, 256. $[Pd(NH_3)_4][Pd(NH_3)_2(SO_3)_2][Pd(NH_3)_3(SO_3)]\cdot H_2O$ |
| 89696 | 2.015 Å | Gromilov, S. A.; Baidina, I. A.; Tatarchuk, V. V.; Virovets, A. V.; Druzhinina, I. A. *Z. Strukt. Khim.* **1999**, *40*, 777. $[Pd(NH_3)_4]_3[Pd(NH_3)_3Cl]_2Cl_8$ |
| 15990 | 2.018 Å | Dickinson, B. N. *Z. Kristallogr.* **1934**, *88*, 281. $[Pd(NH_3)_4]_2Cl_2\cdot H_2O$ |
| 430363 | 2.023 Å | Gubanov, A. I.; Danilenko, A. M.; Smolentsev, A. I.; Kuratieva, N. V.; Venediktov, A. B.; Korenev, S. V. *J. Struct. Chem.* **2016**, *57*, 1606. $[Pd(NH_3)_4]_3[IrF_6]_2Cl_2\cdot H_2O$ |
| ODETAT | 2.026 Å | Hirai, Y.; Igashira-Kamiyama, A.; Kawamoto, T.; Konno, T. *Chem. Lett.* **2007**, *36*, 434. $NH_4[Pd(NH_3)_4]_6[HgBr_3(NH_3)][HgBr_2(S_2C_{15}H_{30}N_4O_6S)]\cdot 30H_2O$ |
| JONTEH | 2.028 Å | Oliveira, W. X. C.; da Costa, M. M.; Pinheiro, C. B.; Fontes, A. P. S.; de Paula, F. C. S.; Jaimes, E. H. L.; de Souza, P. P.; Pereira-Maia, E. C.; Pereira, C. L. M. *Polyhedron* **2014**, *76*, 16. $[Pd(NH_3)_4][PdC_{10}C_4N_2O_6]$ |
| 32615 | 2.031 Å | Tebbe, K.F.; Freckmann, B. *Z. Naturforsch., Teil B* **1982**, *37*, 542. $[Pd(NH_3)_4](I_5)(l_3)$ |
| APDPZC | 2.032 Å | Harlow, R. L.; Simonsen, S. H. *Acta Crystallogr., Sect. B* **1974**, *30*, 1370. $[Pd(NH_3)_4](C_6H_2N_2O_4)$ |
| 169808 | 2.033 Å | Smolentsev, A. I.; Gubanov, A. I.; Zadesenets, A. V.; Plyusnin, P. E.; Baidina, I. A.; Korenev, S. V. *Z. Neorg. Khim.* **2010**, *51*, 736. $[Pd(NH_3)_4](NO_3)_2$ |
| 109471 | 2.034 Å | Solovyov, L. A.; Blochina, M. L.; Kirik, S. D.; Blochin, A. I.; Derikova, M. G. *Powder Diffr.* **1996**, *11*, 13. $[Pd(NH_3)_4]C_2O_4$ |
| ZAKWIR | 2.037 Å | Mealli, C.; Pichierri, F.; Randaccio, L.; Zangrando, E.; Krumm, M.; Holtenrich, D.; Lippert, B. *Inorg. Chem.* **1995**, *34*, 3418. $[Pd(NH_3)_4][PdPtC_{10}H_{21}N_9O_2]_2(NO_3)_6\cdot 6H_2O$ |
| 74028 | 2.038 Å | Laligant, Y. *Eur. J. Solid State Inorg. Chem.* **1993**, *30*, 1017. $[Pd(NH_3)_4]MoO_4$ |
| 425507 | 2.038 Å | Gubanov, A. I.; Filatov, E. Y.; Semitut, E. Y..; Smolentsev, A. I.; Snytnikov, P. V.; Potemkin, D. I.; Korenev, S. V. *Thermochim. Acta* **2013**, *566*, 100. $[Pd(NH_3)_4]MoO_4$ |

| | | |
|---|---|---|
| DEKLIS | 2.038 Å | Gladysheva, M. V.; Plyusnin, P. E.; Komarov, V. Y.; Tsygankova, A. R.; Gerasimov, E. Y.; Shubin, Y. V.; Korenev, S. V. *J. Struct. Chem.* **2022**, *63*, 556. $[Pt(NH_3)_4][Pt(NH_3)_3(NO_2)][Cr(C_2O_4)_3]\cdot 0.8H_2O$ |
| RIFPEF | 2.039 Å | Mikhailov, A.; Konieczny, K. A.; Gladysheva, M.; Plyusnin, P.; Pillet, S.; Schaniel, D. *Inorg. Chem.* **2023**, *62*, 5531. $[Pd(NH_3)_4][Pd(NH_3)_3NO_2][Co(O_2C_2O_2)_3]\cdot 0.72\ H_2O$ |
| 410384 | 2.039 Å | Beck, W.; Klapoetke, T. M.; Knizek, J.; Noeth, H.; Schuett, T. *Eur. J. Inorg. Chem.* **1999**, 523. $[Pd(NH_3)_4][Pd(N_3)_4]$ |
| 72997 | 2.040 Å | Laligant, Y. *Eur. J. Solid State Inorg. Chem.* **1993**, *30*, 681. $[Pd(NH_3)_4]CrO_4$ |
| 248717 | 2.040 Å | Grassl, T.; Korber, N. *Acta Crystallogr., Sect. E* **2014**, *70*, i32. $[Pd(NH_3)_4]Cl_2\cdot 4NH_3$ |
| DEKLEO | 2.040 Å | Gladysheva, M. V.; Plyusnin, P. E.; Komarov, V. Y.; Tsygankova, A. R.; Gerasimov, E. Y.; Shubin, Y. V.; Korenev, S. V. *J. Struct. Chem.* **2022**, *63*, 556. $[Pt(NH_3)_4][Pt(NH_3)_3(NO_2)][Co(C_2O_4)_3]\cdot 0.83H_2O$ |
| TEPZUM | 2.040 Å | Xianqiang Huang, X.; Li, J.; Shen, G.; Xin, N.; Lin, Z.; Chi, Y.; Dou, J.; Li, D.; Hu, C. *Dalton Trans.* **2018**, *47*, 726. $[Pd(NH_3)_4]_3[V_{10}O_{28}]\cdot 8H_2O$ |
| 412607 | 2.041 Å | Ang, H.-G.; Fraenk, W.; Karaghiosoff, K.; Klapoetke, T. M.; Mayer, P.; Noeth, H.; Sprott, J.; Warchhold, M. *Z. Anorg. Allg. Cherm.* **2002**, *628*, 2894. $[Pd(NH_3)_4](N(NO_2)_2)_2$ |
| 417074<br>SOPKOY | 2.041 Å | Yusenko, K. V.; Filatov, E. Y.; Vasilchenko, D. B.; Baidina, I. A.; Zadesenez, A. V.; Shubin, Y. V. *Z. Krist. Cryst. Mater*. **2007**, *222*, 289. $[Pd(NH_3)_4][Cu(H_2O)_2(C_2O_4)_2]$ |
| 417562<br>XIXXOS | 2.041 Å | Zadesenets, A. V.; Filatov, E. Y.; Yusenko, K. V.; Shubin, Y. V.; Korenev, S. V.; Baidina, I. A. *Inorg. Chim. Acta* **2008**, *361*, 199. $[Pd(NH_3)_4][Ni(H_2O)_2(C_2O_4)_2]\cdot 2H_2O$ |
| 421898 | 2.042 Å | Rybinskaya, A. A.; Shusharina, E. A.; Plyusnin, P. E.; Shubin, Y. V.; Korenev, S. V.; Gromilov, S. A. *J. Struct. Chem.* **2011**, *52*, 816. $[Pd(NH_3)_4]_3[Ir(NO_2)_6]_2\cdot H_2O$ |
| 156506<br>KIYVUK | 2.043 Å | Plyusnin, P. E.; Baidina, I. A.; Shubin, Y. V.; Korenev, S. V. *Zh. Neorg. Khim.* **2007**, *52*, 421. $[Pd(NH_3)_4][AuCl_4]_2$ |
| 185538 | 2.043 Å | Shusharina, E. A.; Rybinskaya, A. A.; Plyusnin, P. E.; Shubin, Y. V.; Korenev, S. V.; Gromilov, S. *J. Struct. Chem.* **2011**, *52*, 621. $[Pd(NH_3)_4][Rh(NH_3)(NO_2)_5]$ |
| 420432 | 2.045 Å | Zadesenets, A.; Filatov, E.; Plyusnin, P.; Baidina, I.; Dalezky, V.; Shubin, Y..; Korenev, S.; Bogomyakov, A. *Polyhedron* **2011**, *30*, 1305. $[Pd(NH_3)_4][Co(H_2O)_2(C_2O_4)_2]$ |
| OQIDUO | 2.045 Å | Zadesenets, A.; Filatov, E.; Plyusnin, P.; Baidina, I.; Dalezky, V.; Shubin, Y.; Korenev, S.; Bogomyakov, A. *Polyhedron* **2011**, *30*, 1305. $[Pd(NH_3)_4][Co(H_2O)_2(C_2O_4)_2]$ |
| 169809 | 2.046 Å | Smolentsev, A. I.; Gubanov, A. I.; Zadesenets, A. V.; Plyusnin, P. E.; Baidina, I. A.; Korenev, S. V. *Z. Neorg. Khim.* **2010**, *51*, 736. $[Pd(NH_3)_4]F_2\cdot H_2O$ |
| DEKLOY | 2.046 Å | Gladysheva, M. V.; Plyusnin, P. E.; Komarov, V. Y.; Tsygankova, A. R.; Gerasimov, E. Y.; Shubin, Y. V.; Korenev, S. V. *J. Struct. Chem.* **2022**, *63*, 556. $K_5[Pd(NH_3)_4][Cr(C_2O_4)_3]_2(NO_3)\cdot 6H_2O$ |

| | | |
|---|---|---|
| 417563 XIXXUY | 2.047 Å | Zadesenets, A. V.; Filatov, E. Y.; Yusenko, K. V.; Shubin, Y. V.; Korenev, S. V.; Baidina, I. A. *Inorg. Chim. Acta* **2008**, *361*, 199. $[Pd(NH_3)_4][Zn(H_2O)_2(C_2O_4)_2]\cdot2H_2O$ |
| 430362 | 2.047 Å | Gubanov, A. I.; Danilenko, A. M.; Smolentsev, A. I.; Kuratieva, N. V.; Venediktov, A. B.; Korenev, S. V. *J. Struct. Chem.* **2016**, *57*, 1606. $[Pd(NH_3)_4][IrF_6]\cdot H_2O$ |
| 75383 | 2.052 Å | Laligant, Y. *Eur. J. Solid State Inorg. Chem.* **1994**, *31*, 211. $[Pd(NH_3)_4]Mo_7O_{24}\cdot3NH_3$ |
| 78663 | 2.052 Å | Laligant, Y.; Le Bail, A. *Powder Diffr.* **1995**, *10*, 159. $[Pd(NH_3)_4]Cr_2O_7$ |
| 23 | 2.056 Å | Bell, J. D.; Bowles, J. C.; Cumming, H. J.; Hall, D.; Holland, R. V. *Acta Crystallogr., Sect. B* **1976**, *32*, 634. $[Pd(NH_3)_4]_2Cl_2\cdot H_2O$ |
| **Mean** | **2.039 Å/33 structures** | |

**Platinum(II)**

*Four-coordination, square-planar configuration*

| ICSD/CSD | *d*(Pt-N) | Reference and formula |
|---|---|---|
| FOBLOX | 2.033 Å | Alston, D. R.; Slawin, A. M. Z.; Stoddart, J. F.; Williams, D. J.; Zarzycki, R. *Angew. Chem., Int Ed.* **1987**, *26*, 692. $[Pt(NH_3)_4](PF_6)_2\cdot2C_{50}H_{50}N_2O_{12}\cdot2(CH_3)_2CO$ |
| 49913 | 2.035 Å | Toffoli, P.; Khodadad, P.; Rodier, N. *Acta Crystallogr., Sect. C* **1987**, *43*, 2048. $[Pt(NH_3)_4]_3[PtCl(NH_3)_3]_2Cl_8$ |
| FUTHAF | 2.035 Å | Stender, M.; White-Morris, R. L.; Olmstead, M. M.; Balch, A. L. *Inorg. Chem.* **2003**, *42*, 4505. $[Pt(NH_3)_4][Ag(CN)_2]_2\cdot1.4H_2O$ |
| FICXAQ | 2.036 Å | Alston, D. R.; Slawin, A. M. Z.; Stoddart, J. F.; Williams, D. J.; Zarzycki, R. *Angew. Chem., Int Ed.* **1987**, *26*, 693. $[Pt(NH_3)_4](PF_6)_2\cdot C_{30}H_{42}O_{10}\cdot0.25C_2H_5OH$ |
| KANXON | 2.036 Å | Sadler, P.; Müller, P.; Parsons, S.; Messenger, D. CCDC deposition number 276862, **2005**. $[Pt(NH_3)_4]_2[(NC)_5CNPt(NH_3)_4FeNPt(CN)_5)]\cdot12H_2O$ |
| 156511 | 2.037 Å | Korolkov, I. V.; Gubanov, A. I.; Gromilov, S. A. *Zh. Strukt. Khim.* **2005**, *46*, 492. $[Pt(NH_3)_4][ReCl_6]$ |
| 169807 | 2.037 Å | Smolentsev, A. I.; Gubanov, A. I.; Zadesenets, A. V.; Plyusnin, P. E.; Baidina, I. A.; Korenev, S. V. *Zh. Strukt. Khim.* **2010**, *51*, 736. $[Pt(NH_3)_4](NO_3)_2$ |
| 175609 | 2.037 Å | Lagunova, V.; Filatov, E.; Plyusnin, P.; Kostin, G.; Urlukov, A.; Potemkin, D.; Korenev, S. *Int. J. Hydrogen Energy* **2023**, *48*, 25133. $[Pt(NH_3)_4]WO_4$ |
| 74556 | 2.038 Å | Garnier, E.; El Mouahid, A.; Cernak, J. *Acta Crystallogr., Sect. C* **1994**, *50*, 845. $[Pt(NH_3)_4][SnCl_6]$ |
| 131445 | 2.038 Å | Brieger, L.; Henke, S.; Said Mohamed, A.; Jourdain, I.; Knorr, M.; Strohmann, C. *Inorg. Chim. Acta* **2019**, *495*, 119002. $[Pt(NH_3)_4]Cl_2$ |
| TCQWMPT | 2.038 Å | Endres, H.; Keller, H. J.; Moroni, W.; Nothe, D.; Dong, V. *Acta Crystallogr., Sect. B* **1978**, *34*, 1703. $[Pt(NH_3)_4](C_{12}H_4N_4)_2$ |

| | | |
|---|---|---|
| 1960 | 2.041 Å | Morosin, B.; Fallon, P.; Valentine, J. S. *Acta Crystallogr., Sect. B* **1975**, *31*, 2220. $[Pt(NH_3)_4][CuCl_4]$ |
| XOMVEC | 2.041 Å | Jinzhen, L.; Abrahams, B. F.;Winther-Jensen, B.; Martin, L. L.; Bond, A. M. *ChemCatChem* **2014**, *6*, 2345. $[Pt(NH_3)_4](C_{12}F_4N_4)_2{\cdot}(C_2H_5)_2O{\cdot}1.33\ CH_3OH$ |
| 75276 | 2.042 Å | Garnier, E.; Bele, M. *Acta Crystallogr., Sect. C* **1994**, *50*, 994. $[Pt(NH_3)_4][IrCl_5(H_2O)]{\cdot}2H_2O$ |
| 175610 | 2.043 Å | Lagunova, V.; Filatov, E.; Plyusnin, P.; Kostin, G.; Urlukov, A.; Potemkin, D.; Korenev, S. Int. J. Hydrogen Energy 2023, 48, 25133. $[Pt(NH_3)_4]MoO_4$ |
| 417564 | 2.043 Å | Zadesenets, A. V.; Filatov, E. Y.; Yusenko, K. V.; Shubin, Y. V.; Korenev, S. V.; Baidina, I. A. *Inorg. Chim. Acta* **2008**, *361*, 199. $[Pt(NH_3)_4][Zn(H_2O)_2(C_2O_4)_2]{\cdot}2H_2O$ |
| SEKVIQ | 2.043 Å | Lu, J.; Nafady, A.; Abrahams, B. F.; Abdulhamid, M.; Winther-Jensen, B.; Bond, A. M.; Martin. L. L. *Aust. J. Chem.* **2017**, *70*, 997. $[Pt(NH_3)_4](C_{12}H_4N_4)_2{\cdot}2(CH_3)_2NCHO$ |
| XIXXIM | 2.043 Å | Zadesenets, A. V.; Filatov, E. Y.; Yusenko, K. V.; Shubin, Y. V.; Korenev, S. V.; Baidina, I. A. *Inorg. Chim. Acta* **2008**, *361*, 199. $[Pt(NH_3)_4][Zn(H_2O)_2(C_2O_4)_2]{\cdot}2H_2O$ |
| 162189 | 2.046 Å | Korol'kov, I. V.; Gubanov, A. I.; Yusenko, K. V.; Baidina, I. A.; Gromilov, S. A. *Zh. Strukt. Khim.* **2007**, *48*, 530. $[Pt(NH_3)_4][OsCl_6]$ |
| 417564 | 2.046 Å | Zadesenets, A. V.; Filatov, E. Y.; Yusenko, K. V.; Shubin, Y. V.; Korenev, S. V.; Baidina, I. A. *Inorg. Chim.* |
| XIXXEI | 2.046 Å | *Acta* **2008**, *361*, 199. $[Pt(NH_3)_4][Ni(H_2O)_2(C_2O_4)_2]{\cdot}2H_2O$ |
| 158875 | 2.047 Å | Korolkov, I. V.; Zadesenets, A. V.; Gromilov, S. A.; Yusenko, K. V.; Baldina, I. A.; Korenev, S. V. *Zh. Strukt. Khim.* **2006**, *47*, 503. $[Pt(NH_3)_4](ReO_4)_2$ |
| 158876 | 2.047 Å | Korolkov, I. V.; Zadesenets, A. V.; Gromilov, S. A.; Yusenko, K. V.; Baldina, I. A.; Korenev, S. V. *Zh. Strukt. Khim.* **2006**, *47*, 503. $[Pt(NH_3)_4](ReO_4)_2$ |
| 413829 | 2.047 Å | Casas, J. S.; Parajo, Y.; Romero, Y.; Sanchez-Gonzalez, A.; Sordo, J.; Vazquez-Lopez, E. M. *Z. Anorg. Allg. Chem.* **2004**, *630*, 980. $[Pt(NH_3)_4][PtI_4]$ |
| XIXXOS | 2.047 Å | Zadesenets, A. V.; Filatov, E. Y.; Yusenko, K. V.; Shubin, Y. V.; Korenev, S. V.; Baidin, I. A. *Inorg. Chim. Acta* **2008**, *361*, 199. $[Pt(NH_3)_4][Ni(H_2O)_2(C_2O_4)_2]{\cdot}2H_2O$ |
| HOTVET | 2.047 Å | Avisar-Levy, M.; Levy, O.; Ascarelli, O.; Popov, I.; Bino, A. *J. Alloys Compd.* **2015**, *635*, 48. $[Pt(NH_3)_4][CoC_{10}H_{12}N_2O_8]_2{\cdot}2H_2O$ |
| VAFDOV | 2.047 Å | Khodadad, N.; Rodier, N. *Acta Crystallogr., Sect. B* **1989**, *45*, 208. $[Pt(NH_3)_4](CH_3SO_3)_2$ |
| 20464 | 2.048 Å | Mironov, Y. I.; Bakakin, V. V.; Zemskov, S. V.; Neronova, N. N. *Zh. Strukt. Khim.* **1968**, *9*, 707. $[Pt(NH_3)_4]SO_4$ |
| 417075 | 2.048 Å | Yusenko, K. V.; Filatov, E. Y.; Vasil'chenko, D. B.; Baidina, I. A.; Zadesenetz, A. V.; Shubin, Y. V. *Z. Kristallogr.* **2007**, *26*, 289. $[Pt(NH_3)_4][Cu(C_2O_4)_2]{\cdot}3H_2O$ |

| | | |
|---|---|---|
| FICXEU | 2.049 Å | Alston, D. R.; Slawin, A. M. Z.; Stoddart, J. F.; Williams, D. J.; Zarzycki, R. *Angew. Chem., Int Ed.* **1987**, *26*, 693. $[Pt(NH_3)_4](PF_6)_2\cdot 2C_{34}H_{50}O_{10}$ |
| ROXQUU | 2.049 Å | Vorobyeva, S. N.; Rudzis, Z. V.; Sukhikh, T. S.; Filatov, E. Y.; Plusnin, P. E.; Nadolinny,V. A.; Bogomyakov, A. S.; Korenev, S. V. New. J. Chem. 2024, 48, 15894. $[Pt(NH3)_4][VO(H_2O)(O_2C_2O_2)_2]\cdot 2H_2O$ |
| 248716 | 2.050 Å | Grassl, T.; Korber, N. *Acta Crystallogr., Sect. E* **2014**, *70*, i31. $[Pt(NH_3)_4]Cl_2]\cdot 4NH_3$ |
| 26224 | 2.052 Å | Shandles, R.; Schlemper, E. O.; Murmann, R. K. *Inorg. Chem.* **1971**, *10*, 2785. $[Pt(NH_3)_4][O(Re(CN)_4)_2]$ |
| 60668 | 2.052 Å | Tanaka, M.; Tsujikawa, I.; Toriumi, K.; Ito, T. *Acta Crystallogr., Sect. C* **1986**, *42*, 1105. $[Pt(NH_3)_4][PtI_2(NH_3)_4](HSO_4)_4\cdot 2H_2O$ |
| 420437 OQIFAW | 2.052 Å | Zadesenets, A.; Filatov, E.; Plyusnin, P.; Baidina, I.; Dalezky, V.; Shubin, Y..; Korenev, S.; Bogomyakov, A. *Polyhedron* **2011**, *30*, 1305. $[Pt(NH_3)_4][Co(H_2O)_2(C_2O_4)_2]\cdot 2H_2O$ |
| APTCPT | 2.052 Å | Rochon, F. D.; Melanson, R. *Acta Crystallogr., Sect. B* **1980**, *36*, 691. $[Pt(NH_3)_4][PtCl_3(\mathit{2,6}\text{-}NC_5H_3(CH_3)_2)]\cdot 2H_2O$ |
| FORJOL | 2.052 Å | Khodadad, P.; Rodier, N. *Acta Crystallogr., Sect. C* **1987**, *43*, 1690. $[Pt(NH_3)_4][PtCl_3(SO_3)]_2$ |
| UFEJOG | 2.052 Å | Parsons, S.; Zhu, M.; Sadler, P.; Wood, P. CCDC deposition number 247846, **2004**. $[Pt(NH_3)_4]_2[Ru(CN)_6]\cdot 4H_2O$ |
| ROXRAB | 2.053 Å | Vorobyeva, S. N.; Rudzis, Z. V.; Sukhikh, T. S.; Filatov, E. Y.; Plusnin, P. E.; Nadolinny,V. A.; Bogomyakov, A. S.; Korenev, S. V. New. J. Chem. 2024, 48, 15894. $[Pt(NH3)_4][VO(O_2C_2O_2)_2]_n$ |
| 65766 | 2.054 Å | Rochon, F.D.; Kong, P.C.; Melanson, R. *Acta Crystallogr., Sect. C* **1990**, *46*, 8. $[Pt(NH_3)_4]TcO_4$ |
| JIYWOD | 2.056 Å | Rochon, F. D.; Melanson, R. *Acta Crystallogr., Sect. C* **1991**, *47*, 2300. $[Pt(NH_3)_4][PtCl_3(NH_2CH(CH_3)_2)]_2$ |
| QUDSAJ | 2.057 Å | Williams, A. R.; Hall, L. A.; White, A. J. P.; Williams, D. J. *Inorg. Chim. Acta* **2001**, *314*, 117. $[Pt(NH_3)_4](C_{10}H_5O_3)_2\cdot H_2O$ |
| SIDROM | 2.057 Å | Zhou, M.; Pfennig, B. W.; Steiger, J.; Van Engen, D.; Bocarsly, A. B. *Inorg. Chem.* **1990**, *29*, 2456. $Pt(NH_3)_4]_2[(NC)_5CNPt(NH_3)_4FeNPt(CN)_5)]\cdot 9H_2O$ |
| SOPKUE | 2.057 Å | Yusenko, K. V.; Filatov, E. Y.; Vasilchenko, D. B.; Baidina, I. A.; Zadesenez, A. V.; Shubin, Y. V. *Z. Krist. Cryst. Mater*. **2007**, *222*, 289. $[Pd(NH_3)_4][Cu(H_2O)_2(C_2O_4)_2]$ |
| XOMSOJ | 2.057 Å | Jinzhen, L.; Abrahams, B. F.;Winther-Jensen, B.; Martin, L. L.; Bond, A. M. *ChemCatChem* **2014**, *6*, 2345. $[Pt(NH_3)_4](C_{12}F_4N_4)_2\cdot 2(CH_3)_2NCHO$ |
| 15711 | 2.058 Å | Richardson, J. G.; Benjamin, H.; Moggach, S. A.; Warren, L. R.; Warren, M. R.; Allan, D. R.; Saunders, L. K.; Morrison, C. A.; Robertson, N. *Phys. Chem. Chem. Phys.* **2020**, *22*, 17668. $[Pt(NH_3)_4][PtCl_4]$ |
| 35199 | 2.059 Å | Tanaka, M.; Tsujikawa, I.; Toriumi, K.; Ito, T. *Acta Crystallogr., Sect. B* **1982**, *38*, 2793. $[Pt(NH_3)_4][PtBr_2(NH_3)_4](HSO_4)_4$ |

| | | |
|---|---|---|
| 194210 | 2.060 Å | Lucier, B. E. G.; Johnston, K. E.; Xu, W.; Hanson, J. C.; Senanayake, S. D.; Yao, S.; Bourassa, M. W.; Srebro, M.; Autschbach, J.; Schurko, R. W. *J. Am. Chem. Soc.* **2014**, *136*, 1333. $[Pt(NH_3)_4][PtCl_4]$ |
| 26615 | 2.063 Å | Atoji, M.; Richardson, Jr., J. W.; Rundle, R. E. *J. Am. Chem. Soc.* **1957**, *79*, 3017. $[Pt(NH_3)_4][PtCl_4]$ |
| **Mean** | **2.047 Å/49 structures** | |

**Copper(I)**

| | | |
|---|---|---|
| *Two-coordinatioon, linear configuration* | | |
| YAMTIT | 1.872 Å | Ermolaev, A. V.; Smolentsev, A. I.; Mironov, Y. V *J. Struct. Chem.* **2021**, *62*, 585. $[Cu(NH_3)_2]_2[Cu_3Re_6C_6H_{10}N_9OSe_8]$ |
| 428698 | 1.885 Å | Woidy, P.; Karttunen, A. J.; Widenmeyer, M.; Niewa, R.; Kraus, F. *Chem. – Eur. J.* **2015**, *21*, 3290. $[Cu(NH_3)_2]F{\cdot}NH_3$ |
| 428697 | 1.893 Å | Woidy, P.; Karttunen, A. J.; Widenmeyer, M.; Niewa, R.; Kraus, F. *Chem. – Eur. J.* **2015**, *21*, 3290. $[Cu(NH_3)_3]_2[Cu_2(NH_3)_4]F_4{\cdot}4NH_3$ |
| 170947 | 1.898 Å | Margraf, G.; Bats, J. W.; Bolte, M.; Lerner, H. W.; Wagner, M. *Chem. Commun.* **2003**, 956. $[[Cu(NH_3)_2]Br$ |
| 410751 | 1.945 Å | Cascales, C.; Gutierrez Puebla, E.; Iglesias, M.; Monge, M. A.; Ruiz Valero, C. *Angew. Chem., Int. Ed.* **1999**, *38*, 2436. $NH_4[Cu(NH_3)_2][Ge_9O_{19}]$ |
| **Mean** | **1.887 Å/4 structures** | |
| | | |
| *Three-coordinatioon, T-shaped configuration* | | |
| 428697 | 1.937 + 2.257 Å | Woidy, P.; Karttunen, A. J.; Widenmeyer, M.; Niewa, R.; Kraus, F. *Chem. – Eur. J.* **2015**, *21*, 3290. $[Cu(NH_3)_3]_2[Cu_2(NH_3)_4]F_4{\cdot}4NH_3$ |

**Copper(II)**

| | | |
|---|---|---|
| *Four-coordinatioon, square-planar configuration* | | |
| 1961 | 1.997 Å | Morosin, B.; Fallon, P.; Valentine, J. S. *Acta Crystallogr., Sect E* **1975**, *31*, 2220. $[Cu(NH_3)_4][PtCl_4]$ |
| 14222 | 1.997 Å | Baglio, J.A.; Vaughan, P.A. *J. Inorg. Nucl. Chem.* **1970**, *32*, 803. $[Cu(NH_3)_4][CuBr_2]_2$ |
| 14223 | 1.997 Å | Baglio, J.A.; Vaughan, P.A. *J. Inorg. Nucl. Chem.* **1970**, *32*, 803. $[Cu(NH_3)_4][CuCl_2]_2{\cdot}H_2O$ |
| TIBWUX | 2.007 Å | Peng, S.-W.; Miao, Y.-L.; Song, W.-D. *Acta Crystallogr., Sect E* **2007**, *63*, m1145. $[Cu(NH_3)_4](C_5H_3N_2O_2)_2$ |
| DAXXEG | 2.012 Å | Suzuki, S.; Morita, Y.; Fukui, K.; Sato, K.; Shiomi, D.; Takui, T.; Nakasuji, K. *Inorg. Chem.* **2005**, *44*, 8197. $[Cu(NH_3)_4](C_7H_3N_4)_2$ |
| XEGZUI | 2.015 Å | Das, J.; Shem-Tov, D.; Zhang, S.; Gao, C.-Z.; Zhang, L.; Yao, C.; Flaxer, E.; Stierstorfer, J.; Wurzenberger, M.; Rahinov, I.; Gozin, M. *Chem. Eng. J.* **2022**, *443*, 136246. $[Cu(NH_3)_4](C_2N_5O_4S)_2$ |

| | | |
|---|---|---|
| XAQTOA | 2.019 Å | Jia, L.-N.; Hou, L. *Acta Crystallogr., Sect C* **2012**, *68*, m45. $[Cu(NH_3)_4]_n[Cu_4(CN)_6]_n$ |
| XAQTOA01 | 2.020 Å | Grifasi, F.; Priola, E.; Chierotti, M. R.; Diana, E.; Garino, C.; Gobetto, R. *Eur. J. Inorg. Chem.* **2016**, 2975. $[Cu(NH_3)_4]_n[Cu_4(CN)_6]_n$ |
| **Mean** | **2.008 Å/8 structures** | |

*Five-coordination, square pyramidal configuration*

| | | |
|---|---|---|
| ONEVIN | 2.028+2.267 Å | Mironov, Y. V.; Brylev, K. A.; Kim, S.-J.; Kozlova, S. G.; Kitamura, N.; Fedorov, V. E. *Inorg. Chim. Acta* **2011**, *370*, 363. $[Cu(NH_3)_5]_2[Re_6Se_8(CN)_4(OH)_2]\cdot 8H_2O$ |
| 201229 | 2.029+2.192 Å | Duggan, M.; Ray, N.; Hathaway, B. J.; Tomlinson, G.; Brint, P.; Pelin, K. *J. Chem. Soc., Dalton Trans.* **1980**, 1342. $K[Cu(NH_3)_5](PF_6)_3$ |
| 262582 BAWLOC | 2.031+2.282 Å | Mironov, Y. V.; Gayfulin, Y. M.; Kozlova, S. G.; Smolentsev, A. I.; Tarasenko, M. S.; Nizovtsev, A. S.; Fedorov, V. E. *Inorg., Chem.* **2012**, *51*, 4359. $[Cu(NH_3)_5]_{2.6}[Re_{12}CS_{14}(SO_2)_3(CN)_6]_{0.6}[Re_{12}CS_{14}(SO_2)_2(SO_3)(CN)_5(CN)][Cu(NH_3)_4]_{0.4}\cdot 5H_2O$ |
| 262580 BAWLES | 2.032+2.262 Å | Mironov, Y. V.; Gayfulin, Y. M.; Kozlova, S. G.; Smolentsev, A. I.; Tarasenko, M. S.; Nizovtsev, A. S.; Fedorov, V. E. *Inorg., Chem.* **2012**, *51*, 4359. $[Cu(NH_3)_5][Re_{12}CS_{14}(SO_2)_3(CN)_6]\cdot 9.5H_2O$ |
| ROPROH | 2.039+2.211 Å | Breen, O. D.; Keene, T. D. *Acta Crystallogr., Sect. E* **2024**, *80*, 596. $[Cu(NH_3)_5]Cl_2\cdot H_2NC(O)NH_2$ |
| GUSMIT | 2.040+2.143 Å | Wisser, B.; Labahn, A.; Nather, C.; Janiak, C. *Z. Anorg. Allg. Chem.* **2020**, *646*, 734. $[Cu(NH_3)_5](C_{20}H_{12}O_2)\cdot 2C_{20}H_{14}O_2$ |
| GUSMOZ | 2.054+2.151 Å | Wisser, B.; Labahn, A.; Nather, C.; Janiak, C. *Z. Anorg. Allg. Chem.* **2020**, *646*, 734. $[Cu(NH_3)_5](C_{20}H_{12}O_2)\cdot 2C_{20}H_{14}O_2$ |
| **Mean** | **2.036 + 2.215 Å/7 structures** | |

*Six-coordination, Jahn-Teller distorted octahedral configuration*

| ICSD/CSD | *d*(Cu-N) | Reference and formula |
|---|---|---|
| HEKDEJ | 1.867+1.939+2.382 Å | Gayfulin, Y. M.; Smolentsev, A. I.; Kozlova, S. G.; Novozhilov, I. N.; Plyusnin, P. E.; Kompankov, N. B.; Mironov, Y. V. *Inorg. Chem.* **2017**, *56*, 12389. $K_2[Cu(NH_3)_6][Cu(NH_3)(H_2O)_5]_2[CuRe_{12}C_7H_{12}N_{10}O_3S_{14}]_2\cdot 10H_2O$ |
| OLELEW | 2.048+2.156 Å | Shmilovits, M.; Diskin-Posner, Y.; Vinodu, M.; Goldberg, I. *Cryst. Growth Des.* **2003**, *3*, 855. $[Cu(NH_3)_6][PtC_{48}H_{24}N_4O_8]\cdot 4H_2O$ |
| 22239 | 2.075+2.615 Å | Distler, T.; Vaughan, P. A. *Inorg. Chem.* **1967**, *6*, 126. $[Cu(NH_3)_6]Cl_2$ |
| 419094 | 2.093+2.340 Å | Woidy; P.; Meng, W.; Kraus, F. *Z. Naturforsch., Teil B* **2014**, *69*, 1. $[Cu(NH_3]_6]F_2\cdot 10H_2O$ |
| 22240 | 2.149+2.448 Å | Distler, T.; Vaughan, P. A. *Inorg. Chem.* **1967**, *6*, 126. $[Cu(NH_3)_6]Br_2$ |

| | | |
|---|---|---|
| **Mean** | **2.091+2.390 Å/4 structures** | |

*Six-coordination, octahedral configuration*

| | | |
|---|---|---|
| 10255 | 2.064 Å | Raymond, K. N.; Meek, D. W.; Ibers, J. A. *Inorg. Chem.* **1968**, *7*, 1111. $[Cu(NH_3)_6][CuCl_5]$ |
| 35153 | 1.966 Å | Bernal, I.; Korp, J. D.; Schlemper, E. O.; Hussain, M. S. *Polyhedron* **1982**, *1*, 365. $[Cu(NH_3)_6][CuCl_5]$ |

**Copper(III)**

*Four-coordination square-planar configuration*

| | | |
|---|---|---|
| YIZGIZ | 2.239 Å | Eschenroeder, E. C.V.; Turrina, A.; Picone, A. L.; Cinque, G.; Frogley, M. D.; Cox, P. A.; Howe, R. F.; Wright, P. A. *Chem. Mater.* **2014**, *26*, 1434. $(C_2H_5)_4N)_n[Cu(NH_3)_4]_n[Al_3(PO_4)_3]_{4n}$ |
| YIZGOF | 2.239 Å | Eschenroeder, E. C.V.; Turrina, A.; Picone, A. L.; Cinque, G.; Frogley, M. D.; Cox, P. A.; Howe, R. F.; Wright, P. A. *Chem. Mater.* **2014**, *26*, 1434. $(C_2H_5)_4N)_n[Cu(NH_3)_4]_n[Al_3(PO_4)_3]_{4n}\cdot nH_2O$ |

**Silver(I)**

*Two-coordination, linear configuration*

| | | |
|---|---|---|
| TACKUH | 2.032 Å | Chen, G.-H.; He, Y.-P.; Liang, F.-P.; Zhang, L.; Zhang, J. *Dalton Trans.* **2020**, *49*, 17194. $((CH_3)_4N)((CH_3)_2NH_2)[Ag(NH_3)_2]_6[Ti_4C_{138}H_{72}O_{36}]\cdot 28H_2O$ |
| LURDIM | 2.090 Å | Sun, D.; Zhang, N.; Luo, G.-G.; Xu, Q.-J.; Huang, R.-B.; Zheng, L.-S. *Polyhedron* **2010**, *29*, 1842. $[Ag(NH_3)_2]_4(C_6H_2(COO)_4)\cdot 5H_2O$ |
| 419924 | 2.095 Å | Kraus, F.; Baer, S. A.; Fichtl, M. B. *Eur. J. Inorg. Chem.* **2009**, 441. $[Ag(NH_3)_2]F\cdot 2NH_3$ |
| KIMZOY | 2.098 Å | Zhu, Y.; Li, X.; Li, Y.; Wang, Q.; Lu, X. *Inorg. Chim. Acta* **2019**, *484*, 42. $[Ag(NH_3)_2](C_8H_5Cl_2O_2)$ |
| TACKIV | 2.099 Å | Chen, G.-H.; He, Y.-P.; Liang, F.-P.; Zhang, L.; Zhang, J. *Dalton Trans.* **2020**, *49*, 17194. $((CH_3)_2NH_2)_5[Ag(NH_3)_2]_3[Ti_4C_{138}H_{72}O_{36}]$ |
| GAPDEI | 2.100 Å | Deng, Z.-P.;, S. G.; Ng, S. W. *Acta Crystallogr,, Sect. E* **2012**, *68*, m225. $NH_4[Ag(NH_3)_2]_2[Ag_2C_{12}H_{24}N_8](C_6H_4ClO_4S)_2\cdot 3H_2O$ |
| TACKOB | 2.100 Å | Chen, G.-H.; He, Y.-P.; Liang, F.-P.; Zhang, L.; Zhang, J. *Dalton Trans.* **2020**, *49*, 17194. $((CH_3)_4N)((CH_3)_2NH_2)_4[Ag(NH_3)_2]_3[Ti_4C_{138}H_{72}O_{36}]\cdot 1.5H_2O$ |
| 69986 | 2.106 Å | Zak, Z.; Ruzicka, A.; Glovyak, T. *Can. J. Chem.* **1991**, *69*, 1080. $[Ag(NH_3)_2][Ag(SeO_3N)Ag(NH_3)]\cdot 2H_2O$ |
| 125310 | 2.107 Å | Yang, Y.-C.; Liu, X.; Deng, X.-B.; Wu, L.-M.; Chen. L. *JACS Au* **2022**, *2*, 2059. $[Ag(NH_3)_2]_2SO_4$ |
| WIKDIF | 2.107 Å | Haiges, R.; Christe, K. O. *Inorg. Chem.* **2013**, *52*, 7249. $[Ag(NH_3)_2]_2[Ag_4C_{12}N_{42}O_{36}]$ |
| KURTAT | 2.108 Å | Sun, D.; Luo, G.-G.; Zhang, N.; Huang, R.-B. *Acta Crystallogr., Sect. E* **2010**, *66*, m406. $[Ag(NH_3)_2]_2(C_8H_3NO_6)\cdot H_2O$ |

| | | |
|---|---|---|
| 36585 | 2.110 Å | Zachwieja, U.; Jacobs, H. *Z. Kristallogr.* **1992**, *201*, 207. $[Ag(NH_3)_2]_2SO_4$ |
| 433114 | 2.110 Å | Sun, C.; Zhang, C.; Jiang, C.; Yang, C.; Du, Y.; Zhao, Y.; Hu, B.; Zheng, Z. *Nature Commun.* **2018**, *9*, 1269. $[Ag(NH_3)_2][Ag_3(N_5)_4]$ |
| VUSQUW | 2.111 Å | Sun, D.; Luo, G.-G.; Zhang, N.; Wei, Z.-H.; Yang, C.-F.; Huang, R.-B.; Zheng, L.-S. *Chem. Lett.* **2010**, *39*, 190. $[Ag(NH_3)_2][Ag(C_{12}H_9N_5O_8)Ag(NH_3)_2]$ |
| 78195 | 2.112 Å | Yang, Y.-C.; Liu, Xi.; Zhu, C.-F.; Zhu, L.; Wu, L.-M.; Chen, L. *Angew. Chem.* **2023**, *62*, e202301404. $[Ag(NH_3)_2]_2SO_4$ |
| 111796 | 2.113 Å | Fogaca, L. A.; Kováts, É.; Németh, G.; Kamarás, K.; Béres, K. A.; Németh, P.; Petruševski, V.; Bereczki, L.; Holló, B. B.; Sajó, I. E.; Klébert, S; Farkas, A.; Szilágyi, I. M.; Kótai, L. *Inorg. Chem.* **2021**, *60*, 3749. $[Ag(NH_3)_2]MnO_4$ |
| LAJWAV | 2.113 Å | You, Z.-L.; Zhu, H.-L.; Liu, W.-S. *Acta Crystallogr., Sect. E* **2004**, *60*, m1624. $[Ag(NH_3)_2](C_6H_2(NO_2)_3O)$ |
| 77995 | 2.114 Å | Yang, Y.-C.; Liu, Xi.; Zhu, C.-F.; Zhu, L.; Wu, L.-M.; Chen, L. *Angew. Chem.* **2023**, *62*, e202301404. $[Ag(NH_3)_2]_2SeO_4$ |
| 100238 | 2.114 Å | Maurer, H. M.; Wiess, A. *Z. Kristallogr.* **1977**,*146*, 227. $[Ag(NH_3)_2][Ag(NO_2)_2]$ |
| 250264 | 2.114 Å | Kokunov, Y. V.; Gorbunova, Y. E.; Khmelevskaya, L. V. *Zh. Neorg. Khim.* **2005**, *50*, 304. $[Ag(NH_3)_2]PF_6$ |
| HAMMIS | 2.114 Å | Zhong-Lu You, Z.-L.; Zhu, H.-L.; Liu,. W.-S. *Acta Crystallogr., Sect. E* **2004**, *60*, m1903. $[Ag(NH_3)_2](p\text{-}O_2NC_6H_4(COO))$ |
| JEHWOJ | 2.114 Å | Nygren, C. L.; Coppens, P. CCDC deposition number 604898, **2006**. $[Ag(NH_3)_2][AgC_{12}H_4N_6O_{14})$ |
| 253559 | 2.117 Å | Kraus, F.; Fichtl, M.; Baer, S. *Acta Crystallogr., Sect. E* **2016**, *72*, 1860. $[Ag(NH_3)_2]_2[Ag(NH_3)_3][SnF_6]F_2$ |
| LELNUN | 2.117 Å | Sun, D.; Liu, F.-J.; Huang, R.-B.; Zheng, L.-S. *CrystEngComm* **2013**, *15*, 1185. $[Ag(NH_3)_2][AgC_8H_3NO_6]$ |
| AWEHUG | 2.118 Å | Deng, Z.-P.; Huo, L.-H.; Li, M.-S.; Zhang, L.-W. Zhu, Z.-B.; Zhao, H.; Gao, S. *Cryst. Growth Des.* **2011**, *11*, 3090. $(NH_4)_2[Ag(NH_3)_2]2(C_6H_4O_8S_2)\cdot 5H_2O$ |
| 35735 | 2.119 Å | Yamaguchi, T.; Lindqvist, O. *Acta Chem. Scand., Ser. A* **1983**, *37*, 685. $[Ag(NH_3)_2]NO_3$ |
| QUWVEK | 2.119 Å | Sun, D.; Zhang, N.; Huang, R.-B.; Zheng, L.-S. *Acta Crystallogr., Sect. C* **2010**, *66*, m174. $[Ag(NH_3)_2][Ag(C_7H_5N_2O_4)_2]$ |
| TEJKOJ | 2.120 Å | Wu, G.-Q.; Chen, W.-Z. *Acta Crystallogr., Sect. E* **2006**, *62*, m1144. $[Ag(NH_3)_2]_2[Pt(C_2O_4)_2]$ |
| BADGET | 2.121 Å | Zheng, S.-L.; Tong, M.-L.; Chen, X.-M.; Ng, S.-W. *J. Soc. Chem., Dalton Trans.* **2002**, 360. $[Ag(NH_3)_2]_2[Ag_2C_{12}H_{24}N_8](C_6H_2(COO)_4)\cdot 3H_2O$ |
| WIJVUG | 2.121 Å | Pajunen, A.; Pajunen, S. *Acta Crystallogr., Sect. C* **1994**, *50*, 1884. $[Ag(NH_3)_2]_2[Ni(C_2H_3N_3O_2)_2]$ |
| 426384 | 2.122 Å | Woidy, P.; Kraus, F. *Z. Anorg. Allg. Chem.* **2013**, *639*, 2643. $[Ag(NH_3)_2]CH_3COO$ |

| | | |
|---|---|---|
| 111795 | 2.123 Å | Fogaca, L. A.; Kováts, É.; Németh, G.; Kamarás, K.; Béres, K. A.; Németh, P.; Petruševski, V.; Bereczki, L.; Holló, B. B.; Sajó, I. E.; Klébert, S; Farkas, A.; Szilágyi, I. M.; Kótai, L. *Inorg. Chem.* **2021**, *60*, 3749. $[Ag(NH_3)_2]MnO_4$ |
| RIVTUM | 2.123 Å | Whitcomb, D. R.; Rajeswaran, M. *Inorg. Chim. Acta* **2008**, *361*, 1357. $[Ag(NH_3)_2][Ag(NH_3)(OC_8Cl_4O_4)]\cdot H_2O$ |
| LELNUN01 | 2.124 Å | Zhang, T.; Wang, D.-F.; Huang, R.-B.; Zheng, L.-S. *Inorg. Chim. Acta* **2015**, *427*, 299. $[Ag(NH_3)_2][AgC_8H_3NO_6]$ |
| LELPID | 2.125 Å | Sun, D.; Liu, F.-J.; Huang, R.-B.; Zheng, L.-S. *CrystEngComm* **2013**, *15*, 1185. $[Ag(NH_3)_2][Ag_5C_{32}H_{18}N_6O_{24}]$ |
| 412419 | 2.127 Å | Nockemann, P.; Meyer, G. *Z. Anorg. Allg. Chem.* **2002**, *628*, 1636. $[Ag(NH_3)_2]ClO_4$ |
| XIFKOE | 2.129 Å | Zheng, S.-L.; Volkov, A.; Nygren, C. L.; Coppens, P. *Chem. Eur. J.* **2007**, *13*, 8583. $[Ag(NH_3)_2](C_{20}H_{17}O_3)\cdot C_{20}H_{18}O_3\cdot CH_3CN$ |
| JEHWUP | 2.132 Å | Nygren, C. L.; Coppens, P. CCDC deposition number 604912, **2006**. $[Ag(NH_3)_2](C_{29}H_{27}O_3)\cdot C_{29}H_{28}O_3\cdot 4H_2O$ |
| XUSFAT | 2.132 Å | Gerasimchuk, N.; Esaulenko, A. N.; Dalley, N. K.; Moore, C. *Dalton Trans.* **2010**, *39*, 749. $[Ag(NH_3)_2][C_3H_2N_3O_2]$ |
| 252664 | 2.134 Å | Kravchenko, E. A.; Gippius, A. A.; Korlyukov, A. A.; Vologzhanina, A. V.; Avdeeva, V. V.; Malinina, E. A.; Ulitin, E. O.; Kuznetsov, N. T. *Inorg. Chim. Acta* **2016**, *447*, 22. $[Ag(NH_3)_2]_2B_{10}Cl_{10}$ |
| JEHXEA | 2.134 Å | Nygren, C. L.; Coppens, P. CCDC deposition number 604909, **2006**. $[Ag(NH_3)_2](C_{19}H_{15}O_3)\cdot 3H_2O$ |
| UBEFUF | 2.134 Å | Kravchenko, E. A.; Gippius, A. A.; Korlyukov, A. A.; Vologzhanina, A. V.; Avdeeva, V. V.; Malinina, E. A.; Ulitin, E. O.; Kuznetsov, N. T. *Inorg. Chim. Acta* **2016**, *447*, 22. $[Ag(NH_3)_2]_2(B_{10}Cl_{10})$ |
| XOBDOI | 2.134 Å | Zheng, G.-L.; Li, Y.-Y.; Deng, R.-P.; Song, S.-Y.; Zhang, H.-J. *CrystEngComm* **2008**, *10*, 658. $[Ag(NH_3)_2]_{2n}[Ag_{12}K_4C_{150}H_{132}N_4O_{52}]_nCl_{2n}\cdot 11nH_2O$ |
| AWEGOZ | 2.135 Å | Deng, Z.-P.; Huo, L.-H.; Li, M.-S.; Zhang, L.-W. Zhu, Z.-B.; Zhao, H.; Gao, S. *Cryst. Growth Des.* **2011**, *11*, 3090. $[Ag(NH_3)_2](C_7H_7O_4S)$ |
| 99527 | 2.140 Å | Wickleder, M.S.; Pley, M. *J. Solid State Chem.* **2004**, *177*, 2073. $[Ag(NH_3)_2]Ag[OsO_3N]_2$ |
| JEHWID | 2.140 Å | Nygren, C. L.; Coppens, P. CCDC deposition number 604899, **2006**. $[Ag(NH_3)_2](C_{19}H_{15}O_3)\cdot C_{19}H_{16}O_3\cdot C_{10}H_8O_2\cdot 4H_2O$ |
| JAQPEX | 2.141 Å | Paul, B.; Nather, C.; Fromm, K. M.; Janiak, C. *CrystEngComm* **2005**, *7*, 309. $[Ag(NH_3)_2](C_{20}H_{13}O_2)\cdot C_{20}H_{14}O_2\cdot 2CH_3OH\cdot H_2O$ |
| XIWXUY | 2.143 Å | Galassi, R.; Ricci, S.; Burini, A.; Macchioni, A.; Rocchigiani, L.; Marmottini, F.; Tekarli, S. M.; Nesterov, V. N.; Omary, M. A. *Inorg. Chem.* **2013**, *52*, 14124. $[Ag(NH_3)_2][Ag_3C_{12}H_4N_{16}O_{16}]$ |

| | | |
|---|---|---|
| XOBDIC | 2.144 Å | Zheng, G.-L.; Li, Y.-Y.; Deng, R.-P.; Song, S.-Y.; Zhang, H.-J. *CrystEngComm* **2008**, *10*, 658. $[Ag(NH_3)_2](C_{28}H_{23}O_4)$ |
| JAQPOH | 2.149 Å | Paul, B.; Nather, C.; Fromm, K. M.; Janiak, C. *CrystEngComm* **2005**, *7*, 309. $[Ag(NH_3)_2](C_{20}H_{13}O_2)\cdot C_{20}H_{14}O_2\cdot 2CH_3OH\cdot 2H_2O$ |
| TACKUQ | 2.154 Å | Chen, G.-H.; He, Y.-P.; Liang, F.-P.; Zhang, L.; Zhang, J. *Dalton Trans.* **2020**, *49*, 17194. $((CH_3)_2NH_2)_2[Ag(NH_3)_2]_6[Ti_4C_{138}H_{72}O_{36}]$ |
| 64628 | 2.158 Å | Ziegler, B.; Seitz, K.; Babel, D. *Z. Naturforsch., Teil B* **1988**, *43*, 1589. $[Ag(NH_3)_2]Ag_2[Co(CN)_6]$ |
| 65949 | 2.164 Å | Zachwieja, U.; Jacobs, H. *Z. Anorg. Allg. Chem.* **1989**, *571*, 37. $[Ag(NH_3)_2]NO_3$ |
| 64627 | 2.165 Å | Ziegler, B.; Seitz, K.; Babel, D. *Z. Naturforsch., Teil B* **1988**, *43*, 1589. $[Ag(NH_3)_2]Ag_2[Fe(CN)_6]$ |
| 410752 | 2.170 Å | Cascales, C.; Gutierrez Puebla, E.; Iglesias, M.; Monge, M. A.; Ruiz Valero, C. *Angew. Chem., Int. Ed.* **1999**, *38*, 2436. $NH_4[Ag(NH_3)_2][Ge_9O_{19}]$ |
| **Mean** | **2.124 Å/54 structures** | |

*Three-coordination, triangular configuration*

| | | |
|---|---|---|
| 240350 | 2.263 Å | Nilsson, K. B.; Persson, I.; Kessler, V. G. *Inorg. Chem.* **2006**, *45*, 6912. $[Ag(NH_3)_3]ClO_4\cdot 0.47NH_3$ |
| 430314 | 2.274 Å | Müller, T. G.; Buchner, M. R.; Scheubeck, T. J.; Korber, N.; Kraus, F. *Z. Anorg. Allg. Chem.* **2016**, *642*, 796. $[Ag(NH_3)_3]N_3$ |
| 65948 | 2.283 Å | Zachwieja, U.; Jacobs, H. *Anorg. Allg. Chem.* **1989**, *571*, 37. $[Ag(NH_3)_3]NO_3$ |
| 253559 | 2.299 Å | Kraus, F.; Fichtl, M.; Baer, S. *Acta Crystallogr., Sect. E* **2016**, *72*, 1860. $[Ag(NH_3)_2]_2[Ag(NH_3)_3][SnF_6]F_2$ |
| **Mean** | **2.280 Å/4 structures** | |

*Four-coordination, tetrahedral configuration*

| | | |
|---|---|---|
| 430315 | 2.348 Å | Müller, T. G.; Buchner, M. R.; Scheubeck, T. J.; Korber, N.; Kraus, F. *Z. Anorg. Allg. Chem.* **2016**, *642*, 796. $[Ag(NH_3)_4]N_3\cdot NH_3$ |
| **Mean** | **2.348 Å/1 structure** | |

*Dimeric $[(NH_3)_3AgAg(NH_3)_4]$ complex*

| | | |
|---|---|---|
| 418758 | 2.277+2.342 Å | Meng W.; Kraus, F. *Eur. J. Inorg. Chem.* **2008**, 3068. $[(NH_3)_3AgAg(NH_3)_4][HfF_6]\cdot NH_3$ |
| | 3.141 Å | |
| 418759 | 2.279+2.350 Å | Meng W.; Kraus, F. *Eur. J. Inorg. Chem.* **2008**, 3068. $[(NH_3)_3AgAg(NH_3)_4][ZrF_6]\cdot NH_3$ |
| | 3.132 Å | |
| **Mean** | **2.278+2.346 Å/2 structures** | |

**Gold(I)**

*Two-coordination, linear configuration*

| | | |
|---|---|---|
| 80216 | 2.020 Å | Mingos, D. M. P.; Yau, J.; Menzer, S.; Williams, D. J. *J. Chem. Soc., Dalton Trans.* **1995**, 319. $[Au(NH_3)_2]_2Br_2$ |
| 425324 | 2.032 Å | Scherf, L. M.; Baer, S. A.; Kraus, F.; Bawaked, S. M.; Schmidbaur, H. *Inorg. Chem.* **2013**, *52*, 2157. $[Au(NH_3)_2]Cl \cdot 4NH_3$ |
| DIWZOB | 2.038 Å | Strey, M.; Doring, C.; Jones, P. G. *Z. Naturforsch.,Teil B* **2018**, *73*, 125. $[Au(NH_3)_4][Au(SCN)_2]$ |
| 249444 | 2.046 Å | Zheng S.; Nygren, C. L.; Messerschmidt, M.; Coppens, P. *Chem. Commun.* **2006**, 3711. $[Au(NH_3)_2]NO_3$ |
| 249443 | 2.053 Å | Zheng S.; Nygren, C. L.; Messerschmidt, M.; Coppens, P. *Chem. Commun.* **2006**, 3711. $[Au(NH_3)_2]ClO_4$ |
| **Mean** | **2.038 Å/5 structures** | |

**Gold(III)**

*Four-coordination, square planar configuration*

| | | |
|---|---|---|
| 420 | 2.024 Å | Weishaupt, M.; Straehle, J. *Z. Naturforsch.,Teil B* **1976**, *31*, 554. $[Au(NH_3)_4](NO_3)_3$ |
| **Mean** | **2.024 Å/1 structure** | |

**Zinc(II)**

*Four-coordination, tetrahedral configuration*

| ICSD/CSD | *d*(Zn-N) | Reference and formula |
|---|---|---|
| NUPDEJ | 2.006 Å | Virovets, A. V.; Gayfulin, Y. M.; Peresypkina, E. V.; Mironov, Y. V.; Naumov, N. G. *CrystEngComm* **2015**, *17*, 1477. $[Zn(NH_3)_4]_2[Mo_{12}Re_6ZnS_{24}(CN)_{18}] \cdot 2H_2O$ |
| 425027 | 2.007 Å | Kysliak, O.; Beck, J. *Eur. J. Inorg. Chem.* **2013**, 124. $[Zn(NH_3)_6][Zn(NH_3)_4]_2(TeSe_3)_3$ |
| YAGGET | 2.007 Å | Yang Qu, Y.; Liu, Z.-D.; Tan, M.-Y.; Zhu, H.-L. *Acta Crystallogr, Sect. E* **2004**, *60*, m1343. $[Zn(NH_3)_6](C_6H_2(NO_2)_3O)_2 \cdot 3H_2O$ |
| TAQPIN | 2.010 Å | Szimhardt, N.; Bolter, M. F.; Born, M.; Klapotke, T. M.; Stierstorfer, J. *Dalton Trans.* **2017**, *46*, 5033. $[Zn(NH_3)_6](C_2N_{12}O_4)_2$ |
| 31874 | 2.011 Å | Yamaguchi, T.; Lindqvist, O. *Acta Chem. Scand., Ser. A* **1981**, *35*, 811. $[Zn(NH_3)_4]I_2$ |
| ZEHPUA | 2.011 Å | Imer, M. R.; Gonzalez, M.; Veiga, N.; Kremer, C.; Suescun, L.; Arizaga, L. *Dalton Trans.* **2017**, *46*, 15736. $[Zn(NH_3)_6](H_4B_4O_9)$ |
| 74790 | 2.015 Å | Hillebrecht, H.; Thiele, G.; Koppenhoefer, A.; Vahrenkamp, H. *Z. Naturforsch., Teil B* **1994**, *49*, 1163. $[Zn(NH_3)_4](ClO_4)_2$ |
| 425027 | 2.016 Å | Kysliak, O.; Beck, J. *Eur. J. Inorg. Chem.* **2013**, 124. $[Zn(NH_3)_4]TeSe_3$ |

| | | |
|---|---|---|
| 97431 | 2.016 Å | Migdal-Mikuli, A.; Mikuli, E.; Hetmanczyk, L.; Natkaniec, I.; Holderna-Natkaniec, K.; Lasocha, W. *J. Solid State Chem.* **2003**, *174*, 357. $[Zn(NH_3)_4](BF_4)_2$ |
| 162384 | 2.016 Å | Sajo, I.E.; Kotai, L.; Keresztury, G.; Gacs, I.; Pokol, G.; Kristof, J.; Soptrajanov, B.; Petrusevski, V. M.; Timpu, D.; Sharma, P. K. *Helv. Chim. Acta* **2008**, *91*, 1646. $[Zn(NH_3)_4](MnO_4)_2$ |
| CEKDAB | 2.020 Å | Kalinina, I. V.; Pervukhina, N.V.; Podberezskaya, N. V.; Fedin, V. P. Russ. *J. Coord. Chem.* **2002**, *29*, 389. $[Zn(NH_3)_4][(Mo_4Te_4(CN)_{12}]$ |
| 426403 | 2.034 Å | Kysliak, O.; Beck, J. *Z. Anorg. Allg. Chem.* **2013**, *639*, 2860. $[Zn(NH_3)_4][(NH_3)_3ZnSbSe_3]\cdot 3NH_3$ |
| SOTSOJ | 2.037 Å | Klufers, P.; Wilhelm, U. *J. Organometal. Chem.* **1991**, *421*, 39. $[Zn(NH_3)_4][(CO)_5CrHCr(CO)_5]_2$ |
| 4421 | 2.047 Å | Stomberg, R. Acta Chem. Scand. 1969, 23, 2755. $[Zn(NH_3)_4][Mo(O_2)_4]$ |
| 165104 | 2.048 Å | Skogareva, L. S.; Minaeva, N. A.; Filippova, T. V. *Zh. Neorg. Khim.* **2009**, *54*, 1411. $[Zn(NH_3)_4]S_2O_8$ |
| 4399 | 2.052 Å | Guggenberg, G. J. *Inorg. Chem.* **1969**, *8*, 2771. $[Zn(NH_3)_4]B_8H_8$ |
| FUZCUY | | |
| 25305 | 2.052 Å | Müller, A.; Boeschen, I.; Sievert, W. *Z. Naturforsch., Teil B* **1970**, *25*, 311. $[Zn(NH_3)_4][OsO_3N]_2$ |
| 100307 | 2.057 Å | Tebbe, K. F. *Z. Kristallogr.* **1980**, *153*, 297. $[Zn(NH_3)_4](I_3)_2$ |
| NUNPOD | 2.162 Å | Virovets, A. V.; Gayfulin, Y. M.; Peresypkina, E. V.; Mironov, Y. V.; Naumov, N. G. *CrystEngComm* **2015**, *17*, 1477. $[Zn(NH_3)_4]_2[Mo_3Re_3ZnS_8(CN)_6]\cdot 2H_2O$ |
| **Mean** | **2.022 Å/18 structures** | |

*Six-coordination, octahedral configuration*

| ICSD/CSD | *d*(Zn-N) | Reference and formula |
|---|---|---|
| 426566 | 2.187 Å | Kysliak, O.; Beck, J. *Inorg. Chem. Commun.* **2013**, *38*, 146. $[Zn(NH_3)_6]S_6$ |
| 425027 | 2.189 Å | Kysliak, O.; Beck, J. *Eur. J. Inorg. Chem.* **2013**, 124. $[Zn(NH_3)_6][Zn(NH_3)_4]_2(TeSe_3)_3$ |
| 426394 | 2.189 Å | Kysliak, O.; Beck, J. *Z. Anorg. Allg. Chem.* **2013**, *639*, 2860. $[Zn(NH_3)_6]Sb_4S_7$ |
| 430318 | 2.192 Å | Müller, T. G.; Buchner, M. R.; Scheubeck, T. J.; Korber, N.; Kraus, F. *Z. Anorg. Allg. Chem.* **2016**, *642*, 796. $[Zn(NH_3)_6](N_3)_2\cdot 4NH_3$ |
| RAJNOF | 2.207 Å | Brumm, H.; Jansen, M. *Z. Anorg. Allg. Chem.* **2001**, *627*, 1433. $[Zn(NH_3)_6]C_{60}\cdot 6NH_3$ |
| 426401 | 2.232 Å | Kysliak, O.; Beck, J. *Z. Anorg. Allg. Chem.* **2013**, *639*, 2860. $[Zn(NH_3)_6][(NH_3)_3ZnSbS_4]\cdot 2NH_3$ |
| 248620 | 2.276 Å | Xiong, W.-W.; Miao, J.; Li, P.-Z.; Zhao, Y.; Liu, B.; Zhang, Q. *J. Solid State Chem.* **2014**, *218*, 146. $[Zn(NH_3)_6][Ag_3Zn_4Sn3Se_{13}]$ |
| **Mean** | **2.199 Å/6 structures** | |

**Cadmium(II)**

*Four-coordination, tetrahedral configuration*

| ICSD/CSD | *d*(Cd-N) | Reference and formula |
|---|---|---|
| CEKDEF | 2.289 Å | Kalinina, I. V.; Pervukhina, N. V.; Podberezskaya, N. V.; .Fedin, V. P *Russ. J. Coord. Chem.* **2002**, *29*, 389. $[Cd(NH_3)_4][(Mo_4Te_4(CN)_{12}]$ |
| **Mean** | **2.289 Å/1 structure** | |

*Six-coordination, octahedral configuration*

| ICSD/CSD | *d*(Cd-N) | Reference and formula |
|---|---|---|
| 426458 | 2.346 Å | Ledneva, A. Y.; Smolentsev, A. I.; Naumov, N. G. *J. Coord. Chem.* **2013**, *66*, 4363. $[Cd(NH_3)_6][(NH_3)_5CdOHRe_6S_8(CN)_4NH_3]_2 \cdot 5H_2O$ |
| 408602 PUHJOR | 2.372 Å | Himmel, K.; Jansen, M. *Eur. J. Inorg. Chem.* **1998**, 1183. $[Cd(NH_3)_6]C_{60} \cdot 6NH_3$ |
| BICSOW | 2.375 Å | Paul, B.; Nather, C.; Walfort, B.; Fromm, K. M.; Zimmermann, B.; Lang, H.; Janiak, C. *CrystEngComm* **2004**, *6*, 293. $[Cd(NH_3)_6]C_{20}H_{12}O_2 \cdot 2C_{20}H_{14}O_2$ |
| 424362 | 2.375 Å | Kraus, F. *Monatsh. Chem.* **2012**, *143*, 1097. $[Cd(NH_3)_6]F_2 \cdot H_2O$ |
| **Mean** | **2.367 Å/4 structures** | |

**Mercury(II)**

*Two-coordination, linear configuration*

| | | |
|---|---|---|
| 412828 | 2.074 Å | Nockemann, P.; Meyer, G. *Z. Anorg. Allg. Chem.* **2003**, *629*, 123. $[Hg(NH_3)_2][HgCl_3]_2$ |

*Four-coordination, tetrahedral configuration*

| ICSD/CSD | *d*(Cd-N) | Reference and formula |
|---|---|---|
| 412829 | 2.265 Å | Nockemann, P.; Meyer, G. *Z. Anorg. Allg. Chem.* **2003**, *629*, 123. $[Hg(NH_3)_4](ClO_4)_2$ |
| 173286 | 2.346 Å | Nilsson, K. B.; Maliarik, M.; Persson, I.; Fischer, A.; Ullström, A.-S.; Eriksson, L.; Sandström, M. *Inorg. Chem.* **2008**, *47*, 1953. $[Hg(NH_3)_4](ClO_4)_2$ |
| **Mean** | **2.306 Å/2 structures** | |

**Aluminum(III)**

*Six-coordination, octahedral configuration*

| ICSD/CSD | *d*(Cd-N) | Reference and formula |
|---|---|---|
| 203004 | 2.044 Å | Peters, K.; Bock, J.; Jacobs, H. *J. Less-Common Metal.* **1989**, *154*, 243. $[Al(NH_3)_6]I_3{\cdot}NH_3$ |
| UVIDAF | 2.049 Å | Guo, Y.; Wu, H.; Zhou, W.; Yu, X. *J. Am. Chem. Soc.* **2011**, *133*, 4690. $[Al(NH_3)_6](BH_4)_2[Li_2B_3H_{12}]$ |
| JERGOF | 2.050 Å | Tang, Z.; Tan, Y.; Wu, H.; Gu, Q.; Zhou, W.; Jensen, C. M.; Yu, X. *Acta Mater.* **2013**, *61*, 4787. $[Al(NH_3)_6](BH_4)_3$ |
| **Mean** | **2.048 Å/3 structures** | |

**Gallium(III)**

*Six-coordination, octahedral configuration*

| ICSD/CSD | *d*(Cd-N) | Reference and formula |
|---|---|---|
| 426497 | 2.081 Å | Zhang, S.; Hintze, F.; Schnick, W.; Niewa, R. *J. Less-Common Metal.* **1989**, *154*, 243. $[Ga(NH_3)_6]I_3{\cdot}NH_3$ |
| **Mean** | **2.081 Å/1 structure** | |

**Structures of ammine solvated metal ions in liquid ammonia solution**

| | *d*(M-N) | *N* | Reference and formula |
|---|---|---|---|
| Copper(I) | 2.00 Å | 3 | Nilsson, K. B.; Persson, I. *Dalton. Trans*. **2004**, 1312. |
| Silver(I) | 2.26 Å | 3 | Nilsson, K. B.; Kessler, V. G.; Persson, I. *Inorg. Chem.* **2006**, *45*, 6912. |
| Gold(I) | 2.022 Å | 2 | Nilsson, K. B.; Kessler, V. G.; Persson, I. *Inorg. Chem.* **2006**, *45*, 6912. |
| Copper(II) | 2.065+2.29 Å | 4+2 | Nilsson, K. B.; Eriksson, L.; Kessler, V. G.; Persson, I. *J. Mol. Liq.* **2007**, *131-132*, 113. |
| | 2.00 +2.19 | 4+1 | Valli, M.; Matsuo, S.; Wakita, H.; Yamaguchi, T.; Nomura, M. *Inorg. Chem.* **1996**, *35*, 5642. |
| Zinc(II) | 2.117 Å | 5 | Nilsson, K. B.; Eriksson, L.; Kessler, V. G.; Persson, I. *J. Mol. Liq.* **2007**, *131-132*, 113. |
| Cadmium(II) | 2.347 | 6 | Nilsson, K. B.; Eriksson, L.; Kessler, V. G.; Persson, I. *J. Mol. Liq.* **2007**, *131-132*, 113. |
| Mercury(II) | 2.225 | 4 | Nilsson, K. B.; Maliarik, M.; Persson, I.; Fischer, A.; Ullström, A.-S.; Eriksson, L.; Sandström, M. *Inorg. Chem.* **2008**, *47*, 1953. |

**Structures of ammine solvated metal ions in aqueous ammonia solution**

| | | | |
|---|---|---|---|
| Silver(I) | 2.15 Å | 2 | Nilsson, K. B.; Kessler, V. G.; Persson, I. *Inorg. Chem.* **2006**, *45*, 6912. |
| Gold(I) | 2.025 Å | 2 | Nilsson, K. B.; Kessler, V. G.; Persson, I. *Inorg. Chem.* **2006**, *45*, 6912. |
| Copper(II) | 2.051+2.29 Å | 4+2 | Nilsson, K. B.; Eriksson, L.; Kessler, V. G.; Persson, I. *J. Mol. Liq.* **2007**, *131-132*, 113. |
| Zinc(II) | 2.028 Å | 4 | Nilsson, K. B.; Eriksson, L.; Kessler, V. G.; Persson, I. *J. Mol. Liq.* **2007**, *131-132*, 113. |
| Cadmium(II) | 2.347 | 6 | Nilsson, K. B.; Eriksson, L.; Kessler, V. G.; Persson, I. *J. Mol. Liq.* **2007**, *131-132*, 113. |
| Mercury(II) | 2.226 | 4 | Nilsson, K. B.; Maliarik, M.; Persson, I.; Fischer, A.; Ullström, A.-S.; Eriksson, L.; Sandström, M. *Inorg. Chem.* **2008**, *47*, 1953. |

**Table S3.** Structure of *N,N'*-dimethylthioformamide solvated metal ions in the solid state and *N,N'*-dimethylthioformamide solution.

***Solid state***

**Iron(II)**

*Six-coordination, octahedral configuration*

| CSD | *d*(Fe-S) | Reference and formula |
|---|---|---|
| FAVDOV | 2.541 Å | Baumgartner, O. *Acta Crystallogr., Sect. C* **1986**, *42*, 1723. $[Fe(SHCN(CH_3)_2)_6](ClO_4)_2$ |

**Nickel(II)**

*Six-coordination, octahedral configuration*

| CSD | *d*(Ni-S) | Reference and formula |
|---|---|---|
| RUZBUJ | 2.459 Å | Kristiansson, O.; Persson, I.; Bobicz, D.; Xu, D. *Inorg. Chim. Acta* **2003**, *344*, 15. $[Ni(SHCN(CH_3)_2)_6](ClO_4)_2$ |

**Copper(I)**

*Four-coordination, tetrahedral configuration*

| CSD | *d*(Cu-S) | Reference and formula |
|---|---|---|
| MERZOY | 2.337 Å | Stålhandske, C. M. V.; Stålhandske, C. I.; Persson, I.; Sandström, M.; Jalilehvand, F. *Inorg. Chem.* **2001**, *40*, 6684. $[Cu(SHCN(CH_3)_2)_4](ClO_4)_2$ |

**Zinc(II)**

*Four-coordination, tetrahedral configuration*

| CSD | *d*(Zn-S) | Reference and formula |
|---|---|---|
| NEFXAX | 2.240 Å | Stålhandske, C. M. V.; Stålhandske, C. I.; Sandström, M.; Persson, I. *Inorg. Chem.* **1997**, *36*, 3167. $[Zn(SHCN(CH_3)_2)_4](CF_3SO_3)_2$ |

**Cadmium(II)**

*Six-coordination, octahedral configuration*

| CSD | *d*(Cd-S) | Reference and formula |
|---|---|---|
| NEFXEB | 2.714 Å | Stålhandske, C. M. V.; Stålhandske, C. I.; Sandström, M.; Persson, I. *Inorg. Chem.* **1997**, *36*, 3167. $[Cd(SHCN(CH_3)_2)_6](ClO_4)_2$ |

**Mercury(II)**

*Two-coordination, linear configuration*

| CSD | *d*(Hg-S) | Reference and formula |
|---|---|---|
| NEFXOL | 2.351 Å | Stålhandske, C. M. V.; Stålhandske, C. I.; Sandström, M.; Persson, I. *Inorg. Chem.* **1997**, *36*, 3167. $[Hg(SHCN(CH_3)_2)_2](ClO_4)_2$ |

***DMTF solution***

**Iron(III)**

*Four-coordination, tetrahedral configuration*

| *d*(Fe-S) | Reference and formula |
|---|---|
| 2.206 Å | Lundberg, D.; Ullström, A.-S.; D'Angelo, P.; I. Persson, I. *Inorg. Chim. Acta* 2007, **360**, 1809. $[Fe(SHCN(CH_3)_2)_6]^{3+}$ |

**Nickel(II)**

*Six-coordination, octahedral configuration*

| *d*(Ni-S) | Reference and formula |
|---|---|
| 2.459 Å | Kristiansson, O.; Persson, I.; Bobicz, D.; Xu, D. *Inorg. Chim. Acta* **2003**, *344*, 15. $[Ni(SHCN(CH_3)_2)_6]^{2+}$ |

**Copper(I)**

*Four-coordination, tetrahedral configuration*

| *d*(Cu-S) | Reference and formula |
|---|---|
| 2.36 Å | Stålhandske, C. M. V.; Stålhandske, C. I.; Persson, I.; Sandström, M.; Jalilehvand, F. *Inorg. Chem.* **2001**, *40*, 6684. $[Cu(SHCN(CH_3)_2)_4]^+$ |

**Silver(I)**

*Four-coordination, tetrahedral configuration*

| *d*(Ag-S) | Reference and formula |
|---|---|
| 2.48 Å | Stålhandske, C. M. V.; Stålhandske, C. I.; Persson, I.; Sandström, M.; Jalilehvand, F. *Inorg. Chem.* **2001**, *40*, 6684. $[Ag(SHCN(CH_3)_2)_4]^+$ |

**Gold(I)**

*Two-coordination, linear configuration*

| *d*(Cu-S) | Reference and formula |
|---|---|
| 2.290 Å | Stålhandske, C. M. V.; Stålhandske, C. I.; Persson, I.; Sandström, M.; Jalilehvand, F. *Inorg. Chem.* **2001**, *40*, 6684. $[Cu(SHCN(CH_3)_2)_4]^{+}$ |

**Zinc(II)**

*Four-coordination, tetrahedral configuration*

| *d*(Zn-S) | Reference and formula |
|---|---|
| 2.362 Å | Stålhandske, C. M. V.; Persson, I.; Sandström, M.; Kamienska-Piotrowicz, E. *Inorg. Chem.* **1997**, *36*, 3174. $[Zn(SHCN(CH_3)_2)_4]^{2+}$ |

**Cadmium(II)**

*Six-coordination, octahedral configuration*

| *d*(Zn-S) | Reference and formula |
|---|---|
| 2.69 Å | Stålhandske, C. M. V.; Persson, I.; Sandström, M.; Kamienska-Piotrowicz, E. *Inorg. Chem.* **1997**, *36*, 3174. $[Zn(SHCN(CH_3)_2)_4]^{2+}$ |

**Mercury(II)**

*Two-coordination, linear configuration*

| *d*(Hg-S) | Reference and formula |
|---|---|
| 2.362 Å | Stålhandske, C. M. V.; Persson, I.; Sandström, M.; Kamienska-Piotrowicz, E. *Inorg. Chem.* **1997**, *36*, 3174. $[Hg(SHCN(CH_3)_2)_2]^{2+}$ |

**Gallium(III)**

*Four-coordination, tetrahedral configuration*

| *d*(Ga-S) | Reference and formula |
|---|---|
| 2.333 Å | Ö. Topel, I. Persson, D. Lundberg and A.-S. Ullström, A.-S. *Inorg. Chim. Acta* 2010, **363**. 988. $[Ga(SHCN(CH_3)_2)_4]^{3+}$ |

**Bismuth(III)**

*Six-coordination, octahedral configuration*

| *d*(Bi-S) | Reference and formula |
|---|---|
| 2.794 Å | Näslund, J.; Persson, I.; Sandström, M. *Inorg. Chim. Acta* 2000, **39**, 4012. $[Bi(SHCN(CH_3)_2)_6]^{3+}$ |

**Table S4.** Summary of structures of homoleptic copper(I), silver(I) and gold(I) complexes with phosphorus donor ligands in the solid state and solution. Citations with purple colour are not included the mean M-P bond distances.

**Phosphorus donor solvents**

***Phoshite solvents***

*Trimethylphosphite*

**Copper(I)**

*Four-coordination, tetrahedral configuration*

| CSD code | *d*(Cu-P) | Reference, compound formula |
|---|---|---|
| SIYSIC | 2.242 Å | Fuchs, R.; Klufers, P. *Z. Naturforsch., Sect B* **1991**, *46*, 507. $[Cu(P(OCH_3)_3)_4][Cu(Co(CO)_4)_2]$ |

***Phosphine solvents***

**Copper(I)**

*Two-coordination, linear configuration*

| CSD code | *d*(Cu-P) | Reference, compound formula |
|---|---|---|
| *Tri(iso-propyl)phosphine* | | |
| GOXLOX | 2.206 Å | Jiang, T.; Zhang, K.; Shen, Y.; Hamdaoui, M.; Dontha, R.; Liu, J.; Spingler, B.; Duttwyler, S. *Dalton Trans.* **2019**, *48*, 17192. $[Cu(P(CH(CH_3)_2)_2](C_3H_{12}B_{11})$ |
| *Tri(t-butyl)phosphine* | | |
| LACNIP | 2.212 Å | Oeschger, R.; Chen, P.; Trapp, N. CCDC deposition number 1453515, **2016**. $[Cu(P(C(CH_3)_3)_2]CF_3SO_3{\cdot}2C_4H_8O$ |
| NOGZER | 2.218 Å | Ho, C. C.; Ariafard, A.; Hyland, C. J. T; Bissember, A. C. *Organometallics* **2019**, *38*, 2683. $[Cu(P(C(CH_3)_3)_2]PF_6{\cdot}2C_4H_8O$ |
| *Tris(benzyl)phosphine* | | |
| BALMOQ | 2.190 Å | Ainscough, E. W.; Brodie, A. M.; Burrell, A. K.; Hanna, J. V.; Healy, P. C.; Waters , J. M. *Inorg. Chem.* **1999**, *38*, 201. $[Cu(P(CH_2C_6H_5)_3)_2]PF_6$ |
| TETJAD | 2.196 Å | Akrivos, P. D.; Karagiannidis, P. P.; Raptopoulou, C. P.; Terzis, A.; Stoyanov, S. *Inorg. Chem.* **1996**, *35*, 4082. $[Cu(P(CH_2C_6H_5)_3)_2]$ $[CuBr_2]$ |
| QEDHEM | 2.205 Å | Ainscough, E. W. ; Brodie, A. M.; Burrell, A. K.; Freeman, G. H.; Jameson, G. B.; Bowmaker, G. A.; Hanna, J. V.; Healy, P. C. *J. Chem. Soc., Dalton Trans.* **2001**, 144. $[Cu(P(CH_2C_6H_5)_3)_2][CuCl_2]$ |

**Mean 2.197 Å/3 structures**

**Mean all 2.205 Å/6 structures**

*Four-coordination, tetrahedral configuration*

| CSD code | *d*(Cu-P) | Reference, compound formula |
|---|---|---|
| *Trimethylphosphine* | | |
| WADFOW | 2.251 Å | Eichhofer, A.; Fenske, D.; Holstein, W. *Angew. Chem., Int. Ed.* **1993**, *32*, 242. $[Cu(P(CH_3)_3)_4]_2[Cu_{13}C_{18}H_{54}Cl_6P_9]$ |
| PIJTOS | 2.263 Å | Schneider, S.; Dzudza, A.; Raudaschl-Sieber, G.; Marks, T. J. *Chem. Mater.* **2007**, *19*, 2768. $[Cu(P(CH_3)_3)_4][Cu_5C_{12}H_{36}S]\cdot 2C_4H_8O$ |
| MUQHEL | 2.264 Å | Patow, R. ; Fenske, D. *Z. Anorg. Allg. Chem.* **2002**, *628*, 1279. $[Cu(P(CH_3)_3)_4]_3[CuTa_6C_3H_9PS_{17}]\cdot 2CH_3CN$ |
| GAXSUT | 2.265 Å | Dempsey, D. F.; Girolami, G. S. *Organometallics* **2019**, *38*, 2683. $[Cu(P(CH_3)_3)_4][Cu(CH_3)_2]$ |
| VIVVON | 2.265 Å | Kischel, M.; Dornberg, G.; Krautscheid, H. *Inorg. Chem.* **2014**, *53*, 1614. $[Cu(P(CH_3)_3)_4][(CH_3)_2Al(SC_6H_5)_2]$ |
| MOMJON | 2.266 Å | Patow, R. ; Fenske, D. *Z. Anorg. Allg. Chem.* **2002**, *628*, 1279. $[Cu(P(CH_3)_3)_4]_2[Cu_6Ta_2C_{24}H_{54}N_6P_6S_6Se_6]\cdot 2(CH_3)_2NCHO$ |
| QEGFIT | 2.266 Å | Kluge, O.; Krautscheid, H. *Inorg. Chem.* **2012**, *51*, 6655. $[Cu(P(CH_3)_3)_4][(CH_3)_2Ga(SC_6H_5)_2]$ |
| QEGFUF | 2.266 Å | Kluge, O.; Krautscheid, H. *Inorg. Chem.* **2012**, *51*, 6655. $[Cu(P(CH_3)_3)_4][CH_3Ga(SC_6H_5)_3]\cdot 3CH_3OH$ |
| KOTLOW | 2.267 Å | Kociok-Kohn, G.; Mahon, M. F.; Molloy, K. C.; Sudlow, A. L. *Main Group Met. Chem.* **2014**, *37*, 11. $[Cu(P(CH_3)_3)_4][(CH_3)_3PSbCl_4SbCl_4(P(CH_3)_3]$ |
| KURTIA | 2.267 Å | Chi, K.-M.; Farkas, J.; Hampden-Smith, M. J.; Kodas, T. T.; Duesler, E. N. *J. Chem. Soc., Dalton Trans.* **1992**, 3111. $[Cu(P(CH_3)_3)_4][CuCl_2]$ |
| KURTIA01 | 2.267 Å | Margraf, G.; Lerner, H.-W.; Wagner, M.; Bolte, M. *Acta Crystallogr., Sect. E* **2004**, *60*, m186. $[Cu(P(CH_3)_3)_4][CuCl_2]$ |
| QEGFOZ | 2.267 Å | Kluge, O.; Krautscheid, H. *Inorg. Chem.* **2012**, *51*, 6655. $[Cu(P(CH_3)_3)_4][(CH_3)_2Ga(SeC_6H_5)_2]$ |
| RAHZOS | 2.267 Å | Kluge, O.; Krautscheid, H. CCDC deposition number 907038, **2016**. $[Cu(P(CH_3)_3)_4][CH_3)_2Ga(S_2C_2H_4)]$ |
| KOTKIP | 2.270 Å | Kociok-Kohn, G.; Mahon, M. F.; Molloy, K. C.; Sudlow, A. L. *Main Group Met. Chem.* **2014**, *37*, 11. $[Cu(P(CH_3)_3)_4][Cl_2ZnCl_2Cu(P(CH_3)_3)_2]$ |
| KURTOG | 2.270 Å | Chi, K.-M.; Farkas, J.; Hampden-Smith, M. J.; Kodas, T. T.; Duesler, E. N. *J. Chem. Soc., Dalton Trans.* **1992**, 3111. $[Cu(P(CH_3)_3)_4][CF_3COCHCOCF_3]$ |
| KIDFEI | 2.271 Å | Bowmaker, G. A.; Healy, P. C.; Engelhardt, L. M.; Kildea, J. D.; Skelton, B. W.; White, A. H. *Aust. J. Chem.* **1990**, *43*, 1697. $[Cu(P(CH_3)_3)_4]Cl$ |
| KIDFIM | 2.271 Å | Bowmaker, G. A.; Healy, P. C.; Engelhardt, L. M.; Kildea, J. D.; Skelton, B. W.; White, A. H. *Aust. J. Chem.* **1990**, *43*, 1697. $[Cu(P(CH_3)_3)_4]Br\cdot C_6H_6$ |

| | | |
|---|---|---|
| VIVTEB | 2.271 Å | Kischel, M.; Dornberg, G.; Krautscheid, H. *Inorg. Chem.* **2014**, *53*, 1614. $[Cu(P(CH_3)_3)_4][Cu_5C_{15}H_{39}P_3S_6]\cdot 1.5C_4H_8O$ |
| GOFYAE | 2.272 Å | Zhang, K.; Shen, Y.; Yang, X.; Liu, J.; Jiang, T.; Finney, N.; Spingler, B.; Duttwyler, S. *Chem. – Eur. J.* **2019**, *25*, 8754. $[Cu(P(CH_3)_3)_4]_2[CuC_{27}H_{89}B_{44}P_5]$ |
| VIVWAA | 2.272 Å | Kischel, M.; Dornberg, G.; Krautscheid, H. *Inorg. Chem.* **2014**, *53*, 1614. $[Cu(P(CH_3)_3)_4][(CH(CH_3)_2)_2Al(SC_6H_5)_2]$ |
| MOMJAZ | 2.274 Å | Patow, R. ; Fenske, D. *Z. Anorg. Allg. Chem.* **2002**, *628*, 1279. $[Cu(P(CH_3)_3)_4]_2[Cu_6Ta_2C_{24}H_{54}N_6P_6S_{12}]\cdot 4CH_3CN$ |
| QEGGAM | 2.276 Å | Kluge, O.; Krautscheid, H. *Inorg. Chem.* **2012**, *51*, 6655. $[Cu(P(CH_3)_3)_4][CH_3Ga(SeC_6H_5)_3]\cdot 4CH_3OH$ |
| RAHZIM | 2.276 Å | Kluge, O.; Krautscheid, H. CCDC deposition number 907039, **2016**. $[Cu(P(CH_3)_3)_4][CH_3)_2In(S_2C_2H_4)]$ |
| PECFIQ | 2.277 Å | Liebing, P.; Merzweiler, K. *Z. Anorg. Allg. Chem.* **2022**, *648*, e02100339. $[Cu(P(CH_3)_3)_4][Cu_5C_{36}H_{52}N_4P_2S_4Si_2]$ |
| KIDFOS | 2.278 Å | Bowmaker, G. A.; Healy, P. C.; Engelhardt, L. M.; Kildea, J. D.; Skelton, B. W.; White, A. H. *Aust. J. Chem.* **1990**, *43*, 1697. $[Cu(P(CH_3)_3)_4]I\cdot C_6H_6$ |
| QEGGEQ | 2.281 Å | Kluge, O.; Krautscheid, H. *Inorg. Chem.* **2012**, *51*, 6655. $[Cu(P(CH_3)_3)_4][CH_3Ga(TeC_6H_5)_3]$ |
| JASTON | 2.282 Å | Wagner, M.; Margraf, G.; Lerner, H.-W.; Bolte, M. CCDC deposition number 199144, 2005. . $[Cu(P(CH_3)_3)_4]Br$ |
| **Mean** | **2.270 Å/27 structures** | |

*Phosphinetriyl)triethanol*

| | | |
|---|---|---|
| NENFER | 2.281 Å | Bharathi, D. S.; Sridhar, M. A.; Prasad, J. S.; Samuelson, A. G. *Inorg. Chem. Commun.* **2001**, *4*, 490. $[Cu(P(C_2H_4OH)_3)P(C_2H_4O)]$ |
| **Mean all** | **2.270 Å/28 structures** | |

**Silver(I)**

*Two-coordination, linear configuration*

| CSD code | *d*(Ag-P) | Reference, compound formula |
|---|---|---|
| *Trimethylphosphine* | | |
| MOPCAV | 2.377 Å | Alyea, E. C.; Kannan, S.; Meehan, P. R. *Acta Crystallogr., Sect. C* **2002**, *58*, m365. $[Ag(P(CH_3)_3)_2]PF_6$ |

*Tri(tert-butyl)phosphine*

| | | |
|---|---|---|
| UHAPAW | 2.394 Å | Zopes, D.; Hegemann, C.; Schlafer, J.; Tyrra, W.; Mathur, S. *Inorg. Chem.* **2015**, *54*, 3781. $[Ag(P(C(CH_3)_3)_2][Au(CF_3)_2]$ |
| GEQHOB | 2.397 Å | Higelin, A.; Krossing, I. CCDC deposition number 912619, **2012**. $[Ag(P(C(CH_3)_3)_2][Al(OC(CF_3)_3)_4]$ |
| GEQHOB01 | 2.405 Å | Bolte, M. CCDC deposition number 1027889, **2014**. $[Ag(P(C(CH_3)_3)_2][Al(OC(CF_3)_3)_4]$ |
| **Mean** | **2.393 Å/3 structures** | |

*Tricyclohexylphosphine*

| | | |
|---|---|---|
| LUTDUA | 2.376 Å | Altaf, M.; Stoeckli-Evans, H. *Polyhedron* **2010**, *29*, 701. $[Ag(P(C_6H_{11})_3)_2]SbF_6$ |
| LUTDIO | 2.379 Å | Altaf, M.; Stoeckli-Evans, H. *Polyhedron* **2010**, *29*, 701. $[Ag(P(C_6H_{11})_3)_2]PF_6$ |
| KUVVON01 | 2.382 Å | Fraga, J.; Schmalle, H. W.; Berke, H. CCDC deposition number 621764, **2016**. $[Ag(P(C_6H_{11})_3)_2][AgSO_3CF_3(P(C_6H_{11})_3)_2]$ |
| QIQGUW | 2.386 Å | Demonti, L.; Tabikh, H.; Saffon-Merceron, N.; Nebra, N. *Eur. J. Inorg. Chem.* **2023**, *26*, e202300042. $[Ag(P(C_6H_{11})_3)_2][Ag(CF_3)_4]$ |
| KUVVON | 2.395 Å | Partyka, D. V.; Deligonul, N. *Inorg. Chem.* **2009**, *48*, 9463. $[Ag(P(C_6H_{11})_3)_2]CF_3SO_3$ |
| QAZKAG | 2.403 Å | Blacque, O.; Fraga-Hernandez, J.; Berke, H. CCDC deposition number 1502022, **2017**. $[Ag(P(C_6H_{11})_3)_2][W(NO)_2(NCCH_3)Cl(OSO_2CF_3)_2]$ |
| **Mean** | **2.387 Å/6 structrures** | |

*Tris(2-Cyanoethyl)phosphine*

| | | |
|---|---|---|
| TILTAJ | 2.383 Å | Liu, C. W.; Pan, H.; Fackler Jr., J. P.; Wu, G.; Wasylishen, R. E. ; Shang, M. *J. Chem. Soc., Dalton Trans.* **1995**, 3691. $[Ag(P(C_2H_4CN)_3)_2]NO_3$ |

*Tris(benzyl)phosphine*

| | | |
|---|---|---|
| EPUDEZ | 2.374 Å | Ainscough, E. W.; Brodie, A. M.; Burrell, A. K.; Hanna, J. V.; Healy, P. C.; Waters , J. M. *Inorg. Chem.* **1999**, *38*, 201. $[Ag(P(CH_2C_6H_5)_3)_2]PF_6$ |
| EPUDID | 2.376 Å | Ainscough, E. W.; Brodie, A. M.; Burrell, A. K.; Hanna, J. V.; Healy, P. C.; Waters , J. M. *Inorg. Chem.* **1999**, *38*, 201. $[Ag(P(CH_2C_6H_5)_3)_2]BF_4$ |
| EPUCUO | 2.381 Å | Ainscough, E. W.; Brodie, A. M.; Burrell, A. K.; Hanna, J. V.; Healy, P. C.; Waters , J. M. *Inorg. Chem.* **1999**, *38*, 201. $[Ag(P(CH_2C_6H_5)_3)_2]_8[AgCl_3]Cl_6\cdot 6H_2O$ |
| EPUDAV | 2.403 Å | Ainscough, E. W.; Brodie, A. M.; Burrell, A. K.; Hanna, J. V.; Healy, P. C.; Waters , J. M. *Inorg. Chem.* **1999**, *38*, 201. $[Ag(P(CH_2C_6H_5)_3)_2]_2[AgBr_3]\cdot 1.38H_2O$ |
| **Mean** | **2.384 Å/4 structures** | |

| | | |
|---|---|---|
| **Mean all** | **2.386 Å/15 structures** | |

*Four-coordination, tetrahedral configuration*

| CSD code | *d*(Ag-P) | Reference, compound formula |
|---|---|---|
| *Phosphinetriyl)trimethanol* | | |
| UBOPUZ | 2.486 Å | Tapanelli, S.; Habluetzel, A.; Pellei, M.; Marchio, L.; Tombesi, A.; Cappare, A.; Santini, C. *J. Inorg. Biochem.* **2017**, *166*, 1. $[Ag(P(CH_2OH)_3)_4]PF_6{\cdot}H_2O$ |
| GEQHOB02 | 2.603 Å | Schwab, M. M.; Himmel, D.; Kacprzak, S.; Radtke, V.; Kratzert, D.; Weis, P.; Wernet, M.; Peter, A.; Yassine, Z.; Schmitz, D.; Scheidt, E.-W.; Scherer, W.; Weber, S.; Feuerstein, W.; Breher, F.; Higelin, A.; Krossing, I. *Chem. – Eur. J.* **2017**, *24*, 918. $[Ag(P(C(CH_3)_3)_2][Al(OC(CF_3)_3)_4]$ |
| **Mean** | **2.603 Å/1 structure** | |

**Gold(I)**

*Two-coordination, linear configuration*

| CSD code | *d*(Au-O) | Reference, compound formula |
|---|---|---|
| *Trimethylphosphine* | | |
| QAKCEN | 2.301 Å | Lasanta, T.; Lopez-de-Luzuriaga, J. M.; Monge, M.; Olmos, M. E.; Pascual, D. *Dalton Trans.* **2016**, *45*, 6334. $[Au(P(CH_3)_3)_2][AuHg_5C_{36}F_{24}]$ |
| KIMXUD | 2.301 Å | Zapf, L.; Radius, U.; Finze, M. *Dalton Trans.* **2023**, *52*, 9553. $[Au(P(CH_3)_3)_2][Au(C_{10}H_5F_{17}N_2P)_2]$ |
| JEVYEP01 | 2.302 Å | Khairul, W. M.; Fox, M. A.; Zaitseva, N. N.; Gaudio, M.; Yufit, D. S.; Skelton, B. W.; White, A. H.; Howard, J. A. K; Bruce, M. I.; Low, P. J. *Dalton Trans.* **2009**, 610. $[Au(P(CH_3)_3)_2]PF_6$ |
| JEVYEP | 2.303 Å | Horvath, U. E. I.; Raubenheimer, H. G. *Acta Crystallogr., Sect. E* **2007**, *63*, m567. $[Au(P(CH_3)_3)_2]PF_6$ |
| LEXJAZ | 2.304 Å | Angermaier, K.; Zeller, E.; Schmidbaur, H. *J. Organometal. Chem.* **1994**, *472*, 371. $[Au(P(CH_3)_3)_2]Cl{\cdot}2CHCl_3$ |
| **Mean** | **2.302 Å/5 structures** | |

| | | |
|---|---|---|
| *Triethylphosphine* | | |
| TOVWOQ | 2.295 Å | Hanna, S. D.; Khan, S. I.; Zink, J. I. *Inorg. Chem.* **1996**, *35*, 5813. $[Au(P(C_2H_5)_3)_2][Au_3C_{20}H_{30}N_4P_2S_4]$ |
| LASVEG | 2.297 Å | Nakamoto, M.; Koijman, H.; Paul, M.; Hiller, W.; Schmidbaur, H. *Z. Anorg. Allg. Chem.* **1993**, *619*, 1341. $[Au(P(C_2H_5)_3)_2][Au(S_2C_6H_4)_2]$ |
| JOTLAI | 2.300 Å | Glodek, M.; Pawledzio, S.; Makal, A.; Plazuk, D. *Chem. – Eur. J.* **2019**, *25*, 13131. $[Au(P(C_2H_5)_3)_2][Au(C_2COC_6H_5)_2]$ |

| | | |
|---|---|---|
| JOTJIO | 2.305 Å | Glodek, M.; Pawledzio, S.; Makal, A.; Plazuk, D. *Chem. – Eur. J.* **2019**, *25*, 13131. $[Au(P(C_2H_5)_3)_2][Au(C_4COC_6H_4OCH_3)_2]$ |
| NIBMOD02 | 2.306 Å | Jastrzebska, R.; Poreba, T.; Cova, F.; Tchon, D. M.; Makal, A. *IUCrJ* **2024**, *11*, 737. $[Au(P(C_2H_5)_3)_2][Au(C_2COC_{16}H_9)_2]$ |
| JOTKUB | 2.308 Å | Glodek, M.; Pawledzio, S.; Makal, A.; Plazuk, D. *Chem. – Eur. J.* **2019**, *25*, 13131. $[Au(P(C_2H_5)_3)_2][Au(C_2COC_6H_5)_2]$ |
| NIBMOD03 | 2.309 Å | Jastrzebska, R.; Poreba, T.; Cova, F.; Tchon, D. M.; Makal, A. *IUCrJ* **2024**, *11*, 737. $[Au(P(C_2H_5)_3)_2][Au(CCCOC_{16}H_8)_2]$ |
| JOTJOU | 2.311 Å | Glodek, M.; Pawledzio, S.; Makal, A.; Plazuk, D. *Chem. – Eur. J.* **2019**, *25*, 13131. $[Au(P(C_2H_5)_3)_2][Au(C_4COC_6H_5)_2]$ |
| NIBMOD | 2.312 Å | Glodek, M.; Makal, A.; Paluch, P.; Kadziolka-Gawel, M.; Kobayashi, Y.; Zakrzewski, J.; Plazuk, D. *Dalton Trans.* **2018**, *47*, 6702. $[Au(P(C_2H_5)_3)_2][Au(C_2COC_{16}H_9)_2]$ |
| LASVEG01 | 2.313 Å | Davila, R. M.; Staples, R. P.; Fackler, Jr., J. P. *Z. Anorg. Allg. Chem.* **1993**, *619*, 1341. $[Au(P(C_2H_5)_3)_2][Au(S_2C_6H_4)_2]$ |
| NIBLUI | 2.313 Å | Glodek, M.; Makal, A.; Paluch, P.; Kadziolka-Gawel, M.; Kobayashi, Y.; Zakrzewski, J.; Plazuk, D. *Dalton Trans.* **2018**, *47*, 6702. $[Au(P(C_2H_5)_3)_2][Au(C_2COC_5H_5FeC_5H_5)_2]$ |
| JOTKEL | 2.314 Å | Glodek, M.; Pawledzio, S.; Makal, A.; Plazuk, D. *Chem. – Eur. J.* **2019**, *25*, 13131. $[Au(P(C_2H_5)_3)_2][Au(C_2COC_6H_2F_3)_2]$ |
| JOTKIP | 2.318 Å | Glodek, M.; Pawledzio, S.; Makal, A.; Plazuk, D. *Chem. – Eur. J.* **2019**, *25*, 13131. $[Au(P(C_2H_5)_3)_2][Au(C_2COC_6H_4OC_3)_2]$ |
| JOTKAH | 2.319 Å | Glodek, M.; Pawledzio, S.; Makal, A.; Plazuk, D. *Chem. – Eur. J.* **2019**, *25*, 13131. $[Au(P(C_2H_5)_3)_2][Au(C_4COC_6H_2F_3)_2]$ |
| **Mean** | **2.309 Å/14 structures** | |

*Tri-iso-propylphosphine*

| | | |
|---|---|---|
| TEQNEM | 2.315 Å | D.Fuhrmann, H.Krautscheid, H. *A. Anorg. Allg. Chem.* **2022**, *648*, e202200099. $[Au(P(CH(CH_3)_2)_3)_2][(CH_3)_2ClSnCl(S)SnCl(CH_3)_2]$ |

*Tri-n-butylphosphine*

| | | |
|---|---|---|
| HATPUN | 2.308 Å | Staples, R. J.; Fackler, Jr., J. P; Khan, M. N. I.; Winpenny, R. E. P. *Acta Crystallogr., Sect. C* **1994**, *50*, 191. $[Au(P(C_4H_9)_3)_2]B(C_6H_5)_4$ |

*Tri-tert-butylphosphine*

| | | |
|---|---|---|
| ZAJZIT01 | 2.307 Å | Marsch, R. E. *Acta Crystallogr., Sect. B* **2002**, *58*, 893. $[Au(P(C(CH_3)_3))_2]BF_4{\cdot}2CHCl_3$ |
| ZAJZIT | 2.310 Å | Sladek, A.; Schmidbauer, H. *Z. Naturforsch., Sect. B* **1995**, *50*, 859. $[Au(P(C(CH_3)_3))_2]BF_4{\cdot}2CHCl_3$ |
| WILZIA01 | 2.319 Å | Touil, M.; Bechem, B.; Hashmi, A. S. K.; Engels, B.; Omary, M. A.; Rabaa, H. *J. Mol. Struct.* **2010**, *957*, 21. $[Au(P(C(CH_3)_3))_2]Cl$ |
| SAQVAI | 2.321 Å | Schneider, D.; Schuster, O.; Schmidbaur, H. *Dalton Trans.* **2005**, 1940. $[Au(P(C(CH_3)_3))_2]Br_3{\cdot}Br_2$ |
| WILZIA | 2.322 Å | Zeller, E.; Schier, A.; Schmidbaur, H. *Z. Naturforsch., Sect. B* **1994**, *49*, 1243. $[Au(P(C(CH_3)_3))_2]Cl$ |
| NOGZAN | 2.333 Å | Ho, C. C.; Ariafard, A.; Hyland, C. J. T; Bissember, A. C. *Organometallics* **2019**, *38*, 2683. $[Au(P(C(CH_3)_3))_2]N(SO_2CF_3)_2$ |
| **Mean** | **2.319 Å/6 structures** | |

*Tricyclohexylphosphine*

| | | |
|---|---|---|
| ZEMPEM | 2.311 Å | Staples, R. J.; Fackler, Jr., J. P. *Z. Krist. Cryst. Mater.* **1995**, *210*, 696. $[Au(P(C_6H_{11})_3)_2]_2[Au(S_2C_2(CN)_2)_2]$ |
| FIVLEB | 2.312 Å | A.J.Wynd, A.J.Welch, A. J. Chem. Commun. 1987, 1174. $[Au(P(C_6H_{11})_3)_2]_2[AuB_{20}H_{25}]{\cdot}4CH_2Cl_2$ |
| FAXHER | 2.313 Å | Bowmaker, G. A.; Brown, C. L.; Hart, R. D.; Healy, P. C.; Rickard, C. E. F.; White, A. H. *J. Chem. Soc., Dalton Trans.* **1999**, 881. $[Au(P(C_6H_{11})_3)_2]I{\cdot}P(C_6H_{11})_3$ |
| FAQBEF | 2.314 Å | Anandhi, U.; Sharp, P. R. *Angew. Chem., Int. Ed.* **2004**, *43*, 6128. $[Au(P(C_6H_{11})_3)_2][GaI_3(C_{10}H_{15})]$ |
| FAXCAI01 | 2.316 Å | Bowmaker, G. A.; Brown, C. L.; Hart, R. D.; Healy, P. C.; Rickard, C. E. F.; White, A. H. *J. Chem. Soc., Dalton Trans.* **1999**, 881. $[Au(P(C_6H_{11})_3)_2]Br$ |
| KARCAI | 2.316 Å | N.W.Alcock, M.G.H.Wallbridge, M.Jasztal, M. CCDC deposition number 271188, **2005**. $[Au(P(C_6H_{11})_3)_2]_2[Au(CB_8H_{12})_2]{\cdot}2CH_2Cl_2$ |
| BOPLOH01 | 2.318 Å | Bowmaker, G. A.; Brown, C. L.; Hart, R. D.; Healy, P. C.; Rickard, C. E. F.; White, A. H. *J. Chem. Soc., Dalton Trans.* **1999**, 881. $[Au(P(C_6H_{11})_3)_2]Cl$ |
| BOPLOH10 | 2.321 Å | J.A.Muir, M.M.Muir, L.B.Pulgar, P.G.Jones, G.M.Sheldrick, G. M. *Acta Crystallogr., Sect. C* **1985**, *41*, 1174. $[Au(P(C_6H_{11})_3)_2]Cl$ |
| **Mean** | **2.315 Å/8 structures** | |

*Tris(2-Cyanoethyl)phosphine*

| | | |
|---|---|---|
| PERFEX | 2.314 Å | Khan, Md. N. I.; King, C.; Fackler, Jr., J. P.; Winpenny, R. E. W. *Inorg. Chem.* **1993**, *32*, 2502. $[Au(P(C_2H_4CN)_3)_2]Cl$ |

| | | |
|---|---|---|
| *Tris(benzyl)phosphine* | | |
| KAFZOI | 2.298 Å | Ainscough, E. W.; Bowmaker, G. A.; Brodie, A. M.; Freeman, G. H.; Hanna, J. V.; Healey, P. C.; Robinson, W. T.; Skelton, B. W.; Smith, M. E.; Sobolev, A. N.; White, A. H. *Eur. J. Inorg. Chem.* **2010**, 2044. $[Au(P(CH_2C_6H_5)_3)_2]BF_4$ |
| KAFZIC | 2.301 Å | Ainscough, E. W.; Bowmaker, G. A.; Brodie, A. M.; Freeman, G. H.; Hanna, J. V.; Healey, P. C.; Robinson, W. T.; Skelton, B. W.; Smith, M. E.; Sobolev, A. N.; White, A. H. *Eur. J. Inorg. Chem.* **2010**, 2044. $[Au(P(CH_2C_6H_5)_3)_2]Cl\cdot H_2O$ |
| **Mean** | **2.300 Å/2 structures** | |
| **Mean all** | **2.311 Å/38 structures** | |

*Four-coordination, tetrahedral configuration*

| CSD code | *d*(Au-P) | Reference, compound formula |
|---|---|---|
| *Trimethylphosphine* | | |
| FESLUM | 2.388 Å | Malberg, J.; Wiegand, T.; Eckert, H.; Bodensteiner, M.; Wolf, R. *Chem. – Eur. J.* **2013**, *19*, 2356. $[Au(P(CH_3)_3)_4][AuCo_2C_{88}H_{120}P_8]\cdot 2C_4H_8O$ |

**Triphenylphosphine**
**Copper(I)**
*Three-coordination, triangular configuration*

| CSD code | *d*(Cu-P) | Reference, compound formula |
|---|---|---|
| LIVNUC | 2.270 Å | Lee, G. M.; Bowes, E. G.; Vogels, C. M.; Decken, A.; Westcott, S. A. *Tetrahedron* **2019**, *75*, 2106. $[Cu(P(C_6H_5)_3)_3](B(O_2C_6H_3ONO_2)_2)$ |
| GOFYUY | 2.276 Å | Zhang, K.; Shen, Y.; Yang, X.; Liu, J.; Jiang, T.; Finney, N.; Spingler, B.; Duttwyler, S. *Chem. – Eur. J.* **2019**, *25*, 8754. $[Cu(P(C_6H_5)_3)_3][Cu_3C_{78}H_{82}B_{22}P_4]$ |
| EDICOL | 2.277 Å | Eckenhoff, W. T.; Biernesser, A. B.; Pintauer, T. *Inorg. Chem.* **2012**, *51*, 11917. $[Cu(P(C_6H_5)_3)_3]B(C_6H_5)_4$ |
| GOXLIR | 2.278 Å | Jiang, T.; Zhang, K.; Shen, Y.; Hamdaoui, M.; Dontha, R.; Liu, J.; Spingler, B.; Duttwyler, S. *Dalton Trans.* **2019**, *48*, 17192. $[Cu(P(C_6H_5)_3)_3]C_3H_{12}B_{11}$ |
| OKAXOP | 2.280 Å | Balagurova, E. V.; Pisareva, I. V.; Godovikov, I. A.; Smol'yakov, A. F.; Dolgushin, F. M.; Chizhevsky, I. T. *Izv. Akad. Nauk SSSR, Ser.Khim.* **2013**, 548. $[Cu(P(C_6H_5)_3)_3][CoC_4H_{22}B_{18}]\cdot 2CH_2Cl_2$ |
| SUXCIX | 2.286 Å | Li, D.; Li, R.; Qi, Z.; Feng, X.; Cai, J.; Shi, X. *Inorg. Chem. Commun.* **2001**, *4*, 483. $[Cu(P(C_6H_5)_3)_3][Cu_4C_{90}H_{72}N_9P_4](BF_4)_2$ |
| SUXCIX | 2.286 Å | Li, D.; Li, R.; Qi, Z.; Feng, X.; Cai, J.; Shi, X. *Inorg. Chem. Commun.* **2001**, *4*, 483. $[Cu(P(C_6H_5)_3)_3][Cu_4C_{90}H_{72}N_9P_4](BF_4)_2$ |
| WIMLEK | 2.287 Å | Du, S.; Hodson, D. E.; Lei, P.; McGrath, T. D.; Stone, F. G. A. *Inorg. Chem.* **2007**, *46*, 6613. $[Cu(P(C_6H_5)_3)_3][Ru_6C_{16}H_9B_8O_{15}]\cdot 2CH_2Cl_2$ |
| SOTSID | 2.292 Å | Klufers, P.; Wilhelm, U. *J. Organometal. Chem.* **1991**, *421*, 39. $[Cu(P(C_6H_5)_3)_3][(CO)_5CrHCr(CO)_5]$ |
| DEZYUC | 2.295 Å | Doyle, G.; Eriksen, K. A.; Van Engen, D. *Organometallics* **1985**, *4*, 2201. $[Cu(P(C_6H_5)_3)_3][V(CO)_6]$ |
| ZEPYEY | 2.296 Å | Davidson, J. L.; Lindsell, W. E.; McCullough, K. J.; McIntosh, C. H. *Organometallics* **1995**, *14*, 3497. $[Cu(P(C_6H_5)_3)_3][Mo(SC_6F_5)_4(C_5H_5)]\cdot 0.5(CH_2Cl_2$ |
| EDIDAY | 2.314 Å | Eckenhoff, W. T.; Biernesser, A. B.; Pintauer, T. *Inorg. Chem.* **2012**, *51*, 11917. $[Cu(P(C_6H_5)_3)_3]\cdot P(C_6H_5)_3\cdot (CH_3)_2CO$ |
| **Mean** | **2.286 Å/12 structures** | |

**Silver(I)**
*Three-coordination, triangular configuration*

| CSD code | *d*(Ag-P) | Reference, compound formula |
|---|---|---|
| ASIZUX | 2.410 Å | Clarke, A. J.; Ingleson, M. J.; Kociok-Kohn, G.; Mahon, M. F.; Patmore, N. J.; Rourke, J. P.; Ruggiero, G. D.; Weller, A. S. *J. Am. Chem. Soc.* **2004**, *126*, 1503. $[Ag(P(CH_3)_3)_2]B_{11}C_{12}H_{34}$ |

| | | |
|---|---|---|
| PAGMNT | 2.485 Å | Coucouvanis, D.; Baenziger, N. C.; Johnson, S. M. *Inorg. Chem.* **1974**, *13*, 1191. $[Ag(P(CH_3)_3)_2][Ni(S_2C_2(CN)_2)_2]$ |
| JOBQAS01 | 2.493 Å | Kuprat, M.; Schulz, A.; Thomas, M.; Villinger, A. *Can. J. Chem.* **2018**, *96*, 502. $[Ag(P(C_6H_5)_3)_3]CF_3SO_3{\cdot}2CH_2Cl_2$ JOBQAS 2.495 Å Nieger, M.; Hupfer, H.; Niecke, E.; Radseck, J. CCDC deposition number 112920, **1998**. $[Ag(P(C_6H_5)_3)_3]CF_3SO_3{\cdot}2CH_2Cl_2$ |
| SAXLIN | 2.502 Å | Franken, A.; McGrath, T. D.; Stone, F. G. A. *Organometallics* **2005**, *24*, 5157. $[Ag(P(C_6H_5)_3)_3][FeC_4H_8B_7O_3]$ |
| **Mean** | **2.493 Å/3 structures** | |

*Four-coordination, tetragonal configuration*

| CSD code | *d*(Ag-P) | Reference, compound formula |
|---|---|---|
| TACXAZ | 2.599 Å | Noll, J.; Korb, M.; Lang, H. *Acta Crystallogr., Sect. E* **2016**, *72*, 318. $[Ag(P(C_6H_5)_3)_4][Ag(P(C_6H_5)_3)(C_6H_{61}NO_6)]{\cdot}18CH_3OH$ |
| ANOXUZ | 2.600 Å | Exner, R. M.; Jenne, C.; Wegener, B. *Z. Anorg. Allg. Chem.* **2021**, *547*, 500. $[Ag(P(C_6H_5)_3)_4]_2(B_{12}H_{12}){\cdot}P(C_6H_5)_3$ |
| QEKWAH | 2.603 Å | Schwab, M. M.; Himmel, D.; Kacprzak, S.; Radtke, V.; Kratzert, D.; Weis, P.; Wernet, M.; Peter, A.; Yassine, Z.; Schmitz, D. ; Scheidt, E.-W.; Scherer, W.; Weber, S.; Feuerstein, W.; Breher, F.; Higelin, A.; Krossing, I. *Chem. – Eur. J.* **2017**, *24*, 918. $[Ag(P(C_6H_5)_3)_4][Al(OCF_3)_4]{\cdot}C_6H_4F_2$ |
| SULXOP | 2.603 Å | Sun, Q.; Nie, H.-H.; Su, H.-F.; Yang, S.-Y.;.; Teo, B. K. *Inorg. Chem.* **2020**, *59*, 8836. $[Ag(P(C_6H_5)_3)_4]_2[Ag_{14}C_{192}H_{202}O_{12}P_8S_{12}]{\cdot}12CH_3OH{\cdot}43.7H_2O$ |
| GORPUB | 2.611 Å | Zhdanov, A. P.; Voinova, V. V.; Klyukin, I. N.; Buzanov, G. A.; Grigorev, M. S.; Zhizhin, K. Y.; Kuznetsov, N. T. *Koord. Khim.* **2019**, *45*, 563. $[Ag(P(C_6H_5)_3)_4](B_{10}H_{12}N){\cdot}2(CH_3)_2NCHO$ |
| OTAHOH | 2.611 Å | Bowmaker, G. A.; Effendy; Hanna, J. V.; Healy, P. C.; King, S. P.; Pettinari, C.; Skelton, B. W.; White, A. H. *Dalton Trans.* **2011**, *40*, 7210. $[Ag(P(C_6H_5)_3)_4](HOCOO){\cdot}2C_2H_5OH{\cdot}3H_2O$ |
| JAJFAF | 2.614 Å | Mobs, J.; Luy, J.-N.; Shlyaykher, A.; Tonner, R.; Heine, J. *Dalton Trans.* **2021**, *50*, 15855. $[Ag(P(C_6H_5)_3)_4]_3(HP(C_6H_5)_3)_2[Bi_7I_{26}]{\cdot}2CH_3CN$ |
| VUFXOM | 2.616 Å | Malinina, E. A.; Korolenko, S. E.; Kubasov, A. S.; Goeva, L. V.; Avdeeva, V. V.; Kuznetsov, N. T. *Polyhedron* **2020**, *184*, 114566. $[Ag(P(C_6H_5)_3)_4]$ $[Ag(P(C_6H_5)_3)_2(B_{12}H_{12})$ |
| ANOYIO | 2.617 Å | Exner, R. M.; Jenne, C.; Wegener, B. *Z. Anorg. Allg. Chem.* **2021**, *547*, 500. $[Ag(P(C_6H_5)_3)_4]_2(B_{12}I_{12}){\cdot}2CH_3CN$ |
| DERYUX | 2.618 Å | Avdeeva, V. V.: Buzin, M. I.; Dmitrienko, A. O.; Dorovatovskii, P. V.; Malinina, E. A.; Kuznetsov, N. T.; Voronova, E. D.; Zubavichus, Y. V.; Vologzhanina, A. V. *Chem. – Eur. J.* **2017**, *23*, 16819. |

| | | |
|---|---|---|
| JUPDEG | 2.618 Å | Avdeeva, V. V.; Buzanov, G. A.; Malinina, E. A.; Kuznetsov, N. T.; Vologzhanina, A. V. *Crystals* **2020**, *10*, 389. $[Ag(P(C_6H_5)_3)_4](C_4B_{10}H_{17}O_2)[Ag(P(C_6H_5)_3)_2(C_{24}H_{30}B_{10}P_2]\cdot 0.1H_2O$ |
| IBOJOB | 2.620 Å | Avdeeva, V. V; Polyakova, I. N.; Vologzhanina, A. V; Malinina, E. A; Zhizhin, K. Y.; Kuznetsov, N. T. *Polyhedron* **2017**, *123*, 396. $[Ag(P(C_6H_5)_3)_4][AgC_{36}H_{40}B_{10}ClP_2]$ |
| UZUKAE | 2.620 Å | Schneider, L. N.; Krauel, E.-M. T.; Deutsch, C.; Urbahns, K.; Bischof, T.; Maibom, K. A. M.; Landmann, J.; Keppner, K.; Kerpen, C.; Hailmann, M.; Zapf, L.; Knuplez, T.; Bertermann, R.; Ignat'ev, N. V.; Finze, M. *Chem. -Eur. J.* **2021**, *27*, 10973. $[Ag(P(C_6H_5)_3)_4]N(CF_3)_2)\cdot CH_2Cl_2$ |
| PITMEN | 2.622 Å | Jenne, C.; Wegener, B. *Z. Anorg. Allg. Chem.* **2018**, *644*, 1123. $[Ag(P(C_6H_5)_3)_4](C_3B_{12}H_9Cl_{11}N)\cdot 5.35C_2Cl_2$ |
| XEGQIJ | 2.623 Å | Ellis, D. D.; Spek, A. L. *Acta Crystallogr., Sect. C* **2000**, *56*, e547. $[Ag(P(C_6H_5)_3)_4]B(C_6H_5)_4\cdot CH_3CN$ |
| LUTDUO | 2.625 Å | Altaf, M.; Stoeckli-Evans, H. *Polyhedron* **2010**, *29*, 701. $[Ag(P(C_6H_5)_3)_4]SbF_6\cdot CHCl_3$ |
| SUXLEF | 2.625 Å | Golubev, A. V.; Kubasov, A. S.; Turyshev, E. S; Bykov, A. Y.; Zhizhin, K. Y.; Kuznetsov, N. T. *Zh. Neorg. Khim.* **2020**, *65*, 1333. $[Ag(P(C_6H_5)_3)_4](C_{72}H_{60}B_{10}N_2O_4S_2)$ |
| DUQGII | 2.626 Å | A.S.Kubasov, E.Yu.Matveev, I.N.Klyukin, A.I.Nichugovskiy, K.Yu Zhizhin, N.T.Kuznetsov, N. T. *Inorg. Chim. Acta* **2020**, *510*, 119749. $[Ag(P(C_6H_5)_3)_4](C_5B_{10}H_{19}O)\cdot CH_2Cl_2$ |
| AROVEI | 2.628 Å | Yang, S.-Y.; Xie, Z.-X.; Ng, S. W. *Acta Crystallogr., Sect. C* **2004**, *60*, m123. $[Ag(P(C_6H_5)_3)_4][Sn_2(CF_3COO)_6]$ |
| USUMAY | 2.629 Å | Priola, E.; Bonometti, E.; Rabezzana, R.; Buscaino, R.; Chierotti, M. R.; Operti, L.; Diana, E. *Inorg. Chem. Commun.* **2016**, *70*, 35. $[Ag(P(C_6H_5)_3)_4][Cd(P(C_6H_5)Cl_3]$ |
| EDUVAC | 2.631 Å | Ng, S. W. *Acta Crystallogr., Sect. E* **2012**, *68*, m1536. $[Ag(P(C_6H_5)_3)_4](CF_3COO)\cdot C_2H_5OH$ |
| VUFYAZ | 2.631 Å | Malinina, E. A.; Korolenko, S. E.; Kubasov, A. S.; Goeva, L. V.; Avdeeva, V. V.; Kuznetsov, N. T. *Polyhedron* **2020**, *184*, 114566. $[Ag(P(C_6H_5)_3)_4](B_{12}H_{12})\cdot P(C_6H_5)_3$ |
| UHANOI | 2.633 Å | Zopes, D.; Hegemann, C.; Schlafer, J.; Tyrra, W.; Mathur, S. *Inorg. Chem.* **2015**, *54*, 3781. $[Ag(P(C_6H_5)_3)_4][Au(CF_3)_2]$ |
| RUGTOC | 2.635 Å | Long, D.-L.; Xin, X.-Q.; Chen, X.-M.; Kang, B.-S. *Polyhedron* **1997**, *16*, 1259. $[Ag(P(C_6H_5)_3)_4]_2[Mo_6O_{19}]\cdot 3CH_2Cl_2$ |
| IBOTEB | 2.637 Å | Avdeeva, V. V; Polyakova, I. N.; Vologzhanina, A. V; Malinina, E. A; Zhizhin, K. Y.; Kuznetsov, N. T. *Polyhedron* **2017**, *123*, 396. $[Ag(P(C_6H_5)_3)_4][AgC_{36}H_{39}B_{10}ClP_2]$ |
| HILKAS | 2.643 Å | Sun, S. B.; Dong, Y. J.; Lai, X. L.; Yuan, Z. Y.; Zhang, H.; Xie, J. L.; Wang, L. S.; Lei, P. X. *J. Coord. Chem.* **2023**, *49*, 176. $[Ag(P(C_6H_5)_3)_4](C_{40}H_{47}O_4S_4)\cdot (CH_3)_2NCHO$ |
| YADQEB | 2.646 Å | Jiang, Y.-H.; Cui, L.-N.; Huang, X.; Jin, Q.-H.; Zhang, C.-L. *Acta Crystallogr., Sect. E* **2011**, *67*, m1499. $[Ag(P(C_6H_5)_3)_4]CF_3SO_3\cdot CH_2Cl_2$ |

| | | |
|---|---|---|
| ISEMUP | 2.647 Å | Jin Wen, Yu-Han Jiang, Man-Hua Wu, Qiong-Hua Jin, Hui-Li Gong, H.-L. *Z. Kristallogr. – New Cryst Struct.* **2011**, *226*, 269. $[Ag(P(C_6H_5)_3)_4]CF_3SO_3$ |
| OFOYAJ | 2.647 Å | Romualdo, L. L.; Bessler, K. E.; Deflon, V. M.; Niquet, E. *Z. Anorg. Allg. Chem.* **2002**, *628*, 1098. $[Ag(P(C_6H_5)_3)_4](C_6H_5NNC(CN)_2)$ |
| CUGYIM | 2.659 Å | Pelizzi, C.; Pelizzi, G.; Tarasconi, P. *J. Organometal. Chem.* **1984**, *277*, 29. $[Ag(P(C_6H_5)_3)_4][Sn(O_2NO)_2(C_6H_5)_2Cl]\cdot[Sn(O_2NO)_3(C_6H_5)_2]$ |
| NOPMIO | 2.666 Å | Bowmaker, G. A.; Effendy; Hart, R. D.; Kildea, J. D.; de Silva, E. N.; Skelton, B. W.; White, A. H. *Aust. J. Chem.* **1997**, *50*, 539. $[Ag(P(C_6H_5)_3)_4]_2SiF_6\cdot C_5H_5N$ |
| IXINEI | 2.678 Å | Ng, S. W.; Rae, A. D. *Z. Kristallogr. Cryst. Mater.* **2003**, *218*, 581. $[Ag(P(C_6H_5)_3)_4][Sn(C_6H_5)_3(OOCCF_3)_2]$ |
| DUQGOO | 2.685 Å | Kubasov, A. S.; Matveev, E. Y.; Klyukin, I. N.; Nichugovskiy, A. I; Zhizhin, K. Y.; Kuznetsov, N. T. *Inorg. Chim. Acta* **2020**, *510*, 119749. $[Ag(P(C_6H_5)_3)_4](C_4B_{10}H_{17}O_2)$ |
| **Mean** | **2.629 Å/33 structures** | |

**Gold(I)**

*Two-coordination, linear configuration*

| CSD code | *d*(Au-O) | Reference, compound formula |
|---|---|---|
| KASXUX01 | 2.295 Å | Wang, J.-C.; Wang, Y. *Acta Crystallogr., Sect. B* **1993**, *49*, 131. $[Au(P(CH_3)_3)_2](C(CN)_3)$ |
| TEZYOO | 2.297 Å | Hofer, M.; Nevado, C. **2012**, 1338. $[Au(P(CH_3)_3)_2][Au(C_6F_5)_4]$ |
| BUJXEJ | 2.300 Å | Bruce, M. I.; Walton, J. K.; Skelton, B. W.; White, A. H. *J. Chem. Soc., Dalton Trans.* **1983**, 809. $[Au(P(CH_3)_3)_2](C_5(COOCH_3)_5)\cdot CH_3OH$ |
| IMIJEU | 2.300 Å | Savjani, N.; Lancaster, S. J.; Bew, S.; Hughes, D. L.; Bochmann, M. *Dalton Trans.* **2011**, *40*, 1079. $[Au(P(CH_3)_3)_2](C_{44}B_2F_{34}NO_2)\cdot C_4H_8O$ |
| VUXHED | 2.305 Å | Hueber, D.; Hoffmann, M.; de Fremont, P.; Pale, P.; Blanc, A. O*rganometallics* **2015**, *34*, 5065. $[Au(P(CH_3)_3)_2]_2[NCCH_3Au(P(CH_3)_3)_2]_2[W_{12}SiO_{40}]$ |
| WAGFUH | 2.305 Å | Seidel, G.; Lehmann, C. W.; Furstner, A. *Angew. Chem., Int. Ed.* **2010**, *49*, 8466. $[Au(P(CH_3)_3)_2]N(SO_2CF_3)_2$ |
| WEVZAY01 | 2.306 Å | Marsh, R. E.; Spek, A. L. *Acta Crystallogr., Sect. B* **2001**, 57, 800. $[Au(P(CH_3)_3)_2]((NC)_2C_2(CN)_2)\cdot(C_2H_5)_2O$ |
| WEVZAY | 2.307 Å | Alonso, P. J.; Cerrada, E.; Garin, J.; Gimeno, M. C.; Laguna, A.; Laguna, M.; Jones, P. G. *Synth. Met.* **1993**, *55*, 1772. $[Au(P(CH_3)_3)_2]\ ]((NC)_2C_2(CN)_2)\cdot(C_2H_5)_2O$ |
| BOSLON | 2.309 Å | Weis, P.; Riddlestone, I. M.; Scherer, H.; Krossing, I. *Chem. – Eur. J.* **2019**, *25*, 12159. $[Au(P(CH_3)_3)_2][Al(OC(CF)_3)_4]$ |

| | | |
|---|---|---|
| GACRAD | 2.310 Å | Baenziger, N. C.; Bennett, W. E.; Soboroff, D. M.; O'Donnell, P. S.; Doyle, J. R. *Polyhedron* **1998**, *17*, 2379. $[Au(P(CH_3)_3)_2]((NC)_2CC_6H_4C(CN)_2)$ |
| WIMLUA | 2.311 Å | Du, S.; Hodson, B. E.; Lei, P.; McGrath, T. D.; Stone, F. G. A. *Inorg. Chem.* **2007**, *46*, 6613. $[Au(P(CH_3)_3)_2][Ru_5C_{53}H_{43}B_8O_{10}P_2]\cdot 3CH_2Cl_2$ |
| SUBSIT | 2.312 Å | Braunschweig, H.; Radacki, K.; Shang, R. *Chem. Sci.* **2015**, *6*, 2989. $[Au(P(CH_3)_3)_2]B(C_6H_2F_3)_4\cdot C_6H_5OH$ |
| QICVOO | 2.313 Å | Mohamed, A. A.; Burini, A.; Galassi, R.; Paglialunga, D.; Galan-Mascaros, J.-R.; Dunbar, K. R.; Fackler, Jr., J. P. *Inorg. Chem.* **2007**, *46*, 2348. $[Au(P(CH_3)_3)_2][Cu_6C_{30}H_{12}F_{36}N_{12}O_6]\cdot 2CH_3CN$ |
| SOSTAV | 2.313 Å | Baukova, T. V.; Ellert, O. G.; Kuz'mina, L. G.; Dvortsova, N. V.; Lemenovskii, D. A.; Rubezhov, A. Z. *Mendeleev Commun.* **1991**, 22. $[Au(P(CH_3)_3)_2]((NC)_2C_2(CN)_2)$ |
| KUJPAK | 2.314 Å | Wang, M.; Zhang, S.; Gong, Y.; Zhang, W.; Wang, Y.; Chen, Y.; Zheng, Q.; Liu, Z.; Tang, C. *Angew. Chem., Int. Ed.* **2024**, *63*, e202409283. $[Au(P(CH_3)_3)_2]C_3B_{10}H_{13})_2]$ |
| BARBUT | 2.315 Å | Less, R. J.; Guan, B.; Muresan, N. M.; McPartlin, M.; Reisner, E.; Wilson, T. C.; Wright, D. S. *Dalton Trans.* **2012**, *41*, 5919. $[Au(P(CH_3)_3)_2](C_5(CN)_5)$ |
| UXITUS | 2.315 Å | Jones, P. G. CCDC deposition number 1508593, **2016**. $[Au(P(CH_3)_3)_2](CF_3SO_3$ |
| KASXUX | 2.316 Å | Baukova, T. V.; Kravtsov, D. N.; Kuz'mina, L. G.; Dvortsova, N. V.; Porai-Koshits, M. A.; Perevalova, E. G. *J. Organometal. Chem.* **1989**, *372*, 465. $[Au(P(CH_3)_3)_2](C(CN)_3)$ |
| WAGFUH01 | 2.320 Å | Grirrane, A.; Garcia, H.; Corma, A.; Alvarez, E. *Chem. – Eur. J.* **2013**, *19*, 12239. $[Au(P(CH_3)_3)_2]N(SO_2CF_3)_2$ |
| ZEPYIC | 2.322 Å | Anderson, S.; Mullica, D. F.; Sappenfield, E. L.; Stone F. G. A. *Organometallics* **1995**, *14*, 3516. $[Au(P(CH_3)_3)_2][RuC_4H_1B_9ClO_2]$ |
| **Mean** | **2.309 Å/20 structures** | |

*Three-coordination, triangular configuration*

| CSD code | *d*(Au-O) | Reference, compound formula |
|---|---|---|
| ANOYUA | 2.367 Å | Exner, R. M.; Jenne, C.; Wegener, B. *Z. Anorg. Allg. Chem.* **2021**, *647*, 500. $[Au(P(C_6H_5)_3)_3]B_{12}I_{12}$ |
| TPAWTB10 | 2.373 Å | Guggenberger, L. J. *J. Organometal. Chem.* **1974**, *81*, 271. $[Au(P(C_6H_5)_3)_3](B_9H_{12}S)$ |
| NODZUD | 2.375 Å | Konarev, D. V.; Khasanov, S. S.; Otsuka, A.; Ishikawa, M.; Yamochi, H.; Saito, G.; Lyubovskaya, D. R. N. *Inorg. Chem.* **2014**, *53*, 6850. $[Au(P(C_6H_5)_3)_3]_2(C_{60})_2\cdot C_6H_4Cl_{12}$ |
| ZETBIJ | 2.377 Å | Olbrich, F.; Lagow, R. J. *Z. Anorg. Allg. Chem.* **1995**, *621*, 1929. $[Au(P(C_6H_5)_3)_3]SiF_5\cdot 0.5CH_2Cl_2$ |
| CUHDEQ | 2.378 Å | Hashimoto, A.; Yoshinari, N.; Konno, T. *Chem. Lett.* **2015**, *44*, 749. $[Au(P(C_6H_5)_3)_3]NO_3$ |
| EGUSAA | 2.379 Å | Hamel, A.; Schier, A.; Schmidbaur, H. *Z. Naturforsch., Sect. B* **2002**, *57*, 877. $[Au(P(C_6H_5)_3)_3]Cl\cdot 2CH_2Cl_2$ |
| NIWKOV | 2.381 Å | Malberg, J.; Bodensteiner, M.; Paul, D.; Wiegand, T.; Eckert, H.; Wolf, R. *Angew. Chem., Int. Ed.* **2014**, *53*, 2771. $[Au(P(C_6H_5)_3)_3][AuCo_2C_{40}H_{72}P_8]\cdot P(C_6H_5)_3\cdot C_6H_{14}$ |

| | | |
|---|---|---|
| TPAUPB | 2.384 Å | Jones, P. G. *Acta Crystallogr., Sect. B* **1980**, *36*, 3105. $[Au(P(C_6H_5)_3)]B(C_6H_5)_3)_4$ |
| ZEPYAU | 2.386 Å | Davidson, J. L.; Lindsell, W. E.; McCullough, K. J.; McIntosh, C. H. *Organometallics* **1995**, *14*, 3497. $[Au(P(C_6H_5)_3)_3][MoC_{29}H_5F_{20}S_4]\cdot 0.5CH_2Cl_2$ |
| AUBPHA | 2.402 Å | Jones, P. G. *J. Chem. Soc., Chem. Commun.* **1980**, 1031. $[Au(P(C_6H_5)_3)]B(C_6H_5)_4\cdot P(C_6H_5)_3\cdot CHCl_3$ |
| BIVRUT | 2.403 Å | Beurskens, P. T.; Pet, R.; Noordik, J. H.; van der Velden, J. W. A.; Bour, J. J. *Cryst. Struct. Commun.* **1982**, *11*, 1039. $[Au(P(C_6H_5)_3)_3]_2[Ag(P(C_6H_5)_3)]NO_3$ |
| **Mean** | **2.382 Å/11 structures** | |

*Four-coordination, tetrahedral configuration*

| CSD code | *d*(Au-O) | Reference, compound formula |
|---|---|---|
| KICLOX10 | 2.523 Å | Lansun, Z.; Huahui, Y.; Wenbing, Y.; Qianer, Z. *J. Xiamen Univ.* **1990**, *29*, 421. $[Au(P(C_6H_5)_3)_4]ClO_4$ |
| AUBPHC | 2.533 Å | Jones, P. G. *J. Chem. Soc., Chem. Commun.* **1980**, 1031. $[Au(P(C_6H_5)_3)_4]B(C_6H_5)_4\cdot CH_3CN$ |
| AUBPHB | 2.606 Å | Jones, P. G. *J. Chem. Soc., Chem. Commun.* **1980**, 1031. $[Au(P(C_6H_5)_3)_4]B(C_6H_5)_4$ |
| **Mean** | **2.528 Å/2 structures** | |

**Structures of trialkyl phosphite and phosphine solvated metal ions in liquid ammonia solution**

| | *d*(M-P) | *N* | Reference and formula |
|---|---|---|---|
| *Copper(I)* | | | |
| Trimethyl phosphite | 2.245 Å | 4 | Nilsson, K. B.; Persson, I. *Dalton. Trans*. **2004**, 1312. |
| Triethyl phosphite | 2.258 Å | 4 | Nilsson, K. B.; Persson, I. *Dalton. Trans*. **2004**, 1312. |
| Tri(iso-propyl) phosphite | 2.258 Å | 4 | Nilsson, K. B.; Persson, I. *Dalton. Trans*. **2004**, 1312. |
| Tri(*n*-butyl) phosphite | 2.249 Å | 4 | Nilsson, K. B.; Persson, I. *Dalton. Trans*. **2004**, 1312. |
| Tri(*n*-butyl) phosphine | 2.278 Å | 4 | Nilsson, K. B.; Persson, I. *Dalton. Trans*. **2004**, 1312. |
| *Silver(I)* | | | |
| Triethyl phosphite | 2.482 Å | 3 | Nilsson, K. B.; Persson, I.; Kessler, V. G *Inorg. Chem.* **2006**, 45, 6912. |
| Tri(*n*-butyl) phosphite | 2.513 Å | 3 | Nilsson, K. B.; Persson, I.; Kessler, V. G *Inorg. Chem.* **2006**, 45, 6912. |
| Tri(*n*-butyl) phosphine | 2.528 Å | 3 | Nilsson, K. B.; Persson, I.; Kessler, V. G *Inorg. Chem.* **2006**, 45, 6912. |
| *Gold(I)* | | | |
| Triethyl phosphite | 2.367 Å | 3 | Nilsson, K. B.; Persson, I.; Kessler, V. G *Inorg. Chem.* **2006**, 45, 6912. |
| Tri(*n*-butyl) phosphine | 2.399 Å | 3 | Nilsson, K. B.; Persson, I.; Kessler, V. G *Inorg. Chem.* **2006**, 45, 6912. |

**Table S5.** Summary of structures of acetonitrile solvated metal ions in the solid state. Citations in purple text have not been included in the calculated mean bond distances.

**Lithium(I)**

*Four-coordination, tetrahedral configuration*

| CSD code | *d*(Li-O) | Reference, compound formula |
|---|---|---|
| YADTUS | 1.996 Å | Hoyer, M.; Hartl, H. *Z. Anorg. Allg. Chem.* **1992**, *612*, 45. $[Li(NCCH_3)_4](Cu_2I_3)$ |
| IDILEO | 2.004 Å | Breunig, J. M.; Wietelmann, U. ; Lerner, H.-W.; Bolte, M. *Acta Crystallogr., Sect. E* **2013**, *69*, m253. $[Li(NCCH_3)_4]B(NCS)_4$ |
| SUVXEM | 2.012 Å | Yokota, Y.; Young Jr., V. G.; Verkade, J. G. *Acta Crystallogr., Sect. C* **1999**, *55*, 196. $[Li(NCCH_3)_4]ClO_4$ |
| NIXSES | 2.013 Å | Bach, A.; Hoyer, M.; Hartl, H. *Z. Naturforsch., Teil B* **1997**, *52*, 1497. $[Li(NCCH_3)_4]_4[Cd_6I_{16}]\cdot 1.5CH_3CN$ |
| QIXXON | 2.015 Å | Møller, K. T.; Paskevicius, M.; Andreasen, J. G.; Lee, J.; Chen-Tan, N.; Overgaard, J.; Payandeh, S.; Silvester, D. S.; Buckley, C. E.; Jensen, T. R. *Chem. Commun.* **2019**, *55*, 3410. |
| RUFQUG | 2.017 Å | Redshaw, C.; Walton, M. J.; Michiue, K.; Chao, Y.; Walton, A.; Elo, P.; Sumerin, V.; Jiang, C.; Elsegood, M. R. J. *Dalton Trans.* **2015**, *44*, 12292. $[Li(NCCH_3)_4][V_2LiC_{134}H_{163}NO_{14}]\cdot 9.67CH_3CN$ |
| RUFQOA | 2.021 Å | Redshaw, C.; Walton, M. J.; Michiue, K.; Chao, Y.; Walton, A.; Elo, P.; Sumerin, V.; Jiang, C.; Elsegood, M. R. J. *Dalton Trans.* **2015**, *44*, 12292. $[Li(NCCH_3)_4][V_2LiC_{134}H_{163}NO_{14}]\cdot 8CH_3CN$ |
| CAYMIB | 2.024 Å | Suzuki, Y. ; Ishida, S. ; Sato, S. ; Isobe, H. ; Iwamoto, T. *Angew. Chem., Int. Ed.* **2017**, *56*, 4593. $[Li(NCCH_3)_4](C_{26}H_{54}BSi_5)$ |
| AWOXIU | 2.027 Å | Seo, D. M.; Boyle, P. D.; Henderson, W. A. *Acta Crystallogr., Sect. E* **2011**, *67*, m1148. $[Li(NCCH_3)_4]PF_6\cdot CH_3CN$ |
| VIXVUU | 2.027 Å | Rotter, O.; Schuster, M.; Kidik, M.; Schon, O.; Klapotke, T. M.; Karaghiosoff, K. *Inorg. Chem.* **2008**, *47*, 1663. $[Li(NCCH_3)_4]_2(P_2Se_8)$ |
| PENZIT | 2.032 Å | Seo, D. M.; Boyle, P. D.; Borodin, O.; Henderson, W. A. *RSC Adv.* **2012**, *2*, 8014. $[Li(NCCH_3)_4]PF_6\cdot 2CH_3CN$ |
| SAWTAL | 2.032 Å | Raston, C. L.; Whitaker, C. R.; White, A. H. *Aust. J. Chem.* **1989**, *42*, 201. $[Li(NCCH_3)_4]I$ |
| YENMIP | 2.032 Å | Zhang, B.; Koberl, M.; Pothig, A.; Cokoja, M.; Herrmann, W. A.; Kuhn, F. E. *Z. Naturforsch., Teil B* **2012**, *67*, 1030. $[Li(NCCH_3)_4](B(C_6F_5)_4$ [ |
| VIPTUJ | 2.034 Å | Jacoby, D.; Floriani, C.; Chiesi-Villa, A.; Rizzoli, C. *Chem. Commun.* **1991**, 220. $[Li(NCCH_3)_4][FeN_4C_{28}H_{32}]$ |
| JISBOF | 2.037 Å | Ebner, F. ; Greb, L. *J. Am. Chem. Soc.* **2018**, *140*, 17409. $[Li(NCCH_3)_4](C_{28}H_{33}N_4Si)$ |
| KUGJIH | 2.038 Å | Redshaw, C.; Walton, M. J.; Lee, D. S.; Jiang, C. ; Elsegood, M. R. J.; Michiue, K. *Chem. Eur. J.* **2015**, *21*, 5199. $[Li(NCCH_3)_4][V_2C_{88}H_{105}O_{10}]\cdot 8CH_3CN$ |

| | | |
|---|---|---|
| QABKIQ | 2.039 Å | Blasing, K.; Ellinger, S.; Harloff, J.; Schulz, A.; Sievert, K.; Taschler, AC.; Villinger, A.; Taschler, C. Z. *Chem. Eur. J.* **2016**, *22*, 4175. $[Li(NCCH_3)_4][Li(CN)_6P_2F_6]$ |
| **Mean** | **2.024 Å/17 structures** | |

**Sodium(I)**

*Five-coordination, square-pyramidal configuration*

| | | |
|---|---|---|
| GUQZEY | 2.388 Å | Arbaoui, A.; Redshaw, C.; Elsegood, M. R. J.; Wright, V. E.; Yoshizawa, A.; Yamato, T. *Chem. Asian J.* **2010**, *5*, 621. $[Na(NCCH_3)_5]_2[Fe_4Na_2C_{136}H_{166}N_2O_{16}]\cdot[Fe_4Na_4C_{144}H_{178}N_6O_{16}]\cdot 9.46CH_3CN$ |
| KUGHIF | 2.445 Å | Redshaw, C.; Walton, M. J.; Lee, D. S.; Jiang, C.; Elsegood, M. R. J.; Michiue, K. *Chem. Eur. J.* **2015**, *21*, 5199. $[Na(NCCH_3)_5]_2[V_4C_{88}H_{105}O_{10}]\cdot 4CH_3CN$ |
| KAWMUS | 2.458 Å | Ceriotti, A.; Daghetta, M.; El Afefey, S. ; Ienco, A.; Longoni, G.; Manca, G.; Mealli, C.; Zacchini, S.; Zarra, S. *Inorg. Chem.* **2011**, *50*, 12553. $((n\text{-}C_4H9)_4N)_2[Na(NCCH_3)_5][Pt_5Sn_6C_5H_3Cl_{12}O_8]\cdot 2CH_3CN$ |
| **Mean** | **2.452 Å/2 structures** | |

*Six-coordination, octahedral configuration*

| CSD code | *d*(Na-O)$_b$ | Reference, compound formula |
|---|---|---|
| FUMQUZ | 2.499 Å | Müller, U. ; Noll, A. *Z. Kristallogr.-New Struct.* **2000**, *215*, 191. $[Na(NCCH_3)_6][TaCl_6]$ |

**Potassium(I)**

*Six-coordination, octahedral configuration*

| CSD code | *d*(Na-O)$_b$ | Reference, compound formula |
|---|---|---|
| MEKNAU | 2.838 Å | Müller, U. ; Noll, A. *Z. Kristallogr.-New Struct.* **2000**, *215*, 191. $[K(NCCH_3)_6]_2[U(OC_{19}H_{22}N)_6]$ |

**Magnesium(II)**

*Six-coordination, octahedral configuration*

| CSD code | *d*(Mg-O) | Reference, compound formula |
|---|---|---|
| ERALAM | 2.144 Å | Keyzer, E. N.; Glass, H. F. J.; Liu, Z.; Bayley, P. M.; Dutton, S. E.; Grey, C. P.; Wright, D. S. *J. Am. Chem. Soc.* **2016**, *138*, 8682. $[Mg(NCCH_3)_6](PF_6)_2$ |
| OKUPER | 2.148 Å | Levason, W.; Pugh, D.; Purkis, J. M.; Reid, G. *Dalton Trans.* **2016**, *45*, 7900. $[Mg(NCCH_3)_6](B(2,4\text{-}(CF_3)_2C_6H_3)_4)_2$ |
| FUNJAZ | 2.149 Å | Drew, M. G. B.; Claire, P. P. K.; Willey, G. R. *J. Cherm. Soc., Dalton Trans.* **1988**, 215. . $[Mg(NCCH_3)_6](SbCl_4)_2$ |

| | | |
|---|---|---|
| GADPOT | 2.149 Å | Veryasov, G.; Matsumoto, K.; Hagiwara, R. *Dalton Trans.* **2016**, *45*, 2810. $[Mg(NCCH_3)_6](N(SO_2CF_3)_2)_2$ |
| WIBJOG | 2.151 Å | Willey, G. R.; Palin, J.; Drew, M. G. B. *J. Chem. Soc., Dalton Trans.* **1994**, 1799. $[Mg(NCCH_3)_6][CH_3CNTiCl_4)O]\cdot 4CH_3CN$ |
| LOQQEQ | 2.154 Å | Rodl, C.; Schwedtmann, K.; Weigand, J. J.; Wolf, R. *Chem. Eur. J.* **2019**, *25*, 6180. $[Mg(NCCH_3)_6][Co(C_{10}H_{18}P_2)_2]\cdot 2CH_3CN$ |
| DOTZIV | 2.155 Å | Rabe, S.; Müller, U. *Z. Naturforsch., Teil B* **2000**, *55*, 553. $[Mg(NCCH_3)_6](AsCl_3)_2\cdot 2AsCl_4$ |
| POBROP | 2.155 Å | Chitnis, S.S.; Burford, N.; McDonald, R.; Ferguson, M. J. *Inorg. Chem.* **2014**, *53*, 5359. $[Mg(NCCH_3)_6](SbCl_4(P(CH_3)_3)_2$ |
| XUHJIW | 2.155 Å | Schorpp, M.; Krossing, I. *Chem. Sci.* **2020**, *11*, 2068. $[Mg(NCCH_3)_6][Al(OC(CF_3)_3)_4]_2\cdot C_6H_4F_2$ |
| TANQEE | 2.157 Å | Waters, A. F.; White, A. H. *Aust. J. Chem.* **1996**, *49*, 27. $[Mg(NCCH_3)_6][MgBr_4]$ |
| HAVLOG | 2.160 Å | Cheung, M.-S.; Chan, H.-S.; Xie, Z. *Organometallics* **2005**, *24*, 4468. $[Mg(NCCH_3)_6][ErC_{16}H_{48}B_{20}N_4]\cdot CH_3CN$ |
| KIPWUB | 2.233 Å | Willey, G. R.; Collins, H.; Drew, M. G. B. *J. Chem. Soc., Dalton Trans.* **1991**, 961. $[Mg(NCCH_3)_6]_2[Bi_4Cl_{16}]$ |
| **Mean** | **2.152 Å/11 structures** | |

**Calcium(II)**

*Eight-coordination, square antiprismatic configuration*

| CSD code | *d*(Ca-O) | Reference, compound formula |
|---|---|---|
| OKUPOB | 2.514 Å | Levason, W.; Pugh, D.; Purkis, J. M.; Reid, G. *Dalton Trans.* **2016**, *45*, 7900. $[Ca(NCCH_3)_8](B(2,4\text{-}(CF_3)_2C_6H_3)_4)_2$ |
| **Mean** | **2.514 Å/1 structures** | |

**Strontium(II)**

*Eight-coordination, square antiprismatic configuration*

| CSD code | *d*(Sr-O) | Reference, compound formula |
|---|---|---|
| OKUQIW | 2.682 Å | Levason, W.; Pugh, D.; Purkis, J. M.; Reid, G. *Dalton Trans.* **2016**, *45*, 7900. $[Sr(NCCH_3)_8](B(2,4\text{-}(CF_3)_2C_6H_3)_4)_2$ |
| **Mean** | **2.682 Å/1 structure** | |

**Barium(II)**

*Eight-coordination, square antiprismatic configuration*

| CSD code | $d$(Ba-N)+$d$(Ba-O) | Reference, compound formula |
|---|---|---|
| PUWKUQ | 2.917 + 2.716 Å | Asmis, K. R.; Beele, B. B.; Jenne, C.; Kawa, S.; Knorke, H.; Nierstenhofer, M. C.; Wang, X.-B.; Warneke, J. ; Warneke, Z. ; Yuan, Q. *Chem. Eur. J.* **2020**, *26*, 14594. $[Ba(NCCH_3)_8(H_2O)]\cdot(B_{12}Cl_{11}NO_2)\cdot(C_2H_5)_2O\cdot CH_3CN$ |

**Yttrium(III)**

*Eight-coordination, square antiprismatic configuration*

| CSD code | $d$(Y-O) | Reference, compound formula |
|---|---|---|
| PAPXOW | 2.482 Å | Zhu, X.; Li, G.; Xu, F.; Zhang, Y.; Xue, M. Shen, Q. *Tetrahedron* **2017**, *73*, 1451. $[Y(NCCH_3)_9][AlCl_4]_3\cdot CH_3CN$ |
| **Mean** | **2.482 Å/1 structures** | |

**Lanthanum(III)**

*Nine-coordination, tricapped prismatic configuration*

| CSD code | $d$(La-N) | Reference, compound formula |
|---|---|---|
| GIWVUD | 2.628 Å | Deacon, G. B.; Gortler, B.; Junk, P. C.; Lork, E.; Mews, R.; Petersen, J.; Zemva, B. *J. Chem. Soc., Dalton Trans.* **1998**, 3887. $[La(NCCH_3)_9](AsF_6)_3\cdot CH_3CN$ |
| ZACWIM | 2.639 Å | Brown, J. L.; Davis, B. L.; Scott, B. L.; Gaunt, A. J. *Inorg. Chem.* **2015**, *54*, 11958. $[La(NCCH_3)_9][LaI_6]\cdot CH_3CN$ |
| ZACWOS | 2.642 Å | Brown, J. L.; Davis, B. L.; Scott, B. L.; Gaunt, A. J. *Inorg. Chem.* **2015**, *54*, 11958. $[La(NCCH_3)_9][AlCl_4]_3\cdot CH_3CN$ |
| **Mean** | **2.636 Å/3 structures** | |

**Cerium(III)**

*Eight-coordination, square antiprismatic configuration*

| CSD code | $d$(Ce-N) | Reference, compound formula |
|---|---|---|
| ZACWUY | 2.611 Å | Brown, J. L.; Davis, B. L.; Scott, B. L.; Gaunt, A. J. *Inorg. Chem.* **2015**, *54*, 11958. $[Ce(NCCH_3)_9][AlCl_4]_3\cdot CH_3CN$ |
| **Mean** | **2.611 Å/1 structure** | |

**Praseodymium(III)**

*Nine-coordination, tricapped prismatic configuration*

| CSD code | *d*(Pr-N) | Reference, compound formula |
|---|---|---|
| OBUHOI | 2.553 Å | Willey, G. R.; Aris, D. R.; Errington, W. *Inorg. Chim. Acta* **2001**, *318*, 97. $[Pr(NCCH_3)_9][SnCl_5(NCCH_3)]_3$ |
| GIWWEO | 2.591 Å | Deacon, G. B.; Gortler, B.; Junk, P. C.; Lork, E.; Mews, R.; Petersen, J.; Zemva, B. *J. Chem. Soc., Dalton Trans.* **1998**, 3887. $[Pr(NCCH_3)_9](AsF_6)_3 \cdot CH_3CN$ |
| OBUHIC | 2.600 Å | Willey, G. R.; Aris, D. R.; Errington, W. *Inorg. Chim. Acta* **2001**, *318*, 97. $[Pr(NCCH_3)_9]_2[SnCl_5(OC_4H_8)]_5[SnCl_5(NCCH_3)]$ |
| **Mean** | **2.581 Å/3 structures** | |

**Neodymium(III)**

*Nine-coordination, tricapped prismatic configuration*

| CSD code | *d*(Nd-N) | Reference, compound formula |
|---|---|---|
| ZACWEI | 2.571 Å | Brown, J. L.; Davis, B. L.; Scott, B. L.; Gaunt, A. J. *Inorg. Chem.* **2015**, *54*, 11958. $[Nd(NCCH_3)_9]_2[NdI_5(NCCH_3)][NdI_6]I \cdot 3CH_3CN$ |
| ZACXAF | 2.571 Å | Brown, J. L.; Davis, B. L.; Scott, B. L.; Gaunt, A. J. *Inorg. Chem.* **2015**, *54*, 11958. $[Ce(NCCH_3)_9][AlCl_4]_3 \cdot CH_3CN$ |
| PAPXUC | 2.574 Å | Zhu, X.; Li, G.; Xu, F.; Zhang, Y.; Xue, M. Shen, Q. *Tetrahedron* **2017**, *73*, 1451. $[Nd(NCCH_3)_9][FeCl_4]_3 \cdot CH_3CN$ |
| ZACXAF01 | 2.575 Å | Zhu, X.; Li, G.; Xu, F.; Zhang, Y.; Xue, M.; Shen Q. *Tetrahedron* **2017**, *73*, 1451. $[Ce(NCCH_3)_9][AlCl_4]_3 \cdot CH_3CN$ |
| **Mean** | **2.573 Å/4 structures** | |

**Samarium(III)**

*Nine-coordination, tricapped prismatic configuration*

| CSD code | *d*(Sm-N) | Reference, compound formula |
|---|---|---|
| GIWVUD | 2.534 Å | Deacon, G. B.; Gortler, B.; Junk, P. C.; Lork, E.; Mews, R.; Petersen, J.; Zemva, B. *J. Chem. Soc., Dalton Trans.* **1998**, 3887. $[Sm(NCCH_3)_9](AsF_6)_3 \cdot CH_3CN$ |
| **Mean** | **2.534 Å/1 structure** | |

**Terbium(III)**

*Nine-coordination, tricapped prismatic configuration*

| CSD code | *d*(Tb-N) | Reference, compound formula |
|---|---|---|
| LILVIO | 2.503 Å | Zottnick, S. H.; Daul, W. G.; Kerpen, C.; Finze, M.; Müller-Buschbaum, K. *Chem. Eur. J.* **2018**, *24*, 15287. $[Tb(NCCH_3)_9][Tb_2C_{18}H_{18}B_9N_{18}]$ |
| **Mean** | **2.503 Å/1 structure** | |

**Thulium(III)**

*Eight-coordination, square antiprismatic configuration*

| CSD code | *d*(Er-O) | Reference, compound formula |
|---|---|---|
| KUBMUQ | 2.411 Å | Bodizs, G.; Raabe, I.; Scopelliti, R.; Krossing, I.; Helm, L. *Dalton Trans.* **2009**, 5137. $[Tm(NCCH_3)_8][Al(OC(CF_3)_3)_4]$ |
| **Mean** | **2.411 Å/1 structure** | |

**Ytterbium(II)**

*Seven-coordination*

| CSD code | *d*(Yb-N) | Reference, compound formula |
|---|---|---|
| WIJZOH | 2.525 Å | Xemard, M.; Zimmer, S.; Cordier, M.; Goudy, V.; Ricard, L.; Clavaguera, C.; Nocton, G. *J. Am. Chem. Soc.* **2018**, *140*, 14433. $[Yb(NCCH_3)_7](C_9H_9)_2$ |
| **Mean** | **2.525 Å/1 structures** | |

**Ytterbium(III)**

*Eight-coordination, square antiprismatic configuration*

| CSD code | *d*(Yb-N) | Reference, compound formula |
|---|---|---|
| AJOWIF | 2.556 Å | Evans, W. J.; Johnston, M. A.; Greci, M. A.; Gummersheimer, T. S. ; Ziller, J. W. *Polyhedron* **2003**, *22*, 119. $[Yb(NCCH_3)_8](B(C_6H_5)_4)_2{\cdot}2CH_3CN$ |
| GIWWIS | 2.395 Å | Deacon, G. B.; Gortler, B.; Junk, P. C.; Lork, E.; Mews, R.; Petersen, J.; Zemva, B. *J. Chem. Soc., Dalton Trans.* **1998**, 3887. $[Yb(NCCH_3)_9](AsF_6)_3{\cdot}CH_3CN$ |
| **Mean** | **2.xxx Å/x structures** | |

**Uranium(III)**

*Nine-coordination*

| CSD code | *d*(U-N) | Reference, compound formula |
|---|---|---|
| AJEKEF | 2.614 Å | Enriquez, A. E.; Matonic, J. H.; Scott, B. L.; Neu, N. P. *Chem. Commun.* **2003**, 1892. $[U(NCCH_3)_9]I_3$ |
| **Mean** | **2.614 Å/1 structure** | |

**Plutonium(III)**

*Nine-coordination*

| CSD code | *d*(Pu-N) | Reference, compound formula |
|---|---|---|
| AJEKEF | 2.572 Å | Enriquez, A. E.; Matonic, J. H.; Scott, B. L.; Neu, N. P. *Chem. Commun.* **2003**, 1892. $[Pu(NCCH_3)_9](PF_6)_3{\cdot}CH_3CN$ |
| **Mean** | **2.572 Å/1 structure** | |

**Vanadium(II)**

*Six-coordination, octahedral configuration*

| CSD code | *d*(V-O) | Reference, compound formula |
|---|---|---|
| BEQQIX | 2.109 Å | Janas, Z.; Jerzykiewicz, L. B.; Przybylak, S.; Richards, R. L.; Sobota, P. *Organometallics* **2000**, *19*, 4252. $[V(NCCH_3)_6][VCl_2(S(CH_2)_2O(CH_2)_2S)]_2$ |
| CUPYER | 2.109 Å | Chandrasekhar, P.; Bird, P. H. *Inorg. Chim. Acta* **1985**, *97*, L31. $[V(NCCH_3)_6][ZnCl_4]$ |
| CUPYER01 | 2.113 Å | Clemente, D. A. *Inorg. Chim. Acta* **2005**, *358*, 1725. $[V(NCCH_3)_6][ZnCl_4]$ |
| YIRLOA | 2.116 Å | Hitchcock, P. B.; Hughes, D. L.; Leigh, G. J.; Sanders, J. R.; de Souza, J.; McGarry, C. J.; Larkworthy, L. F. *J. Chem. Soc., Dalton Trans.* **1994**, 3683. $[V(NCCH_3)_6]I_4$ |
| TOQYUW | 2.117 Å | Rheingold, A. L.; Miller, J. S. CCDC deposition number 1961260, **2019**. $[V(NCCH_3)_6]I_2{\cdot}CH_3CN$ |
| **Mean** | **2.113 Å/5 structures** | |

**Chromium(II)**

*Four-coordination, square-planar configuration*

| CSD code | *d*(Cr-N) | Reference, compound formula |
|---|---|---|
| NOFKYUO | 2.066 Å | Henriques, R. T.; Herdtweck, E.; Kuhn, F. E.; Lopes, A. D.; Mink, J.; Romao, C. C *J. Chem. Soc., Dalton Trans.* **1998**, 1293. $[Cr(NCCH_3)_2)_4](BF_4)_2$ |

*Six-coordination, Jahn-Teller distorted octahedral configuration*

| CSD code | *d*(Cr-N) | Reference, compound formula |
|---|---|---|
| WUTDEW | 2.079 + 2.423 Å | Thangavel, A.; Wieliczko, M.; Scarborough, C.; Dittrich, B.; Bacsa, J. *Acta Crystallogr., Sect. C* **2015**, *71*, 936. $[Cr(NCCH_3)_2)_6][Cr(NCCH_3)_2)_6](B(C_6H_5)_4)_2]\cdot 2CH_3CN$ |
| **Mean** | **2.079+2.423 Å/1 structure** | |

**Chromium(III)**

*Six-coordination, octahedral configuration*

| CSD code | *d*(Cr-N) | Reference, compound formula |
|---|---|---|
| ACALEW | 2.000 Å | Hatlevik, O.; Arif, A. M.; Miller, J. S. *J. Phys. Chem. Solids* **2004**, *65*, 61. $[Cr(NCCH_3)_2)_6](BF_4)_2$ |
| **Mean** | **2.000 Å/1 structure** | |

**Manganese(II)**

*Six-coordination, octahedral configuration*

| CSD code | *d*(Mn-O) | Reference, compound formula |
|---|---|---|
| ZOTYAI | 2.222 Å | Weller, F.; Mai, H.-J.; Dehnicke, K. *Z. Naturforsch. Teil B* **1996**, *51*, 298. $[Mn(NCCH_3)_6][MnI_4]$ |
| ZOTYAI01 | 2.222 Å | Rheingold, A. L.; Miller, J. S. CCDC deposition number 1961270, **2016**. $[Mn(NCCH_3)_6][MnI_4]$ |
| ZOWPEJ | 2.223 Å | Sarbajna, A.; He, Y.-T.; Dinh, M. H.; Gladkovskaya, O.; Wahidur Rahaman, S. M.; Karimata, A.; Khaskin, E. M.; Lapointe, S.; Fayzullin, R. R.; Khusnutdinova, J. R. *Organometallics* **2019**, *38*, 4409. $(NO)[Mn(NCCH_3)_6](C_{23}H_{32}BrN_3)_2(BF_4)_6$ |
| UPIBAY | 2.227 Å | Ilyukhin, A.; Gavrikov, A. CCDC deposition number 1476232, **2016**. $[Mn(NCCH_3)_6][Dy(O_2NO)_4]$ |
| DADYEP | 2.228 Å | Gavrikov, A.; Koroteev, P.; Ilyukhin, A.; Efimov, N.; Kostopoulos, A. K.;Baranchikov, A.; Tyurin, A.; Kirdyankin, D.; Gavrichev, K.; Tuna, F.; Dobrokhotova, Z. *Polyhedron* **2017**, *122*, 184. $[Mn(NCCH_3)_6][Tm(O_2NO)_4]$ |
| DADYIT | 2.228 Å | Gavrikov, A.; Koroteev, P.; Ilyukhin, A.; Efimov, N.; Kostopoulos, A. K.;Baranchikov, A.; Tyurin, A.; Kirdyankin, D.; Gavrichev, K.; Tuna, F.; Dobrokhotova, Z. *Polyhedron* **2017**, *122*, 184. $[Mn(NCCH_3)_6][Yb(O_2NO)_4]$ |
| IQUVAU | 2.228 Å | Schmucker, M. CCDC deposition number 2073096, **2021**. $[Mn(NCCH_3)_6](N(SO_2CF_3)_2)_2]\cdot CH_3CN$ |
| IQUVEY | 2.228 Å | Schmucker, M. CCDC deposition number 2073097, **2021**. $[Mn(NCCH_3)_6][Mn_2(CF_3COO)_6]\cdot 0.36CH_3CN$ |
| HASBUA | 2.249 Å | Lei, Z.; Clerac, R.; Heijboer, P.; Schmitt, W. *Angew, Chem. Int. Ed.* **2012**, *51*, 3007. $[Mn(NCCH_3)_6][Mn_{13}C_{32}H_{72}Cl_6O_{32}P_8]Cl_2\cdot 6CH_3CN\cdot 2.25H_2O$ |
| **Mean** | **2.228 Å/9 structures** | |

**Technetium(II)**

*Six-coordination, octahedral configuration*

| CSD code | *d*(Tc-N) | Reference, compound formula |
|---|---|---|
| TEQNAE | 2.062 Å | Cotton, F. A.; Haefner, S. C.; Sattelberger, A. P. *J. Am. Chem. Soc.* **1996**, 118, 5486. $[Tc(NCCH_3)_6](BF_4)_2$ |
| **Mean** | **2.062 Å/1 structure** | |

**Rhenium(II)**

*Six-coordination, octahedral configuration*

| CSD code | *d*(Rh-O) | Reference, compound formula |
|---|---|---|
| LADJEJ | 2.055 Å | Bolliger, R.; Meola, G.; Braband, H.; Blacque, O.; Siebenmann, L.; Nadeem, Q.; Alberto, R. *Inorg. Chem.* **2021**, 50, 17600. $[Re(NCCH_3)_6](CF_3SO_3)_2$ |
| **Mean** | **2.055 Å/1 structure** | |

**Iron(II)**

*Six-coordination, octahedral configuration*

| CSD code | *d*(Fe-O) | Reference, compound formula |
|---|---|---|
| NUQDEL | 2.144 Å | Brake, H.; Peresypkina, E.; Virovets, A. V.; Piesch, M.; Kremer, W.; Zimmermann, L.; Klimas, C.; Scheer, M. *Angew. Chem., Int. Ed.* **2020**, *59*, 16241. $[Fe(NCCH_3)_6][As_4I_{14}]$ |
| DEQDOV | 2.145 Å | Musgrave, R. A.; Hailes, R. L. N.; Schafer, A.; Russell, A. D.; Gates, P. J.; Manners, I. *Dalton Trans.* **2018**, *47*, 2759. $[Fe(NCCH_3)_6]B_{12}Cl_{12}$ |
| SUZRUC | 2.150 Å | Itazaki, M.; Ito, M.; Nakashima, S.; Nakazawa, H. *Dalton Trans.* **2016**, *45*, 1327. $[Fe(NCCH_3)_6][Fe(CO)_4(In(Br_3)_2]$ |
| NUBTOW | 2.151 Å | Rheingold, A. L.; Miller, J. S. CCDC deposition number 1961180, **2019**. $[Fe(NCCH_3)_6][FeBr_4]$ |
| NUQQAU | 2.151 Å | Brake, H.; Peresypkina, E.; Virovets, A. V.; Piesch, M.; Kremer, W.; Zimmermann, L.; Klimas, C.; Scheer, M. *Angew. Chem., Int. Ed.* **2020**, *59*, 16241. $[Fe(NCCH_3)_6][As_6I_8]$ |
| RAPXOY01 | 2.151 Å | Ito, M.; Itazaki, M.; Nakazawa, H. *ChemCatChem* **2016**, *8*, 3323. $[Fe(NCCH_3)_6](PF_6)_2$ |
| RAPXOY | 2.152 Å | Clegg, J. K.; Cremers, J.; Hogben, A. J.; Breiner, B.; Smulders, M. M. J.; Thoburn, J. D.; Nitschke, J. R. *Chem. Sci.* **2013**, *4*, 68. $[Fe(NCCH_3)_6](PF_6)_2$ |
| ACEYOW01 | 2.153 Å | Lutz, M.; Spek, A. L. ; Gosiewska, S. ; Klein Gebbink, R. J. M. ; van Koten, G. CCDC deposition number 235737, **2004**. $[Fe(NCCH_3)_6](SbF_6)_2$ |

| | | |
|---|---|---|
| SUZSAJ | 2.153 Å | Itazaki, M.; Ito, M.; Nakashima, S.; Nakazawa, H. *Dalton Trans.* **2016**, *45*, 1327. $[Fe(NCCH_3)_6][Fe(CO)_4(In(I_3)_2]$ |
| NOZZOU | 2.154 Å | Rheingold, A. L.; Miller, J. S. CCDC deposition number 1961020, **2019**. $[Fe(NCCH_3)_6][FeI_4]$ |
| ACEYOW | 2.154 Å | Brennessel, W. W.; Brooks, N. R. ; Mehn, M. P. ; Que Jr., L.; Young Jr., V. G. Acta Crystallogr., Sect. E. **2001**, *57*, m545. $[Fe(NCCH_3)_6](SbF_6)_2$ |
| DEQDIP | 2.155 Å | Musgrave, R. A.; Hailes, R. L. N.; Schafer, A.; Russell, A. D.; Gates, P. J.; Manners, I. *Dalton Trans.* **2018**, *47*, 2759. $[Fe(NCCH_3)_6](B(C_6F_5)_4)_2$ |
| SUZROW | 2.160 Å | Itazaki, M.; Ito, M.; Nakashima, S.; Nakazawa, H. *Dalton Trans.* **2016**, *45*, 1327. $[Fe(NCCH_3)_6][Fe(CO)_4(In(Cl_3)_2]$ |
| HIRTIL01 | 2.161 Å | Lutz, M.; Spek, A. L.; Gosiewska, S.; Klein Gebbink, R. J. M.; van Koten, G. CCDC deposition number 235634, **2004**. $[Fe(NCCH_3)_6][Cl_3FeOFeCl_3]$ |
| HMCIFE15 | 2.161 Å | Lavrent'ev, I. P.; Korableva, L. G.; Lavrent'eva, E. A.; Nifontova, G. A.; Khidekel, M. L.; Gusakovskaya, I. G.; Larkina, T. I.; Arutyunyan, L. D.; Filipenko, O. S.; Ponomarev, V. I.; Atovmyan, L. O. *Transition Met. Chem.* **1980**, *5*, 193. $[Fe(NCCH_3)_6][FeCl_4]$ |
| ZUKCEP | 2.161 Å | Wright, M. A. ; Wright, J. A. CCDC deposition number 1057431, **2015** $[Fe(NCCH_3)_6][Br_3FeOFeBr_3]$ |
| ZUKCEP01 | 2.161 Å | Wang, K. ; Prior, T. J. ; Hughes, D. L. ; Arbaoui, A. ; Redshaw, C. *Dalton Trans..* **2021**, *50*, 8057. $[Fe(NCCH_3)_6][Br_3FeOFeBr_3]$ |
| HIRTIL | 2.165 Å | Kuhn, N.; Kotowski, H.; Maichle-Moessmer, C.; Abram, U. *Z. Anorg. Allg. Chem.* **1998**, *624*, 1653. $[Fe(NCCH_3)_6][Cl_3FeOFeCl_3]$ |
| HMCIFE01 | 2.191 Å | Constant, G.; Daran, J.-C.; Jeannin, Y. *J. Organometal. Chem.* **1972**, *44*, 353. $[Fe(NCCH_3)_6][FeCl_4]$ |
| **Mean** | **2.155/18 structures** | |

**Ruthenium(II)**

*Six-coordination, octahedral configuration*

| CSD code | *d*(Ru-N) | Reference, compound formula |
|---|---|---|
| FEWRII | 2.013 Å | Brown, M.; Fontaine, X. L. R.; Greenwood, N. N.; Kennedy, J. D.; Thornton-Pett, M. *J. Chem. Soc., Dalton Trans.* **1987**, 1169. $[Ru(NCCH_3)_6][RuH_2(B_{10}H_{11})(C_6(CH_3)_6]_2$ |
| FILDEL | 2.022 Å | Underwood, C. C.; Stadelman, B. S.; Sleeper, M. L.; Brumaghim, J. L. *Inorg. Chim. Acta* **2013**, *405*, 470. $[Ru(NCCH_3)_6](BF_4)_2$ |
| FILDEL01 | 2.022 Å | Abbas, M. A.; McMillen, C. D.; Brumaghim, J. L. *Inorg. Chim. Acta* **2013**, *468*, 308. $[Ru(NCCH_3)_6](BF_4)_2$ |
| PUHSOB | 2.025 Å | Masland, J.; Diaz, J.; Eady, S.; Lobkovsky, E.; Larsen, A. *Acta Crystallogr., Sect. E* **2010**, *66*, m325. $[Ru(NCCH_3)_6](B_{11}CH_6Br_6)_2\cdot CH_3CN$ |

| | | |
|---|---|---|
| VIYMIZ | 2.025 Å | Zhilyaev, A. N.; Fomina, T. A.; Kuz'menko, I. V.; Katser, S. B.; Baranovskii, I. B. *Zh. Neorg. Khim.* **1990**, *35*, 2253. $[Ru(NCCH_3)_6](PF_6)_2$ |
| GEQPEZ | 2.028 Å | Haddow, M.; Russell, A.; Manners, I. CCDC deposition number 880469, **2012**. $[Ru(NCCH_3)_6](CF_3SO_3)_2$ |
| VARWIU | 2.028 Å | Luginbuhl, W.; Ludi, A.; Raselli, A.; Bürgi, H.-B. *Acta Crystallogr., Sect. C* **1989**, *45*, 1428. $[Ru(NCCH_3)_6](p\text{-}CH_3C_6H_4SO_3)_2\cdot 2H_2O$ |
| ACEYAI | 2.033 Å | Anzellotti, A.; Briceno, A. *Acta Crystallogr., Sect. E* **2001**, *57*, m538. $[Ru(NCCH_3)_6][ZnCl_4]\cdot 2.55H_2O$ |
| **Mean** | **2.025 Å/8 structures** | |

**Cobalt(II)**

*Six-coordination, octahedral configuration*

| CSD code | *d*(Co-N) | Reference, compound formula |
|---|---|---|
| XIFMUW | 2.085 Å | Mondal, A.; Durdevik, S.; Chamoreau, L.-M.; Journaux, Y.; Julve, M.; Lisnard, L.; Lescouezec, R. *Chem. Commun.* **2013**, *49*, 1181. $[Co(NCCH_3)_6][Co_{12}C_{150}H_{231}B_4F_4N_{37}O_{44}]\cdot 9CH_3CN\cdot 1.5H_2O$ |
| CAKTER | 2.103 Å | Keler, M.; Stammler, H.-G.; Neumann, B.; Roschenthaler, G.-V.; Hoge, B. *Inorg. Chem.* **2021**, *60*, 16466. $[Co(NCCH_3)_6][Ag(PF_2(CF_2(CF_3))_3)_3]\cdot 2CH_3CN$ |
| ESUCON | 2.107 Å | Wang, K.; Prior, T. J.; Hughes, D. L.; Arbaoui, A.; Redshaw, C. *Dalton Trans.* **2021**, *50*, 8057. $[Co(NCCH_3)_6][CoBr_3(NCCH_3)]_2\cdot 2CH_3CN$ |
| DAGRUB | 2.110 Å | Fernandez, I. I. L.; Donaldson, S.; Schipper, D. E.; Andleeb, S.; Whitmire, K. *Inorg. Chem.* **2016**, *55*, 11560. $[Co(NCCH_3)_6][Bi_4C_{12}H_{18}F_{30}O_{22}]$ |
| UMEKUV | 2.111 Å | Avdeeva, V. V.; Vologzhanina, A. V.; Ugolkova, E. A.; Minin, V. V.; Malinina, E. A.; Kuznetsov, N. T. *J. Solid State Chem.* **2021**, *296*, 121989. $[Co(NCCH_3)_6]B_{10}Cl_{10}$ |
| JOYPUI01 | 2.113 Å | Malkov, A. E.; Fomina, I. G.; Sidorov, A. A.; Aleksandrov, G. G.; Egorov, I. M.; Latosh, N. I.; Chupakhin, O. N.; Rusinov, G. L.; Rakitin, Y. V.; Novotortsev, V. M.; Ikorskii, V. N.; Eremenko, I. L.; Moiseev, I. I. *J. Mol. Struct.* **2003**, *656*, 207. $[Co(NCCH_3)_6][CoCl_3(NCCH_3)]_2\cdot 2CH_3CN$ |
| OCELIS | 2.113 Å | Parsons, S.; Tan, X.; Winpenny, R.; Wood, P. A. CCDC deposition number 248191, **2004**. $[Co(NCCH_3)_6][Gd(O_2NO)_5]$ |
| OFINAT | 2.113 Å | Hijazi, A. K.; Al Hmaideen, A.; Syukri, S.; Radhakrishnan, N.; Herdtweck, E.; Voit, B.; Kuhn, F. E. *Eur. J. Inorg. Chem.* **2008**, 2892. $[Co(NCCH_3)_6](B(C_6F_5)_4)_2$ |
| PUYCUK | 2.113 Å | Xue, T.; Vicic, D. A. *Organometallics* **2020**, *39*, 3715. $[Co(NCCH_3)_6](PF_6)_2$ |
| SURQAA | 2.113 Å | Saber, M. R.; Singh, M. K.; Dunbar, K. R. *Chem. Commun.* **2020**, *56*, 8492. $[Co(NCCH_3)_6](BF_4)_2$ |
| JOYPUI | 2.114 Å | Cotton, F. A.; Daniels, L. M.; Jordan IV, G. T.; Murillo, C. A. *Polyhedron* **1998**, *17*, 589. $[Co(NCCH_3)_6][CoCl_3(NCCH_3)]_2\cdot 2CH_3CN$ |

| | | |
|---|---|---|
| SIGMEA | 2.114 Å | Veith, M.; Godicke, B.; Huch, V. *Z. Anorg. Allg. Chem.* **1989**, *579*, 99. $[Co(NCCH_3)_6][SnCl_3]_2 \cdot 2CH_3CN$ |
| GUDTEH | 2.117 Å | Gavrikov, A. V.; Ilyukhin, A. B.; Belova, E. V.; Yapryntsev, A. D.; Dobrokhotova, Z. V.; Khrushcheva, A. V.; Efimov, N. N. *Ceramics Int.* **2020**, *46*, 13014. $[Co(NCCH_3)_6][Sm(O_2NO)_5]$ |
| JOYPUI02 | 2.117 Å | Winpenny, R.; Marshall, P.; Parsons, S.; Messenger, D. CCDC deposition number 278257, **2005**. $[Co(NCCH_3)_6][CoCl_3(NCCH_3)]_2 \cdot 2CH_3CN$ |
| GOJLEY | 2.123 Å | Ariyananda, P. W. G.; Guardino, E. T.; Rosenthal, J.; Yap, G. P. A. CCDC deposition number 865171, **2012**. $[Co(NCCH_3)_6][CoBr_4]$ |
| **Mean** | **2.112 Å/14 structures** | |

**Rhodium(III)**

*Six-coordination, octahedral configuration*

| CSD code | *d*(Rh-N) | Reference, compound formula |
|---|---|---|
| VENYAO | 1.987 Å | Prater, M. E.; Pence, L. E.; Clerac, R.; Finniss, G. M.; Campana, C.; Auban-Senzier, P.; Jerome, D.; Canadell, E.; Dunbar, K. R *J. Am. Chem. Soc.* **1999**, *121*, 8005. $[Rh(NCCH_3)_6](BF_4)_3$ |
| **Mean** | **1.987 Å/1 structure** | |

**Nickel(II)**

*Six-coordination, octahedral configuration*

| CSD code | *d*(Ni-N) | Reference, compound formula |
|---|---|---|
| XEQNEO | 2.051 Å | Bernardi, A.; Ciabatti, I.; Femoni, C.; Iapalucci, M. C.; Longoni, G.; Zacchini, S. *Dalton Trans.* **2013**, *42*, 407. $[Ni(NCCH_3)_6][Cu_2Ni_{29}C_{44}H_{14}N_4O_{32}] \cdot 4CH_3CN$ |
| SIGNIF | 2.059 Å | Veith, M.; Godicke, B.; Huch, V. *Z. Anorg. Allg. Chem.* **1989**, *579*, 99. $[Ni(NCCH_3)_6][Sn_2Cl_5][SnCl_3] \cdot 0.5CH_3CN$ |
| XENBIE | 2.065 Å | Kawazu, Y.; Hoke, H.; Yamada, Y.; Umecky, T.; Ozutsumi, K.; Takamuku, T. *Phys. Chem. Chem. Phys.* **2017**, *19*, 31335. $[Ni(NCCH_3)_6](N(SO_2CF_3)_2)_2$ |
| FUJVAH01 | 2.067 Å | Bougon, R.; Charpin, P.; Christe, K. O.; Isabey, J.; Lance, M.; Nierlich, M.; Vigner, J.; Wilson W. W. *Inorg. Chem.* **1988**, *27*, 1389. $[Ni(NCCH_3)_6][SbF_6]_2$ |
| OCELAK | 2.068 Å | Parsons, S.; Tan, X.; Winpenny, R.; Wood, P. A. CCDC deposition number 248070, **2004**. $[Ni(NCCH_3)_6][Gd(O_2NO)_5]$ |
| ACNNIZ | 2.070 Å | Søtofte, I.; Hazell, R. G. ; Rasmussen, S. E. *J. Acta Crystallogr., Sect. B* **1976**, *32*, 1692. $[Ni(NCCH_3)_6][ZnCl_4]$ |

| | | |
|---|---|---|
| SICSEF | 2.072 Å | Shmakova, A. A.; Akhmetova, M. M.; Volchek, V. V.; Romanova, T. E.; Korolkov, I.; Sheven, D. G.; Adonin, S. A.; Abramov, P. A.; Sokolov, M. N. *New. J. Chem.* **2018**, *42*, 7940. $[Ni(NCCH_3)_6]_2[NbW_{11}O_{40}]\cdot 1.7CH_3CN$ |
| XUGBIK | 2.072 Å | Pietikainen, J.; Maaninen, A.; Laitinen, R. S.; Oilunkaniemi, R.; Valkonen, J. *Polyhedron* **2002**, *21*, 1089. $[Ni(NCCH_3)_6][Te_2Cl_{10}]$ |
| FUJVAH | 2.075 Å | Leban, I.; Gantar, D.; Frlec, B.; Russell, D. R.; Holloway, J. H. *Acta Crystallogr., Sect. C* **1987**, *43*, 1888. $[Ni(NCCH_3)_6][SbF_6]_2$ |
| **Mean** | **2.067 Å/9 structures** | |

**Palladium(II)**

*Four-coordination, square-planar configuration*

| CSD code | *d*(Pd-N) | Reference, compound formula |
|---|---|---|
| KUXSIF | 1.956 Å | T.Gebauer, G.Frenzen, K.Dehnicke, K. *Z. Naturforsch., Teil B* **1992**, *47*, 1505. $[Pd(NCCH_3)_4]BF_4)_2$ |
| ZOYFUO | 1.968 Å | Massa, W; Wocadlo, S.; Dehnicke, K.; Gebauer, T. *Z. Krist. Cryst. Mater.* **1996**, *211*, 120. $[Pd(NCCH_3)_4][SnCl_5(NCCH_3)]_2$ |
| NUYWAF | 1.976 Å | v.Ahsen, B.; Bley, B.; Proemmel, S.; Wartchow, R.; Willner, H.; Aubke, F. *Z. Anorg. Allg. Chem.* **1992**, *47*, 1505. $[Pd(NCCH_3)_4](SbF_6)_2$ |
| **Mean** | **1.967 Å/3 structures** | |

**Platinum(II)**

*Four-coordination, square-planar configuration*

| CSD code | *d*(Pd-N) | Reference, compound formula |
|---|---|---|
| NUYVUY | 1.972 Å | v. Ahsen, B.; Bley, B.; Proemmel, S.; Wartchow, R.; Willner, H.; Aubke, F. *Z. Anorg. Allg. Chem.* **1992**, *47*, 1505. $[Pd(NCCH_3)_4](SbF_6)_2$ |
| **Mean** | **1.972 Å/1 structure** | |

**Copper(I)**

*Two-coordination, linaear fashion*

| CSD code | $d(Cu\text{-}N)_{eq}$ | Reference, compound formula |
|---|---|---|
| MIZBAY | 1.844 Å | Liang, H.-C.; Kim, E.; Incarvito, C. D.; Rheingold, A. L.; Karlin, K. D. *Inorg. Chem.* **2002**, *41*, 2209. $[Cu(NCCH_3)_2]B(C_6F_5)_4$ |
| **Mean** | **1.844 Å/1 structure** | |

*Three-coordination, triangular fashion (often distorted)*

| CSD code | $d$(Cu-N)$_{eq}$ | Reference, compound formula |
|---|---|---|
| IRUNIS | 1.933 Å | Malinina, E. A.; Zhizhin, K. Y; Mustyatsa, V. N.; Goeva, L. V.; Polyakova, I. N.; Kuznetsov, N. T. *Zh. Neorg. Khim.* **2003**, *48*, 1102. $[Cu(NCCH_3)_3][AgB_{10}H_{10}]$ |
| LIHYIN | 1.942 Å | Moussa, M. E.; Piesch, M.; Fleischmann, M.; Schreiner, A.; Seidl, M.; Scheer, M. *Dalton Trans*. **2018**, *47*, 16031. $[Cu(NCCH_3)_3][Cu(NCCH_3)_4][Al(OC(CF_3)_3)_4]_2{\cdot}2.98CH_2Cl_2$ |
| **Mean** | **1.938 Å/2 structures** | |

*Four-coordination, tetrahedral configuration,*

| CSD code | $d$(Cu-N)$_{eq}$+ $d$(Cu-N)$_{ax}$ | Reference, compound formula |
|---|---|---|
| EMUBOF | 1.922 Å | Demir, S.; Cepni, H. M.; Bilgin, N.; Holynska, M.; Yilmaz, F. *Polyhedron* **2016**, *115*, 236. $[Cu(NCCH_3)_4][Cu_3C_{14}H_9IN_2O_{10}]{\cdot}[Cu_3C_{14}H_{10}IN_2O_{10}]{\cdot}CH_3CN{\cdot}CH_3COOH{\cdot}8H_2O$ |
| GUHTAG | 1.967 Å | Becker, S.; Behrens, U.; Schindler, S. *Eur. J. Inorg. Chem.* **2015**, 2437. $[Cu(NCCH_3)_4][Cu_2Br_4]_n$ |
| USEVOF01 | 1.976 Å | He, H.; Sun. F.; Ma, S.; Zhu, G. *Inorg. Chem.* **2016**, *55*, 9071. $[Cu(NCCH_3)_4][Cu_3C_{14}H_{10}IN_2O_{10}]2I{\cdot}(CH_3)_2NCHO$ |
| HIWSIS | 1.984 Å | Dehnhardt, N.; Both, A.; Heine, J. *Dalton Trans.* **2019**, *48*, 5222. $[Cu(NCCH_3)_4]_4[Sb_{10}I_{34}]$ |
| TACNCU11 | 1.985 Å | Cao, B.; Wei, Y.; Shi, M. *Org. Chem. Front.* **2018**, *5*, 423. $[Cu(NCCH_3)_4]ClO_4$ |
| NAQMUN02 | 1.986 Å | Clegg, W.; Harrington, P. W. CCDC deposition number 2051675, **2020**. $[Cu(NCCH_3)_4]BF_4$ |
| WAWQAQ01 | 1.987 Å | Peloquin, A. J. CCDC deposition number 2165642, **2022**. $[Cu(NCCH_3)_4]I_3{\cdot}I_2$ |
| HIWSOY | 1.988 Å | Dehnhardt, N.; Both, A.; Heine, J. *Dalton Trans.* **2019**, *48*, 5222. $[Cu(NCCH_3)_4][Sb_2I_7]$ |
| HIWSUE | 1.989 Å | Dehnhardt, N.; Both, A.; Heine, J. *Dalton Trans.* **2019**, *48*, 5222. $[Cu(NCCH_3)_4]_4[Sb_6I_{22}]{\cdot}2CH_3CN$ |
| NIXSIW | 1.989 Å | Bach, A.; Hoyer, M.; Hartl. H. *Z. Naturforsch., Teil B* **1997**, *52*, 1497. $[Cu(NCCH_3)_4]_4[Cd_6I_{16}]{\cdot}1.5CH_3CN$ |
| QIFXIO | 1.989 Å | Zhang, C.-P.; Vicic, D. A. *Organometallics* **2012**, *31*, 7812. $[Cu(NCCH_3)_4](CF_3O)$ |
| TACNCU | 1.989 Å | Csöregh, I.; Kierkegaard, P.; Norrestam, R. *Acta Crystallogr., Sect. C* **1975**, *31*, 314. $[Cu(NCCH_3)_4]ClO_4$ |
| TACNCU01 | 1.989 Å | Rheingold, A. L. CCDC deposition number 155071, **2000**. $[Cu(NCCH_3)_4]ClO_4$ |
| TACNCU13 | 1.989 Å | Rukk, N. S.; Buzanov, G. A.; Kabernik, N. S.; Kuzmina, L. G.; Davydova, G. A.; Efimov, N. N.; Shamsiev, R. S.; Belus, S. K.; Kozhukhova, E. I.; Retivov, V. M.; Ivanova, T. V. *Mendeleev Commun.* **2022**, *32*, 123. $[Cu(NCCH_3)_4]ClO_4$ |
| TIWRIA | 1.989 Å | Chambers, R. D.; Gray, W. K.; Vaughan, J. F. S.; Medebielle, M.; Korn, S. R.; Batsanov, A. S.; Lehmann, C. W.; Howard, J. A. K. *J. Chem. Soc., Perkin Trans. 1* **1997**, 135. $[Cu(NCCH_3)_4](C_5(CF_3)_5)$ |
| YIBYOZ | 1.989 Å | M.Kleinwachter, L.Vendier, C.Dinoi, M.Etienne, M. *Dalton Trans.* **2013**, *42*, 10102. $[Cu(NCCH_3)_4]BF_4$ |
| NAQMUN04 | 1.900 Å | Fronczek, F. R. CCDC deposition number 2172363, **2022**. $[Cu(NCCH_3)_4]BF_4$ |

| | | |
|---|---|---|
| NUFSEN | 1.990 Å | Fielden, J.; Long, D.-L.; Cronin, L.; Kogerler, P. *Polyhedron* **2009**, *28*, 2803. $[Cu(NCCH_3)_4]_2[Cu_2Mo_6C_8H_{12}N_4O_{26}]$ |
| TACNCU03 | 1.990 Å | Bolte, M. ; Scheuermann, S. CCDC deposition number 717648, **2009**. $[Cu(NCCH_3)_4]ClO_4$ |
| TACNCU09 | 1.990 Å | Yao, B.; Wang, Z.-L.; Zhang, H.; Wang, D.-X.; Zhao, L.; Wang, M.-X. *J. Org. Chem.* **2012**, *77*, 3336. $[Cu(NCCH_3)_4]ClO_4$ |
| TACNCU06 | 1.991 Å | Hao, H.-G.; Zheng, X.-D.; Lu, T.-B. *Angew. Chem, Int. Ed.* **2010**, *49*, 8148. $[Cu(NCCH_3)_4]ClO_4$ |
| TACNCU07 | 1.991 Å | Fallis, I. A.; Coles, S. J.; Hursthouse, M. B. *Univ. Southampton, Cryst. Struct. Report Archive* **2001**, 1044. $[Cu(NCCH_3)_4]ClO_4$ |
| OYAMAF | 1.993 Å | Haupt, A.; Duvinage, D. Lork, E.; Ponomarenko, M.; Roschenthaler, G.-V. *Angew, Chem., Int. Ed.* **2021**, *60*, 17866. $[Cu(NCCH_3)_4](SF_5O)\cdot CH_3CN$ |
| TACNCU10 | 1.993 Å | Brotherton, W. S.; Clark, R. J.; Zhu, L. *J. Org. Chem.* **2012**, *77*, 6443. $[Cu(NCCH_3)_4]ClO_4$ |
| TACNCU12 | 1.993 Å | Kiriakopoulos, R.; Boyle, P. D.; Dawe, L. N. CCDC deposition number 1968554, **2019**. $[Cu(NCCH_3)_4]ClO_4$ |
| PAGSEZ | 1.994 Å | Gorbachev, V.; Trapp, N.; Chen, P. CCDC deposition number 2038420, **2020**. $[Cu(NCCH_3)_4]PF_6$ |
| HIWSEO | 1.994 Å | Dehnhardt, N.; Both, A.; Heine, J. *Dalton Trans.* **2019**, *48*, 5222. $[Cu(NCCH_3)_4]_4[Sb_7I_{25}]\cdot CH_3CN$ |
| HUJWIU | 1.994 Å | Pineda, E. M.; Heesing, C.; Tuna, F.; Zheng, Y.-Z.; McInnes, E. J. L.; Schnack, J; Winpenny, R. E. P. *Inorg. Chem.* **2015**, *54*, 6331. $[Cu(NCCH_3)_4][Cu_3Tb_9C_{99}H_{189}O_{55}P_6]$ |
| IGIWAZ | 1.994 Å | Nouch, R.; Woodward, S.; Willcox, D.; Robinson, D.; Lewis, W. *Organometallics* **2020**, *39*, 834. $[Cu(NCCH_3)_4]_2[Cu_4(CF_3COO)_{10}]\cdot C_2H_2Cl_2$ |
| NAQMUN | 1.994 Å | Jones, P. J.; Crespo, O. *Acta Crystallogr., Sect. C* **1998**, *54*, 18. $[Cu(NCCH_3)_4]BF_4$ |
| NAQMUN01 | 1.994 Å | Bats, J. W.; Kretz, T.; Lerner, H.-W. *Acta Crystallogr., Sect. C* **2009**, *65*, m94. $[Cu(NCCH_3)_4]BF_4$ |
| QINTEO | 1.994 Å | Royappa, A. T.; Stepherson, J. R.; Vu, O. D.; Royappa, A. D.; Stern, C. L.; Müller, P. |
| TACNCU02 | 1.994 Å | Bowmaker, G. A.; Gill, D. S.; Skelton, B. W.; Somers, N.; White, A. H. *Z. Naturforsch., Teil B* **2004**, *59*, 1307. $[Cu(NCCH_3)_4]ClO_4$ |
| LIHYOT | 1.995 Å | Moussa, M. E.; Piesch, M.; Fleischmann, M.; Schreiner, A.; Seidl, M.; Scheer, M. *Dalton Trans.* **2018**, *47*, 16031. $[Cu(NCCH_3)_4][Al(OC(CF_3)_3)_4]\cdot CH_3CN$ |
| LIMXUA03 | 1.995 Å | Lutz, M.;Tazelaar, J.C. G. J.; Slootweg, J. C.; Lammertsma, K. CCDC deposition number 815342, **2011**. $[Cu(NCCH_3)_4]PF_6\cdot CH_3CN$ |
| TACNCU08 | 1.995 Å | Sarma, M.; Mondal, B. *Inorg. Chem.* **2011**, *50*, 3206. $[Cu(NCCH_3)_4]ClO_4$ |
| HUJVUF | 1.996 Å | Pineda, E. M.; Heesing, C.; Tuna, F.; Zheng, Y.-Z.; McInnes, E. J. L.; Schnack, J; Winpenny, R. E. P. *Inorg. Chem.* **2015**, *54*, 6331. $[Cu(NCCH_3)_4][Cu_3Dy_9C_{99}H_{189}O_{55}P_6]$ |
| LIMXUA01 | 1.996 Å | Dakin, L. A.; Ong, P. C.; Panek, J. S.; Staples, R. J.; Stavropoulos, P. *Organometallics* **2000**, *19*, 2896. $[Cu(NCCH_3)_4]PF_6\cdot CH_3CN$ |

| | | |
|---|---|---|
| NUFSIR | 1.996 Å | Fielden, J.; Long, D.-L.; Cronin, L.; Kogerler, P. *Polyhedron* **2009**, *28*, 2803. $[Cu(NCCH_3)_4]_4[Mo_8O_{26}]]\cdot 2CH_3CN$ |
| LIMXUA02 | 1.999 Å | Bak, J. M.; Effendy, S.; Grabowsky, S.; Lindoy, L. F.; Price, J. R.; Skelton, B. W.; White, A. H. *CrystEngComm* **2013**, *15*, 1125. $[Cu(NCCH_3)_4]PF_6\cdot CH_3CN$ |
| TACNCU04 | 1.999 Å | Beloglazkina, E. K.; Shimorsky, A. V.; Mazhuga, A. G.; Shilova, O. V.; Tafeenko, V. A.; Zyk , N. V. *Zh. Obshch. Khim.* **2009**, *79*, 1147. $[Cu(NCCH_3)_4]ClO_4$ |
| TIRDOO | 1.999 Å | Chai, W.-X.; Wu, L.-M.; Li, J.-Q.; Che, L. *Inorg. Chem.* **2007**, *46*, 8698. $[Cu(NCCH_3)_4][BiCuI_5]$ |
| ENEPOC | 2.000 Å | Knaust, J. M.; Knight, D. A.; Keller, S. W. *J. Chem. Cryst.* **2003**, *33*, 813. $[Cu(NCCH_3)_4]CF_3SO_3$ |
| HUJVOZ | 2.000 Å | Pineda, E. M.; Heesing, C.; Tuna, F.; Zheng, Y.-Z.; McInnes, E. J. L.; Schnack, J; Winpenny, R. E. P. *Inorg. Chem.* **2015**, *54*, 6331. $[Cu(NCCH_3)_4][Cu_3Er_9C_{99}H_{189}O_{55}P_6]$ |
| BANMOU | 2.001 Å | Gill, D. S.; Singh, R.; Rana, D. S.; Wagler, J.; Kroke, E. *Z. Naturforsch., Teil B* **2011**, *66*, 1042. $[Cu(NCCH_3)_4]HSO_4$ |
| DAGFAV | 2.001 Å | Fernandez, I. I. L.; Donaldson, S.; Schipper, D. E.; Andleeb, S.; Whitmire, K. *Inorg. Chem.* **2016**, *55*, 11560. $[Cu(NCCH_3)_4][Bi_2C_{10}F_{15}O_{11}\cdot CF_3COOH$ |
| IWAHAQ01 | 2.001 Å | Brooks, N. R.; Schaltin, S.; Van Hecke, K.; Van Meervelt, L.; Binnemans, K.; Fransaer, J. *Chem. Eur. J.* **2011**, *17*, 5054. $[Cu(NCCH_3)_4](N(SO_2CF_3)$ |
| ENEPOC01 | 2.002 Å | Irangu, J.; Ferguson, M. J.; Jordan, R. B. *Inorg. Chem.* **2005**, *44*, 1619. $[Cu(NCCH_3)_4]CF_3SO_3$ |
| HUJWAM | 2.003 Å | Pineda, E. M.; Heesing, C.; Tuna, F.; Zheng, Y.-Z.; McInnes, E. J. L.; Schnack, J; Winpenny, R. E. P. *Inorg. Chem.* **2015**, *54*, 6331. $[Cu(NCCH_3)_4][Cu_3Gd_9C_{99}H_{189}O_{55}P_6]$ |
| BOSYEQ | 2.006 Å | Yan-Ling Li, Y.-L.; Wang, J.; Luo, P.; Ma, X.-H.; Dong, X.-Y.; Wang, Z.-Y.; Du, C.-X.; Zang, S.-Q.; Mak, T. C. W. *Adv. Sci.* **2019**, 1900833. $[Cu(NCCH_3)_4]B_{19}C_4H_{20}S\cdot[Cu(NCCH_3)_4]\cdot CH_3CN$ |
| LIMXUA | 2.006 Å | Black, J. R.; Levason, W.; Webster, M. *Acta Crystallogr., Sect. C* **1995**, *51*, 623. $[Cu(NCCH_3)_4]PF_6\cdot CH_3CN$ |
| EDEJIG | 2.010 Å | Llewellyn, D. B.; Adamson, D.; Arndtsen, B. A. *Org. Lett.* **2000**, *2*, 4165. $[Cu(NCCH_3)_4](B(O_2C_{20}H_5)_2)\cdot[Cu(CH_3CN)(B(O_2C_{20}H_5)_2)]\cdot CH_3CN$ |
| VEHXAJ | 2.010 Å | Fielden, J.; Quasdorf, K.; Cronin, L.; Kogerler, P. *Dalton Trans.* **2012**, *41*, 9876. $H_3[Cu(NCCH_3)_4][Mo_{12}F_4O_{46}P_4]\cdot 4CH_3CN\cdot 32H_2O$ |
| OQIZUL | 2.011 Å | Tahli, A.; Elshaarawy, R. F. M.; Koc, U.; Kautz, A. C.; Janiak, C. *Polyhedron* **2016**, *17*, 579. $[Cu(NCCH_3)_4][Cu_3C_{22}H_{14}NO_{15}]$ |
| TEQSUE | 2.013 Å | Zhang, Y.; Sun, W.; Freund, C.; Santos, A. M.; Herdtweck, E.; Mink, J.; Kuhn, F. E. *Inorg. Chim. Acta* **2006**, *359*, 4723. $[Cu(NCCH_3)_4]B(C_6F_5)_4$ |
| HUJWEQ | 2.057 Å | Pineda, E. M.; Heesing, C.; Tuna, F.; Zheng, Y.-Z.; McInnes, E. J. L.; Schnack, J; Winpenny, R. E. P. *Inorg. Chem.* **2015**, *54*, 6331. $[Cu(NCCH_3)_4][Cu_3Ho_9C_{99}H_{189}O_{55}P_6]$ |

**Mean 1.995 Å/54 structures**

**Copper(II)**

*Six-coordination, centrosymmetric Jahn-Teller distorted octahedral fashion,*

| CSD code | $d$(Cu-N)$_{eq}$+ $d$(Cu-N)$_{ax}$ | Reference, compound formula |
|---|---|---|
| NIHCIR | 1.993 + 2.386 Å | Hijazi, A. K.; Yeong, H. Y.; Zhang, Y.; Herdtweck, E.; Nuyken, O.; Kuhn, F. E. *Macromol. Rapid Commun.* **2007**, *28*, 670. $[Cu(NCCH_3)_6](B(C_6F_4)_2$ |
| XAYFAG | 2.001 + 2.370 Å | Rabeah, J.; Stosser, R.; Jiao, H.; Troyanov, S. I.; Radnik, J.; Dwelk, H.; Meisel, M.; Bruckner, A. *Chem. Eur. J.* **2012**, *18*, 6433. $[Cu(NCCH_3)_6]F_2{\cdot}2[V_4C_{24}H_{20}O_{18}P_4]{\cdot}2CH_3CN$ |

**Mean 1.997 + 2.378 Å/2 structures**

**Silver(I)**

*Two-coordination, linear configuration*

| CSD code | $d$(Ag-N)$_{eq}$ | Reference, compound formula |
|---|---|---|
| FILTEA | 2.082 Å | Zhang, Y.; Santos, A. M.; Herdtweck, E.; Mink, J.; Kuhn, F. E. *New J. Chem.* **2005**, *29*, 366. $[Ag(NCCH_3)_2](B(3,5\text{-}C_6H_4(CF_3)_2)_4$ |
| ZOLKUJ | 2.085 Å | Monticelli, M.; Baron, M.; Tubaro, C.; Bellemin-Laponnaz, S.; Graiff, C.; Bottaro, G.; Armelao, L.; Orian, L. *ACS Omega* **2019**, *4*, 4192. $[Ag(NCCH_3)_4][Au_2C_{34}F_{42}N_{10}](PF_6)_3{\cdot}(C_2H_5)_2O$ |
| EWIYUG | 2.096 Å | Pliquett, D.; Schulz, P. S.; Heinemann, F. W.; Bause, A.; Wasserscheid, P. *Dalton Trans.* **2016**, *18*, 28242. $[Ag(NCCH_3)_2](PF_3(CF(CF_3)_3)$ |
| COWFIG | 2.105 Å | Tang, Y.; Yu, B. *Eur. J,. Inorg. Chem.* **2019**, 107. $[Ag(NCCH_3)_2]_2[Ag(N(SO_2CF_3)_2)_2]$ |
| EQEQOH | 2.110 Å | Raducan, M.; Rodriquez-Escrich, C.; Cambeiro, X. C.; Escudero-Adan, E. C.; Pericas, M. A.; Echavarren, A. M. *Chem. Commun.* **2011**, *47*, 4893. $[Ag(NCCH_3)_2]SbF_6$ |
| RIQYAU | 2.123 Å | Louise M.; Heinekey, G. D. M. CCDC deposition number 1882471, **2018**. $[Ag(NCCH_3)_2]BF_4$ |
| KADCOI | 2.150 Å | Ahmad, R.; Hardie, M. J. *Cryst. Growth Des.* **2003**, *3*, 493. $[Ag(NCCH_3)_2][CoB_{18}C_4H_{22}]$ |
| AWAJAK | 2.151 Å | McGlone, T.; Streb, C.; Busquets-Fite, M.; Yan, J.; Gabb, D.; Long, D.-L.; Cronin, L. *Cryst. Growtrh Des.* **2011**, *11*, 2471. $[Ag(NCCH_3)_2]_2[Ag_2W_{10}(C_{12}H_{18}N_6O_{32}S_2)_2]$ |
| XOCGOL | 2.153 Å | Karpova, E. V.; Boltalin, A. I.; Korenev, Y. M.; Zakharov, M. A.; Troyanov, S. I. *Koord. Khim.* **2001**, *28*, 311. $[Ag(NCCH_3)_4][Ag_2(CF_3COO)_3]$ |
| AWAJEO | 2.195 Å | McGlone, T.; Streb, C.; Busquets-Fite, M.; Yan, J.; Gabb, D.; Long, D.-L.; Cronin, L. *Cryst. Growtrh Des.* **2011**, *11*, 2471. $[Ag(NCCH_3)_2]_2[Ag_2W_{10}(C_{12}H_{18}N_6O_{32}S_2)_2]$ |

| | | |
|---|---|---|
| XAHKEY | 2.237 Å | McGlone, T.; Streb, C.; Long, D.-L.; Cronin, L. *Adv. Mater.* **2010**, *22*, 4275. $[Ag(NCCH_3)_4][Ag(NCCH_3)_2][Ag_4W_{19}C_{16}H_{28}N_8O_{62}]\cdot 6CH_3CN$ (bent coordination) |
| **Mean** | **2.117 Å/9 structures** | |

*Three-coordination, triangular fashion (often distorted)*

| CSD code | $d$(Ag-N)$_{eq}$ | Reference, compound formula |
|---|---|---|
| YUJKAQ | 2.214 Å | Wolper, C.; Rodriguez-Gimeno, A.; Iborra, K. C.; Kuhn, H.; Luttig, A. K.; Moll, S.; Most, C.; Freytag, M.; Dix, I.; Jones, P. G.; Blaschette, A. *Z. Naturforch., Teil B* **2009**, *64*, 952. $[Ag(NCCH_3)_3][Ag(C_{12}H_8F_2NO_4S_2)_2]\cdot CH_3CN$ |
| WIJQAI | 2.215 Å | J.K.Jabor, R.Stosser, Nguyen Huu Thong, B.Ziemer, M.Meisel, M. Angew. Chem., Int. Ed. 2007, 46, 6354. $[Ag(NCCH_3)_3]F\cdot[V_4P_4O_8(C_6H_5)_4]$ |
| SULNAR | 2.236 Å | Shmakova, A. A.; Berezin, A. S.; Abramov, P. A.; Sokolov, M. N. *Inorg. Chem.* **2020**, *59*, 1853. $[Ag(NCCH_3)_4]_2[Ag(NCCH_3)_3]_2[NbW_{11}PO_{40}]$ |
| KEWWER | 2.252 Å | Clegg, W.; Harrington, R. W. CCDC deposition number 1836903, **2018**. $[Ag(NCCH_3)_3]_2[Ag(NC_6H_4N)_2][Mo_{12}PO_{40}]$ |
| SAPRIM | 2.256 Å | McGlone, T.; Thiel, J.; Streb, C.; Long, D.-L.; Cronin, L. *Chem. Commun.* **2012**, *48*, 359. $[Ag(NCCH_3)_3]_3[H_3V_{10}O_{28}]\cdot CH_3CN$ |
| QUTNOI | 2.279 Å | Rhule, J. T.; Neiwert, W. A.; Hardcastle, K. I.; Do, B. T.; Hill, C. L. *J. Am. Chem. Soc.* **2001**, *123*, 12101. $[Ag(NCCH_3)_3]_2Ag_{2.4}[Ag_6Mo_{24}C_{20}H_{30}N_2O_{80}P_2](NO_3)_{0.4}\cdot 1.3CH_3CN\cdot 1.5H_2O$ |
| **Mean** | **2.242 Å/6 structures** | |

*$[Ag_2(NCCH_3)_2]$ dimer*

| CSD code | $d$(Ag-N)/$d$(Ag···Ag) | Reference, compound formula |
|---|---|---|
| YAWCOR | 2.251 Å<br>3.548 Å | Peryshkov, D. V.; Strauss, S. H. *Inorg. Chem.* **2017**, *56*, 4072. $[(CH_3CN)_2AgCH_3CNAg(NCCH_3)_2]$ |

*Four-coordination, tetrahedral fashion (often distorted)*

| CSD code | $d$(Cu-N) | Reference, compound formula |
|---|---|---|
| GUHMOL | 2.244 Å | Tsang, C.-T.; Sun, J.; Xie, Z. *J. Organometal. Chem.* **2000**, *613*, 99. $[Ag(NCCH_3)_4][CoB_{18}C_4H_{16}Br_6]$ |
| WAKLUP | 2.255 Å | Blaschette, A.; Jones, P. G.; Hamman, T.; Naveke, M.; Schomburg, D.; Cammenga, H. K.; Epple, M.; Steppuhn, I. *Z. Anorg. Allg. Chem.* **1993**, *619*, 912. $[Ag(NCCH_3)_4][Ag(N(SO_2CH_3)_2)_2]$ |

| | | |
|---|---|---|
| MUTGUD | 2.259 Å | Ang, H.-G.; Fraenk, W.; Karaghiosoff, K.; Klapotke, T. M.; Mayer, P.; Noth, N.; Sprott, J.; Warchhold, M. *Z. Anorg. Allg. Chem.* **2002**, *628*, 2894. $[Ag(NCCH_3)_4][Ag_3N_{12}O_{16}]$ |
| CETMOD | 2.261 Å | Nilsson, K.; Oskarsson, Å. *Acta Chem. Scand., Ser. A* **1984**, *38*, 79. $[Ag(NCCH_3)_4]ClO_4$ |
| FIXMIJ02 | 2.264 Å | Parsons, S.; McCormack, C.; Bailey, P. CCDC deposition number 1419678, **2015**. $[Ag(NCCH_3)_4]BF_4$ |
| CETMOD01 | 2.266 Å | Jones, P. G.; Bembenek, E. *Z. Krist. Cryst. Mater.* **1993**, *208*, 213. $[Ag(NCCH_3)_4]ClO_4$ |
| FIXMIJ01 | 2.267 Å | Müller-Bunz, H.; Farrel, K.; Albrech, M. CCDC deposition number 1029404, **2014**. $[Ag(NCCH_3)_4]BF_4$ |
| CETMOD02 | 2.268 Å | Bak, J. M.; Effendy; Grabowsky, S.; Lindoy, L. F.; Price, J. R.; Skelton, B. W.; White, A. H. *CrystEngComm* **2013**, *15*, 1125. $[Ag(NCCH_3)_4]ClO_4$ |
| YOLCUZ | 2.268 Å | Goettel, J. T.; Turnbull, D.; Gerken, M. *J. Flourine Chem.* **2015**, *174*, 8. $[Ag(NCCH_3)_4]$ $[ReF_4O_2]\cdot 2CH_3CN$ |
| FIXMIJ | 2.269 Å | Aly, A. A. M.; Walfort, B.; Lang, H. *Z. Kristallogr.-New Cryst. Struct.* **2004**, *219*, 489. $[Ag(NCCH_3)_4]BF_4$ |
| WUZJUY | 2.270 Å | Zhang, Y.; Liu, J.; Duttwyler, S. *Eur. J. Inorg. Chem.* **2015**, 5158. $[Ag(NCCH_3)_4]B_{12}H_{11}(NH_3)\cdot 3CH_3CN$ |
| MIXFIK | 2.272 Å | Dortbudak, C.-C.; Lux, K.; Kornath, A. *Z. Naturforsch., Teil B* **2014**, *69*, 373. $[Ag(NCCH_3)_4](FSO_3)$ |
| XASBUQ | 2.274 Å | Lutz, M.; Spannring, P.; Gebbink, R. J. M. K. CCDC deposition number 859179, **2011**. $[Ag(NCCH_3)_4]PF_6$ |
| XASBUQ01 | 2.276 Å | Bak, J. M.; Effendy; Grabowsky, S.; Lindoy, L. F.; Price, J. R.; Skelton, B. W.; White, A. H. *CrystEngComm* **2013**, *15*, 1125. $[Ag(NCCH_3)_4]PF_6$ |
| KADDEZ | 2.280 Å | Ahmad, R.; Hardie, M. J. *Cryst. Growth. Des.* **2003**, *3*, 493. $[Ag(NCCH_3)_4]_2[CoB_{18}C_{27}H_{30}]\cdot C_{27}H_{30}O_6\cdot 0.25CH_3CN$ |
| XAHKEY | 2.281 Å | McGlone, T.; Streb, C.; Long, D.-L.; Cronin, L. *Adv. Mater.* **2010**, *22*, 4275. $[Ag(NCCH_3)_4][Ag(NCCH_3)_2][Ag_4W_{19}C_{16}H_{28}N_8O_{62}]\cdot 6CH_3CN$ |
| EWIYOA | 2.285 Å | Pliquett, D.; Schulz, P. S.; Heinemann, F. W.; Bause, A.; Wasserscheid, P. *Phys. Chem. Chem. Phys.* **2016**, *18*, 28242. $[Ag(NCCH_3)_4](PF_3(CF_2(CF_3))_3)$ |
| EFACUO01 | 2.288 Å | Khan, E. S. T.; Waldbusser, A. L.; Carrasco, M. C.; Pourhadi, H.; Hematian, S. *Dalton Trans.* **2021**, *50*, 7433. $[Ag(NCCH_3)_4]B(C_6F_5)_4$ |
| IKIQOK | 2.291 Å | He, X.; Liu, H.-X.; Zhao, L. *Chem. Commun.* **2016**, *52*, 5682. $[Ag(NCCH_3)_4]_2[AgC_{38}H_{12}F_{30}N_4O_{20}]$ |
| EFAQOU | 2.293 Å | Kim, E. H.; Lee, H. M.; Jeong, M. S.; Ryu, J. Y.; Lee, J.; Lee, B. Y. *ACS Omega* **2017**, *2*, 765. $[Ag(NCCH_3)_4]B(C_6F_5)_4$ |
| OPARED | 2.298 Å | Dang, D.-B.; Gao, H.; Bai, Y.; Hu, X.-F.; Yang, F.; Chen, Y.; Niu, J.-Y. *Inorg. Chem. Commun.* **2010**, *13*, 37. $[Ag(NCCH_3)_4][Ag_3Mo_{12}SiC_{16}H_{24}N_8O_{40}]$ |
| SULNAR | 2.311 Å | Shmakova, A. A.; Berezin, A. S.; Abramov, P. A.; Sokolov, M. N. *Inorg. Chem.* **2020**, *59*, 1853. $[Ag(NCCH_3)_4]_2[Ag(NCCH_3)_3]_2[NbW_{11}PO_{40}]$ |
| YAWCUX | 2.345 Å | Peryshkov, D. V.; Strauss, S. H. *Inorg. Chem.* **2017**, *56*, 4072. $[Ag(NCCH_3)_4]B_{12}F_{12}$ |
| **Mean** | **2.275 Å/22 structures** | |

**Gold(I)**

*Two-coordination, linear configuration*

| CSD code | $d$(Au-N)$_{eq}$ | Reference, compound formula, |
|---|---|---|
| PANCUC | 1.936 Å | Willner, H.; Schaebs, J.; Hwang, G.; Mistry, F.; Jones, R.; Trotter, J.; Aubke, F. *J. Am. Chem. Soc.* **1992**, *114*, 8972. $[Au(NCCH_3)_2]SbF_6$ |
| ROQQEV | 1.960 Å | Shehadi, I.; Abla, F.; Wakefield, B.; Reibenspies, J.; Arooj, M.; Mohamed, A. A. *Res. Chem. Intermed.* **2020**, *46*, 593. $[Au(NCCH_3)_2][AuCl_4]$ |
| UWISAW | 1.962 Å | Engesser, T. A.; Friedmann, C.; Martens, A.; Kratzert, D.; Malinowski, P. J.; Krossing, I. *Chem. Eur. J.* **2016**, *22*, 15085. $[Au(NCCH_3)_2][GaCl_4]$ |
| PITMOX | 1.966 Å | Jenne, C.; Wegener, B. *Z. Anorg. Allg. Chem.* **2018**, *644*, 1123. $[Au(NCCH_3)_2](B_{12}Cl_{11}(NH(CH_3)_2)$ |
| UNISEA | 1.967 Å | Engesser, T. A.; Friedmann, C.; Martens, A.; Kratzert, D.; Malinowski, P. J.; Krossing, I. *Chem. Eur. J.* **2016**, *22*, 15085. $[Au(NCCH_3)_2][Al(OC(CF_3)_3)_4]$ |
| UNISOK | 1.970 Å | Engesser, T. A.; Friedmann, C.; Martens, A.; Kratzert, D.; Malinowski, P. J.; Krossing, I. *Chem. Eur. J.* **2016**, *22*, 15085. $[Au(NCCH_3)_2][Au(NCB(CF_3)_3]$ |
| **Mean** | **1.960 Å/6 structures** | |

**Zinc(II)**

*Four-coordination, tetrahedral configuration*

| CSD code | $d$(Zn-N) | Reference, compound formula |
|---|---|---|
| USALUW | 1.976 Å | Rach, S. F.; Herdtweck, E.; Kuhn, F. E. *J. Organomet. Chem.* **2011**, *696*, 1817. $[Zn(NCCH_3)_4](B(C_6F_5)_4)_2$ |
| DAMQAK | 1.999 Å | Yang, H.-L.; Yang, F.; Zhu, H.-L. *Z. Kristallogr.-New Struct.* **2004**, *219*, 349. $[Zn(NCCH_3)_4]SO_4$ |
| **Mean** | **1.988 Å/2 structures** | |

*Six-coordination, octahedral configuration*

| CSD code | $d$(Zn-N) | Reference, compound formula |
|---|---|---|
| UWEMIV | 2.132 Å | Groutchik, K.; Jaiswal, K.; Dobrovetsky, R. *Org. Biomol. Chem.* **2021**, *19*, 5544. $[Zn(NCCH_3)_6]B_{12}I_{12}$ |
| JEPKEV | 2.133 Å | Akkus, O. N.; Decken, A.; Knapp, C.; Passmore, J. *J. Chem. Cryst.* **2006**, *36*, 321. $[Zn(NCCH_3)_6][AsF_6]_2$ |
| **Mean** | **2.133 Å/2 structures** | |

**Aluminum(III)**

*Six-coordination, octahedral configuration*

| CSD code | *d*(Al-N) | Reference, compound formula |
|---|---|---|
| REVSOA | 1.932 Å | Babian-Kibala, E.; Chen, H.; Cotton, F. A.; Daniels, L. M.; Falvello, L. R.; Schmid, G.; Yao, Z. *Inorg. Chim. Acta* **1996**, *250*, 359. $[Al(NCCH_3)_6][TaCl_6]_3 \cdot 3CH_3CN$ |
| REVSIU | 1.943 Å | Babian-Kibala, E.; Chen, H.; Cotton, F. A.; Daniels, L. M.; Falvello, L. R.; Schmid, G.; Yao, Z. *Inorg. Chim. Acta* **1996**, *250*, 359. $[Al(NCCH_3)_6][NbCl_6]_3 \cdot 3CH_3CN$ |
| RIWDUW | 1.954 Å | Favier, F.; Pascal, J. L.; Belin, C.; Tillard-Charbonnel, M. *Acta Crystallogr., Sect. C* **1997**, *53*, 1234. $[Al(NCCH_3)_6][Te_3Cl_{15}]_3 \cdot CH_3CN$ |
| REVSUG | 1.955 Å | Babian-Kibala, E.; Chen, H.; Cotton, F. A.; Daniels, L. M.; Falvello, L. R.; Schmid, G.; Yao, Z. *Inorg. Chim. Acta* **1996**, *250*, 359. $[Al(NCCH_3)_6][SbCl_6]_3 \cdot 3CH_3CN$ |
| **Mean** | **1.946 Å/4 structures** | |

**Tin(II)**

*Five-coordination, square-pyramidal configuratrion with gap for anti-bonding orbital*

| CSD code | *d*(Sn-N) | Reference, compound formula |
|---|---|---|
| VUHQUN | 2.264+2.480 Å | Schorpp, M.; Heizmann, T.; Schmucker, M.; Rein, S.; Weber, S.; Krossing, I. *Angew. Chem., Int. Ed.* **2020**, *59*, 9453. $[Sn(NCCH_3)_5][Al(OC(CF_3)_3)_4]_2 \cdot$*1,2*-$C_6H_4F_2$ |

*Six-coordination, very distorted with gap for anti-bonding orbital*

| CSD code | *d*(Sn-N) | Reference, compound formula |
|---|---|---|
| HEDYUN | 2.530 Å | Schleep, M.; Hettich, C.; Kratzert, D.; Scherer, H.; Krossing, I. *Chem. Commun.* **2017**, *53*, 10914. $[Sn(NCCH_3)_6][Al(OC(CF_3)_3)_4]_2$ |

**Table S6.** Summary of structures of pyridine solvated metal ions in the solid state. Citations in purple text have not been included in the calculated mean bond distances.

**Copper(I)**

*Two-coordination, linear configuration,*

| CSD code | *d*(Cu-N) | Reference, compound formula |
|---|---|---|
| OGEJEP | 1.901 Å | Yang, W.; Lu, C.; Zhuang, H. *J. Chem. Soc., Dalton Trans.* **2002**, 2879. $[Cu(NC_5H_5)_4]_4[Mo_{12}O_{40}S]$ |

*Four-coordination, tetrahedral configuration,*

| CSD code | *d*(Cu-N) | Reference, compound formula |
|---|---|---|
| YAGMAX | 1.903 Å | Al Shamaileh, E.; Al-Far, R. CCDC deposition number 665843, **2016**. $[Cu(NC_5H_5)_4]I$ |
| ABAFER | 2.045 Å | Royappa, A. T.; Royappa, A. D.; Moral, R. F.; Rheingold, A. L.; Papoular, R. J.; Blum, D. M.; Duong, T. Q.; Stepherson, J. R.; Vu, O. D.; Chen, B.; Suchomel, M. R.; Golen, J. A.; Andre, G.; Kourkoumelis, N.; Mercer, A. D. ; Pekarek, A. M. ; Kelly, D. C. *Polyhedron* **2016**, 119, 563. $[Cu(NC_5H_5)_4](O_2CCO_2H)$ |
| BADTAB10 | 2.046 Å | Nilsson, K. ; Oskarsson, Å. *Acta Chem. Scand.* **1982**, *36*, 605. $[Cu(NC_5H_5)_4]ClO_4$ |
| GIGXOK01 | 2.046 Å | Coles, S. J.; Hursthouse, M. B.; Sengul, A.; Altin, S.; Kurt, O. *Acta Crystallogr., Sect. E* **2007**, *63*, m1734. $[Cu(NC_5H_5)_4]PF_6$ |
| GIGXOK | 2.062 Å | Horvat, G.; Portada, T.; Stilinovic, V.; Tomisic, V. *Acta Crystallogr., Sect. E* **2007**, *63*, m1734. $[Cu(NC_5H_5)_4]PF_6$ |
| **Mean** | **2.050 Å/4 structures** | |

**Silver(I)**

*Two-coordination, linear configuration,*

| CSD code | *d*(Ag-N) | Reference, compound formula |
|---|---|---|
| EWIZAN | 2.106 Å | Pliquett, D.; Schulz, P. S.; Heinemann, F. W.; Bause, A.; Wasserscheid, P. *Phys Chem. Chem. Phys.* **2016**, *18*, 28242. $[Ag(NC_5H_5)_2](PF_3(CF_3)_3)$ |
| NEVREM | 2.125 Å | Chen, C. Y.; Zeng, J. Y.; Lee, H. M. *Inorg, Chim. Acta* **2007**, *360*, 21. $[Ag(NC_5H_5)_2]BF_4$ |
| NEVRIQ | 2.129 Å | Chen, C. Y.; Zeng, J. Y.; Lee, H. M. *Inorg, Chim. Acta* **2007**, *360*, 21. $[Ag(NC_5H_5)_2]PF_6$ |
| LUKZAU | 2.130 Å | Bedin, M.; Karim, A.; Reitti, M; Carlsson, A.-C. C.; Topic, F.; Cetina, M.; Pan, F.; Havel, V.; Al-Ameri, F.; Sindelar, V.; Rissanen, J. K; Grafenstein, J.; Erdelyi, M. *Chem. Sci.* **2015**, *6*, 3746. $[Ag(NC_5H_5)_2]PF_6{\cdot}C_2H_2Cl_2$ |
| NEVRAI | 2.130 Å | Chen, C. Y.; Zeng, J. Y.; Lee, H. M. *Inorg, Chim. Acta* **2007**, *360*, 21. $[Ag(NC_5H_5)_2]ClO_4$ |

| | | |
|---|---|---|
| LULNOX | 2.132 Å | Bedin, M.; Karim, A.; Reitti, M; Carlsson, A.-C. C.; Topic, F.; Cetina, M.; Pan, F.; Havel, V.; Al-Ameri, F.; Sindelar, V.; Rissanen, J. K; Grafenstein, J.; Erdelyi, M. *Chem. Sci.* **2015**, *6*, 3746. $[Ag(NC_5H_5)_2]ClO_4{\cdot}CH_2Cl_2$ |
| ILUJOO | 2.134 Å | Ruiz, J.; Mosquera, M. E. G.; Garcia, G.; Patron, E.; Riera, V.; Garcia-Granda, S.; Van der Maelen, F. *Angew. Chem., Int. Ed.* **2003**, *42*, 4767. $[Ag(NC_5H_5)_2][Ru(CN(C(CH_3)_3)_4(P(C_6H_5)_2C(NC_5H_5)P(C_6H_5)_2)]_2(BF_4)_5{\cdot}3CH_2Cl_2{\cdot}2H_2O$ |
| QOCKID | 2.134 Å | Wang, G.-M.; Li, J.-H.; Li, Z.-X.; Wang, P.; Li, H. *Z. Anorg. Allg. Chem.* **2008**, *634*, 1192. $[Ag(NC_5H_5)_2]_2(H_4B_{10}O_{18})$ |
| LUKZIC | 2.137 Å | Bedin, M.; Karim, A.; Reitti, M; Carlsson, A.-C. C.; Topic, F.; Cetina, M.; Pan, F.; Havel, V.; Al-Ameri, F.; Sindelar, V.; Rissanen, J. K; Grafenstein, J.; Erdelyi, M. *Chem. Sci.* **2015**, *6*, 3746. $[Ag(NC_5H_5)_2]SbF_6{\cdot}C_2H_2Cl_2$ |
| LULNIR | 2.138 Å | Bedin, M.; Karim, A.; Reitti, M; Carlsson, A.-C. C.; Topic, F.; Cetina, M.; Pan, F.; Havel, V.; Al-Ameri, F.; Sindelar, V.; Rissanen, J. K; Grafenstein, J.; Erdelyi, M. *Chem. Sci.* **2015**, *6*, 3746. $[Ag(NC_5H_5)_2]BF{\cdot}C_2H_2Cl_2$ |
| IZEYAP | 2.141 Å | Jones, P. G.; Zerbe, E.-M. CCDC deposition number 1517185, **2016**. $[Ag(NC_5H_5)_2][Ag(N(SO_2CH_3)_2)_2]$ |
| IZEYUJ | 2.147 Å | Jones, P. G.; Zerbe, E.-M. CCDC deposition number 1517206, **2016**. $[Ag(NC_5H_5)_2](C_5H_6N)[Ag_2(N(SO_2C_6H_4)_4)_2$ |
| DITCEO | 2.153 Å | Dyason, J. C.; Healy, P. C.; Engelhardt, L. M.; White, A. H. *Aust. J. Chem.* **1985**, *38*, 1325. $[Ag(NC_5H_5)_2]_4[Ag(NC_5H_5)_4](ClO_4)_5$ |
| RAYWOE | 2.153 Å | Bowmaker, G. A.; Effendy; Lim, K. C.; Skelton, B. W.; Sukarianingsih, D.; White, A. H. *Inorg. Chim. Acta* **2005**, *358*, 4342. $[Ag(NC_5H_5)_2]_2(H_4B_{10}O_{18})$ |
| KELDAK | 2.160 Å | May, N. V.; Bayat, N.; Beres, K. A.; Bombicz, P.; Petrusevski, V. M.; Lendvay, G.; Farkas, A.; Kotai, L. *Inorganics* **2022**, *10*, 123. $[Ag(NC_5H_5)_2]ClO_4{\cdot}C_5H_5N$ |
| AGPYNO | 2.162 Å | Menchetti, S.; Rossi, G.; Tazzoli, V. *Rend. Ist. Lomb. Acc. Sci. Lett. A* **1970**, *104*, 309. $[Ag(NC_5H_5)_2]NO_3$ |
| BISVAC | 2.164 Å | Pouessel, J.; Thuery, P.; Berthet, J.-C.; Cantat, T. *Dalton Trans.* **2016**, *43*, 4415. $[Ag(NC_5H_5)_2]((C_6H_5)_4P)_2[Y(NO_2)_6]$ |
| BISTUU | 2.165 Å | Pouessel, J.; Thuery, P.; Berthet, J.-C.; Cantat, T. *Dalton Trans.* **2016**, *43*, 4415. $[Ag(NC_5H_5)_2]((C_6H_5)_4P)_2[Sm(NO_2)_6]$ |
| AGPYNO02 | 2.176 Å | Bedin, M.; Karim, A.; Reitti, M; Carlsson, A.-C. C.; Topic, F.; Cetina, M.; Pan, F.; Havel, V.; Al-Ameri, F.; Sindelar, V.; Rissanen, J. K; Grafenstein, J.; Erdelyi, M. *Chem. Sci.* **2015**, *6*, 3746. $[Ag(NC_5H_5)_2]NO_3$ |
| **Mean** | **2.143 Å/19 structures** | |

*Three-coordination, distorted triangular configuration,*

| CSD code | $d$(Ag-N) | Reference, compound formula |
| --- | --- | --- |
| WEFHUM | 2.307 Å | Costa, R.; Engle, J. T.; Ziegler, C. J. .*Porphyrins Phthalocyanines* **2012**, *16*, 175. $[Ag(NC_5H_5)_3][AgC_{35}H_{19}F_6N_7](NO_3)_2$ |

*Four-coordination, tetrahedral configuration,*

| CSD code | $d$(Ag-N) | Reference, compound formula |
| --- | --- | --- |
| DITCEO | 2.294 Å | Dyason, J. C.; Healy, P. C.; Engelhardt, L. M.; White, A. H. *Aust. J. Chem.* **1985**, *38*, 1325. $[Ag(NC_5H_5)_2]_4[Ag(NC_5H_5)_4](ClO_4)_5$ |
| POXREC | 2.304 Å | Kovacs, G. B.; May, N. V.; Bombicz, P. A.; Klebert, S.; Nemeth, P.; Menyhard, A.; Novodarszki, G.; Petrusevski, V.; Franguelli, F. P.; Magyari, J.; Beres, K.; Szilagyi, I. M.; Kotai, L. *RSC Adv.* **2019**, *9*, 28387. . $[Ag(NC_5H_5)_4][Ag(NC_5H_5)_2(OMnO_3)]MnO_4$ |
| IWOYIE | 2.310 Å | Hailmann, M.; Wolf, N.; Renner, R.; Schafer, T.; Hupp, B.; Steffen, A.; Finze, M. *Angew. Chem., Int. Ed.* **2016**, *55*, 10507. $[Ag(NC_5H_5)_4][Ag_7C_{67}H_{99}B_{44}N_{11}]\cdot 2C_5H_5N$ |
| BADTEF10 | 2.322 Å | Nilsson, K. ; Oskarsson, Å. *Acta Crystallogr., Sect. A* **1981**, *37*, C227b. $[Ag(NC_5H_5)_4]ClO_4$ |
| LUJLIP | 2.322 Å | McNelly, A. G.; Christensen, K. E.; Thompson, A. L. *Acta Crystallogr., Sect. E* **2024**, *80*, 1326. $[Ag(NC_5H_5)_4]PF_6$ |
| LUJLUB | 2.324 Å | McNelly, A. G.; Christensen, K. E.; Thompson, A. L. *Acta Crystallogr., Sect. E* **2024**, *80*, 1326. $[Ag(NC_5H_5)_4]SbF_6$ |
| PITLOW | 2.353 Å | Jenne, C.; Wegenerm B. *Z. Anorg. Allg. Chem.* **2018**, *644*, 1123. $[Ag(NC_5H_5)_4](C_3H_9B_{12}Cl_{11}N)$ |
| **Mean** | **2.318 Å/7 structures** | |

**Gold(I)**

*Two-coordination, linear configuration,*

| CSD code | $d$(Ag-N) | Reference, compound formula |
| --- | --- | --- |
| UJOQAN | 2.021 Å | Corbo, R.; Ryan, G. F.; Haghighatbin, M. A.; Hogan, C. F.; Wilson, D. J. D.; Hulett, M. D.; Barnard, P. J.; Dutton, J. L. *Inorg. Chem.* **2016**, *55*, 2830. $[Au(NC_5H_5)_2]BF_4$ |
| AXENUO01 | 2.025 Å | Doring, C.; Jones, P. G. *Z. Naturforsch., Sect. B* **2014**, *69*, 1315. $[Au(NC_5H_5)_2][Au(SCN)_2]$ |
| **Mean** | **2.023 Å/2 structures** | |

**Mercury(II)**

*Six-coordination, second order Jahn-Teller distorted octahedral configuration,*

| CSD code | $d$(Ag-N) | Reference, compound formula |
| --- | --- | --- |
| VINKEI01 | 2.445 Å | Åkesson, R.; Sandström, M.; Stålhandske, C. I.; Persson, I. *Acta Chem. Scand.* **1991**, *45*, 2830. $[Hg(NC_5H_5)_6](CF_3SO_3)_2$ |

**Table S7.** Summary of M-P bond distances homoleptic copper(I), silver(I) and gold(I) complexes of trialkyl- and triphenyl phosphites and phosphines in the solid state and solution. The value within parenthesis denotes the number of reported structures.

**Phosphites**

| | Copper(I) | | Silver(I) | | Gold(I) | |
|---|---|---|---|---|---|---|
| | Solid state | Solution | Solid state | Solution | Solid state | Solution |
| Methyl phosphite | 2.244 | 2.245 | | | | |
| Ethyl phosphite | 2.245 | 2.258 | | 2.483 | | 2.367 |
| Iso-Propyl phosphite | 2.279 | 2.258 | | | | |
| n-Butyl phosphite | | 2.249 | | 2.513 | | |
| Phenyl phosphite | 2.255 | | 2.495 | 2.485 | | |

**Phosphines**

| | | Copper(I) | | Silver(I) | | Gold(I) | |
|---|---|---|---|---|---|---|---|
| | *N* | Solid state | Solution | Solid state | Solution | Solid state | Solution |
| Trialkyl phosphines | 2 | 2.205 (6) | - | 2.386 (15) | - | 2.311 (38) | - |
| | 3 | - | | - | 2.528 | - | 2.399 |
| | 4 | 2.270 (28) | 2.278 | 2.603 (1) | - | - | |
| Triphenyl phosphine | 2 | - | - | - | - | 2.309 (20) | - |
| | 3 | 2.286 (12) | - | 2.493 (3) | - | 2.382 (11) | - |
| | 4 | - | - | 2.629 (33) | - | 2.528 (2) | - |

**Table S8**. Electronegativity values, $\chi_m$, from ref. 1, ionic radius, $r$/Å, from ref. 2, and the calculated covalent index, $(\chi_m)^2r$, of metal ions.

| Metal ion | $\chi_m$ | $r$ | $(\chi_m)^2r$ |
|---|---|---|---|
| $Li^{+}$ | 0.98 | 0.59 | 0.57 |
| $Na^{+}$ | 0.93 | 1.02 | 0.88 |
| $Mg^{2+}$ | 1.31 | 0.72 | 1.24 |
| $Ca^{2+}$ | 1.00 | 1.12 | 1.12 |
| $Sr^{2+}$ | 0.95 | 1.26 | 1.14 |
| $Ba^{2+}$ | 0.89 | 1.47 | 1.16 |
| $La^{3+}$ | 1.10 | 1.216 | 1.47 |
| $Sm^{3+}$ | 1.17 | 1.125 | 1.54 |
| $Zr^{4+}$ | 1.33 | 0.84 | 1.49 |
| $V^{2+}$ | 1.63 | 0.79 | 2.10 |
| $Cr^{3+}$ | 1.66 | 0.615 | 1.69 |
| $Mn^{2+}$ | 1.55 | 0.83 | 1.99 |
| $Fe^{2+}$ | 1.83 | 0.78 | 2.61 |
| $Ru^{2+}$ | 2.2 | 0.77 | 3.73 |
| $Co^{2+}$ | 1.88 | 0.745 | 2.63 |
| $Rh^{3+}$ | 2.28 | 0.665 | 3.46 |
| $Ir^{3+}$ | 2.2 | 0.68 | 3.29 |
| $Ni^{2+}$ | 1.91 | 0.69 | 2.52 |
| $Pd^{2+}$ | 2.2 | 0.64 | 3.10 |
| $Pt^{2+}$ | 2.2 | 0.60 | 2.90 |
| $Cu^{2+}$ | 1.90 | 0.73 | 2.63 |
| $Ag^{+}$ | 1.93 | 1.00 | 3.72 |
| $Zn^{2+}$ | 1.65 | 0.74 | 2.01 |
| $Cd^{2+}$ | 1.69 | 0.95 | 2.71 |
| $Al^{3+}$ | 1.61 | 0.535 | 1.39 |
| $Ga^{3+}$ | 1.81 | 0.62 | 2.03 |
| $In^{3+}$ | 1.78 | 0.80 | 2.53 |
| $Tl^{3+}$ | 1.80 | 0.885 | 2.87 |

Ref. 1. *Handbook of Chemistry and Physics*, Ed. Haynes, W. M. CRC Press, Taylor and Francis Group, Boca Raton, USA, p. 9-97.
Ref. 2 Shannon, R. D. Revised Effective Ionic Radii and Systematic Studies of Interatomic Distances in Halides and Calcogenides. *Acta Crystallogr., Sect. A* **1976**, *32*, 751-767. https://doi.org/10.1107/S0567739476001551

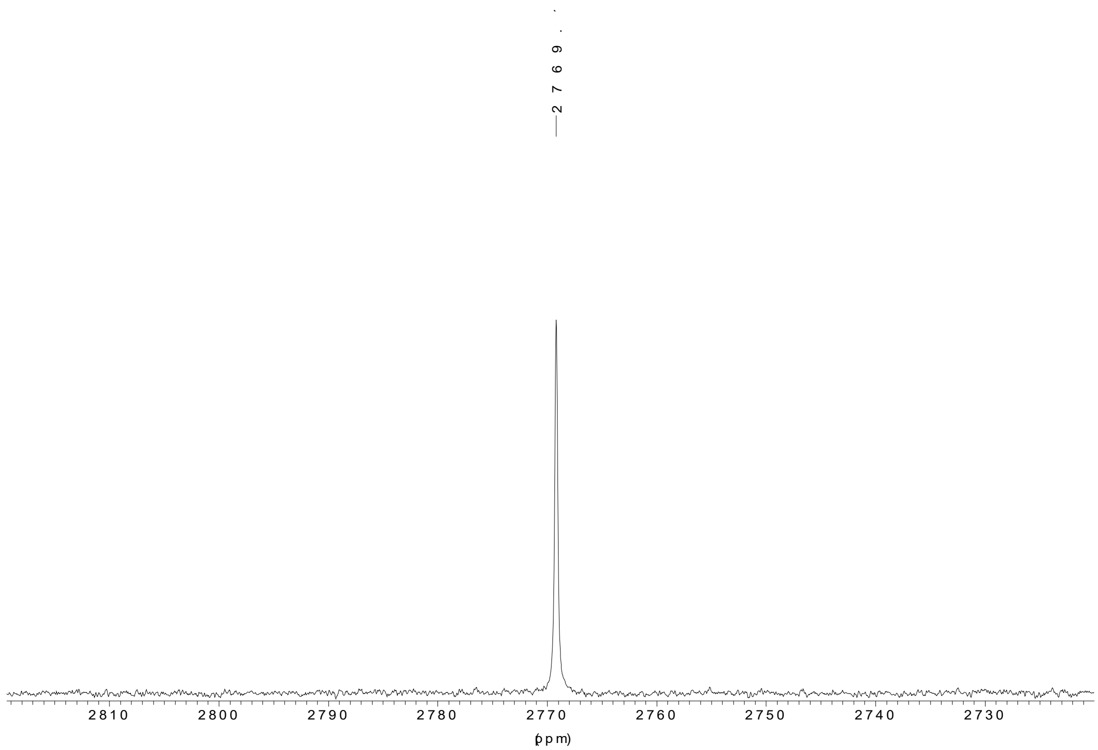


**Figure S1**. $^{205}Tl$ NMR spectrum of a 0.046 M solution of hexaamminethallium(III) perchlorate in liquid ammonia at room temperature.

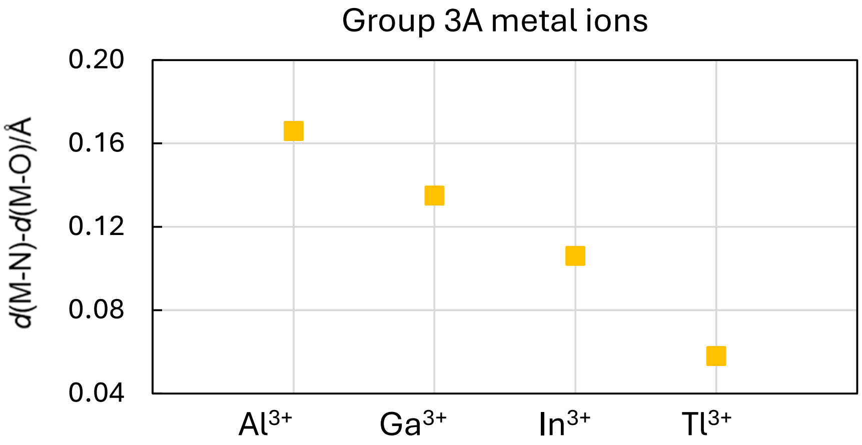


**Figure S2.** Difference in M-N and M-O bond distances in group 3A metal ion ammine solvates and hydrates.

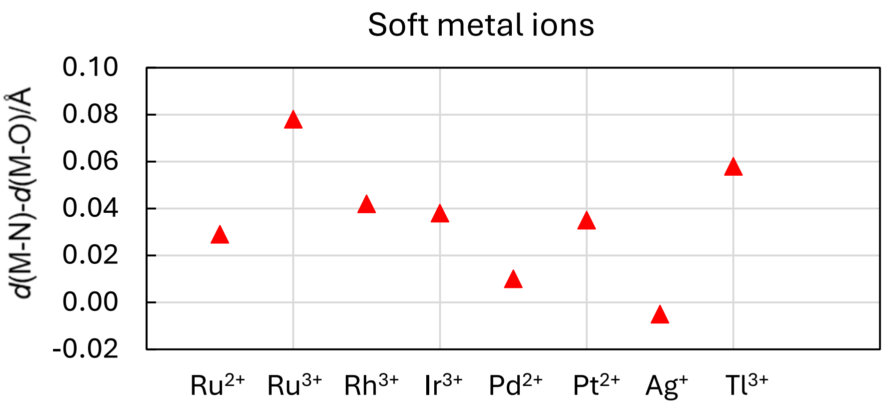


**Figure S3**. Difference in M-N and M-O bond distances in the softest metal ion ammine solvates and hydrates.
